# Immunomodulators and Advanced Therapies for Induction of Remission in Crohn’s Disease: A Systematic Review and Network Meta-Analysis

**DOI:** 10.1093/ibd/izaf191

**Published:** 2025-09-19

**Authors:** Vasiliki Sinopoulou, Morris Gordon, Shiyao Liu, Daniel Arruda Navarro Albuquerque, Aderonke Ajiboye, Sudheer Kumar Vuyyuru, Shellie Radford, Gordon Moran

**Affiliations:** School of Medicine, University of Central Lancashire, Preston, United Kingdom; School of Medicine, University of Central Lancashire, Preston, United Kingdom; School of Medicine, University of Central Lancashire, Preston, United Kingdom; School of Medicine, University of Central Lancashire, Preston, United Kingdom; School of Medicine, University of Central Lancashire, Preston, United Kingdom; Faculty of Medicine & Health Sciences, University of Nottingham, Nottingham, United Kingdom; Faculty of Medicine & Health Sciences, University of Nottingham, Nottingham, United Kingdom; Faculty of Medicine & Health Sciences, University of Nottingham, Nottingham, United Kingdom

**Keywords:** gastroenterology, inflammatory bowel disease, evidence synthesis

## Abstract

**Background:**

Previous reviews for Crohn’s disease (CD) treatment have rarely considered advanced and immunomodulator medical therapies together. Our aim was to compare all therapies for efficacy and safety in induction of remission.

**Methods:**

We searched databases up to June 2025. Our outcomes were clinical remission and response, endoscopic remission, and safety outcomes. We performed network meta-analyses and estimated risk ratios (RR) and 95% CIs. We used GRADE to assess certainty of results, and surface under the cumulative ranking curve for ranking treatments.

**Results:**

A total of 79 RCTs with 20 724 participants were included. Interventions ranged from 2 to 30 weeks. There was moderate GRADE certainty of effectiveness over placebo for clinical remission for combination of adalimumab with thiopurines (RR, 2.87; 95% CI, 1.99-4.14; RD (Risk difference)  = 35.3%; NNT (Number needed to treat) = 3, large magnitude), guselkumab (RR, 2.5; 95% CI, 1.95-3.21; RD = 28.4%; NNT = 4, moderate magnitude, adalimumab (RR, 2.46; 95% CI, 1.84-3.29; RD = 27.6% NNT = 4, moderate magnitude), combination of infliximab with thiopurines (RR, 2.43; 95% CI, 1.71-3.44; RD = 27%; NNT = 4, moderate magnitude), and ustekinumab (RR, 2.04; 95% CI, 1.69-2.46; RD = 19.6% NNT = 5, small magnitude). For endoscopic remission, there was moderate GRADE certainty of effectiveness for risankizumab (RR, 3.48; 95% CI, 2.18-5.58; RD = 17.4%, moderate magnitude). The certainty on safety varied, but treatments appear generally safe in the short term.

**Conclusion:**

Combination of anti-tumor necrosis factors (anti-TNFs) and immunomodulators followed by anti-TNF monotherapy had large effect size with moderate certainty for the induction of clinical remission. More novel therapies appear to have similar effect sizes but with increased imprecision of the estimates.

Key Messages *What is already known?*

Advanced therapies brought on a revolution in Crohn’s disease treatment, which consisted of corticosteroids and immunomodulators. Network meta-analyses compare and rank multiple therapies; however in gastroenterology, their results are not appropriately assessed for certainty and magnitude of effect.

*What is new here?*

We conducted network meta-analyses and GRADE-assessed the certainty of our results. We used pre-agreed thresholds to determine magnitude effect sizes.

*How can this study help patient care?*

Combination of anti-TNFs and immunomodulators followed by anti-TNF monotherapy appear to be largely effective, with moderate GRADE certainty. Newer therapies appear to have similar effect sizes but the current evidence is limited. There were no clear safety concerns, however evidence is sparse.

## Introduction

Crohn’s disease (CD) is a complex immune mediated inflammatory bowel disease (IBD), affecting approximately 1 in 300 patients in countries with high prevalence such as the United States of America and the United Kingdom and is expected to increase in future.^1^^,^^2^ Recent studies have observed increasing incidence in developing countries indicating a raising burden all over the world.^3^

Conventional therapies such as corticosteroids and immunomodulators played a central role in the management of IBD before the development of advanced targeted therapies. The management principles since then have been transforming, since the approval of the first biologic therapy targeting tumor necrosis factor (TNF), infliximab, in early 2000s. Recent scientific advances enabled us to understand the immune pathways driving inflammation in CD, leading to identification of various potential molecular therapeutic targets. Consequently, various classes of advanced therapies namely anti-integrins, anti-interleukin (IL)-12/23p49, anti-IL23p19 and oral small molecules (JAKi), have been developed in the last 2 decades. With the availability of multiple treatment options, the choice of therapy can be challenging in clinical practice. While head-to-head blinded controlled trials are the ideal method for comparing the efficacy of different drugs, practical limitations make it challenging to conduct multiple trials encompassing all available treatment options. To date, only 3 head-to-head trials have been completed in CD.^4–6^

A network meta-analysis (NMA) combines direct and indirect comparison of different therapies which cannot be achieved with pairwise meta-analyses. The evolving data landscape necessitates updated analyses that compare immunomodulators or combination of immunomodulators with advanced therapies, something that has not been attempted previously. Furthermore, there has been increasing recognition of endoscopic outcomes along with clinical outcomes, and this has been advocated by international organizations.^7^ Comparative efficacy of advanced therapies in induction of endoscopic outcomes is as important as clinical efficacy for therapeutic decision-making. Similarly, safety of these therapies along with efficacy plays a key role in treatment choice.

The aim of this NMA was to comparatively assess clinical, endoscopic, and safety outcomes for all available advanced therapies and immunomodulators, including combination therapies, for induction of remission in CD, thereby aiding therapeutic decision-making.

## Materials and Methods

A protocol for this review was made publicly available prospectively through the University of Central Lancashire’s online repository.^8^ We followed the PRISMA reporting guidelines and AMSTAR 2 standards.^9^^,^^10^ The present work was exempt from ethics approval.

### Literature search

We searched MEDLINE, EMBASE, Cochrane Library, and Web of Science from inception to February 2024 (supplementary material, Appendix 1). We also scanned the included studies of previously published systematic reviews on medical therapies in CD and applied our inclusion and exclusion criteria.^11^^,^^12^ There were no limits on follow-up time, language, setting, gender, disease activity, disease duration, disease location, or any other factors.

### Study selection

Trials on adult participants (≥18 years of age) with active CD as defined by the included studies were included in the NMA for induction of remission. Phase 3 and 2b randomized control trials (RCTs) comparing advanced therapies with any other active comparator, placebo or no treatment for induction of remission in CD, cluster RCTs, and the precrossover phases of cross-over RCTs were considered for inclusion. RCT results published only as abstracts, press release, or as results posted in a trial register were included if outcome data were available. Nonrandomized or quasi-randomized trials or parts of trials, such as nonrandomized induction phase, long-term follow-ups, or nonrandomized control groups were excluded. Trials comparing only different dosages of the same treatment, top-down vs bottom-up strategies, dose escalation, or trough levels were excluded.

The included interventions comprised all advanced therapies and their biosimilars including, TNF-α inhibitors, anti-integrins, IL-12/23p40 antagonists, IL-23p19 antagonists, JAK inhibitors, and others. All types of administration routes and dose regimens were considered for inclusion. A threshold was set for populations with mixed exposure to azathioprine/6-mercaptopurine in which, if the prevalence of azathioprine/6-mercaptopurine use was more than 50% of participants, the population was deemed as receiving combination therapy of the advanced therapy with azathioprine/6-mercaptopurine.

Title/abstract and full-text screening were performed in duplicate by 2 experienced reviewers (M.G., V.S.), and disagreements were resolved by discussion and consensus with a senior author.

### Outcomes

The primary outcome was the proportion of patients who achieved clinical remission, as defined by the original study (eg, a CDAI score <150). If the outcomes were reported at more than 1 time point, we used the one defined as primary by the original studies. The secondary outcomes were clinical response, endoscopic remission, withdrawals due to adverse events (WAEs), serious adverse events (SAEs), and total adverse events (TAEs).

### Outcome thresholds

We used previously agreed upon outcome thresholds for the assessment of imprecision of magnitude effects which were decided via an online Delphi survey of IBD stakeholders (clinicians, nurses, patients) (Table S4).^13–15^ Pre-agreed thresholds are the recommended approach for assessing confidence in NMA and guidelines.^16^

### Data extraction and risk of bias assessment

Data extraction included demographic and baseline characteristics, intervention details, and outcome data. Risk of bias assessment was assessed using the Cochrane risk of bias 1 tool.^17^ They were performed in duplicate by 2 experienced reviewers from the author team (M.G., V.S., S.L., D.A.N.A., A.A.) and disagreements were resolved by discussion and consensus with a senior author.

### Statistical analysis

All review outcomes were dichotomous and were expressed in risk ratios (RRs) with corresponding 95% CIs, using a modified intention-to-treat and random effects model analysis, in which all randomized participants were included in analyses, irrespective of whether they received treatment or not. The unit of analysis was the participant for all outcomes. We performed analyses on a modified intention-to-treat basis, where missing study data were counted as treatment failures or potential withdrawals due to adverse effects.^8^ In studies were multiple dosages of the same treatment were assigned as different intervention groups, the intervention groups of the same treatment were combined for analysis. Analyses were conducted for the data defined by the authors as primary end points or end of the randomized study data, due to the variation in the end points reported by the included studies, which would not allow for connected networks of the similar end points to be formed.

Frequentist framework network meta-analysis methodology was used.^18^ We assessed the assumption of transitivity by comparing the distribution of potential effect modifiers across the pairwise comparisons.^8^ Heterogeneity was assessed statistically using the I^2^ statistic for each pairwise comparison and with the loop-specific approach for the direct and indirect estimates.^8^ Surface under the cumulative ranking curve (SUCRA) was used to rank treatments, and placebo was used as the comparison treatment for all other treatments. Funnel plots were used to assess publication bias for pairwise analyses with at least 10 studies. The presence of small-study effects was assessed via comparison-adjusted funnel plots. Statistical analyses were performed using the R statistical software and netmeta package.^19^^,^^20^

### Subgroup and sensitivity analyses

The preplanned subgroup analyses for the primary outcome of clinical remission were:

- Patients naïve to advanced treatments (>50% of all participants being naïve) versus patients that had failed advanced treatments previously (>50% of all participants being not naïve)
- Separating the dosages for each treatment that were combined in the main analysis
- Per identical time points of measurement of the outcome

The preplanned sensitivity analysis for the primary outcome of clinical remission was removal of studies where the population is mixed regarding the use of azathioprine/6-mercaptopurine (ie, if >20% of all participants on concomitant azathioprine, 6-mercaptopurine, studies were removed)

Two additional unplanned sensitivity analyses were conducted to test the effect of advanced/biologic treatments specifically: one where studies including exclusively patients exposed to azathioprine and 6-mercaptopurinewere removed, and another where studies published before the beginning of the biologic treatment era were removed. We chose 2003 as the beginning of this era, the year when infliximab was approved by the FDA for the treatment of CD.

Three unplanned sensitivity analyses were conducted for the outcome of endoscopic remission: for studies with up to 26 weeks of follow-up, an endoscopic remission definition of SES-CD score ≤4, and patients previously exposed to biologic treatment.

### GRADE assessment for the certainty of evidence

The GRADE framework was used to assess the certainty of the evidence.^21^ The direct and indirect evidence certainty was assessed on risk of bias, inconsistency/heterogeneity, indirectness, and publication bias. Following that, the network evidence certainty was assessed on imprecision and incoherence, taking into account the percentage of contribution of the direct and indirect evidence. Two review authors (M.G., V.S.) rated the certainty, and disagreements were resolved by discussion and consensus. The evidence was rated as “high,” “moderate,” “low,” or “very low” according to the GRADE framework. The results were presented using “GRADEing of Relative Effect Diagram of NMA” (GORDON) plots to aid the interpretation and integration of efficacy, ranking, magnitude and certainty data,^22^ which represents the magnitude and certainty of results ranked by magnitude of effect within a given certainty class. When outcomes were of very low certainty, meaning conclusions should not be drawn (regardless of the magnitude or absolute effects seen), they were not included in these plots.

GRADE was used in combination with SUCRA to rank treatments. In the summary of findings tables (Table S5), treatments are ranked from higher to lower SUCRA probability and their corresponding GRADE certainty and estimates are presented. In the abstract, results section, and graphical plot figures, treatments are presented from high to low GRADE certainty and ranked by SUCRA probability within their respective GRADE assessment rating (high, moderate, or low).

## Results

**Figure 1.**
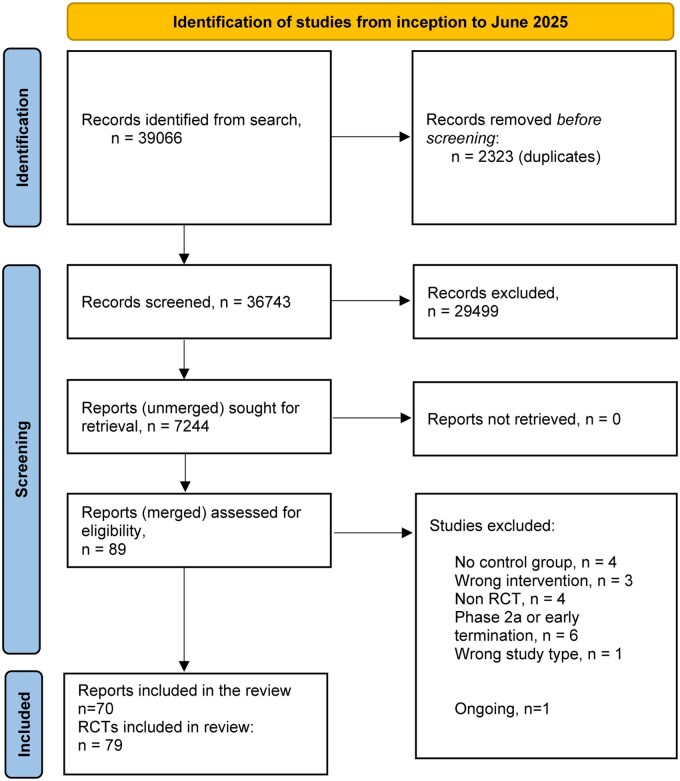
PRISMA flow diagram.

A total of 39 066 records were identified by the systematic search and its updates. Eighty-nine records (associated reports of the same RCT were merged) were assessed, and 19 were excluded with reasons which resulted in 79 included RCTs (*n* = 20 724, 70 published reports) (Figure 1;  Appendix 2, Tables S1, S3). Available data on potential effect modifiers were evenly distributed among studies (Tables S1, S2). No cluster trials were identified and no crossover trials reported precrossover data for analysis, per our inclusion criteria.

Follow-up ranged between 2 and 30 weeks. Four studies with azathioprine/6-mercaptopurine use for more than 50% of participants were deemed as receiving combination therapy of the advanced therapy with azathioprine/6-mercaptopurine. Clinical disease activity at baseline was moderate to severe, with 5 studies also including patients with mild activity. In most studies, clinical disease activity was measured on the Crohn's disease activity index (CDAI) scale, remission defined as CDAI <150, and clinical response as a reduction of 70 or 100 points on the CDAI scale. Of the studies that reported baseline endoscopic activity, only one included mild disease activity patients alongside moderate and severe. Endoscopic activity was mostly measured on the SES-CD scale and endoscopic remission definitions based around it. Thirty-seven studies included fully or mostly biologically naïve patients (≥50%) and 27 fully or mainly exposed patients (>50%) (Tables S1, S2). More details on age, sex, dosages, regimens, disease activity scores and remission definitions, concomitant medications and duration of the studies can be found in Tables S1 and S2. The similarities of the populations’ baseline characteristics, the characteristics of the interventions and comparators, and the included outcome definitions were deemed sufficient to support the assumption of transitivity.

A summary of the risk of bias assessment and detailed judgement reasons for all included RCTs can be found in the supplementary material (Supplement 2: Risk of bias assessment and supporting judgements). Summary of findings tables and GRADE decisions for all outcomes can be found in the supplementary material in Tables S5, network plots in Figure S1, network forest plots, SUCRA probabilities, and direct/indirect/network estimates forest plots in Figure S2, and subgroup and sensitivity analyses in Figure S3.

Comparison-adjusted funnel plots for the assessment of small study effects were balanced (Figure S4). No pairwise comparisons included 10 or more studies; therefore publication bias funnel plots were not generated.

Sixty-nine (87%) of the included RCTs were either fully or partially funded by pharmaceutical companies. Two had no funding, of which one involved an advanced therapy, and the rest, of which 2 involved advanced therapies, did not mention funding details, meaning 95% of RCTs on advanced therapies declared funding from the pharmaceutical industry (Table S1).

### Induction of clinical remission

**Figure 2.**
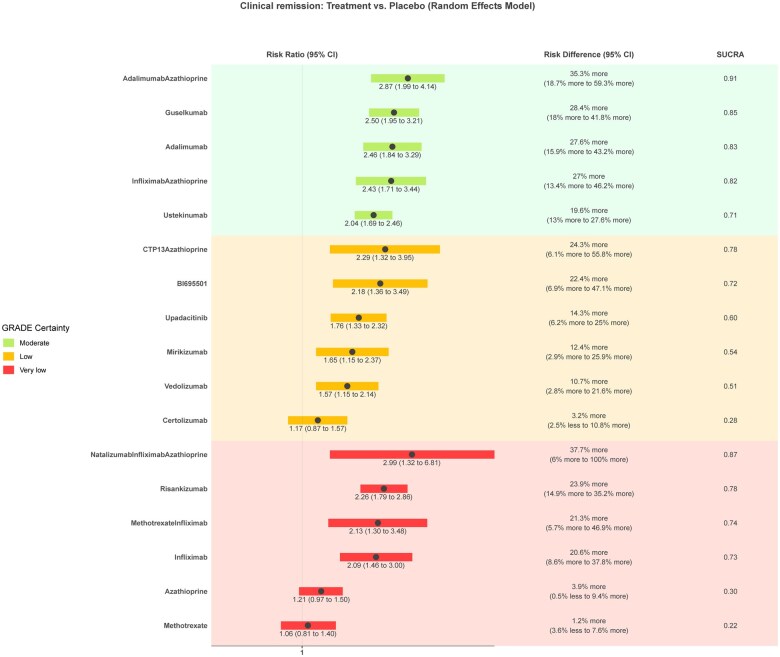
GORDON plot of clinical remission network results with placebo as comparison, for clinically relevant treatments.

Sixty-five studies (*n* = 19 854) assessing 32 interventions were included in the clinical remission network meta-analysis (Table S2, Figure 5A). Placebo had a mean rate of 19% (range 3%-89%) at inducing clinical remission. Network heterogeneity was 28.3% (I^2^). Table 1 and Figure 2 provide a summary and graphical presentation of the results for this outcome. GORDON plots containing relative effects and risk differences compared to placebo for all treatments, SUCRA probability rankings, and GRADE certainty for all treatments can be found in Supplement 3.

**Table 1.** Summary of findings table for the outcome of clinical remission. The reasons for the GRADE judgements can be found in supplementary Table S5. Abbreviations: CI, confidence interval; NA, not applicable; NNT, number needed to treat; RR, risk ratio.

Clinical Remission							
Patient or population: people with active Crohn's disease							
Settings: hospital setting							
Intervention: biologics/purine analogues/methotrexate							
Comparison: placebo							
Treatment	Network evidence		Anticipated absolute effects for network estimate			NNT (95% CI)	Notes
	RR	Certainty	Risk with Placebo^a^	Risk with Agent^b^	% Risk Difference with Agent^c^		
	(95% CI)						
Adalimumab and purine analogues	2.87 (1.99 to 4.14)	Moderate	189 per 1000	542 per 1000 (376 to 782)	35.3% more (18.7% more to 59.3% more)	3 (2 to 5)	It is probably better than placebo by a large effect size (ranging from small to large)
		⊕⊕⊕⊖					
Natalizumab with Infliximab and purine analogues	2.99 (1.32 to 6.81)	Very low	189 per 1000	565 per 1000 (249 to 1000)	37.6% more (6% more to 100% more)	NA	The evidence is very uncertain
		⊕⊖⊖⊖					
Guselkumab	2.5 (1.95 to 3.21)	Moderate	189 per 1000	473 per 1000 (369 to 607)	28.4% more (18% more to 41.8% more)	4 (2 to 6)	It's probably better than placebo by a moderate effect size (ranging from small to large)
		⊕⊕⊕⊖					
Adalimumab	2.46 (1.84 to 3.29)	Moderate	189 per 1000	465 per 1000 (348 to 622)	27.6% more (15.9% more to 43.3% more)	4 (2 to 6)	It's probably better than placebo by a moderate effect size (ranging from small to large)
		⊕⊕⊕⊖					
Humicade	3.14 (0.7 to 13.94)	Very low	189 per 1000	593 per 1000 (132 to 1000)	40.4% more (5.7% less to 100% more)	NA	The evidence is very uncertain
		⊕⊖⊖⊖					
Infliximab with purine analogues	2.43 (1.71 to 3.44)	Moderate	189 per 1000	459 per 1000 (323 to 650)	27% more (13.4% more to 46.1% more)	4 (2 to 7)	It's probably better than placebo by a moderate effect size (ranging from small to large)
		⊕⊕⊕⊖					
Risankizumab	2.26 (1.79 to 2.86)	Very low	189 per 1000	427 per 1000 (338 to 541)	23.8% more (14.9% more to 35.2% more)	NA	The evidence is very uncertain
		⊕⊖⊖⊖					
CTP13 with purine analogues	2.29 (1.32 to 3.95)	Low	189 per 1000	433 per 1000 (249 to 747)	24.4% more (6% to 55.8% more)	NA	It may be better than placebo by a moderate effect size (ranging from trivial to large)
		⊕⊕⊖⊖					
BI695501	2.18 (1.36 to 3.49)	Low	189 per 1000	412 (257 to 660)	22.3% more (6.8% more 47.1% more)	NA	It may be better than placebo by a moderate effect size (ranging from trivial to large)
		⊕⊕⊖⊖					
MethotrexateInfliximab	2.13 (1.3 to 3.48)	Very low	189 per 1000	403 per 1000 (246 to 658)	21.4% more (5.7% more to 46.9% more)	NA	The evidence is very uncertain
		⊕⊖⊖⊖					
Infliximab	2.09 (1.46 to 3.00)	Very low	189 per 1000	395 per 1000 (276 to 567)	20.6% more (8.7% more to 37.8% more)	NA	The evidence is very uncertain
		⊕⊖⊖⊖					
Ustekinumab	2.04 (1.69 to 2.46)	Moderate	189 per 1000	386 per 1000 (319 to 465)	19.7% more (13% more to 27.6% more)	5 (4 to 8)	It's probably better than placebo by a small effect size (ranging from small to moderate)
		⊕⊕⊕⊖					
Upadacitinib	1.76 (1.33 to 2.32)	Low	189 per 1000	333 per 1000 (251 to 438)	14.4% more (6.2% more to 24.9% more)	NA	It may be better than placebo by a small effect size (ranging from trivial to moderate)
		⊕⊕⊖⊖					
Mirikizumab	1.65 (1.15 to 2.37)	Low	189 per 1000	312 per 1000 (217 to 448)	12.3% more (2.8% more to 25.9% more)	NA	It may be better than placebo by a small effect size (ranging from trivial to moderate)
		⊕⊕⊖⊖					
Vedolizumab	1.57 (1.15 to 2.14)	Low	189 per 1000	297 per 1000 (217 to 404)	10.8% more (2.8% more to 21.5% more)	NA	It may be better than placebo by a small effect size (ranging from trivial to moderate)
		⊕⊕⊖⊖					
Filgotinib	1.55 (1.17 to 2.06)	Low	189 per 1000	293 per 1000 (221 to 389)	10.4% more (3.2% to 20% more)	NA	It may be better than placebo by a small effect size (ranging from trivial to small)
		⊕⊕⊖⊖					
Natalizumab	1.37 (1.07 to 1.75)	Low	189 per 1000	259 per 1000 (202 to 331)	7% more (1.3% more to 14.2% more)	NA	It may be better than placebo by a trivial effect size (ranging from trivial to small)
		⊕⊕⊖⊖					
Etrolizumab	1.3 (0.87 to 1.95)	Very Low	189 per 1000	246 per 1000 (164 to 369)	5.7% more (2.5% less to 18% more)	NA	The evidence is very uncertain
		⊕⊖⊖⊖					
Purine analogues	1.21 (0.97 to 1.5)	Very low	189 per 1000	229 per 1000 (183 to 284)	4% more (0.6% less to 9.5% more)	NA	The evidence is very uncertain
		⊕⊖⊖⊖					
Tesnatilimab	1.19 (0.77 to 1.83)	Very low	189 per 1000	225 pe 1000 (146 to 346)	3.6% more (4.3% less to 15.7% more)	NA	The evidence is very uncertain
		⊕⊖⊖⊖					
Certolizumab	1.17 (0.87 to 1.57)	Low	189 per 1000	221 per 1000 (164 to 297)	3.2% more (2.5% less to 10.8% more)	NA	It may be the same as placebo with an effect that can range from trivially less than placebo to small effect more than placebo
		⊕⊕⊖⊖					
Tofacitinib	1.18 (0.8 to 1.73)	Low	189 per 1000	223 per 1000 (151 to 327)	3.4% more (3.8% less to 13.8% more)	NA	It may be the same as placebo with an effect that can range from trivially less than placebo to small effect more than placebo
		⊕⊕⊖⊖					
Onercept	1 (0.5 to 2.01)	Very low	189 per 1000	189 per 1000 (95 to 380)	0% (9.5% less to 19.1% more)	NA	The evidence is very uncertain
		⊕⊖⊖⊖					
Methotrexate	1.06 (0.81 to 1.4)	Very low	189 per 1000	200 per 1000 (153 to 265)	1.1% more (3.6% less to 7.6% more)	NA	The evidence is very uncertain
		⊕⊖⊖⊖					
Andecaliximab	0.76 (0.33 to 1.78)	Very low	189 per 1000	144 per 1000 (62 to 336)	4.5% less (12.7% less to 14.7% more)	NA	The evidence is very uncertain
		⊕⊖⊖⊖					
Etanercept	0.43 (0.09 to 2.19)	Very low	189 per 1000	81 per 1000 (17 to 414)	10.8% less (17.2% less to 22.5% more)	NA	The evidence is very uncertain
		⊕⊖⊖⊖					
Apilimod mesylate	0.67 (0.34 to 1.35)	Low	189 per 1000	127 per 1000 (64 to 255)	6.2% less (12.5% less to 6.6% more)	NA	It may be the same as placebo with an effect that can range from a small effect less than placebo to a trivial effect more than placebo
		⊕⊕⊖⊖					

GRADE Working Group grades of evidence. High certainty: we are very confident that the true effect lies close to that of the estimate of the effect. Moderate certainty: we are moderately confident in the effect estimate; the true effect is likely to be close to the estimate of the effect, but there is a possibility that it is substantially different. Low certainty: our confidence in the effect estimate is limited; the true effect may be substantially different from the estimate of the effect. Very low certainty: we have very little confidence in the effect estimate; the true effect is likely to be substantially different from the estimate of effect. Abbreviations: CI, confidence interval; RR, risk ratio.

a The risk with placebo has been calculated based on the cumulative placebo rates of all studies with a placebo arm.

b The risk with treatment has been calculated by multiplying the risk with control with the RR (95% CI). If the calculation results in more than 1000 per 1000 people, the number has been capped to 1000. Numbers have been rounded up to the closest whole number.

c The percentage of risk difference was calculated by subtracting the risk with control from the risk with treatment (95% CI) and dividing by 10. If the calculation results in more than 100%, the number was capped to 100%. Numbers have been rounded up to the closest whole number.

* Red coloring indicates the treatment crosses the line of no effect.

None of the interventions were rated high for GRADE certainty. Five treatments were rated at moderate GRADE certainty and are probably more effective in inducing clinical remission compared to placebo. In order of SUCRA ranking, these are combination of adalimumab with azathioprine/6-mercaptopurine (RR, 2.87 [95% CI, 1.99-4.14]; NNT = 3 [95% CI, 2-5] large effect magnitude), guselkumab (RR, 2.5 [95% CI, 1.95-3.21]; NNT = 4 [95% CI, 2-6] moderate effect magnitude), adalimumab (RR, 2.46 [95% CI, 1.84-3.29] NNT = 4 [95% CI, 2-7] moderate magnitude), combination of infliximab with azathioprine/6-mercaptopurine (RR, 2.43 [95% CI, 1.71-3.44] NNT = 4 [95% CI, 2-7] moderate magnitude), and ustekinumab (RR, 2.04 [95% CI, 1.69-2.46] NNT = 5 [95% CI, 4-8] small magnitude).

Seven treatments were rated at low GRADE certainty and are maybe more effective in inducing clinical remission compared to placebo. In order of SUCRA ranking, these are combinations of CTP13 and azathioprine/6-mercaptopurine (RR, 2.29; 95% CI, 1.32-3.95, moderate magnitude), BI695501 (RR, 2.18; 95% CI, 1.36-3.49, moderate magnitude), upadacitinib (RR, 1.76; 95% CI, 1.33-2.32, small magnitude), mirikizumab (RR, 1.65; 95% CI, 1.15-2.37, small magnitude), vedolizumab (RR, 1.57; 95% CI, 1.15-2.06, small magnitude), filgotinib (RR, 1.55; 95% CI, 1.17-2.06, small magnitude), and natalizumab (RR, 1.37; 95% CI, 1.07-1.75, trivial magnitude). Three treatments were rated at low GRADE certainty and are maybe similar to placebo at inducing clinical remission: certolizumab (RR, 1.17; 95% CI, 0.87-1.57), tofacitinib (RR, 1.18; 95% CI, 0.8-1.73), and apilimod mesylate (RR, 0.67; 95% CI, 0.34-1.35).

The results for 12 of the treatments had very low GRADE certainty and no conclusions can be drawn about them (Table 1).

**Figure 3.**
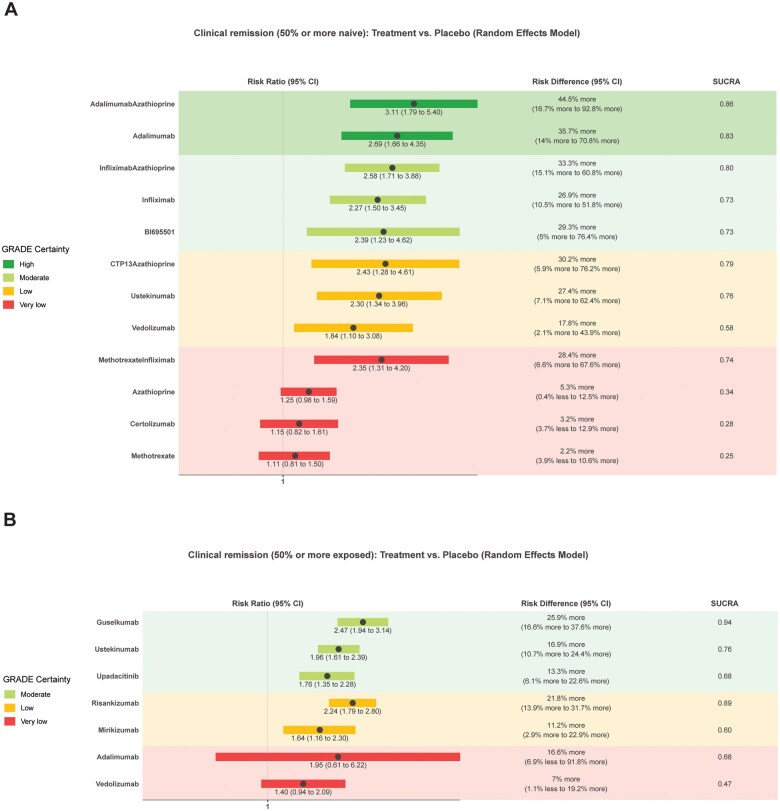
GORDON plots for the subgroup analysis for clinical remission in studies with 50% or more participants naïve to advanced treatments (A), or 50% or more participants exposed to advanced treatments (B). Network results with placebo as comparison, for clinically relevant treatments.

The preplanned subgroup analysis results for patients less or more than 50% naïve or exposed to advanced treatments revealed similar results (Figure 3).

Inspection of the preplanned sensitivity analysis for concomitant azathioprine/6-mercaptopurine use did not reveal considerable differences to the main network results, neither did the unplanned sensitivity analyses for studies only on advanced/biologic treatments and studies published on or after 2003 (Figure S3). Subgroup analyses per dosages and time point of measurement were not possible due to lack of data.

### Induction of clinical response

**Figure 4.**
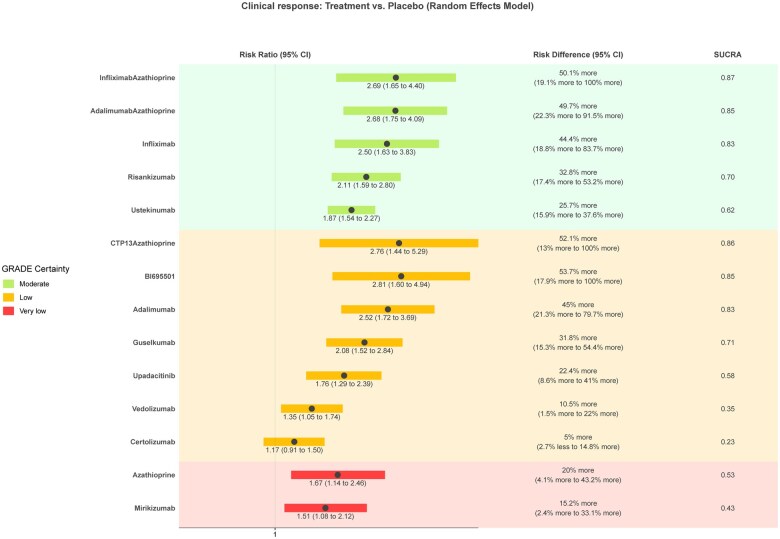
GORDON plot of clinical response network results with placebo as comparison, for clinically relevant treatments.

Fifty-five studies (*n* = 16 828) assessing 24 interventions were included in the clinical response network meta-analysis (Table S2, Figure 4B). Placebo had a mean rate of 30% (range 0%-56%) at inducing clinical response. Network heterogeneity was 52% (I^2^). Figure 3 and Table S5 provide a graphical presentation and summary of the results for this outcome. GORDON plots containing relative effects and risk differences compared to placebo for all treatments, SUCRA probability rankings, and GRADE certainty for all treatments can be found in Supplement 3.

None of the interventions were rated *high* for GRADE certainty. Five treatments were rated at moderate GRADE certainty and are probably more effective in inducing clinical response compared to placebo. In order of SUCRA ranking, these are the combination of infliximab with azathioprine/6-mercaptopurine (RR, 2.69 [95% CI, 1.65-4.4] NNT = 2 [95% CI, 1 to 5] large effect magnitude), combination of adalimumab with azathioprine/6-mercaptopurine (RR, 2.68 [95% CI, 1.75-4.09] NNT = 2 [95% CI, 1 to 5] large magnitude), infliximab (RR, 2.5 [95% CI, 1.63-3.83] NNT = 3 [95% CI, 2-6] large magnitude), risankizumab (RR, 2.11 [95% CI, 1.59-2.8] NNT = 3 [95% CI, 2-6] moderate magnitude) and ustekinumab (RR, 1.87 [95% CI, 1.54-2.27] NNT = 4 [95% CI, 3-6] moderate magnitude).

Seven treatments were rated at *low* GRADE certainty and are maybe more effective in inducing clinical response compared to placebo. In order of SUCRA ranking, these are the combination CTP13 with azathioprine/6-mercaptopurine (RR, 2.76 [95% CI, 1.44-5.29] large magnitude), BI695501 (RR, 2.81 [95% CI, 1.6-4.94] large magnitude), adalimumab (RR, 2.52 [95% CI, 1.72-3.69] large magnitude), guselkumab (RR, 2.08 [95% CI, 1.52-2.84] moderate magnitude), upadacitinib (RR, 1.76 [95% CI, 1.29-2.39] small magnitude), vedolizumab (RR, 1.35 [95% CI, 1.05-1.74] trivial magnitude), and filgotinib (RR, 1.32 [95% CI, 1.02-1.73] trivial magnitude). Three treatments were rated at low GRADE certainty and are possibly similar to placebo at inducing clinical response: tofacitinib (RR, 1.3; 95% CI, 0.9-1.87), certolizumab (RR, 1.17; 95% CI, 0.91-1.5), and apilimod mesylate (RR, 0.59; 95% CI, 0.32-1.09).

The results for 8 of the treatments had very low GRADE certainty, and no conclusions can be drawn about them (Table S5).

### Induction of endoscopic remission

**Figure 5.**
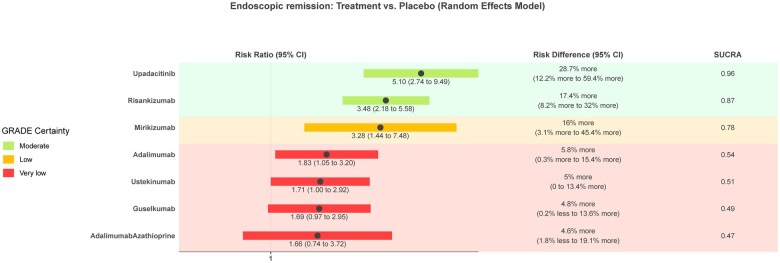
GORDON plot of endoscopic remission network results with placebo as comparison, for clinically relevant treatments.

Twenty studies (*n* = 7543) assessing 11 interventions were included in the endoscopic remission network meta-analysis (Table S2, Figure 5C). Placebo had a mean rate of 12% (range 0%-28%) at inducing endoscopic remission. Network heterogeneity was 33.4% (I^2^). Figure 4 and Table S5 provide a graphical presentation and summary of the results for this outcome. GORDON plots containing relative effects and risk differences compared to placebo for all treatments, SUCRA probability rankings, and GRADE certainty for all treatments can be found in Supplement 3.

**Figure 6.**
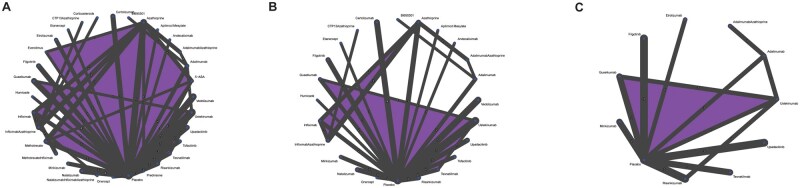
Network plots for clinical remission (A), clinical response (B), endoscopic remission (C).

None of the interventions were rated high for GRADE certainty. Two treatments were rated at moderate GRADE certainty and are probably more effective in inducing endoscopic remission compared to placebo. In order of SUCRA ranking, these are upadacitinb (RR, 5.1 [95% CI, 2.74-9.49] NNT = 3 [95% CI, 2 to 8] large effect magnitude) and risankizumab (RR, 3.48 [95% CI, 2.18-5.58] NNT = 6 [95% CI, 3-12] moderate magnitude).

One treatment was rated at low GRADE certainty and is perhaps more effective in inducing endoscopic remission compared to placebo: mirikizumab (RR, 3.28; 95% CI, 1.44-7.48, small magnitude). Two treatments were rated at low GRADE certainty and are perhaps similar to placebo at inducing endoscopic remission: etrolizumab (RR, 1.56; 95% CI, 0.63-3.86) and tesnatilimab (RR, 0.83; 95% CI, 0.36-1.92).

The results for 5 of the treatments had very low GRADE certainty, and no conclusions can be drawn about them (Table S5).

Visual inspection of the subgroup and sensitivity analyses results for studies up to 26 weeks, endoscopic remission definition of SES-CD score ≤4, and studies with patients previously exposed to biologic treatment did not reveal considerable differences from the main endoscopic network results (Figure S3). All 3 analyses are shown in the network plots in Figure 6.

### WAEs, SAEs, and TAEs

Seventy-five studies (*n* = 20 724) assessing 32 interventions were included in the WAEs network meta-analysis, with 56 studies (*n* = 19 720) assessing 28 interventions for SAEs, and 57 studies (*n* = 18 183) assessing 29 interventions for TAEs (Table S2). Network heterogeneity was 38.7%, 0% and 59.8% respectively (I^2^). Table S5 provide summaries of the results for these outcomes. GORDON plots containing relative effects and risk differences compared to placebo for all treatments, SUCRA probability rankings, and GRADE certainty for all treatments can be found in Supplement 3.

For WAEs, risankizumab had low certainty for fewer withdrawals due to adverse events compared to placebo, while mirikizumab, tofacitinib, ustekinumab, vedolizumab, upadacitinib, natalizumab, apilimod mesylate, certolizumab, filgotinib, and tesnatilimab had low certainty for no difference with placebo. All other treatments were of very low certainty, and no conclusions can be drawn about them.

For SAEs, apilimod mesylate, ustekinumab, guselkumba, natalizumab, etrolizumab, adalimumab, vedolizumab, and upadacitinib had moderate certainty for no difference with placebo. Risankizumab had low certainty for trivially fewer events, while etanercept, mirikizumab, tofacitinib, and certolizumab had low certainty for no difference. All other treatments were of very low certainty, and no conclusions can be drawn about them.

For TAEs, there was moderate certainty for no difference for ustekinumab, and natalizumab. There was low certainty for no difference forrisankizumab, apilimod mesylate, mirikizumab, tofacitinib, adalimumab, vedolizumab, certolizumab, and etrolizumab. Tesnatilimab had low certainty for large amount of more events compared to placebo. All other treatments were of very low certainty, and no conclusions can be drawn about them.

## Discussion

In the last 2 decades, several advanced therapies have been investigated for the treatment of CD, and many of them have been approved for clinical use. With the access to multiple effective therapeutic agents, positioning them in the management algorithm is challenging. Direct head-to-head clinical trials are crucial for comparative efficacies of different advanced therapies; while these are still very limited in CD, a well-executed NMA can partially address this problem by indirect comparison of these therapies.^4–6^^,^^23^^,^^24^ With 79 included RCTs and 20 724 participants, we have conducted a highly inclusive NMA for induction in CD, while maintaining the assumption of transitivity and navigating methodological challenges in the best way possible. To our knowledge, this NMA provides the most expansive comparison of therapies on this topic and transparently assesses the certainty of the evidence with the GRADE methodology.

We found that that combination of anti-TNFs (adalimumab, infliximab) and immunomodulators is the most effective treatment strategy (NNT= 3 and 4) with the largest effect size for induction of clinical remission. This is a novel finding that has not been previously described for adalimumab and is not directly in line with the direct evidence provided in the DIAMOND study.^25^ Previous evidence synthesis did not thoroughly investigate the effect of combination therapy with immunomodulators. We have made an a priori methodological assumption that cohorts with greater than 50% immunomodulator combination use with an advanced therapy were described as combination therapies and not monotherapies. However, when sensitivity analysis tested this assumption, the findings did not change.

There was moderate certainty of evidence for the aforementionedicombination therapies, adalimumab monotherapy, guselkumab, and ustekinumab. Newer advanced therapies and oral small molecules were also effective for induction of endoscopic remission, but certainty of evidence was lower. On endoscopic remission, IL-23p19 antagonists (risankizumab) and JAK1 inhibitor (upadacitinib) are probably associated with better endoscopic remission.

Anti-TNF with or without immunomodulators remain a common first-line therapy in guidelines and algorithms, although approximately one-fifth of patients experience primary nonresponse to anti-TNF agents, and another one-fifth lose response every year.^26^ Combination of thiopurines and anti-TNFs has been shown to improve clinical outcomes by preventing antidrug antibody formation. The pivotal SONIC trial demonstrated superior efficacy of combination of thiopurines and infliximab.^27^ Although combination of adalimumab and thiopurines did not show clinical benefit over monotherapy in DIAMOND trial, it showed significantly higher endoscopic improvement.^25^ In the large UK wide prospective PANTS study, concomitant use of thiopurines was associated with reduced formation of antidrug antibodies, higher drug levels at the induction therapy, which in turn was associated with higher clinical response rates.^26^ Similar advantage of combination therapy was not observed with other classes of advanced therapies.^28^ Though treatment strategies were not investigated in this study, the high efficacy of combination therapy of thiopurines and anti-TNF therapy has been recently shown in the PROFILE trial, where sustained steroid- and surgery free-remission was observed in 79% of patients in the accelerated stepup group.^29^ The placebo rates we have found are similar to those in other recently published estimates.^30^^,^^31^ As such, our findings do support this approach as an efficacious one, even in the context of the broader ranges of advanced therapeutics. CD management is very challenging, and the fact that some of the included studies include patients who failed advanced therapies or immunomodulators while others did not further complicates interpretation of our results. Active decisions need to be made by clinicians for appropriate therapy strategies.

Though newer advanced therapies and oral small molecules are promising, the certainty of evidence was rated low for most therapies because of imprecision and incoherence between direct and indirect evidence. This does not in any way indicate their lower efficacy or safety but means that from a practical and scholarly perspective we must acknowledge the doubt that exists due to the clear imprecision in magnitude estimates and that further studies could address and enhance coherence and certainty.

Our subgroup analysis for the role of prior exposure to advanced therapy on clinical remission did not reveal major differences to our main network analyses. In the naïve population, we are seeing efficacy of anti-TNF therapies and combination of anti-TNFs with immunomodulators, which reflects current practice and the key recommendations from the most recent British Society of Gastroenterology’s (BSG) IBD guidelines.^15^ In the exposed population its predominantly IL-23 and upadacitinib who have the best efficacy restuls, which again reflects current practice. However, we must highlight some key issues with this analysis that may explain the differences of this finding to other studies on biologic/advanced therapy exposure.^32–34^ The latter studies are mostly based on retrospective data for patients exposed or not exposed to any advanced treatment, or pairwise subgroup data for participants exposed or not exposed to a given advanced treatment. Since such subgroup data in our included studies are sparse, our analysis is based on the baseline characteristics of the included populations, and for this reason we had to choose a naivety threshold of included participants. We decided 50% would be the most fair threshold, albeit arbitrary due to lack of any other information we could base it on. Due to lack of data, not all treatments are present in both networks, and visual inspection for differences can only be performed for the treatments present in both networks. Additionally, the definition of exposure in the included studies varies, as each study defines exposure in relation to the specific treatment it studies, and not to all advanced treatments as a class. It should be acknowledged that the prevalence of being biologic-exposed or having a biologic-failure was higher in participants recruited in more recent trials; this is a limitation for any analysis on naivety/exposure. This matter will become even more complicated with the passage of time as most prospective participants will likely be exposed to multiple advanced treatments. We attempted a similar subgroup analysis for endoscopic remission; however, only 3 of network studies had less than 50% of their participants previously exposed, which meant we could not form networks. Therefore, we are only presenting a sensitivity analysis where the 3 studies have been removed, which is very similar to the main endoscopic network. We are emphasizing this as a major issue for the field: for the factors described previously may not be able to be addressed without a different approach to research in the future, unless there is access to individual patient data.

There are some weaknesses in the current literature we would like to highlight. The absence of results around subgroups such as age, sex, disease activity, concomitant medications, or any other characteristics in the included studies meant we could not conduct network meta-analyses on those. A solution would be that these subgroup results are published or that individual patient data become available. We decided to base our subgroup and sensitivity analyses on the reported baseline characteristics. Very few studies compared immunomodulators or advanced therapies to antibiotics, or corticosteroids as active groups. These therapies are very commonly used but very poorly reported as baseline characteristics. Therefore, we could not perform any analyses on them. The presence of these concomitant medications, however, might explain the placebo rates we observed which we do not normally see in clinical practice. We have taken the best methodological decisions we could in a relatively flawed field and produced the most accurate results possible at this point in time with the data that are available.

Endoscopic healing is associated with superior long-term outcomes including sustained clinical remission, low risk of surgery and hospitalizations, and disease complications.^35–37^ Therefore, endoscopic healing has been recommended by expert consensus guidelines as an important long-term target in the management of CD.^7^ Usually, clinical and endoscopic results for the treatment of CD do not align. In our analysis, newer advanced therapies such as upadacitinib, IL-23p19 (mirikizumab, and risankizumab) antagonists along with anti-TNF (adalimumab) showed superior efficacy in inducing endoscopic remission compared to placebo. There was moderate certainty of evidence for risankizumab and upadacitinib but very low certainty of evidence for adalimumab. In the recent SEQUENCE trial risankizumab showed significantly greater endoscopic remission compared to ustekinumab in patients with CD who had failed anti-TNF therapy.^38^ The timeline and the SES-CD definition used to define endoscopic remission did not affect the outcome. However, these results should be interpreted with caution. There is a lack of high-quality endoscopic assessment data from earlier anti-TNF clinical trials before endoscopic outcomes were mandated by regulatory authorities. Moreover, there has been evolution in the definition of endoscopic outcomes with variations in scoring indices and cut offs used. However, a significant proportion of patients recruited in recent clinical trials would have been exposed to multiple advanced therapies at the time of recruitment and required to have documented predetermined endoscopic disease activity at baseline. The majority of the included studies did not report endoscopic activity as this was not mandated until recently. For this reason, many of the most commonly used therapies are not present in our endoscopic remission results or have low or very low certainty on whether they are efficacious for this outcome or not.

Our analysis has several advantages over previously published NMAs. Our study included all up-to-date studies. Moreover, a significant proportion of patients in clinical trials received concomitant immunomodulators, which was not taken into consideration in previous NMAs. We set a threshold where studies with more than 50% of participants receiving concomitant purine analogues were considered combination therapy studies. We gave greater importance to certainty of evidence by GRADE analysis contrary to SUCRA ranking, which was the case with previous NMAs.^39^^,^^40^ SUCRA is a purely statistical method, and relying solely on it for treatment ranking without accounting for certainty of evidence can result in misinterpretation of NMA results.

We acknowledge limitations of our NMA. First, intervention doses were combined. Some included studies, especially those evaluating immunomodulators were conducted in prebiologic era when the disease profile, disease severity and baseline characteristics might have been different to the patients recruited in recent clinical trials. However, there were few such studies and definition of clinical outcomes were predominantly based on CDAI. We performed a sensitivity analysis removing older studies which did not reveal major differences to the main analysis. We cannot comment on the ideal duration of combination therapies, beyond their duration as interventions in the included studies, neither can we comment on ideal dosing regimens. Another limitation is that the networks were sparse for all outcomes as the head-to-head comparisons of active treatments are very limited. It is also disappointing that despite the size, cost, and high impact of many of the trials, unclear reporting of core quality appraisal details is still common, and this limits the certainty of evidence in any syntheses. We contacted authors when information was missing, and this has mitigated unclarity to an extent.^41^ Another limitation that must be considered is innate in the NMA approach: NMA is not the same as direct comparison studies, and although it can give strong signals through indirect and network consideration, we would not propose pairwise meta-analysis to be ignored. This is particularly pertinent in borderline cases. For example, vedolizumab had a clinical remission magnitude just at the border of trivial to small, with low GRADE certainty. However, in direct pairwise meta-analysis, the magnitude of effect was trivial and as such in the recent BSG guidelines overall magnitude was noted as trivial.^15^^,^^34^ Additional, as seen through this example, is the key limitation that lower certainties of evidence mean our confidence is lower and future studies may change findings. Whilst clinicians must act on the best evidence they have at hand, considering this is key, and this is not a limitation in the methods per se but a caution for use of the results.

We have clear proposal for future research. Reporting of core quality criteria in trial manuscripts is vital and should not require contacting RCT authors.^42^ Reporting of key patient characteristics is currently variable. Key factors such as the concomitant use of therapies such as immunomodulators, or prior exposure; randomizing in a fashion that accounts for these factors will account for this and enhance future syntheses. Endoscopic reporting is also key. The ability to use NMA in turn does perhaps support the use of more direct head to head trials, and we would join calls to consider the role of placebo controlled trials in the field.^4^^,^^5^ A method of addressing this in an innovative fashion is to consider the use of a multi-arm multi-stage platform study. The authors, together with expert colleagues, have recently laid out an approach to this that could simultaneously add substantial head to head and direct data increasing the certainty of future analyses. ^43^^,^^44^

To conclude, on network meta-analysis, combination of anti-TNFs and immunomodulators followed by anti-TNF monotherapy had a large effect size with moderate certainty for the induction of clinical remission. There was moderate certainty of evidence supporting newer advanced therapies including upadacitinib and risankizumab for induction of endoscopic remission. There was no increased risk of short-term serious adverse events with any of the advanced therapies. Novel therapies appear to have examples of similarly important effect sizes but are currently limited due to the imprecision of the limited evidence base at present and future research should target these therapies.

## Supplementary Material

izaf191_Supplementary_Data

## Data Sharing

Most data are shared in the online supplementary material. However, further data are available upon reasonable request.
