# Supplementary material for: Immunomodulators and Advanced Therapies for Induction of Remission in Crohn’s Disease: A Systematic Review and Network Meta-Analysis
Source: Inflamm Bowel Dis. 2025 Sep 19;32(1):53–66. doi: 10.1093/ibd/izaf191 (PMC12759050; doi:10.1093/ibd/izaf191)
Supplement: izaf191_Supplementary_Data [file izaf191_supplementary_data.zip › Supplement 1.pdf]

Supplementary Content

eTable1. Characteristics of included studies (page 2)

eTable 2. Included studies' efficacy outcome definitions, baseline disease activity, and time of primary outcome measurement (page 52)

eTable 3. Outcomes reported in the included studies (page 94)

eTable 4. Excluded studies and reasons for exclusion (page 104)

eTable 5. Predefined Magnitude Effect Thresholds (page 106)

eTable 6. Summary of Findings Tables and GRADE decisions (page 107)

eFigures1. Network plots (page 144)

eFigures 2. Network forest plots, SUCRA probabilities, and direct/indirect/network estimates forest plots (page 150)

eFigures 3. Subgroup and sensitivity analyses (page 167)

eFigures 4. Comparison adjusted funnel plots for the assessment of small study effects (page 75)

eAppendix 1. Search strategy (page 178)

eAppendix 2. References of included studies (page 181)

eTable1. Characteristics of included studies (79 RCTs)

Study ID (Author, Year)	Intervention group, IG (dosage, numbers randomised)	Comparator group, CG (numbers randomised)	Sex per group (M/F)	Age per group at baseline, mean (SD)	Age at diagnosis, mean (SD)	Disease location per group	Disease Duration per group, mean (SD	Disease Phenotype	Disease activity (moderate/severe or mild)	Previous experience with advanced therapy	Concomitant steroid use	Concomitant azathioprine/ 6-mercaptpurine (>20% or ≤20%)	Funding
**Allez 2023 (TRIDENT Part 1)**	Tesnatilimab (400 mg subcutaneous at Week 0, 200 mg every 2 weeks) (n=73)	Placebo (4 subcutaneous every 2 weeks) (n=72)	IG: 40/33 CG: 41/31	IG: 38.0 (13.3) CG: 38.9 (13.3)	NR	NR	NR	NR	Moderate to severe	Not naive	Bio-NF patients were either currently receiving corticosteroids and/ or immunomodulators	Unclear	Janssen
**Allez 2023 (TRIDENT Part 2)**	Tesnatilimab (400 or 150 or 50 mg subcutaneous at week 0, then half dosage at weeks 2, 4, 8, 12, 16, and 20) (n=148)	Placebo (subcutaneous every 2 weeks from Week 0–4, Week 8, every 2 weeks from Week 12–16, and Week 20) (n= 48)	IG1: 85/63 IG2: 24/23 CG: 34/16	IG1: 36.9 (13.0) IG2: 42 (12.6) CG: 40.6 (13.7)	NR	NR	NR	NR	Moderate to severe	Not naive	Bio-NF patients were either currently receiving corticosteroids and/ or immunomodulators	Unclear	Janssen

	Ustekinumab (6 mg/kg intravenous injection at week 0, 90mg subcutaneous at week 8 and 16) (n=47)												
**Ardizzone 2003**	Azathioprine (2 mg/kg per day orally) (n=27)	Methotrexate (25 mg/week intravenous injection for 3 months, then oral 25 mg/week) (n=27)	IG: 15/12 CG: 13/14	IG: 31 (10) CG: 37 (7)	NR	IG: Small bowel 1, Colon 6, Both 20 CG: Small bowel 5, Colon 4, Both 18	IG: 57.26 (48.58) months CG: 76.60 (58.48) months	NR	NR	Naive	40 mg prednisolone daily for 2 weeks, then 30 and 20 mg daily, for the following 2 and 4 weeks. After 8 weeks, if the patient's condition had remained stable or improved, prednisolone was tapered by 5 mg each week. In those patients whose condition worsened, dosage was increased to a maximum	Part of intervention	Unclear

											daily dose of 40 mg.		
**Arora 1999**	Methotrexate (15 mg/week oral, dose adjusted up to 22.5 mg/week) (n=15)	Placebo (n=18)	Unclear	Unclear	Unclear	Unclear	Unclear	Unclear	Unclear	Naive	Prednisone tapered according to clinical status (physician's decision) either after 12 weeks (MTX 6 pa- tients, placebo 12 patients),or at any time during the study (MTX 9 patients, placebo 6 patients)	Part of intervention	Unclear
**Candy 1995**	Azathioprine (2.5 mg/kg) (n=33)	Placebo (n=30)	IG: 7/26<br>CG: 11/19	IG: 33-9 (15-60)<br>CG: 31 8 (21-62) median and range	NR	IG: ileal 8 ileocolitis 20, colonic 5<br>CG: ileal 6, ileocolitis 19, colonic 5	IG: 3.7 (0.6-19.3) year<br>CG: 4.7 (0.2-19.4) year Median (range)	NR	Unclear	Naive	At day 1, all randomised patients were given prednisolone at a starting dose of 1 mg/kg/day. They were requested to decrease this dose by 5 mg per week to zero at the end of week 12.	Part of intervention	Unclear

**Chen 2020**	Adalimumab (160 mg at week 0, 80 mg at week 2, and 40 mg at weeks 4 and 6) + Azathioprine (unclear) (n=102)	Azathioprine (unclear) (n=103)	IG: 67/35 CG: 73/30	IG: 33.2 (10.2) CG: 32.6 (9.5)	NR	IG: Colonic 19 Ileal 22 Ileal-colonic 59 upper-disease 9 CG: Colonic 23 Ileal 8 Ileal-colonic 58 upper-disease 10	IG: 3.1 (3.2) year CG: 2.3 (2.7) year	NR	Moderate to severe	Naive	steroid use IG: 31 CG: 32	Part of intervention	AbbVie
**Chen 2025 (NCT03234907)**	Vedolizumab (300 mg intravenous once at Weeks 0, 2, and 6) (n=144)	Placebo (intravenous once at Weeks 0, 2, and 6) (n=71)	IG: 96/48 CG: 54/16	IG: 31.1(10.10) CG: 31.1(8.12)	NR	IG Ileum only: 31 Colon only: 26 Both: 87<br><br>CG Ileum only: 8 Colon only: 14 Both: 48	NR	NR	Moderate to severe	Not Naive	Oral corticosteroids IG: 14 CG: 5	Unclear. Patients were allowed concomitant stable doses of immunomodulators and more than 88% had were refractory or intolerant to them in both groups	Takeda
**Colombel 2007 (CHARM) -Induction (non-responders)**	Adalimumab every other week (40 mg every other week) (n= 88)<br><br>Adalimumab Weekly	Placebo (n=91)	All participants: 108/171	All participants: 37.9 (11.8)	NR	Colonic 206 Ileal 214 Gastroduodenal 10 Other 44	at least 4 months	NR	Moderate to severe	Not naïve >50%	Any steroid: 129 (46.2%) Prednisone: 86 (30.8%) Budesonide: 39 (14.0)	>20%	Abbott

	(40 mg weekly) (n=100)												
**Colombel 2010 (SONIC)**	Infliximab (5 mg/kg intravenous infusion at weeks 0, 2, and 6, then every 8 weeks) (n=169)<br><br>Infliximab (5 mg/kg intravenous infusion at weeks 0, 2, and 6, then every 8 weeks) + Azathioprine (2.5 mg/kg daily oral capsules) (n=169)	Azathioprine (2.5 mg/kg daily oral capsules) (n=170)	IG1: 84/85<br>IG2: 88/81<br>CG: 90/80	IG1: 35.0<br>IG2: 34.0<br>CG: 35.0<br>Median	NR	IG1: Ileum 54 Colon 45 Ileum and colon 64 Proximal gastrointestinal tract 12<br>IG2: Ileum 54 Colon 40 Ileum and colon 73 Proximal gastrointestinal tract 16<br>CG: Ileum 68 Colon 33 Ileum and colon 69 Proximal gastrointestinal tract 7	IG1: 2.2 year<br>IG2: 2.2 year<br>Cg: 2.4 year median	Patients with heterozygous thiopurine methyltransferase phenotype were excluded	Moderate to severe	Naive	Systemic steroids use:<br>IG1: 52<br>IG2: 47<br>CG: 40	Part of intervention	Centocor
**D'Haens 1999**	Infliximab 5mg (5 mg/kg intravenous, single infusion) (n=7)	Placebo (n=8)	IG1: 3/4<br>IG2: 3/4<br>IG3: 3/5<br>CG: 3/5	IG1: 30.1 (5.0)<br>IG2: 30.7 (8.7)<br>IG3: 33.1 (7.8)<br>CG: 34.4 (9.8)	NR	IG1: Ileum 6, Colon 12, Ileocolonic 12<br>IG2: Ileum 6, Colon 12,	NR	NR	Moderate to severe	Naive	steroids use:<br>IG1: 4/7<br>IG2: 3/7<br>IG3: 4/8<br>CG: 5/8	>20%	Centocor

	Infliximab 10mg (10 mg/kg intravenous, single infusion) (n=7) Infliximab 20mg (20 mg/kg intravenous, single infusion) (n=8)					Ileocolonic 12 IG3: Ileum 6, Colon 12, Ileocolonic 12 CG: Ileum 6, Colon 12, Ileocolonic 12							
**D'Haens 2008**	Infliximab (5 mg/kg at weeks 0, 2, and 6) + Azathioprine (2–2.5 mg/kg daily or methotrexate 25 mg each week for 12 weeks with the dose reduced to 15 mg per week thereafter if intolerant to	Corticosteroids (Methylprednisolone 32 mg every day for 3 weeks, then tapering by 4 mg per week; budesonide 9 mg every day for 8 weeks with tapering to discontinuation by 3 mg per week) (n=66)	IG: 22/43 CG: 27/37	IG: 30.0 (11.8) CG: 28.7 (10.9)	NR	IG: Small bowel 14, Ileocolitis 32, Colitis 20 CG: Small bowel 15, Ileocolitis 28, Colitis 21	NR	NR	NR	Naive	Patients had not previously received steroids	Part of intervention	Centocor

	azathioprine) (n=67)												
**D'Haens 2022 (ADVANCE)**	Risankizumab (600 or 1200 mg intravenous at Weeks 0, 4, and 8) (n=675)	Placebo (intravenous at Weeks 0, 4, and 8) (n=186)	IG1: 189/147 IG2: 183/156 CG: 88/87	IG1: 38.3 (13.3) IG2: 37.0 (13.2) CG: 37.1 (13.4)	NR	IG1: Ileal 52, Colonic 115, Ileal-colonic 169 IG2: Ileal 54, Colonic 118, Ileal-colonic 167 CG: Ileal 19, Colonic 70, Ileal-colonic 86	IG1: 9.0 (8.8) years IG2: 8.9 (8.4) years CG: 8.2 (7.1) years	NR	Moderate to severe	Not naïve >50%	steroid use: IG1: 102 IG2: 101 CG: 50	>20%	AbbVie
**D'Haens 2022 (MOTIVATE)**	Risankizumab (600 or 1200 mg intravenous at Weeks 0, 4, and 8) (n=382)	Placebo (intravenous at Weeks 0, 4, and 8) (n=187)	IG1: 92/99 IG2: 102/89 CG: 99/88	IG1: 40.2 (13.6) IG2: 39.3 (12.9) CG: 39.3 (13.5)	NR	IG1: Ileal 33, Colonic 75, Ileal-colonic 83 IG2: Ileal 21, Colonic 74, Ileal-colonic 96 CG: Ileal 26, Colonic 73, Ileal-colonic 88	IG1: 10·9 (7·7) years IG2: 11·8 (9·1) years CG: 12·5 (9·7) years	NR	Moderate to severe	Not naïve >50%	steroid use: IG1: 65 IG2: 62 CG: 68	>20%	AbbVie
**Duan 2013**	Infliximab (5 mg/kg at week 0, 2, 6, and then every 8 weeks) (n=8) Infliximab	Azathioprine (2.5 mg/kg qd) (n=8)	IG1: 7/1 IG2: 8/0 CG: 6/2	IG1: 34.4 (12.6) IG2: 35.4 (18.8) CG: 36.2 (17.6)	NR	NR	NR	NR	Moderate to severe	Unclear	NR	Part of intervention	Unclear

	(5 mg/kg at week 0, 2, 6, and then q8w) + Azathioprine (2.5 mg/kg qd) (n=8)												
**Ewe 1993**	Azathioprine (2.5 mg/kg every day) (n=21)	Placebo (n=21)	NR	IG: 27.3 (18–43) CG: 29.3 (18–48) Mean (range)	NR	IG: Ileocolic 14, Ileum 1, Colon 6 CG: Ileocolic 11, Ileum 5, Colon 5	Symptoms IG: 7.4 years CG: 5 years Diagnosis IG: 4.6 years CG: 3.9 years	NR	Patients with severe CD were excluded	Naive	Prednisolone 60 mg every day, tapering to 10 mg every day.	Part of intervention	Unclear
**Feagan 1995**	Methotrexate (25 mg intramuscular weekly) (n=94)	Placebo (intramuscular weekly) (n=47)	IG: 51/43 CG: 26/21	IG: 34 (1) CG: 36 (2)	NR	IG: Small bowel 30, Colon 15, Both 49 CG: Small bowel 8, Colon 9, Both 30	Months since diagnosis IG: 93 (8) CG: 98 (12)	NR	NR	Naive	All patients received prednisone (20 mg once a day), which was tapered over a period of 10 weeks unless their condition worsened.	Part of intervention	Uncler
**Feagan 2008c**	Vedolizumab (2.0 or 0.5 mg/kg	Placebo (intravenous on Days 1 and 29)	IG1: 31/34 M IG2: 25/37 M	IG1: 38.5 (13.07) IG2: 36.0 (12.67)	NR	NR	Months since diagnosis	NR	NR	Naive	Patients requiring systemic corticosteroids	<20%	Millenium

	intravenous on Days 1 and 29) (n= 127)	(n=58)	CG: 30/28	CG: 34.5 (11.26)			IG1: 96 (94.8) IG2: 105 (99.2) CG: 109 (99.3)				were excluded.		
**Feagan 2014**	Methotrexate (10 mg/week subcutaneous, then 20 mg at week3, 25 mg/week through week 50) + Infliximab (5 mg/kg intravenous at weeks 1, 3, 7, 14, 22, 30, 38, and 46) (n=63)	Infliximab (5 mg/kg intravenous at weeks 1, 3, 7, 14, 22, 30, 38, and 46) (n=63)	IG: 34/29 CG: 37/26	IG: 40.4 (13.3) CG: 38.5 (12.9)	NR	IG: Small bowel 11, Ileocolitis 38, Colitis 14, CG: Small bowel 13, Ileocolitis 35, Colitis 14, Unknown 1	Months since diagnosis IG: 130.9 (119.7) CG: 115.4 (103.2)	NR	NR	Naive	Prednisone induction therapy (15–40 mg/day) within the preceding 6 weeks. Prednisone was tapered, beginning at week 1, and discontinued no later than week 14.	Part of intervention	Merck/Schering Plough and Prometheus Laboratorie
**Feagan 2015a (UNITI-1)**	Ustekinumab (130 mg or 6 mg/kg single intravenous infusion at week 0) (n=494)	Placebo (single intravenous infusion) (n=247)	IG1: 98/147 IG2: 101/148 CG: 118/129	IG1: 37.4 (11.8) IG2: 37.3 (12.5) CG: 37.3 (11.8)	NR	IG1: Ileum 38, Colon 36, Ileum and colon 171, Proximal GI tract 57, Perianal GI tract 107 IG2: Ileum 37, Colon	IG1: 11.8 (8.3) years IG2: 12.7 (9.2) years CG: 12.1 (8.4) years	NR	Moderate to severe	Not	Stable doses of or oral glucocorticoids (≤40 mg of prednisone per day or ≤9 mg of budesonide per day) were permitted.	>20%	Janssen

						40, Ileum and colon 171, Proximal GI tract 54, Perianal GI tract 107 CG: Ileum 28, Colon 48, Ileum and colon 166, Proximal GI tract 45, Perianal GI tract 107					Glucocorticoid at baseline: IG1: 121, IG2: 108, CG:111		
**Feagan 2015b (UNITI-2)**	Ustekinumab (130 mg or 6 mg/kg single intravenous infusion at week 0) (n=418)	Placebo (n=210)	IG1: 104/105 IG2: 90/119 CG: 99/111	IG1: 39.1 (13.8) IG2: 38.4 (13.1) CG: 40.2 (13.1)	NR	IG1: Ileum 53, Colon 44, Ileum and colon 109, Proximal GI tract 34, Perianal GI tract 60 IG2: Ileum 49, Colon 43, Ileum and colon 117, Proximal GI tract 29, Perianal GI tract 61 CG: Ileum 44, Colon 37, Ileum	IG1: 8.7 (8.5) years IG2: 8.7 (8.4) years CG: 10.4 (9.8) years	NR	Moderate to severe	Not	Stable doses of or oral glucocorticoids (≤40 mg of prednisone per day or ≤9 mg of budesonide per day) were permitted. Glucocorticoid at baseline: IG1: 80, IG2: 92, CG:75	>20%	Janssen

						and colon 129, Proximal GI tract 32, Perianal GI tract 57							
**Feagan 2017**	Risankizumab (200 or 600 mg intravenous at Weeks 0, 4, 8) (n=82)	Placebo (intravenous at Weeks 0, 4, 8) (n=39)	IG: 31/51 CG: 16/23	IG: 39 (13) CG: 36 (14)	NR	IG: Colonic 29% Ileal 20% Ileal-colonic 50% CG: Colonic 29% Ileal 20% Ileal-colonic 50%	IG: 14 (9) years CG: 12 (9) years	NR	Moderate to severe	Not	Corticosteroids only IG: 16 CG: 9 Corticosteroids and IM IG: 6 CG: 5	<20%	AbbVie
**Ferrante 2024 (VIVID-1)**	Mirikizumab: a single dose intravenously at weeks 0, 4, and 8	Ustekinumab: a single intravenous dose at week 0 followed by placebo intravenous administrations at weeks 4 and 8<br><br>Placebo: a single dose intravenously at	Miri: 332/247<br><br>Uste: 137/150<br><br>Placebo: 118/81	Miri: 36(12.7)<br><br>Uste: 36.6(12.7)<br><br>Placebo: 36.3(12.7)	NR	Miri: Ileum only: 65 Colon only: 225 Both: 289<br><br>Uste: Ileum only: 29 Colon only: 120 Both: 138<br><br>Placebo: Ileum only: 19 Colon only: 77	Years<br><br>Miri: 7.4(8.2)<br><br>Uste: 7.2(7.7)<br><br>Placebo: 7.8(7.4)	NR	Moderate to severe	Not naive	Miri: 177<br><br>Uste: 90<br><br>Placebo: 58	>20%	Eli Lily

		weeks 0, 4, and 8				Both: 103							
**Ghosh 2003**	Natalizumab (1 Infusion of 3 mg/kg or 2 Infusion of 3 mg/kg or 2 Infusion of 6 mg/kg intravenous infusion four weeks apart) (n=185)	Placebo (n=63)	IG1:27/41 IG2: 30/36 IG3: 25/26 CG: 30/33	IG1: 36 (18-66) IG2: 36 (19-64) IG3: 35 (19-62) CG: 34 (18-68) Median (range)	NR	IG1: Ileum 9, colon 16, ileum and colon 43 IG2: Ileum 17, colon 16, ileum and colon 33 IG3: Ileum 12, colon 16, ileum and colon 23 CG: Ileum 15, colon 11, ileum and colon 37	IG1: 8.4 (0.5-27.6) years IG2: 8.1 (0.5-27.6) years IG3: 7.8 (0.6-29.0) years CG:8.9 (0.3-64.3) years Median (range)	NR	Moderate to severe	Naive	Oral corticosteroids IG1: 31 IG2: 37 IG3: 32 CG: 31	>20%	Elan
**Gordon 2001**	Natalizumab (3 mg/kg single intravenous infusion) (n=18)	Placebo (Single intravenous infusion) (n=12)	IG: 7/11 CG: 5/7	IG: 36.0 (13.2) CG: 34.4 (8.8)	NR	IG: Ileal or ileocecal 7, Colonic 5, Ileal and colonic5, Perianal 5 CG: Ileal or ileocecal 5, Colonic 3, Ileal and colonic4, Perianal 4	IG: 8.5 (9.6) years CG: 8.4 (6.0) years	NR	Mild to moderate	Naive	Prednisolone/budesonide use: IG: 10 CG: 9	>20%	Elan

**Hanauer 2002-ACCENT I (non-responders)**	Infliximab 5mg (5 mg/kg intravenous infusion at week 0, 2 and 6 and every 8 weeks thereafter until week 46) (n= 79) Infliximab 5+10mg (5 mg/kg intravenous infusion at week 0, 2 and 6 and 10 mg/kg every 8 weeks until week 46) (n= 81)	Placebo (5 mg/kg intravenous infusion infliximab at week 0, 2 and 6 and every 8 weeks thereafter until week 46) (n=78)	All participants 109/129	All participants 37 (30-46) median (IQR)	NR	All participants Ileum 63/237, Colon 35/237, Ileum and colon 139/237, Gastroduodenum 19/238	All participants 9.3 (4.6-15.3) median (range)	NR	Moderate to severe	Naive	Patients with concomitant any corticosteroids 118 >20 mg per day 32	>20%	Centocor
**Hanauer 2006 (CLASSIC I)**	Adalimumab (40 mg or 80 mg or 160 mg subcutaneous at week 0 and half	Placebo (at week 0 and 2) (n=74)	IG1: 39/35 IG2: 25/50 IG3: 36/40 CG: 37/37	IG1: 39 (13) IG2: 38 (12) IG3:39 (11) CG: 37 (13)	NR	IG1: Colonic 23, Ileal 45, Ileocolonic 4, Perianal 0, Small bowel 1, Unclassifiable1	NR	NR	Moderate to severe	Naive	IG1: Systemic corticosteroid11, Budesonide 6 IG2: Systemic corticosteroid 32, Budesonide 20	<20%	Abbott

	dose at week 2) (n=224)					IG2: Colonic 17, Ileal 47, Ileocolonic 7, Perianal 1, Small bowel 0, Unclassifiable 3 IG3: Colonic 22, Ileal 40, Ileocolonic 8, Perianal 1, Small bowel 2, Unclassifiable 3 CG: Colonic 14, Ileal 50, Ileocolonic 7, Perianal 0, Small bowel 0, Unclassifiable 3					IG3: Systemic corticosteroid 24, Budesonide 12 CG: Systemic corticosteroid 25, Budesonide 17		
**Hanauer 2021 (VOLTAIRE-CD)**	Adalimumab (160 mg subcutaneous on day 1, 80 mg subcutaneous on day 15, followed	BI695501 (160 mg subcutaneous on day 1, 80 mg subcutaneous on day 15, followed by 40 mg	IG: 41/31 CG: 37/31	IG: 30.0 (25.0–40.0) CG: 35.0 (25.5–46.0) median (IQR)	NR	IG: Ileum 42%, Colon 27%, Ileum-Colon 31% CG: Ileum 41%, Colon 29%,	NR	NR	Moderate to severe	Naïve (>90%)	NR	Unclear	Boehringer Ingelheim

	by 40 mg every 2 weeks) (n=74)	every 2 weeks) (n=72)				Ileum-Colon 30%							
**Hart 2025 (GRAVITI)**	IG1: Guselkumab subcut 400mg every 4 weeks (switched to 200mg after week 16)<br><br>IG1: Guselkumab subcut 400mg every 4 weeks (switched to 100mg after week 16)	CG: Placebo identical to IG	IG1: 70/45<br><br>IG2: 66/49<br><br>CG:67/50	IG1: 39.1(12.56)<br><br>IG2: 37.4(13.32)<br><br>CG: 36.0(12.71)	NR	IG1: Ileum only: 27<br>Colon only: 40<br>Both: 48<br><br>IG2: Ileum only: 25<br>Colon only: 41<br>Both: 49<br><br>CG: Ileum only: 22<br>Colon only: 40<br>Both: 55	NR	NR	Moderate to severe	Not naive	IG1: 38<br><br>IG2: 32<br><br>CG: 33	>20%	Johnson and Johnson
**Jairath 2025 (RELIEVE UCCD)**	IG1: Duvakitug subcutaneously at 2250 mg loading dose followed by	CG: Placebo<br><br>subcutaneously at 2250 mg loading dose	IG1: 27/19<br>IG2: 31/15<br><br>CG:22/24	IG1: 42.5(15.1)<br><br>IG2: 37.8(13.6)<br><br>CG: 38.3(15.1)	NR	NR	IG1: 11.5(10.3)<br><br>IG2: 11.3(11.2)	NR	Moderate to severe	Not naive	IG1: 15<br><br>IG2: 12<br><br>CG: 20	<20%	Teva and Sanofi

	duvakitug 450 mg<br><br>IG2: Duvakitug subcutaneously at 2250 mg loading dose followed by duvakitug 900 mg	followed by placebo					CG: 9.6(7.6)						
**Lemann 2006**	Azathioprine or 6-MP (2–3 mg/kg/day for AZA and 1–1.5 mg/kg/day for 6-MP) (n= 58)	Infliximab (5 mg/kg IV at weeks 0, 2, and 6) Azathioprine or 6-MP (2–3 mg/kg/day for AZA and 1–1.5 mg/kg/day for 6-MP) (n=57)	IG: 24/32 CG: 27/30	Unclear	NR	IG: Small bowel 7, Colon 22, Both 6 CG: Small bowel 16, Colon 12, Both 22	Unclear	NR	Moderate to severe	Naive	All patients started on ≥10 mg/day prednisone/prednisolone. CDAI >150: +15 mg. After 2 weeks, remission: taper; no remission: ↑ to 40 mg/day or 1 mg/kg/day. Relapse: restart/increase steroids, then taper. Failure at ≥40 mg/day: steroid-resistant, offered infliximab.	Part of intervention	GETAID group

**Loftus 2023 U-EXCEL**	Upadacitinib (45 mg once daily) (n=350)	Placebo (once daily) (n=176)	IG: 189/161 CG: 94/82	IG: 39.7 (13.7) CG: 39.3 (13.6)	NR	IG: Ileal 58, Colonic 121, Ileal-colonic 171 CG: Ileal 27, Colonic 57, Ileal-colonic 92	IG: 6.7 (0.1-52.1) years CG: 5.7 (0.3-46.3) years Median (range)	NR	Moderate to severe	Not Naive	Glucocorticoids use: IG: 126 CG: 64	<20%	AbbVie
**Loftus 2023 U-EXCEED**	Upadacitinib (45 mg once daily) (n=324)	Placebo (once daily) (n=171)	IG:169/155 CG: 96/75	IG: 38.4 (13.7) CG: 37.5 (12.1)	NR	IG: Ileal 48, Colonic 112, Ileal-colonic 164 CG: Ileal 23, Colonic 68, Ileal-colonic 80	IG: 9.3 (0.5-55.2) years CG: 9.8 (0.6-46.1) years Median (range)	NR	Moderate to severe	Not naive	Glucocorticoids use: IG: 108 CG: 60	>20%	AbbVie
**Mantzaris 2004**	Infliximab (5 mg/kg intravenous at weeks 0, 2, 6) (n=23)	Infliximab (5 mg/kg intravenous at weeks 0, 2, 6) + Azathioprine (2.5 mg/kg PO) (n=27)	IG: 10/13 CG: 11/16	IG: 34 (20-60) CG: 33 (18-62)	NR	IG: small bowel 31%, ileocolitis 53%, colitis 16% CG: small bowel 32%, ileocolitis 56%, colitis 12%	IG: 2 (1.5-4.5) years CG: 2.5 (2-5) years Mean (range)	NR	NR	Naive	IG: dosage 15 (7.5-30) mg CG: 18 (5-30) mg Median daily dose (range)	Part of intervention	Unclear
**Mate-Jimenez 2000**	Azathioprine (1.5 mg/kg/day oral)	Methotrexate (15 mg/week oral)	Unclear	NR	NR	IG: Small bowel and colon 10, Colon 6	IG: 4.5 (3) years CG1: 4.3 (2) years	NR	NR	Naive	All patients received prednisone IG: dosage 30 (6)	Part of intervention	Unclear

	(n=16)	(n=15)<br><br>5-ASA (3g/day oral) (n=7)				CG1: Small bowel and colon 9, Colon 6<br>CG2: Small bowel and colon 6, Colon 1	CG2: 3.5 (2) years				CG1: 36 (8) mg<br>CG2: 32 (5)<br>Mean last 3 weeks		
**Matsumoto 2016 - DIAMOND**	Adalimumab (160 mg subcutaneous at Week 0, 80 mg at Week 2, and there-after 40 mg at every other week up to 52 weeks)<br>+<br>Azathioprine (25 mg or 50 mg/day increased to a maximum of 100 mg during the initial four weeks) (n=92)	Adalimumab (160 mg subcutaneous at Week 0, 80 mg at Week 2, and there-after 40 mg at every other week up to 52 weeks) (n=85)	IG: 67/24<br>CG: 59/26	IG: 29 (12)<br>CG: 29 (12)	NR	IG: Ileitis 19, Ileocolitis 58, Colitis 14<br>CG: Ileitis 15, Ileocolitis 56, Colitis 14	IG: 3.2 (5.2) years<br>CG: 2.8 (5.9) years	IG: Inflammatory 36, Stricturing 33, Penetrating 22<br>CG: Inflammatory 36, Stricturing 28, Penetrating 21	Moderate to severe	Naive	Steroid use at entry<br>IG: 5<br>CG: 13	Part of intervention	None

**Oren 1997**	Azathioprine (50 mg/day oral) (n=32)	Methotrexate (12.5 mg/week oral) (n=26) Placebo (n=26)	IG:17/14 CG1: 14/12 CG2: 12/14	IG: 34.03 (11.98) CG1: 38.2 (15.65) CG2: 33.43 (12.11)	NR	IG: small bowel 14, large bowel 4, both 12 CG1: small bowel 8, large bowel 9, both 7 CG2: small bowel 12, large bowel 6, both 6	IG: 8.43 (7.38) years CG1: 7.50 (6.85) years CG2: 4.77 (3.94)	NR	NR	Naive	All patients have steroid therapy (at ≥7.5 mg/day) for at least 4 months during the preceding 12 months.	Part of intervention	Unclear
**Panaccione 2024 (GALAXI -2)**	(Group 1) Guselkumab 200 mg IV q4w Followed by 200 mg SC q4w<br><br>(Group 2) Guselkumab 200 mg IV q4w Followed by 100 mg SC q8w	(Group 3) Ustekinumab 6 mg/kg IV Followed by 90 mg SC q8w<br><br>(Group 4) Placebo q4w Followed by Placebo q4w or Ustekinumab 6 mg/kg IV Then 90 mg SC q8w	Group 1: 89/59 Group 2: 72/76 Group 3: 88/62 Group 4: 42/35	Group 1: 36.2(13.03) Group 2: 37.3(12.86) Group 3: 37(12.76) Group 4: 34.1(11.81)	NR	Pooled GALAXI 2/3<br><br>Group 1: Ileum only:80 Colon only:102 Both: 104<br><br>Group 2: Ileum only: 59 Colon only: 113 Both: 114<br><br>Group 3: Ileum only: 55 Colon only: 116 Both: 120	Pooled GALAXI 2/3<br><br>Group 1: 7.1(7.2 Group 2: 7.1(6.7) Group 3:7.3(7.5) Group 4: 7.1(7.5)	NR	Moderate to severe	Not naive	Pooled GALAXI 2/3<br><br>Group 1: 106 Group 2: 109 Group 3: 109 Group 4: 51	Pooled GALAXI 2/3<br><br>>20%	Janssen

						Group 4: Ileum only: 31 Colon only: 62 Both: 55							
**Panaccione 2024 (GALAXI -3)**	(Group 1) Guselkumab 200 mg IV q4w Followed by 200 mg SC q4w<br><br>(Group 2) Guselkumab 200 mg IV q4w Followed by 100 mg SC q8w	(Group 3) Ustekinumab 6 mg/kg IV Followed by 90 mg SC q8w<br><br>(Group 4) Placebo q4w Followed by Placebo q4w or Ustekinumab 6 mg/kg IV Then 90 mg SC q8w	Group 1: 92/59 Group 2: 88/60 Group 3: 84/66 Group 4: 54/26	Group 1: 37.6(13.4) Group 2: 34.8(11.48) Group 3: 37.9(13.6) Group 4: 35.8(12.48)	NR	Pooled GALAXI 2/3<br><br>Group 1: Ileum only:80 Colon only:102 Both: 104<br><br>Group 2: Ileum only: 59 Colon only: 113 Both: 114<br><br>Group 3: Ileum only: 55 Colon only: 116 Both: 120<br><br>Group 4: Ileum only: 31 Colon only: 62	Pooled GALAXI 2/3<br><br>Group 1: 7.1(7.2 Group 2: 7.1(6.7) Group 3:7.3(7.5) Group 4: 7.1(7.5)	NR	Moderate to severe	Not naive	Pooled GALAXI 2/3<br><br>Group 1: 106 Group 2: 109 Group 3: 109 Group 4: 51	Pooled GALAXI 2/3<br><br>>20%	Jannsen

						Both: 55							
**Panes 2017 Induction**	Tofacitinib (5, 10 or 15 mg twice daily) (n=188)	Placebo (n=92)	IG1: 54/32<br>IG2: 39/47<br>IG3: 9/7<br>CG: 31/60	IG1: 40.2 (11.5)<br>IG2: 39.3 (13.7)<br>IG3: 41.3 (14.3)<br>CG: 37.2 (11.7)	NR	IG1: ileum and/or terminal ileum 14, ileum and/or terminal ileum+upper gastrointestinal tract 3, colon 4, colon+upper gastrointestinal tract 12, ileocolon 12, ileocolo+ upper gastrointestinal tract 41<br>IG2: ileum and/or terminal ileum 7, ileum and/or terminal ileum+upper gastrointestinal tract 2,	IG1: 11.2 (8.2) years<br>IG2: 11.3 (9.7) years<br>IG3: 11.1 (8.6) years<br>CG: 10.9 (8.6) years	NR	Moderate to severe	Not naïve >50%	Use of corticosteroids at study entry<br>IG1: 32<br>IG2: 28<br>IG3: 4<br>CG:35	<20%	Pfizer

						colon 5, colon+upper gastrointestinal tract 16, ileocolon 15, ileocolo+ upper gastrointestinal tract 39, missing 2<br>IG3: ileum and/or terminal ileum 1, ileum and/or terminal ileum+upper gastrointestinal tract 0, colon 2, colon+upper gastrointestinal tract 1, ileocolon 3, ileocolo+ upper gastrointestinal tract 9							

						CG: ileum and/or terminal ileum 10, ileum and/or terminal ileum+upper gastrointestinal tract 4, colon 5, colon+upper gastrointestinal tract 18, ileocolon 25, ileocolo+ upper gastrointestinal tract 29							
**Present 1999**	IG1: Infliximab 5mg/kg intravenous at weeks 0,2,6) (n=31)<br><br>IG2: Infliximab 10mg/kg at weeks	Placebo (n=31)	IG1: 15/16<br><br>IG2: 12/20<br><br>CG:17/14	Years<br>IG1**:** 41.2(12.2)<br><br>IG2: 35.0(12.3)<br><br>CG 35.4(8.6)	NR	IG1<br>Ileum: 7<br>Colon: 7<br>Ileum and Colon: 17<br><br>IG2<br>Ileum: 4<br>Colon: 10<br>Ileum and Colon: 18	Years<br>IG1<br>13.6(9.5)<br><br>IG2<br>11.5(8.2)<br><br>CG<br>12.0(7.9)	NR	Mild based on CDAI Score<br>IG1: 184.4 (98.5)<br><br>IG2: 184.9 (97.5)	Naive	Participant on corticosteroids discontinued it 4weeks before enrolment	>20%	Centocor

	0,2,6)<br>(n=32)					CG:<br>Ileum: 3<br>Colon: 9<br>Ileum and Colon: 19			CG:<br>192.9<br>(92.0)				
**Reinisch 2008**	IG1:<br><br>Everolimus 6 mg/day orally (n=60)<br><br>IG2:<br>Azathioprine 2.5 mg/day orally (n=52)	Placebo (n=29)	IG1: 22/38<br>IG2: 26/24<br>CG: 16/12	Median(range).<br>IG:<br>32.0 (18–69)<br><br>IG2:<br>38.5 (19–74)<br><br>CG:<br>39.5 (18–64)	NR	IG1<br>Ileal involvement: 45<br>Colonic involvement: 44<br><br>IG2<br>Ileal involvement: 37<br>Colonic involvement: 38<br><br>CG<br>Ileal involvement: 24<br>Colonic involvement: 28	IG1: 1.6 (0.0–16.2)<br><br>IG2: 1.2 (0.0–20.3)<br><br>CG: 0.6 (0.00–14.9)	NR	Moderate to Severe	Naive	All patients initially received oral prednisone according to local practice either at a dose of 1 mg/kg/day or at a fixed dose of ≥40 mg/day until response	Part of intervention	Novartis
**Rutgeerts 2006**	IG1:<br>Onercept 10 mg (n=44)		IG1: 15/29<br>IG2 :21/21<br>IG3: 17/23<br>IG4: 18/25	IG1<br>34.0 (11.0)<br><br>IG2<br>35.7 (11.5)	NR	IG 1<br>Ileum :31<br>Colon, left side: 19<br>Colon right side: 22	IG1<br>93.5<br>(69.3)<br><br>IG2	NR	Moderate to severe	Naive	Glucocorticoids<br>IG1: 12<br><br>IG 2:14	>20%	Centocor

	IG2:<br>Onercept<br>25mg<br>(n=42)<br><br>IG3:<br>Onercept<br>35mg<br>(n=40)<br><br>IG4:<br>Onercept<br>50mg<br>(n=43)<br><br>All given<br>subcutaneo<br>usly 3<br>times<br>weekly		CG<br>22/16	IG3<br>39.2 (13.2)<br><br>IG4<br>35.6 (10.8)<br><br>CG:<br>39.1 (11.3)		Jejunum:1<br>Duodenum<br>:1<br>Other:4<br><br>IG2<br>Ileum:25<br>Colon, left<br>side:20<br>Colon right<br>side:19<br>Jejunum:1<br>Duodenum<br>: 0<br>Other:6<br><br>IG3<br>Ileum 30:<br>Colon, left<br>side:22<br>Colon right<br>side: 19<br>Jejunum:3<br>Duodenum<br>:0<br>Other:3<br><br>IG4<br>Ileum :34<br>Colon, left<br>side: 24<br>Colon right<br>side: 14<br>Jejunum:1<br>Duodenum<br>:1<br>Other: 5	146.3(10<br>1.9)<br><br>IG3<br>125.2<br>(87.1)<br><br>IG4<br>112.4<br>(102.6)<br><br>CG: 150.<br>(94.8)				IG3: 13<br><br>IG 4: 21		

						CG:<br>Ileum:29<br>Colon, left side:14<br>Colon right side:13<br>Duodenum :3<br>Other:5							
**Rutgeerts 2012 (EXTEND)**	Adalimumab 40 mg subcutaneous every other week<br><br>(n=64)	Placebo (n=65)	IG: 24/40<br><br>CG: 24/41	IG: 37.1 (11.1)<br><br>CG: 37.2 (12.6)	NR	IG:<br>Colon :51<br>Ileum: 48<br>Rectum:19<br>Anal/perianal: 15<br>Gastroduodenum: 3<br>Other:3<br>Jejunum:0<br><br>CG:<br>Colon :57<br>Ileum: 41<br>Rectum:25<br>Anal/perianal: 16<br>Gastroduodenum: 6<br>Other:2<br>Jejunum:1	IG:<br>10.4<br>(8.0)<br><br>CG:<br>9.8 (8.4)	NR	Moderate to severe	Not naïve >50%	Corticosteroids<br>IG 9<br><br>CG 25	>20%	Abbott

**Sandborn 2001a**	IG1: Humicade 10 mg/kg single dose intravenously (n=54)<br><br>IG2: Humicade 20 mg/kg single dose intravenously dose (n=57)<br><br>Retreatment dose intervals of 8 or 12 weeks at a Humicade dose of 10 mg/kg.	Placebo (n=58)	**IG1**<br>**8 weekly**<br>16/13<br>**12 weekly**<br>13/12<br><br>**IG2**<br>**8 weekly**<br>12/16<br>**12weekly**<br>16/13<br><br>**CG**<br>**8 weekly**<br>14/13<br>**12 weekly**<br>15/16	**Median (Range) Years**<br>**IG 1**<br>**8 weekly**<br>28 (18-71)<br>**12 weekly**<br>38 (24-57)<br><br>**IG2**<br>**8 weekly**<br>33(22-76)<br>**12weekly**<br>32 (23-67)<br><br>**CG Placebo**<br>**8 weekly**<br>34 (20-66)<br>**12 weekly**<br>31(18-62)	NR	8weekly/12 weekly<br>**IG1**<br>Ileum: 4/7<br>Ileocolon:17/12<br>Colon:8/6<br><br>**IG 2**<br>Ileum: 8/8<br>Ileocolon:10/15<br>Colon:10/6<br><br>**CG:**<br>Ileum :6/7<br>Ileocolon:11/21<br>Colon: 9/3	**Median (Range) Years**<br><br>**IG1:**<br>**8weekly**<br>4.7 (0-32)<br>**12 weekly**<br>13.8 (1-31)<br><br>**IG2**<br>**8weekly**<br>8.2 (0-24)<br>**12weekly**<br>7.7(0-26)<br><br>**CG Placebo**<br>**8 weekly**<br>11.3 (1-32)<br>**12weekly**<br>7.9 (0-33)	NR	Moderate to severe	Naive	**IG1:**<br>**8weekly :**18<br>**12 weekly:**13<br><br>**IG2**<br>**8weekly:** 14<br>**12weekly:**17<br><br>**CG**<br>**8 weekly:** 9<br>**12weekly:**18	>20%	Celltech
**Sandborn 2001b**	Etanercept 25mg subcutaneo	Placebo (n=20)	IG: 16/7<br>CG:10/10	Median (Range)	NR	IG:<br>Ileum: 3	NR	NR	Moderate to severe	Equally naïve and not	Patients receiving prednisone (no	>20%	Immunex

	usly twice weekly (n=23)			IG 39.3(22-60)<br><br>CG 37.4(20-69)		Ileocolon:18<br>Colon:2<br><br>CG:<br>Ileum: 5<br>Ileocolon:18<br>Colon: 2				native ~50%	upper limit on prednisone dose) or budesonide for a least 4 weeks with a stable dose for at least 2 weeks were eligible<br><br>IG: 10<br>CG: 7		
**Sandborn 2005a (ENACT 1)**	Natalizumab 300 mg intravenous infusion at weeks 0, 4, and 8 (n=724)	Placebo (n=181)	IG: 311/413<br><br>CG: 73/108	IG: 38(12)<br><br>CG: 39(14)	NR	**IG**<br>Ileum: 194 (27)<br>Ileum and colon: 373 (52)<br>Colon: 157 (22)<br><br>**CG**<br>Ileum: 47 (26)<br>Ileocolon: 84 (46)<br>Colon: 49 (27)	Months<br>IG:121(92)<br><br>CG:110 (93)	NR	Moderate to Severe	Naive >50%	Prednisolone (25 mg per day or less) or equivalent, budesonide (6 mg per day or less)<br><br>IG<br>Prednisone: 271<br>Budesonide: 197<br><br>CG:<br>Prednisolone:70<br>Budesonide: 20	>20%	Elan
**Sandborn 2007a**	Adalimumab, 160 mg subcutaneo	Placebo (n=166)	IG:50/109	Mean (years)	NR	IG<br>Colon: 105<br>Ileum: 112	NR	NR	Moderate to severe	Not naive	Prednisone (≤40 mg/d)/ Budesonide	Part of intervention	Elan

	us injections at week 0 and 80 mg at week 2<br><br>(n=159)		CG: 65/109	IG: 37 (12)<br><br>CG: 39 (12)		Rectum: 36<br>Perianal or anus: 27<br>Gastroduodenal:5<br>Jejunum: 6<br>Other: 5<br><br>CG<br>Colon: 113<br>Ileum: 124<br>Rectum:37<br>Perianal or anus: 31<br>Gastroduodenal:16<br>Jejunum: 4<br>Other: 6					(≤9 mg/d) were permitted<br><br>IG:55<br><br>CG:73		
**Sandborn 2007c (PRECISE 1)**	Certolizumab Pegol 400mg subcutaneous at weeks 0, 2, and 4 and then every 4 weeks. (n=331)	Placebo (n=329)	IG:157/174<br>CG:131/197	Mean/ Range<br>IG: 37(12)<br><br>CG: 38(12)		IG:<br>Terminal ileum: 95<br>Colon:87<br>Ileocolon:149<br><br>CG:<br>Terminal ileum: 87<br>Colon:74<br>Ileocolon:167	Median (Range)<br>IG:5(<1–44)<br><br>CG: 5 (<1–40)	NR	Moderate to Severe	Naive >50%	Glucocorticoids only<br>IG 72<br>CG 75<br><br>Glucocorticoids combined with immunosuppressive agents<br>IG 69<br>CG 66	>20%	UCB
**Sandborn 2008 (Population 1)**	Subcutaneous ustekinumab 90	Placebo subcutaneous ustekinum	Subcutaneous ustekinumab 90	Subcutaneous ustekinumab 90		Subcutaneous ustekinumab 90	Subcutaneous ustekinumab 90	NR	Moderate to Severe	Naive >50%	Oral corticosteroids	>20%	Centocor

	mg→placebo (n=25)<br><br>Intravenous ustekinumab 4.5 mg/kg →placebo (n=26)	ab →90 mg (n=26)<br><br>Placebo intravenous ustekinumab →4.5 mg/kg (n=27)	mg→placebo (n=15/10)<br><br>Intravenous ustekinumab 4.5 mg/kg →placebo (n=14/12)<br><br>Placebo subcutaneous ustekinumab →90 mg (n=13/14)<br><br>Placebo intravenous ustekinumab →4.5 mg/kg (n=13/14)	mg→placebo 37 (13)<br><br>Intravenous ustekinumab 4.5 mg/kg →placebo 43(12)<br><br>Placebo subcutaneous ustekinumab →90 mg 37 (14)<br><br>Placebo intravenous ustekinumab →4.5 mg/kg 44(11)		mg→placebo)<br>Ileum: 18<br>Colon: 17<br>Proximal GI Tract: 1<br><br>Intravenous ustekinumab 4.5 mg/kg →placebo<br>Ileum: 22<br>Colon:11<br>Proximal GI Tract: 0<br><br>Placebo subcutaneous ustekinumab →90 mg<br><br>Ileum: 21<br>Colon:14<br>Proximal GI Tract :1<br><br>Placebo intravenous ustekinumab →4.5 mg/kg<br>Ileum:19<br>Colon: 20	mg→placebo 12(10)<br><br>Intravenous ustekinumab 4.5 mg/kg →placebo 13(13)<br><br>Placebo subcutaneous ustekinumab →90 mg 13(11)<br><br>Placebo intravenous ustekinumab →4.5 mg/kg 11(9)				Subcutaneous ustekinumab 90 mg→placebo: 10<br><br>Intravenous ustekinumab 4.5 mg/kg →placebo 20<br><br>Placebo subcutaneous ustekinumab →90 mg: 8<br><br>Placebo intravenous ustekinumab →4.5 mg/kg 19		

						Proximal GI Tract :2							
**Sandborn 2011**	Certolizumab 400mg subcutaneous at weeks 0, 2, and 4.<br><br>(n=223)	Placebo (n=216)	IG: 105/118<br><br>CG: 90/125	IG: 36.3 (12.6)<br><br>CG: 38.8 (12.8)	NR	IG:<br>Terminal ileum: 63<br>Colon:65<br>Ileocolon:90<br>Isolated upper gastrointestinal: 5<br><br>CG:<br>Terminal ileum: 57<br>Colon:61<br>Ileocolon:89<br>Isolated upper gastrointestinal: 8	IG: 7.5 (8.2)<br><br>CG 7.0 (8.5)	NR	Moderate to Severe	Naive	Corticosteroid (with or without immunosuppressants)<br><br>IG 97<br>CG 98	>20%	UCB
**Sandborn 2012 (CERTIFI)- Induction**	Ustekinumab intravenously (in doses of 1, 3, or 6 mg per kilogram of body weight or placebo	Placebo (n=132)	IG: 153/241<br><br>CG: 64/68	IG:38.8(12.6)<br><br>CG:39.5(13.1)	NR	NR	IG: 12.3(8.5)<br><br>CG: 12.4(9.1)	NR	Moderate to Severe	Not naive	Glucocorticoids<br>IG:189<br><br>CG: 73	>20%	Janssen

	(n=394)												
**Sandborn 2012 (CERTIFI) non-responders**	Ustekinumab 90mg (n=109)	Placebo (n=110)	IG:37/72 CG:44/66	IG 73.9(21.2)<br><br>CG: 72.7 (19.8)	NR	**IG**<br>Ileum: 24<br>Colon:34<br>Ileum and colon: 49<br>Proximal GI Tract :11<br><br>**CG**<br>Ileum: 29<br>Colon: 28<br>Ileum and colon: 53<br>Proximal GI Tract :10	Median IG; 10.78 CG: 11.94	NR	Moderate to Severe	Not naive	NR	<20%	Janssen
**Sandborn 2013 (GEMNI II)- Induction**	Vedolizumab 300 mg intravenously at weeks 0 and 2 (n=967)	Placebo (n=148)	IG: 69/79<br><br>CG: 451/516	IG: 38.6 (13.2)<br><br>CG: 35.7 (11.9)	NR	IG:<br>Ileum: 160 (16.5)<br>Colon: :273 (28.2)<br>Ileum and Colon 543(55.2)<br><br>CG:<br>Ileum: 21 (14.2)<br>Colon: 43 (29.1)<br>Ileum and Colon 84(56.8)	IG: 9.2(7.8)<br><br>CG: 8.2(7.8)	NR	Moderate to severe	Equally naïve and not native ~50%	Glucocorticoids only<br><br>IG:336<br>CG: 45<br><br>Glucocorticoids and immunosuppressive agents<br>IG 163<br>CG 26	≤20%	Millenium

**Sandborn 2014**	IG1: Oral tofacitinib 1 mg twice daily(n=36)<br><br>IG2: Oral tofacitinib 5 mg twice daily(n=34)<br><br>IG3: Oral tofacitinib 15 mg twice daily(n=35)	Placebo (n=34)	IG1: 25/11<br><br>IG2: 12/20<br><br>IG3 18/17<br><br>CG: 12/22	IG1 36.6 (12.2)<br><br>IG2 38.7 (10.2)<br><br>IG3 38.1 (11.7)<br><br>CG: 35(12.7)	NR	IG1 Ileal: 6 Colonic: 20 Ileocolonic: 15 Upper disease:1<br><br>IG2 Ileal: 3 Colonic: 18 Ileocolonic: 11 Upper disease: 0<br><br>IG3 Ileal: 10 Colonic:17 Ileocolonic: 17 Upper disease:0<br><br>CG Ileal: 9 Colonic: 14 Ileocolonic: 16 Upper disease: 0	Mean (range) IG1: 11.1 (0.1–28.5)<br><br>IG2 10.9 (0.3–29.3)<br><br>IG3 11.2 (1.5–36.3)<br><br>CG 8.2 (0.1–35.6)	NR	Moderate to severe	Naive	Rectally administered corticosteroids ≥2 weeks before baseline and during treatment	≤20%	Pfizer
**Sandborn 2020d (CELEST)-Induction**	IG1: Upadacitinib 3-mg twice daily (n=39)	Placebo (n=37)	IG1:19/20 IG2:16/21 IG3:19/17 IG4:11/36 IG5:14/19 CG:13/24	Median range IG1 37 (19–66)<br><br>IG2	NR	**IG1** Ileum only: 10(25.6) Colon only: 9(23.1)	Overall Median disease duration: 9.6 years	NR	Moderate to severe	Not naive	Corticosteroid Use IG1:10(28.6) IG2: 21(53.8) IG3: 18(48.6) IG4: 17(47.2)	>20%	AbbVie

	IG2:<br><br>Upadacitinib<br>6-mg, twice daily (n=37)<br><br>IG 3: Upadacitinib<br>12mg twice daily (n=36)<br><br>IG4: Upadacitinib<br>24-mg twice daily (n=36)<br><br>IG5: Upadacitinib<br>24-mg once-daily (n=35)			39 (22-76)<br><br>IG3<br>41(19-70)<br><br>IG4<br>44(20-65)<br><br>IG5 41(21-64)<br><br>CG<br>40 (20-68)		Ileocolonic colon:<br>20(53.1)<br><br>**IG2**<br>Ileum only:<br>6(16.2)<br>Colon only:<br>13(35.1)<br>Ileocolonic:<br>18(48.6)<br><br>**IG3**<br>Ileum only:<br>5 (13.9)<br>Colon only:<br>11(30.6)<br>Ileocolonic:<br>20(55.6)<br><br>**IG4**<br>Ileum only:<br>6(16.7)<br>Colon only:<br>11(30.6)<br>Ileocolonic:<br>19(52.8)<br><br>**IG5**<br>Ileum only:<br>10(28.6)					IG5: 15(41.7)<br>CG: 15(40.5)		

						Colon only: 10(28.6)<br>Ileocolonic: 15(42.9)<br><br>**CG**<br>Ileum only: 9(24.3)<br>Colon only: 6(16.2)<br>Ileocolonic: 22(59.5)							
**Sandborn 2022 (GALAXI 1)**	IG 1: Guselkumab 200mg or 600mg or 1200mg intravenously at weeks 0, 4, and 8 (n=185)<br><br>IG: 2 Ustekinumab 6 mg/kg IV at week 0 and subcutaneous 90 mg	Placebo (n=61)	IG 1 105/80<br><br>IG 2 41/22<br><br>CG: 37/24	IG1 39.6 (13.68)<br><br>IG2 36.1 (12.02)<br><br>CG: 38.9 (12.95)<br><br>:	NR	IG1: Ileum only: 60 (32.4)<br>Colon only: 76(41.1)<br>Ileum and colon: 49(26.5)<br><br>IG2 Ileum only: 12(19.0)<br>Colon only: 29 (46.0)	Overall Mean (SD) 8.8 (8.70)	NR	Moderately to severely active	Not naïve >50%	NR	>20%	Janssen

	at week 8 (n=63)					Ileum and colon: 22(34.9)<br><br>CG:<br>Ileum only: 16 (26.2)<br>Colon only: 26 (42.6)<br>Ileum and colon: 19 (31.1)<br><br>CG:							
**Sandborn 2023-BERGAMONT (cohort 1)**	Etrolizumab (n=241)	Placebo (n=59)	IG:<br>CG:	IG:<br>CG:	IG:<br>CG:	IG:<br>CG:		IG:<br>CG:	IG:<br>CG:	Not naïve >50%		<20%	Hoffmann-La Roche
**Sandborn 2023-BERGAMONT (cohort 3)**	IG1 Etrolizumab 105 mg subcutaneous every 4 weeks (n= 143)<br><br>IG2 Etrolizumab 210mg subcutaneo	Placebo (n=97)	IG1: 74/69<br><br>IG2: 76/69<br><br>CG: 59/38	IG1: 38·3 (13·4)<br><br>IG2: 36·5 (13·1)<br><br>CG: 37·4 (13·7)	NR	IG1 Etrolizumab 105 mg Ileum only: 24<br>Colon only: 28<br>Ileum and colon: 91<br><br>IG2 Etrolizumab 210mg Ileum only: 25	Overall:<br><br>Median (IQR) 6·9 years (IQR 2·7–13·0)	NR	Moderate /severe	Not naïve >50%	Oral corticosteroids were kept stable at 20 mg or less per day prednisone equivalent or 6 mg or less per day budesonide.<br><br>IG 1 54<br>IG 2 53<br>CG: 37	>20%	Hoffmann-La Roche

	us at weeks 0, 2, 4, 8, and 12 (n=145)					Colon only: 37<br>Ileum and colon: 83<br><br>CG:<br>Ileum only: 23<br>Colon only: 17<br>Ileum and colon: 57							
**Sands 2004 (ACCENT II) Induction (Non-responders)**	Infliximab + (intravenous, 5mg/kg maintenance) + Mercaptopurine or Azathioprine (unclear) (n=43)	Placebo + Mercaptopurine or Azathioprine (unclear) (n=44)	All participants: 43/44	All participants: 40 (31-48) median (IQR)	NR	All participants: Ileum 14, Colon 28, Ileum and Colon 45	All participants: 11.7 years (0.3-49.8) median (range)	NR	NR	N aive	All participants: Any dosage: 26<br>>20mg/day: 7	>20%	Centocor
**Sands 2007**	Infliximab (intravenous, 5mg/kg, single dose in week 6) + Azathioprine (NR) (n=27) +	Natalizumab (intravenous,300mg, every 4 weeks, total of 3 infusions) + Infliximab (intraveno	IG: 17/10<br>CG:24/28	IG: 38.9 (13.2)<br>CG: 39.9 (12.6)	NR	IG: Ileum 4, Colonic 8, Ileocolonic 15<br>CG: Ileum 11, Colonic 13, Ileocolonic 28	IG: 120 months (124)<br>CG: 150.3 months (109.4)	NR	NR	Not Naive	IG: 8<br>CG: 14	Part of intervention	Elan and Biogen

	Placebo	us, 5mg/kg, single dose in week 6) + Azathioprine (NR) (n=52)											
**Sands 2010**	IG1: Apilimod 50mg, by mouth, daily (n=73) IG2: Apilimod 100mg, by mouth, daily (n=74)	Placebo (n=73)	IG1: 27/46 IG2: 30/44 CG: 30/43	IG1: 40.6 (12.59) IG2: 41.4 (11.94) CG: 42.6 (13.07)	NR	NR	IG1: 10.82 years (8.64) IG2: 11.35 years (8.98) CG: 11.52 years (9.72)	NR	Moderate	Not naïve >50%	IG1: IG2: CG:	>20%	Synta
**Sands 2014 (GEMINI III)**	Vedolizumab 300mg, intravenously at weeks 0, 2 and 6 (n=209)	Placebo. intravenously at weeks 0, 2 and 6 (n=207)	IG: 91/118 CG: 89/118	IG: 36.9 (20-69) CG: 34.8 (19-77) median (range)	NR:	IG: Ileum 33, Colon 48, Ileocolonic 128 CG: Ileum 29, Colon 52, Ileocolonic 126	IG: 8.4 (0.3-41.8) CG: 8 (0.3-42.9) median (range)	NR	Moderate to severe	Not Naïve	IG: 110 CG: 108	Unclear	Takeda
**Sands 2022 (SEAVUE)**	Adalimumab 160mg, subcutaneous + placebo, intravenou	Ustekinumab 6mg/kg, intravenously on day 0 + subcutaneo	IG: 95/100 CG: 90/101	IG: 37.4 (12.99) CG: 37 (13.23)	NR	IG: ileum 55, colon 34, ileum and colon 103, proximal	IG: 5.8 (7.09) CG: 5.4 (8.36)	NR	Moderate to severe	Naive	IG: steroids including budesonide 75, steroids excluding budesonide	>20%	Janssen

	sly on day 0 + adalimumab 80mg, subcutaneous at week 2, then adalimumab 40mg, subcutaneous every 2 weeks through week 56 (n=195)	us placebo day 0 (4 injections) and at 2 weeks (2 injections), then Ustekinumab 90mg, subcutaneous once every 8 weeks through week 56 + placebo, subcutaneous, once every 2 weeks (n=191)				gastrointestinal tract 17, perianal 41, one or more fistulas 20 (current)<br><br>CG: ileum 60, colon 26, ileum and colon 102, proximal gastrointestinal tract 30, perianal 50, one or more fistulas 17 (current)					46, steroid dose 20mg/day (10-20) mean (SD), budesonide 29, budesonide dose 9mg/day (6-9) mean (SD)<br><br>CG: steroids including budesonide 70, steroids excluding budesonide 42, steroid dose 20mg/day (10-20) mean (SD), budesonide 28, budesonide dose 9mg/day (6-9) mean (SD)		
**Sands 2022 (SERENITY)**	IG1: Mirikizumab 200mg, intravenously, every 4 weeks (n=31)	CG: Placebo, intravenously, every 4 weeks (n=64)	IG1: 17/14<br>IG2: 14/18<br>IG3: 34/30<br>CG: 28/36	IG1: 38.1 (11.8)<br>IG2: 40.4 (13.3)<br>IG3: 37.7 (13.1)<br>CG: 39 (13)	NR	IG1: ileal 6, colonic 14, ileocolonic 11<br>IG2: ileal 5, colonic 10,	IG1: 8.9 (7.4)<br>IG2: 10.8 (9.7)<br>IG3: 8.6 (6.7)	NR	Moderate to severe	Not naïve >50%	IG1: 14<br>IG2: 7<br>IG3: 15<br>CG: 21	>20%	Janssen

	IG2: Mirikizumab 600mg, intravenously, every 4 weeks (n=32)<br>IG3: Mirikizumab 1000mg, intravenously, every 4 weeks (n=64)					ileocolonic 17<br>IG3: ileal 11, colonic 26, ileocolonic 27<br>CG: ileal 11, colonic 25, ileocolonic 28	CG: 10.2 (9.8)						
**Schreiber 2005**	IG1: Certolizumab 100mg, subcutaneously, 3 injections, every 4 weeks (n=74)<br><br>IG2: Certolizumab 200mg, subcutaneously, 3 injections, every 4 weeks (n=72)<br><br>IG1: Certolizum	Placebo (n=73)	IG1: 35/39<br>IG2: 22/50<br>IG3: 32/40<br>CG: 24/49	IG1: 33.5 (18-56)<br>IG2: 40.1 (19-71)<br>IG3: 35.9 (18-67)<br>CG: 35.8 (19-64)	NR	IG1: duodenum 5, ileum 57, cecum 45, ascending colon 35, transverse colon 32, descending colon 39, rectum: 36, perianal 20, other 9<br><br>IG2: duodenum 2, ileum 51, cecum 32, ascending colon 35,	IG1: 7.73 (0-31.8)<br>IG2: 8.84 (0-30.7)<br>IG3: 8.43 (0.2-26.5)<br>CG: 7.95 (0.1-27.6)	NR	Moderate to severe	Naïve	IG1: overall 24, systemic 15, budesonide 9<br>IG2: overall 29, systemic 22, budesonide 8<br>IG3: overall 22, systemic 16, budesonide 6<br>CG: overall 29, systemic 20, budesonide 9	>20%	Celltech

	ab 400mg, subcutaneously, 3 injections, every 4 weeks (n=72)					transverse colon 33, descending colon 40, rectum: 33, perianal 23, other 4<br><br>IG3: duodenum 3, ileum 56, cecum 41, ascending colon 36, transverse colon 37, descending colon 47, rectum: 32, perianal 21, other 7<br><br>CG: duodenum 1, ileum 54, cecum 37, ascending colon 31, transverse colon 33, descending colon 42, rectum: 36, perianal 23, other 5							

**Schreiber 2018a**	IG1: Andecaliximab 150mg, every 2 weeks (n=53) IG2: Andecaliximab 150mg, once a week (n=53) IG3: Andecaliximab 300mg, once a week (n=53)	Placebo, subcutaneous, once a week (n=28)	IG1: 28/25 IG2: 25/28 IG3: 31/22 CG: 13/15	IG1: 38 (12.8) IG2: 39 (13.5) IG3: 42 (11.7) CG: 38 (13.5)	NR	IG1: colonic 10, ileal 7, ileocolonic 36 IG2: colonic 13, ileal 5, ileocolonic 35 IG3: colonic 11, ileal 9, ileocolonic 33 CG: colonic 4, ileal 4, ileocolonic 20	IG1: 12.7 (8.6) IG2: 11.4 (9.23) IG3: 12.6 (10.8) CG: 13.4 (9.3)	NR	Moderate to severe	Not naive	IG1: 24 IG2: 26 IG3: 24 CG: 9	>20%	Gilead
**Schroder 2006**	Infliximab 5mg/kg, intravenously (n= + Methotrexate 20mg, intravenously, 6 infusions at weeks 0-5 followed by Methotrexate 20mg,	Infliximab 5mg/kg, intravenously (n=8)	IG: 6/5 CG: 2/6	IG: 31.6 (9.4) CG: 36.5 (7.3)	NR	IG: terminal ileum 1, ileocolon 7, colon 1, whole gastrointestinal tract 2 CG: terminal ileum 1, ileocolon 5, colon 1, whole	IG: 8.2 (6) years CG: 9.6 (6.8) years	NR	Refractory and dependent on steroids and resistant/intolerant to azathioprine	Naive	IG: 8 CG: 7	Part of intervention	Unclear

	orally, for 28 weeks (n=11)					gastrointestinal tract 1							
**SEQUENCE (Peyrin-Biroulet 2024)**	Ustekinumab, intravenously; ≤55kg: 260mg, >55kg to 85kg: 390mg, >85kg: 520mg + subcutaneous maintenance dose every 8 weeks (n=137) (sample size related to a subgroup comprising the first 50% of the patients to complete the week 24 visit)	Risankizumab 600mg, intravenously, as an induction dose at weeks 0, 4, and 8 + a 360mg subcutaneous maintenance dose every 8 weeks (n=128) (sample size related to a subgroup comprising the first 50% of the patients to complete the week 24 visit)	Data related to the full cohort of patients (IG, n=265; CG, n=255) IG: 131/134 CG: 136/119	Data related to the full cohort of patients (IG, n=265; CG, n=255) IG: 38.3 (13.8) CG: 38 (13.1)	NR	Data related to the full cohort of patients (IG, n=265; CG, n=265) IG: ileal only 45, colonic only 106, ileocolonic 114 CG: ileal only 42, colonic only 102, ileocolonic 111	Data related to the full cohort of patients (IG, n=265; CG, n=255) IG: 7.3 years (0.3-51.9) CG: 7.3 (0.3-40.6)	NR	Moderate to severe	Not naive	Data related to the full cohort of patients (IG, n=265; CG, n=265) IG: 71 CG: 58	<20%	AbbVie
**Summers 1979**	Azathioprine 2.5mg/kg, daily (max dosage	CG1: Placebo (n=77)	IG: 52.5/47.5 %	IG: 29.8 years (11) CG1: 33.7 years (11.7)	NR	IG: extent of small bowel disease 33.1cm	IG: 39 months (61.4) CG1: 47.6	NR	NR	Naive	Treated with steroids within 2 weeks of randomisation:	Part of intervention	Unclear

	250mg)<br>(n=59)	CG2: 5-ASA,<br>1g/15kg, daily (max dosage 5g)<br>(n=74)<br><br>CG3: Prednisone, daily dosage, based on CDAI score;<br><150: 1/4mg/kg,<br>150-300: 1/2mg/kg,<br>>300:3/4mg/kg(n=85)	CG1: 45.5/54.5 %<br><br>CG2: 48.6/51.4 %<br><br>CG3: 52.9/47.1 %	CG2: 29.6 years (10.6)<br>CG3: 31.8 years (11.7)		(37.5), extent of large bowel disease 46.2cm (120.6)<br>CG1: extent of small bowel disease 37.2cm (38.6), extent of large bowel disease 47.8cm (91.8)<br>CG2: extent of small bowel disease 32.2cm (29.6), extent of large bowel disease 54.5cm (101.2)<br>CG3: extent of small bowel disease 44.5cm (59.9),	months (58.6)<br>CG2: 41 months (51.6)<br>CG3: 41.8 (60.9)				IG: 37.3%<br>CG1: 37.7%<br>CG2: 28.4%<br>CG3: 24.7%		

						extent of large bowel disease 36.3cm (36.8) mean (SD)							
**Targan 1997**	IG1: Infliximab 5mg/kg, intravenously (n=27) IG2: Infliximab 10mg/kg, intravenously (n=28) IG3: Infliximab 20mg/kg, intravenously (n=28)	Placebo (n=25)	IG1: 13/14 IG2: 15/13 IG3: 15/13 CG: 10/15	IG1: 37 years (11.8) IG2: 39.3 years (10.6) IG3: 36 years (9.7) CG: 38.5 years (11)	NR	IG1: ileum only 3, ileum and colon 15, colon only 9 IG2: ileum only 4, ileum and colon 14, colon only 10 IG3: ileum only 2, ileum and colon 19, colon only 7 CG: ileum only 8, ileum and colon 10, colon only 7	IG1: 12.5 years (10.3) IG2: 11.5 years (9.6) IG3: 13.5 years (8.8) CG: 10.4 years (7.7)	NR	Moderate to severe	Naive	Prednisone equivalent: IG1: <20mg/day orally: 8; ≥20mg/day orally: 7 IG2: <20mg/day orally: 8; ≥20mg/day orally: 8 IG3: <20mg/day orally: 10; ≥20mg/day orally: 7 CG: <20mg/day orally: 10; ≥20mg/day orally: 6	>20%	Centocor
**Targan 2007 (ENCORE)**	Natalizumab 300mg, intravenously, in 3 infusions at weeks 0,	Placebo, intravenously, in 3 infusions at weeks 0, 4 and 8 (n=250)	IG: 105/154 CG: 102/148	IG: 38.1 years CG: 37.7 years mean, spread NR	NR	IG: ileal 56, colonic 69, ileocolonic 134 CG: ileal 65, colonic	IG: 121.4 months CG: 120.3 mean,	NR	Moderate to severe	Equally naïve and not naïve ~50%	IG: 109 CG: 94	<20%	Elan

	4 and 8 (n=259)					65, ileocolonic 120	spread NR						
**Vermeire 2017 FITZROY induction**	Filgotinib 200mg, once a day (n=130)	Placebo, once a day (n=44)	IG: 59/71<br>CG: 18/26	IG: 37.4 years (11.6)<br>CG: 35.1 years (11.8)	NR	IG: ileal 24, colonic 29, ileocolonic 77<br>CG: ileal 7, colonic 6, ileocolonic 31	IG: 8.8 years (8.5)<br>CG: 6.8 (5.7)	NR	Moderate to severe	Not naïve >50%	IG: 20.7 (8.6)<br>CG: 21.3 (8.6) mean daily dose (SD)	>20%	Takeda
**Vermeire 2017 FITZROY non-responders**	Filgotinib 200 mg once a day (n=25)	Placebo (n=9)	NR (Data available for the full cohort only)	NR (Data available for the full cohort only)	NR	NR (Data available for the full cohort only)	NR (Data available for the full cohort only)	NR	Moderate to severe	Not naïve >50%	NR (Data available for the full cohort only)	Discontinued before the study	Takeda
**Vermeire 2025 - DIVERSITY A**	IG1:Oral filgotinib 200 mg<br><br>IG2: Oral filgotinib 100 mg<br><br>All once daily for 11 weeks.	CG: Placebo once daily for 11 weeks	IG1: 112/110<br><br>IG2: 139/106<br><br>CG: 107/130	IG1: 39(13.8)<br><br>IG2: 39(14.1)<br><br>CG: 38(14.0)	NR	IG1: Ileum only: 31<br>Colon only: 90<br>Both: 101<br><br>IG2: Ileum only: 31<br>Colon only: 104<br>Both: 110<br><br>CG: Ileum only: 33	IG1<br><1 year: 20<br>1-3 years: 40<br>3-7 years: 59<br>>7 years: 103<br><br>IG2<br><1 year: 25<br>1-3 years: 40<br>3-7 years: 58	NR	Moderate to severe	Not naive	IG1: 74<br>IG2: 84<br>CG: 76	>20%	Galapagos and Gilead

						Colon only: 98<br>Both: 106	>7 years: 122<br><br>CG<br><1 year: 24<br>1-3 years: 49<br>3-7 years: 40<br>>7 years: 124						
**Vermeire 2025 - DIVERSITY B**	IG1:Oral filgotinib 200 mg<br><br>IG2: Oral filgotinib 100 mg<br><br>All once daily for 11 weeks.	CG: Placebo once daily for 11 weeks	IG1: 88/114<br><br>IG2: 101/127<br><br>CG: 114/115	IG1: 39(14.2)<br><br>IG2: 42(13.5)<br><br>CG: 39(12.5)	NR	IG1:<br>Ileum only: 30<br>Colon only: 67<br>Both: 105<br><br>IG2:<br>Ileum only: 39<br>Colon only: 85<br>Both: 104<br><br>CG:<br>Ileum only: 34<br>Colon only: 106<br>Both: 89	IG1<br><1 year: 0<br>1-3 years: 13<br>3-7 years: 53<br>>7 years: 136<br><br>IG2<br><1 year: 3<br>1-3 years: 16<br>3-7 years: 46<br>>7 years: 163<br><br>CG	NR	Moderate to severe	Not naive	IG1: 74<br>IG2: 81<br>CG: 91	>20%	Galapagos and Gilead

							<1 year: 3<br>1-3 years: 23<br>3-7 years: 44<br>>7 years: 159						
**Watanabe 2012 - Induction**	IG1: Adalimumab 80/40mg at baseline and week 2 (n=34)<br>IG2: Adalimumab 160/80mg at baseline and week 2 (n=33)	Placebo at baseline and week 2 (n=23)	IG1: 16/18<br>IG2: 20/13<br>CG: 16/7	IG1: 30.6 (9.3)<br>IG2: 32 (9.6)<br>CG: 30.4 (6.9) years, mean (SD)	NR	NR	IG1: 9.2 (6.6)<br>IG2: 11 (7.1)<br>CG: 7.9 (4.7) years, mean (SD)	NR	Moderate to severe	Not naïve >50%	IG1: 6<br>IG2: 8<br>CG: 5	<20%	Takeda
**Watanabe 2020 - Induction**	Vedolizumab 300mg, intravenously at weeks 0, 2 and 6 (n=79)	Placebo, intravenously at weeks 0, 2 and 6 (n=78)	IG: 51/28<br>CG: 52/26	IG: 33.9 (12.3)<br>CG: 32.6 (10.9) years, mean (SD)	NR	IG: ileal 13, colonic 11, ileocolonic 55<br>CG: ileal 9, colonic 19, ileocolonic 50	IG: 9 (6.2)<br>CG: 9.1 (6.5)	NR	Moderate to severe	Not naïve >50%	IG: 22<br>CG: 18	>20%	Takeda
**Winter 2004**	IG1: Certolizumab 1.25mg/kg *,	Placebo, intravenously, single infusion (n=25)	IG1: 0/2<br>IG2: 12/13<br>IG3: 6/11<br>IG4: 10/13<br>CG: 6/19	IG1: 36.5 (31-42)<br>IG2: 36.4 (21-26)	NR	NR	IG1: 9.28 (7.7-10.9)	NR	Moderate to severe	Naive >50%	IG1: 1<br>IG2: 6<br>IG3: 6<br>IG4: 6<br>CG: 7	>20%	Celltech

	intravenously, single infusion (n=2)<br>IG2: Certolizumab 5mg/kg, intravenously, single infusion (n=25)<br>IG3: Certolizumab 10mg/kg, intravenously, single infusion (n=17)<br>IG4: Certolizumab 20mg/kg, intravenously, single infusion (n=23)<br><br>*replaced with a dose of 10mg/kg due to low efficacy			IG3: 40.3 (18-64)<br>IG4: 33.3 (19-60)<br>CG: 32.1 (18-56) years, mean (range)			IG2: 7.52 (0.6-17)<br>IG3: 10.2 (0.9-26)<br>IG4: 7.94 (1.3-18.9)<br>CG: 7.74 (0.1-21.9) years, mean (SD)						
**Ye 2019**	Infliximab 5 mg/kg, intravenously +	CTP13 5 mg/kg, intravenously	IG: 60/49<br>CG: 63/48	IG: 32 (24-45)<br>CG: 35 (26-46)	NR	NR	IG: 5.3 years (7.27)	NR	NR	Naive	IG: 33<br>CG: 37	Part of intervention	Celltrion

	Azathioprine (dosage NR) (n=109)	+ Azathioprine (dosage NR) (n=111)		median (IQR)			CG: 4.2 years (4.59) mean SD						

eTable2. Included studies' efficacy outcome definitions, baseline disease activity, and time of primary outcome measurement (72 RCTs)

**Study ID (Author, Year)**	**Intervention group, IG (numbers randomised)**	**Comparator group, CG (numbers randomised)**	**Length of the intervention**	**Duration of the randomize trial**	**Follow up after the randomize trial has ended (dosage)**	**Definition of clinical remission**	**Definition of clinical response**	**Definition of endoscopic remission**	**Baseline clinical disease activity (CDAI, HBI or IBDQ) mean (SD)**	**Baseline endoscopic disease activity (Rutgeerts score, CDEIS, or SES-CD) mean (SD)**	**Week of primary outcome measurement**
**Allez 2023 (TRIDENT Part 1)**	Tesnatilimab (400 mg subcutaneous at Week 0, 200 mg every 2 weeks) (n=73)	Placebo (4 subcutaneous every 2 weeks) (n=72)	24 weeks	24 weeks	14 weeks (Stop taking study drugs)	CDAI < 150	≥100-point reduction from baseline in CDAI or CDAI <150	SES-CD score ≤2	CDAI IG: 316.2 (63.5) CG: 309.3 (56.5)	SES-CD IG: Remission (0–2)- 9 Mild (3–6)- 14 Moderate (7–16)- 27 Severe (>16)- 22 CG: Remission (0–2)- 14 Mild (3–6)- 22 Moderate (7–16)- 26 Severe (>16)- 9	12
**Allez 2023 (TRIDENT Part 2)**	Tesnatilimab (400 or 150 or 50 mg subcutaneous at week 0, then half	Placebo (subcutaneous every 2 weeks from Week 0–4, Week 8, every	24 weeks	24 weeks	64 weeks (IG1: Tesnatilimab 400 or 150 or 50 mgsubcutaneous every 4 weeks from week24-72	CDAI < 150	≥100-point reduction from baseline in CDAI or CDAI <150	SES-CD score ≤2	IG1: 309.5 (55.0) IG2: CG: 313.1 (47.4)	SES-CD IG1: Remission (0–2)- 24 Mild (3–6)- 37 Moderate (7–16)- 61 Severe (>16)- 24	12

	dosage at weeks 2, 4, 8, 12, 16, and 20) (n=148)<br><br>Ustekinumab (6 mg/kg intravenous injection at week 0, 90mg subcutaneous at week 8 and 16) (n=47)	2 weeks from Week 12–16, and Week 20) (n= 48)			IG2: Ustekinumab 90 mg subcutaneous every 8 weeks from week24-72)					IG2: Remission (0–2)- 8 Mild (3–6)- 15 Moderate (7–16)- 12 Severe (>16)- 12<br>CG: Remission (0–2)- 11 Mild (3–6)- 11 Moderate (7–16)- 24 Severe (>16)- 2	
**Ardizzone 2003**	Azathioprine (2 mg/kg per day orally) (n=27)	Methotrexate (25 mg/week intravenous injection for 3 months, then oral 25 mg/week) (n=27)	6 months	6 months	None	CDAI < 150	NA	NA	IG: 225.96 (62.32)<br>CG: 213.36 (58.67)	NA	12
**Arora 1999**	Methotrexate (n=15)	Placebo (n=18)	1 year	1 year	Unclear	Treatment failure was defined as	NA	NA	NA	NA	12

						no improvement in the CDAI at 3 months with any reduction in steroid dose or the development of severe clinical illness					
**Candy 1995**	Azathioprine (2.5 mg/kg) (n=33)	Placebo (n=30)	12 weeks	12 weeks	12 months (IG1: Azathioprine IG2: Placebo dosage unclear)	CDAI < 150	NA	NA	CDAI IG: 301 (264-358) CG: 282 (240-356) Median (IQR)	NA	12
**Chen 2020**	Adalimumab (160 mg at week 0, 80 mg at week 2, and 40 mg at weeks 4 and 6) + Azathioprine (unclear) (n=102)	Azathioprine (unclear) (n=103)	Week 0, 2, 4 and 6	8 weeks	18 weeks (Adalimumb 40 mg at week 8 and every other week through week 26)	CDAI<150	Decrease in CDAI from baseline by ≥70 points	NA	CDAI IG: 272.1 (48.1) CG: 274.7 (49.1)	NA	4

**Chen 2025 (NCT03234907)**	Vedolizumab (300 mg intravenous once at Weeks 0, 2, and 6) (n=144)	Placebo (intravenous once at Weeks 0, 2, and 6) (n=71)	6 weeks	10 weeks	Week 10-58 maintenance (continued the same study drug, placebo for every 4 weeks and vedolizumab 300 mg for every 8 weeks up to Week 58)	CDAI < 150	Decrease of ≥70 points from baseline	NR	CDAI<br>IG: 298.7(40.19)<br>CG: 295.2(53.36)	SES-CD<br>**IG: 16.1(9.33)**<br>**CG: 16.7(9.62)**	10
**Colombel 2007 (CHARM)-Induction (non-responders)**	Adalimumab every other week (40 mg every other week) (n= 88)<br><br>Adalimumab Weekly (40 mg weekly) (n=100)	Placebo (n=91)	56 weeks	56 weeks	4 weeks (Intervention unclear)	CDAI<150	Decrease in CDAI from baseline by ≥70 points	NA	CDAI<br>301.6 (56.4)<br>IBDQ, median (range)<br>120.0 (55–197)	NA	22
**Colombel 2010 (SONIC)**	Infliximab (5 mg/kg intravenous infusion at weeks	Azathioprine (2.5 mg/kg daily oral capsules) (n=170)	30 weeks	30 weeks	20 weeks (IG1: 5 mg/kg intravenous infusion at week 30, 38, and 46	CDAI < 150	Decrease in CDAI by ≥70 points. (70-point response) or ≥100 points	NA	CDAI<br>IG1: 284.8±62.1<br>IG2: 289.9±55.0<br>CG: 287.2±52.9	NA	26

	0, 2, and 6, then every 8 weeks) (n=169)<br><br>Infliximab (5 mg/kg intravenous infusion at weeks 0, 2, and 6, then every 8 weeks) + Azathioprine (2.5 mg/kg daily oral capsules) (n=169)				IG2: Infliximab 5 mg/kg intravenous infusion at week 30, 38, and 46 and azathioprine 2.5 mg/kg daily oral capsules CG: 2.5 mg/kg daily oral capsules)		(100-point response)							
**D'Haens 1999**	Infliximab 5mg (5 mg/kg intravenous, single infusion) (n=7) Infliximab 10mg (10 mg/kg	Placebo (n=8)	single infusion at week 0	4 weeks	None	NA (continuously measured on CDAI)	NA	NA (continuously measured on CDEIS)	CDAI<br>IG1: 314.4 (18.3)<br>IG2: 336.8 (22.1)<br>IG3: 300.9 (20.3)<br>CG: 276.9 (20.3)	CDEIS<br>IG1: 15.1 (6.9)<br>IG2: 10.6 (7.8)<br>IG3: 13.3 (6.9)<br>CG: 8.4 (6.3)	4			

	intravenous, single infusion) (n=7) Infliximab 20mg (20 mg/kg intravenous, single infusion) (n=8)										
**D'Haens 2008**	Infliximab (5 mg/kg at weeks 0, 2, and 6) + Azathioprine (2–2.5 mg/kg daily or methotrexate 25 mg each week for 12 weeks with the dose reduced to 15 mg per week thereafter if	Corticosteroids (Methylprednisolone 32 mg every day for 3 weeks, then tapering by 4 mg per week; budesonide 9 mg every day for 8 weeks with tapering to discontinuation by 3 mg	104 weeks	104 weeks	None	CDAI<150	CDAI score: for patients with an initial score between 200 and 250 points, a 50-point decrement was regarded as a response; corresponding criteria for patients with scores between 250 and 350 points and scores greater than 350 points were 75 and 100 points, respectively	NR	CDAI<br>IG: 330 (92)<br>CG: 306 (80)<br>IBDQ<br>IG: 122 (33)<br>CG: 136 (28)	NR	14

	intolerant to azathioprine) (n=67)	per week) (n=66)									
**D'Haens 2022 (ADVANCE)**	Risankizumab (600 or 1200 mg intravenous at Weeks 0, 4, and 8) (n=675)	Placebo (intravenous at Weeks 0, 4, and 8) (n=186)	12 weeks	12 weeks	140 days	CDAI < 150 or stool frequency of 2·8 or less, plus average daily abdominal pain score of 1 or less, and both not worse than baseline	Reduction in CDAI of ≥100 points from baseline	SES-CD score ≤4 and at least 2-point reduction and no sub score greater than 1	CDAI<br>IG1: 311.2 (62.4)<br>IG2: 311.5 (68.4)<br>CG: 319.2 (59.4)	SES-CD<br>IG1: 14.7 (7.7)<br>IG2: 13.4 (6.5)<br>CG: 13.8 (6.8)	12
**D'Haens 2022 (MOTIVATE)**	Risankizumab (600 or 1200 mg intravenous at Weeks 0, 4, and 8) (n=382)	Placebo (intravenous at Weeks 0, 4, and 8) (n=187)	12 weeks	12 weeks	140 days	CDAI < 150 or stool frequency of 2·8 or less, plus average daily abdominal pain score of	Reduction in CDAI of ≥100 points from baseline	SES-CD score ≤4 and at least 2-point reduction and no sub score greater than 1.	CDAI<br>IG1: 310.7 (63.6)<br>IG2: 312.5 (61.2)<br>CG: 319.6 (69.8)	SES-CD<br>IG1: 14.4 (7.6)<br>IG2: 15.1 (7.6)<br>CG: 15.0 (8.1)	12
**Duan 2013**	Infliximab (5 mg/kg at week 0, 2, 6, and then	Azathioprine (2.5 mg/kg qd) (n=8)	26 weeks	26 weeks	None	CDAI < 150	Decrease in CDAI from baseline by ≥70 points	NA	NR	NA	26

	every 8 weeks) (n=8)<br><br>Infliximab (5 mg/kg at week 0, 2, 6, and then q8w) + Azathioprine (2.5 mg/kg qd) (n=8)										
**Ewe 1993**	Azathioprine (2.5 mg/kg every day) (n=21)	Placebo (n=21)	4 months	4 months	16 weeks (Intervention unclear)	CDAI < 150	NA	NA	NA	NA	16
**Feagan 1995**	Methotrexate (25 mg intramuscular weekly) (n=94)	Placebo (intramuscular weekly) (n=47)	16 weeks	16 weeks	None	CDAI ≤ 150	NA	NA	CDAI<br>IG: 181 (11)<br>CG: 190 (14)<br>IBDQ<br>IG: 162 (3.4)<br>CG: 159 (5.2)	NA	16
**Feagan 2008c**	Vedolizumab (2.0 or 0.5 mg/kg	Placebo (intravenous on Days 1 and 29)	on days 1 and 29	180 days	None	CDAI < 150	Decrease in CDAI by ≥70 points (70-point response) or	NA	CDAI<br>IG1: 296.6 (55.37)<br>IG2: 288.1 (48.63)	NA	8

	intravenous on Days 1 and 29) (n= 127)	(n=58)					≥100 points (100-point response)		CG: 288.0 (45.83)<br>IBDQ<br>IG1: 131 (26)<br>IG2: 131 (26)<br>CG: 122 (28)		
**Feagan 2014**	Methotrexate (10 mg/week subcutaneous, then 20 mg at week3, 25 mg/week through week 50) + Infliximab (5 mg/kg intravenous at weeks 1, 3, 7, 14, 22, 30, 38, and 46) (n=63)	Infliximab (5 mg/kg intravenous at weeks 1, 3, 7, 14, 22, 30, 38, and 46) (n=63)	50 weeks	50 weeks	Week 50-66 (Intervention unclear)	CDAI<150	NA	NA	CDAI<br>IG: 207.8 (110.8)<br>CG: 207.6 (100.3)	NA	14
**Feagan 2015a (UNITI-1)**	Ustekinumab (130 mg or 6 mg/kg single	Placebo (single intravenous infusion) (n=247)	Single infusion at week 0	8 weeks	None	CDAI < 150	Decrease in CDAI by ≥70 points (70-point response) or ≥100 points	NA	CDAI<br>IG1: 321.0 (64.7)<br>IG2: 327.6 (62.0)<br>CG: 319.0 (59.7)	NA	8

	intravenous infusion at week 0) (n=494)						(100-point response)				
**Feagan 2015b (UNITI-2)**	Ustekinumab (130 mg or 6 mg/kg single intravenous infusion at week 0) (n=418)	Placebo (n=210)	Single infusion at week 0	8 weeks	None	CDAI < 150	Decrease in CDAI by ≥70 points (70-point response) or ≥100 points (100-point response)	NA	CDAI<br>IG1: 304.1 (57.0)<br>IG2: 302.2 (58.9)<br>CG: 302.2 (61.7)	NA	8
**Feagan 2017**	Risankizumab (200 or 600 mg intravenous at Weeks 0, 4, 8) (n=82)	Placebo (intravenous at Weeks 0, 4, 8) (n=39)	8 weeks	12 weeks	12-week open-label intravenous therapy or wash-out period (600 mg risankizumab), then 6-week subcutaneous therapy (four injections of risankizumab 180 mg separated by	CDAI < 150	Decrease from baseline in CDAI score by at least 100 points or a total CDAI score less than 150	CDEIS score of ≤4 at week 12 (≤2 for patients with initial isolated ileitis)	CDAI<br>IG: 300 (247-349)<br>CG: 295 (237-386)<br>median (IQR)	CDEIS<br>IG: 12 (9-17)<br>CG: 11 (8-18)<br>median (IQR)	12

					8-week intervals)						
**Ferrante 2024 (VIVID-1)**	Mirikizumab: a single dose intravenously at weeks 0, 4, and 8	Ustekinumab: a single intravenous dose at week 0 followed by placebo intravenous administrations at weeks 4 and 8<br><br>Placebo: a single dose intravenously at weeks 0, 4, and 8	12 weeks	12 weeks	Treat-through design up to week 52	CDAI <150	PRO (≥30% decrease in stool frequency or abdominal pain score, or both, and neither score worse than baseline)	SES-CD total score of 4 or less and at least a 2-point reduction from baseline with no subscore of more than 1	CDAI<br><br>Miri: 323.1(85.8)<br><br>Uste: 318.5(93.2)<br><br>Placebo: 318.9(86.2)	SES-CD<br><br>Miri: 13.5 (6.6)<br><br>Uste: 13.9(6.6)<br><br>Placebo: 13.1(6.0)	12
**Ghosh 2003**	Natalizumab (1 Infusion of 3 mg/kg or 2 Infusion of 3 mg/kg or 2	Placebo (n=63)	12 weeks	12 weeks	None	CDAI < 150	Decrease in CDAI by 70 or more points from baseline	NA	NR	NA	6

	Infusion of 6 mg/kg intravenous infusion four weeks apart) (n=185)										
**Gordon 2001**	Natalizumab (3 mg/kg single intravenous infusion) (n=18)	Placebo (Single intravenous infusion) (n=12)	Single intravenous infusion	12 weeks	None	CDAI < 150	NA	NA	CDAI<br>IG: 258 (122–436)<br>CG: 273 (191–420)<br>mean (range)	NA	2
**Hanauer 2002-ACCENT I (non-responders)**	Infliximab 5mg (5 mg/kg intravenous infusion at week 0, 2 and 6 and every 8 weeks thereafter until week 46) (n= 79)<br>Infliximab 5+10mg	Placebo (5 mg/kg intravenous infusion infliximab at week 0, 2 and 6 and every 8 weeks thereafter until week 46) (n=78)	46 weeks	54 weeks	None	CDAI<150	Decrease in CDAI by 70 or more points from baseline and at least 25% reduction in the total score	NA	CDAI, all participants<br>291 (249–340)<br>IBDQ: 125 (106–145)<br>median (IQR)	NA	28

	(5 mg/kg intravenous infusion at week 0, 2 and 6 and 10 mg/kg every 8 weeks until week 46) (n= 81)										
**Hanauer 2006 (CLASSIC I)**	Adalimumab (40 mg or 80 mg or 160 mg subcutaneous at week 0 and half dose at week 2) (n=224)	Placebo (at week 0 and 2) (n=74)	At week 0 and week 2	4 weeks	None	CDAI < 150	Decrease in CDAI by ≥70 points (70-point response) or ≥100	NA	CDAI<br>IG1: 299 (57)<br>IG2: 301 (61)<br>IG3: 295(52)<br>CG: 296(60)<br>Mean (SD)<br>IBDQ<br>IG1: 129 (81-218)<br>IG2: 128 (63-200)<br>IG3: 127 (37-192)<br>CG: 131 (52-200)<br>Median (range)	NA	4
**Hanauer 2021 (VOLTAIRE-CD)**	Adalimumab (160 mg subcutaneous on day 1, 80 mg subcutaneous on day 15,	BI695501 (160 mg subcutaneous on day 1, 80 mg subcutaneous on day 15,	24 weeks	24 weeks	24 weeks	CDAI < 150	Decrease in CDAI by ≥70 points	NA	CDAI<br>IG: 296·0 (262·0–328·0)<br>CG: 297·0 (266·0–347·0)<br>median (IQR)	SES-CD<br>IG: <16 60%, ≥ 16 40%<br>CG: <16 59%, ≥ 16 41%	24

	followed by 40 mg every 2 weeks) (n=74)	followed by 40 mg every 2 weeks) (n=72)									
**Hart 2025 (GRAVITI)**	IG1: Guselkumab subcut 400mg every 4 weeks (switched to 200mg after week 16)<br><br>IG1: Guselkumab subcut 400mg every 4 weeks (switched to 100mg after week 16)	CG: Placebo identical to IG	16 weeks	16 weeks	Dosage switch and maintenance continuation between 16-48 weeks	CDAI <150	CDAI score >= 100 points or CDAI score < 150	SES-CD score <4 and at least a 2-point reduction from baseline and no subscore greater than 1 in any individual component	CDAI<br><br>IG1: 297.3(54.69)<br><br>IG2: 300.4(54.32)<br><br>CG: 293(49.09)	SES-CD<br><br>IG1: 11.87 (7.12)<br><br>IG2: 12.2 (6.85)<br><br>CG: 12.0(6.89)	12
**Jairath 2025 (RELIEVE UCCD)**	IG1: Duvakitug subcutaneously at 2250 mg	CG: Placebo<br><br>subcutaneously at 2250 mg	14 weeks	14 weeks	Safety outcomes up to week 18	Mayo score of ≤2 points	Mayo score decrease of at least 2 points AND at least a 30% reduction from baseline	Mayo endoscopic subscore of 0	CDAI<br><br>IG1: 304.7(56.8)<br><br>IG2: 294.1(63.6)	SES-CD<br><br>IG1: 12.7(6.6)<br><br>IG2: 12.3(5.8)	14

	loading dose followed by duvakitug 450 mg<br><br>IG2: Duvakitug subcutaneously at 2250 mg loading dose followed by duvakitug 900 mg	loading dose followed by placebo							CG: 309.4(65.8)	CG: 12.0(5.7)	
**Lemann 2006**	Azathioprine or 6-MP (2–3 mg/kg/day for AZA and 1–1.5 mg/kg/day for 6-MP) (n= 58)	Infliximab (5 mg/kg IV at weeks 0, 2, and 6) Azathioprine or 6-MP (2–3 mg/kg/day for AZA and 1–1.5 mg/kg/day for 6-MP) (n=57)	24 weeks	24 weeks	Week 24-52	CDAI < 150	NA	NA	Unclear	NA	24

**Loftus 2023 U-EXCEL**	Upadacitinib (45 mg once daily) (n=350)	Placebo (once daily) (n=176)	12 weeks	12 weeks	52 weeks maintenance	CDAI < 150	Decrease of ≥100 points from baseline	SES-CD ≤4 and at least 2-point reduction from Baseline and no sub score >1 in any individual variable	CDAI<br>IG: 292.4 (81.3)<br>CG: 293.9 (85.4)	SES-CD<br>IG: 13.7 (7.3)<br>CG: 13.6 (7.0)	12
**Loftus 2023 U-EXCEED**	Upadacitinib (45 mg once daily) (n=324)	Placebo (once daily) (n=171)	12 weeks	12 weeks	52 weeks maintenance	CDAI < 150	Decrease of ≥100 points from baseline	SES-CD ≤4 and at least 2-point reduction from Baseline and no sub score>1 in any individual variable	CDAI<br>IG: 306.6 (89.4)<br>CG: 308.1 (84.3)	SES-CD<br>IG: 15.2 (7.8)<br>CG: 14.9 (7.8)	12
**Mantzaris 2004**	Infliximab (5 mg/kg intravenous at weeks 0, 2, 6) (n=23)	Infliximab (5 mg/kg intravenous at weeks 0, 2, 6) + Azathioprine (2.5 mg/kg PO) (n=27)	8 weeks	8 weeks	8-52 weeks maintenance, off steroids<br>IG: infliximab 5 mg/kg intravenous every 8 weeks<br>CG: infliximab 5 mg/kg intravenous every 8 weeks and azathipprine 2.5 mg/kg/day	CDAI < 150	NA	NA	CDAI<br>IG:220 (55)<br>CG: 232 (65)	NA	6
**Mate-Jimenez 2000**	Azathioprine (1.5 mg/kg/day oral)	Methotrexate (15 mg/week oral)	30 weeks	30 weeks	76 weeks maintenance (intervention unclear)	CDAI< <150 and normal serum orosomuco	NA	NA	CDAI<br>IG: 191 (13)<br>CG1: 200 (14)<br>CG2: 215 (10)	NA	30

	(n=16)	(n=15)<br><br>5-ASA (3g/day oral) (n=7)				id concentration							
**Matsumoto 2016 - DIAMOND**	Adalimumab (160 mg subcutaneous at Week 0, 80 mg at Week 2, and there-after 40 mg at every other week up to 52 weeks) + Azathioprine (25 mg or 50 mg/day increased to a maximum of 100 mg during the initial	Adalimumab (160 mg subcutaneous at Week 0, 80 mg at Week 2, and there-after 40 mg at every other week up to 52 weeks) (n=85)	52 weeks	52 weeks	None	CDAI <150	Reduction of CDAI from the baseline value by more than 70	Decrease of SES-CD of at least 8 points from the baseline, or SES-CD ≤ 4	CDAI<br>IG: 276 (62)<br>CG: 265 (43)	SES-CD<br>IG: 15 (8)<br>CG: 16 (8)	26		

	four weeks) (n=92)										
**Oren 1997**	Azathioprine (50 mg/day oral) (n=32)	Methotrexate (12.5 mg/week oral) (n=26) Placebo (n=26)	9 months	9 months	None	HBS<3 and no steroids	NA	NA	NA	NA	36
**Panaccione 2024 (GALAXI-2)**	(Group 1) Guselkumab 200 mg IV q4w Followed by 200 mg SC q4w<br><br>(Group 2) Guselkumab 200 mg IV q4w Followed by 100 mg SC q8w	(Group 3) Ustekinumab 6 mg/kg IV Followed by 90 mg SC q8w<br><br>(Group 4) Placebo q4w Followed by Placebo q4w or Ustekinumab 6 mg/kg IV Then 90 mg SC q8w	12 weeks	12 weeks		CDAI<150	≥100-point reduction from baseline in CDAI or CDAI <150	SES-CD ≤4 and a ≥2-point reduction from baseline and no subscore greater than 1 in any individual component	Pooled GALAXI 2/3<br><br>Group 1: 295.9(52.7)<br>Group 2: 296.3(54.3)<br>Group 3: 293.1(52.0)<br>Group 4: 293.4(52.7)	Pooled GALAXI 2/3<br><br>Group 1: 12.5(7.2)<br>Group 2: 13.2(7.4)<br>Group 3: 12.9(7.0)<br>Group 4: 13.3(7.5)	12
**Panaccione 2024**	(Group 1) Guselku	(Group 3) Ustekinu	12 weeks	12 weeks	Treat through up to 48 weeks	CDAI<150	≥100-point reduction from baseline	SES-CD ≤4 and a ≥2-point reduction from	Pooled GALAXI 2/3	Pooled GALAXI 2/3	12

**(GALAXI-3)**	mab 200 mg IV q4w Followed by 200 mg SC q4w<br><br>(Group 2) Guselkumab 200 mg IV q4w Followed by 100 mg SC q8w	mab 6 mg/kg IV Followed by 90 mg SC q8w<br><br>(Group 4) Placebo q4w Followed by Placebo q4w or Ustekinumab 6 mg/kg IV Then 90 mg SC q8w					in CDAI or CDAI <150	baseline and no subscore greater than 1 in any individual component	Group 1: 295.9(52.7)<br>Group 2: 296.3(54.3)<br>Group 3: 293.1(52.0)<br>Group 4: 293.4(52.7)	Group 1: 12.5(7.2)<br>Group 2: 13.2(7.4)<br>Group 3: 12.9(7.0)<br>Group 4: 13.3(7.5)	
**Panes 2017 Induction**	Tofacitinib (5, 10 or 15 mg twice daily) (n=188)	Placebo (n=92)	8 weeks	8 weeks	26 weeks maintenance (Placebo, tofacitinib 5 or 10 mg twice daily)	CDAI < 150	Decrease in CDAI by ≥70 points (70-point response) or ≥100 points (100-point response)	NA	CDAI<br>IG1: 314 (53.06)<br>IG2: 320 (61.66)<br>IG3: 328 (76.66)<br>CG: 313 (67.14)	NA	12
**Present 1999**	IG1: Infliximab 5mg/kg intravenous at weeks 0,2,6) (n=31)	Placebo (n=31)			After the first infusion of study medication, patients returned or clinical and laboratory	CDAI < 150	absence of any draining fistulas at two consecutive visits	reduction of 50 percent or more from base line in the number of draining fistulas observed at two or more	Median (IQR)<br><br>IG1: 163 (99–284)<br><br>IG2: 203 (112–254)	NA	NA

	IG2: Infliximab 10mg/kg at weeks 0,2,6) (n=32)		6 weeks	6 weeks	assessments at weeks 2, 6, 10, 14, and 18. Blood samples were drawn at each study visit and at weeks 26 and 34 to determine the serum concentration of infliximab.			consecutive study visits	CG: 162 (126–265)		
**Reinisch 2008**	Azathioprine (n=52)	Placebo (n=29)<br><br>Everolimus (n=63)	3months	3months	12months	NA	CDAI reduction ≥70 from the baseline	NA	**CDAI**<br>IG1: 304 (221–425)<br><br>IG2: 282 (225–435)<br><br>CG: 280(230–432)<br><br>**IBDQ**<br>IG1: 120 (51–187)<br><br>IG2: 126(46–198)<br><br>CG: 120 (70–147)	IG:<br>CG:	12

**Rutgeerts 2006**	IG1: Onercept 10 mg (n=44)<br><br>IG2: Onercept 25mg (n=42)<br><br>IG3: Onercept 35mg (n=40)<br><br>IG4: Onercept 50mg (n=43)<br><br>All given subcutaneously 3 times weekly	Placebo (n=38)	8 weeks	8weeks	Follow-up visits at 2, 4, and 12 weeks after treatment	CDAI<150	Decrease in CDAI by ≥100 points	NA	IG1:307.2 (948.2)<br><br>IG2: 312.2 (52.4)<br><br>IG3:325.3(44.0)<br><br>IG4 315.8 (49.6)<br><br>CG: 311.9 (51.4)	NA	8
**Rutgeerts 2012 (EXTEND)**	Adalimumab 40 mg subcutaneous every other week<br><br>(n=64)	Placebo (n=65)	52 weeks	52 weeks	None	NA	Decrease in CDAI of at least 70 points from baseline	CDEIS score of ≤4	IG: 318.7 (68.6)<br><br>CG: 321.1 (72.1)	**CDEIS**<br>IG:<br>9.8(7.4)<br><br>CG:<br>10.9 (7.2)<br><br>**SES-CD**<br>IG: 11.8 (8.2)<br><br>CG: 13.3 (8.4)	4

**Sandborn 2001a**	Humicade (n=111)<br><br>Retreatment dose intervals of 8 and 12 weeks at a CDP571 dose of 10 mg/kg	Placebo (n=58)	8-week repeating dose group (16 weeks)<br><br>12-week repeating dose group (12 weeks)	24weeks	None	CDAI < 150	Decrease in CDAI by ≥70 points (70-point response) or ≥100 points (100-point response)	NA	IG1<br>8weekly<br>327 (226-473)<br>12weekly<br>304(21-486)<br><br>IG2<br>8weekly<br>289 (222–438)<br><br>12weekly<br>304(217-486)<br><br>CG<br>8weekly 320 (223-449)<br>12weekly<br>343(216-466)	NA	24
**Sandborn 2001b**	Etanercept 25mg subcutaneously twice weekly (n=23)	Placebo (n=20)	8weeks	8weeks	None	CDAI < 150	Decrease in CDAI by 70 or more points from baseline or a CDAI < 150	NA	**CDAI**<br>IG: 303 (226-499)<br><br>CG:265(115-453)<br><br>**IBDQ**<br>IG: 125 (72-173)<br><br>CG: 123 (45-169)	NA	4
**Sandborn 2005a (ENACT 1)**	Natalizumab (n-724)	Placebo (n=181)	8weeks	12weeks	Patients with a CDAI score of 0 to 220 at week 12 who had had a response at	CDAI < 150	Decrease in CDAI by 70 or more points from baseline	NA	IG: 302(60)<br>CG:303(65)	NA	10

					both weeks 10 and 12 without the need for intervention were eligible for the ENACT-2 trial. Eligible patients were randomly reassigned in a 1:1 ratio to receive an infusion of either placebo or 300 mg of natalizumab every four weeks from weeks 12 through 56 and were followed until week 60.						
**Sandborn 2007a**	Adalimumab, 160 mg subcutaneous injections at week 0 and 80 mg at week 2	Placebo (n=166)	2 weeks	4 weeks	None	CDAI < 150	Decrease in CDAI by ≥70 points (70-point response) or ≥100 points (100-point response)	NA	IG: 313 (58)<br>CG: 313(66)	NA	4

	(n=159)										
**Sandborn 2007c (PRECISE 1)**	Certolizumab Pegol 400mg subcutaneous at weeks 0, 2, and 4 and then every 4 weeks. (n=333)	Placebo (n=329)	26 weeks	26 weeks	None	CDAI < 150	Decrease in CDAI by ≥70 points (70-point response) or ≥100 points (100-point response)	NA	IG: 300(64)<br>CG: 297(62)	NA	26
**Sandborn 2008 (Population 1)**	Ustekinumab (n=51)	Placebo (n=53)	Patients were given subcutaneous placebo at weeks 0–3, then ustekinumab at weeks 8–11<br><br>subcutaneous ustekinumab at weeks 0–3, then placebo at weeks 8–11.<br><br>intravenous placebo at week 0, then	28weeks	None	CDAI>150	Decrease in CDAI by ≥70 points (70-point response) or ≥100 points (100-point response)	NA	Subcutaneous ustekinumab 90 mg→placebo 311(10)<br><br>Intravenous ustekinumab 4.5 mg/kg→placebo 325(66)<br><br>Placebo subcutaneous ustekinumab → 90 mg 292(40)<br><br>Placebo intravenous ustekinumab -→4.5 mg/kg 316(56)	NA	8

			ustekinumab at week 8; or<br><br>intravenous ustekinumab at week 0, then placebo at week 8								
**Sandborn 2011**	Certolizumab 400mg subcutaneous at weeks 0, 2, and 4. (n=223)	Placebo (n=216)	4 weeks	6 weeks	None	CDAI < 150	Decrease in CDAI by ≥100 points	NA	**CDAI**<br>IG: 262.1 (59.0)<br><br>CG: 292.7 (61.1)<br><br>**HBI**<br>IG: 9.8 (2.9)<br><br>CG: 9.7 (3.4)	NA	6
**Sandborn 2012 (CERTIFI)-Induction**	Ustekinumab doses of 1, 3, or 6 mg per kilogram of body weight or placebo (n=394)	Placebo (n=132)	Given at week 0	8weeks	None	CDAI < 150	Decrease in CDAI by ≥100 points	NA	IG: 327.7 (64.6)<br><br>CG: 312.4(64.2)	NA	6
**Sandborn 2012 (CERTIFI) non-responders**	Ustekinumab 90mg (n=109)	Placebo (n=110)	Given at weeks 8 and 16	22 weeks	Patients were followed through **36 weeks** for the safety analysis	CDAI < 150	Decrease in CDAI by ≥100 points	NA	IG: 323.5 ± 61.7<br>CG: 327.2 ±64.4	NA	22

**Sandborn 2013 (GEMNI II)- Induction**	Vedolizumab 300 mg intravenously at weeks 0 and 2 (n=220)	Placebo (n=148)	2 weeks	6 weeks	Patients from who had a clinical response (i.e., ≥70-point decrease in the CDAI score) with vedolizumab at week 6 were randomly assigned, in a 1:1:1 ratio, to continue in a blinded fashion to receive vedolizumab every 8 weeks, vedolizumab every 4 weeks, or placebo, for up to 52 weeks.<br><br>Patients who did not have a clinical response at week 6 to vedolizumab	CDAI < 150	Decrease in CDAI by ≥100 points	NA	IG: 323±68<br><br>CG: 35.7±11.9	NA		6

					induction therapy received vedolizumab at a dose of 300 mg every 4 weeks and were followed through week 52. Patients in the placebo group of cohort 1 continued to receive placebo and were also followed through week 52.						
**Sandborn 2014**	IG1: Oral tofacitinib 1 mg twice daily(n= 36)<br><br>IG2: Oral tofacitinib 5 mg twice daily(n= 34)	Placebo (n=34)	4weeks	4weeks	4-week follow-up period.	CDAI < 150	Decrease in CDAI by ≥70 points (70-point response) or ≥100 points (100-point response)	NA	**CDAI**<br>IG1: 300.3(76.2)<br><br>IG2: 297.7(63.7)<br><br>IG3: 308.0(50.8)<br><br>CG: 306.4(62.6)	NA	4

	IG3: Oral tofacitinib 15 mg twice daily(n= 35)										
**Sandborn 2020d (CELEST)-Induction**	IG1: Upadacitinib 3-mg twice daily (n=39)<br><br>IG2: Upadacitinib 6-mg, twice daily (n=37)<br><br>IG 3: Upadacitinib 12mg twice daily (n=36)<br><br>IG4: Upadacitinib 24-mg twice daily	Placebo (n=37)	16 weeks	16 weeks	All patients who completed the 16-week induction period were re-randomized 1:1:1 to receive double-blind maintenance therapy with the immediate-release formulation of upadacitinib at 3 mg twice daily, 12 mg twice daily, or 24 mg once daily for 36 weeks.	Daily Stool Frequency of =<1.5 and AP score of =<1.0, with neither worse than the baseline value	Decrease in CDAI from baseline by ≥70 points	SES-CD ≤ 4 and ≥ 2-point reduction from baseline and no subscore > 1	**CDAI**<br>Median (Range)<br>IG1: 288.0 (180–445)<br><br>IG2: 296.0 (230–599)<br><br>IG3: 280.0 (224–446)<br><br>IG4:277.5 (162–556)<br><br>IG5 305.0 (231–421)<br><br>CG: 276.0 (188–447)	**SES-CD**<br>Median (Range)<br>IG1 15 (4–37)<br><br>IG2 14(4-35)<br><br>IG3 12.5(4-38)<br><br>IG4 12 (5–29)<br><br>IG5 12.0 (4–31)<br><br>CG: 15.0 (4-37)	16

	(n=36)<br><br>IG5: Upadacitinib 24-mg once-daily (n=35)										
**Sandborn 2022 (GALAXI 1)**	IG 1: Guselkumab 200mg or 600mg or 1200mg intravenously at weeks 0, 4, and 8 (n=185)<br><br>IG: 2 Ustekinumab 6 mg/kg IV at week 0 and subcutaneous 90 mg at week 8 (n=63)	Placebo (n=61)	8 weeks	12weeks	None	CDAI < 150	Decrease in CDAI by ≥100 points	SES-CD ≤ 4 and ≥ 2-point reduction from baseline and no subscore > 1	IG1: 145.0 (42.37)<br><br>IG2: 147.2 (42.43)<br><br>CG: 300.8 (49.91)	**SES-CD**<br><br>IG1 12.2 (7.4)<br><br>IG2 15.1 (8.75)<br><br>CG: 12.8 (7.98	12
**Sandborn 2023-BERGAMONT**	Etrolizumab (n=241)	Placebo (n=59)				CDAI < 150	Decrease of at least 70 points on the CDAI	SES-CD ≤4 [≤2 for patients with ileal Crohn's disease only]	IG1: 326·3 (60·4)<br>IG2: 328·0 (61·0)<br>CG: 329·4 (64·0)	SES-CD<br>IG1: 14·36 (7·21)	14

**(cohort 1)**								with no segment having a subcategory score of >1		IG2: 13·12 (7·66)<br>CG: 13·32 (7·51)	
**Sandborn 2023-BERGAMONT (cohort 3)**	Etrolizumab (n=288)	PLA (n=97)	12 weeks	14weeks	Eligibility for entry into the maintenance phase was determined at week 14. Patients with a CDAI-70 response in the 105 mg and 205 mg etrolizumab groups in the induction phase were randomly assigned again in the maintenance phase to receive subcutaneous 105 mg etrolizumab (etrolizumab maintenance group) or placebo (placebo maintenance group) every	CDAI < 150	Decrease of at least 70 points on the CDAI	SES-CD ≤4 [≤2 for patients with ileal Crohn's disease only] with no segment having a subcategory score of >1	IG:1 326·3 (60·4)<br>IG2 328·0 (61·0)<br>CG: 329·4 (64·0)	IG1: 14·36 (7·21)<br>IG2 13·12 (7·66)<br>CG: 13·32 (7·51)	14

					4 weeks for 52 weeks.<br><br>Patients randomly assigned to placebo during induction who achieved CDAI-70 responses received blinded placebo during the maintenance phase after sham re-randomisation							
**Sands 2004 (ACCENT II) Induction (Non-responders)**	Infliximab + (intravenous, 5mg/kg maintenance) + Mercaptopurine or Azathioprine (unclear)	Placebo + Mercaptopurine or Azathioprine (unclear) (n=44)	From week 14 to week 22 (up to crossover)	54 weeks	NA	NA	Response in CDAI by at least 25 per and 70 points	NA	All participants:<br><br>CDAI Score ≥ 150: 56; Score ≥ 220: 30<br><br>IBDQ score: 161 (136-176) median (IQR)	NR	Unclear. Primary outcome was assessed at weeks 0, 14, 30 and 54

	(n=43)										
**Sands 2007**	Infliximab (intravenous, 5mg/kg, single dose in week 6) + Azathioprine (NR) (n=27) + Placebo	Natalizumab (intravenous,300 mg, every 4 weeks, total of 3 infusions) + Infliximab (intravenous, 5mg/kg, single dose in week 6) + Azathioprine (NR) (n=52)	8 weeks	13 months	Eligible patients joined a long-term extension study. Non-eligible patients had a clinical assessment 3 months after the last infusion and telephone contact 6 months after the last infusion.	CDAI < 150	Decrease in CDAI by ≥70 points	NA	IG: CDAI score <250: 17 (63); score ≥250: 10 (37)<br>CG: CDAI score <250: 27 (52); score ≥250: 25 (48) mean (SD	NR	10
**Sands 2010**	IG1: Apilimod 50mg, by mouth, daily (n=73) IG2: Apilimod 100mg, by	Placebo (n=73)	Induction period (days 1-43) and maintenance period (days 44-169)	53 weeks	NA	CDAI < 150	Decrease in CDAI by ≥100 points	NA	CDAI score:<br>IG1: 304 (61)<br>IG2: 300 (59)<br>CG: 305 (65)<br>mean (SD)	CDEIS score:<br>IG1: 10.8 (9)<br>IG2: 8 (7)<br>CG: 9.2 (7)<br>mean (SD)<br><br>SES-CD score:<br>IG1: 11.8 (9)<br>IG2: 10.8 (9)<br>CG: 11 (7)	6

	mouth, daily (n=74)										
**Sands 2014 (GEMINI III)**	Vedolizumab 300mg, intravenously at weeks 0, 2 and 6 (n=209)	Placebo. intravenously at weeks 0, 2 and 6 (n=207)	6 weeks	17 months	NA	CDAI < 150	Decrease in CDAI by ≥100 points	NA	CDAI score: IG: 300 (55.99) CG: 301.6 (61.58) mean (SD)	NR	6
**Sands 2022 (SEAVUE)**	Adalimumab 160mg, subcutaneous + placebo, intravenously on day 0 + adalimumab 80mg, subcutaneous at week 2, then adalimumab 40mg, subcutaneous every 2 weeks through week 56 (n=195)	Ustekinumab 6mg/kg, intravenously on day 0 + subcutaneous placebo day 0 (4 injections) and at 2 weeks (2 injections), then Ustekinumab 90mg, subcutaneous once every 8 weeks through week 56	56 weeks	18 months	NA	CDAI < 150	Decrease in CDAI by ≥100 points	SES-CD ≤2	CDAI score: IG: 301.3 (55) CG: 313.9 (53.2) mean (SD)	SES-CD score: IG: 9.8 (7.04) CG: 9.9 (6.94) mean (SD)	16

		+ placebo, subcutaneous, once every 2 weeks (n=191)									
**Sands 2022 (SERENITY)**	IG1: Mirikizumab 200mg, intravenously, every 4 weeks (n=31) IG2: Mirikizumab 600mg, intravenously, every 4 weeks (n=32) IG3: Mirikizumab 1000mg, intravenously, every 4 weeks (n=64)	CG: Placebo, intravenously, every 4 weeks (n=64)	12 weeks	34 months	NA	CDAI < 150	Decrease in CDAI Score by 100 points or more or a CDAI score <150	SES-CD score of <4 for ileal-colonic disease or <2 for isolated ileal disease, and no sub score	CDAI score: IG1: 348.3 (92.1) IG2: 298.2 (103.7) IG3: 304.5 (94.4) CG: 304.7 (93.1) mean (SD)	SES-CD score: IG1: 14.4 (7.9) IG2: 15.2 (7.4) IG3: 13.1 (6.8) CG: 11.9 (5.6) mean (SD)	12

**Schreiber 2005**	IG1: Certolizumab 100mg, subcutaneously, 3 injections, every 4 weeks (n=74)<br><br>IG2: Certolizumab 200mg, subcutaneously, 3 injections, every 4 weeks (n=72)<br><br>IG1: Certolizumab 400mg, subcutaneously, 3 injections, every 4 weeks (n=72)	Placebo (n=73)	8 weeks	13 months	NA	CDAI < 150	Decrease in CDAI score of ≥ 100	NA	CDAI index reported in terms of % reduction only	NR	12
**Schreiber 2018a**	IG1: Andecaliximab 150mg, every 2	Placebo, subcutaneous, once a	8 weeks	20 months	NA	CDAI < 150	NA	NA	CDAI score:<br>IG1: 320 (54.8)<br>IG2: 335 (61.4)<br>IG3: 329 (60.7)	SES-CD score:<br>IG1: 13 (6.3)<br>IG2: 16 (8.1)<br>IG3: 13 (8.8)	8

	weeks (n=53)<br>IG2: Andecaliximab 150mg, once a week (n=53)<br>IG3: Andecaliximab 300mg, once a week (n=53)	week (n=28)							CG: 298 (61.6)<br>mean (SD)	CG: 13 (7.7)<br>mean (SD)	
**Schroder 2006**	Infliximab 5mg/kg, intravenously (n= + Methotrexate 20mg, intravenously, 6 infusions at weeks 0-5 followed by Methotrexate 20mg, orally, for 28	Infliximab 5mg/kg, intravenously (n=8)	48 weeks	24 months	NA	CDAI < 150	NA	NA	CDAI score:<br>IG: 251 (61)<br>CG: 293 (93)<br>mean (SD)<br><br>IBDQ score:<br>IG: 113 (23)<br>CG: 106 (17)<br>mean (SD)	NR	14

	weeks (n=11)										
**SEQUENCE (Peyrin-Biroulet 2024)**	Ustekinumab, intravenously; ≤55kg: 260mg, >55kg to 85kg: 390mg, >85kg: 520mg + subcutaneous maintenance dose every 8 weeks (n=137) (sample size related to a subgroup comprising the first 50% of the patients to complete the week 24 visit)	Risankizumab 600mg, intravenously, as an induction dose at weeks 0, 4, and 8 + a 360mg subcutaneous maintenance dose every 8 weeks (n=128) (sample size related to a subgroup comprising the first 50% of the patients to complete the week 24 visit)	24 weeks	NR	NA	CDAI < 150	NA	SES-CD <= 4 and at least a 2-point reduction versus Baseline and no sub score greater than 1	Data related to the full cohort of patients (IG, n=265; CG, n=265)<br><br>CDAI score:<br>IG: 307.8 (260.8 – 347.9)<br>CG: 306 (265.9 – 344.8) median (IQR)	Data related to the full cohort of patients (IG, n=265; CG, n=265)<br><br>SES-CD score:<br>IG: 12 (8-19)<br>CG: 12 (8-18) median (IQR)	24

**Summers 1979**	Azathioprine 2.5mg/kg, daily (max dosage 250mg) (n=59)	CG1: Placebo (n=77)<br><br>CG2: 5-ASA, 1g/15kg, daily (max dosage 5g) (n=74)<br><br>CG3: Prednisone, daily dosage, based on CDAI score; <150: 1/4mg/kg, 150-300: 1/2mg/kg, >300:3/4 mg/kg(n =85)	17 weeks	17 weeks	NA	CDAI < 150	NA	NA	CDAI score:<br>IG: 240.7 (60.3)<br>CG1: 241.9 (72)<br>CG2: 256.2 (70.9)<br>CG3: 243.4 (74.6)<br>mean (SD)	NR	17
**Targan 1997**	IG1: Infliximab 5mg/kg, intravenously (n=27)	Placebo (n=25)	12 weeks	9 months	Patients who did not have a clinical response after the first infusion were given a	CDAI < 150	Decrease in CDAI by 70 or more points from baseline	NA	CDAI score:<br>IG1: 312 (56)<br>IG2: 318 (59)<br>IG3: 307 (50)<br>CG: 288 (54)<br>mean (SD)<br><br>IBDQ score:	NR	4

	IG2: Infliximab 10mg/kg, intravenously (n=28) IG3: Infliximab 20mg/kg, intravenously (n=28)				second infusion of open-label infliximab in a dose of 10 mg/kg and followed for an additional 12 weeks				IG1: 122 (29) IG2: 116 (23) IG3: 118 (28) CG: 128 (29) mean (SD)		
**Targan 2007 (ENCORE)**	Natalizumab 300mg, intravenously, in 3 infusions at weeks 0, 4 and 8 (n=259)	Placebo, intravenously, in 3 infusions at weeks 0, 4 and 8 (n=250)	8 weeks	12 months	NA	CDAI < 150	Decrease in CDAI by ≥70 points (70-point response) or ≥100 points (100-point response)	NA	IG: CDAI score <330: 174; ≥330: 84; missing: 1 CG: CDAI score <330: 178; ≥330: 1; missing: 1	NR	12
**Vermeire 2017 FITZROY induction**	Filgotinib 200mg, once a day (n=130)	Placebo, once a day (n=44)	10 weeks	17 months	NA	CDAI < 150	Decrease in CDAI by ≥100 points	SES-CD ≤4 and ulcerated surface subscore ≤ 1in all five segments	CDAI score: IG: 291.3 (53.8) CG: 298.6 (56.8) mean (SD)	SES-CD score: IG: 14.2 (6.8) CG: 15.8 (7.2) mean (SD)	10
**Vermeire 2017 FITZROY non-**	Filgotinib 200 mg once a day (n=25)	Placebo (n=9)	10 weeks	17 months	NA	CDAI>150	Decrease in CDAI by ≥100 points	SES-CD ≤4 and ulcerated surface subscore ≤ 1in all five segments	NR (Data available for the full cohort only)	NR (Data available for the full cohort only)	8

**responders**												
**Vermeire 2025 - DIVERSITY A**	IG1:Oral filgotinib 200 mg<br><br>IG2: Oral filgotinib 100 mg<br><br>All once daily for 11 weeks.	CG: Placebo once daily for 11 weeks	11 weeks	11 weeks	Randomised maintenance phase to week 58	CDAI<150	≥100-point reduction from baseline in CDAI or CDAI <150	SES-CD score of ≤2	CDAI<br><br>IG1: 323(55.6)<br>IG2: 322(55.5)<br>CG: 320(59.4)	SES-CD<br><br>IG1:13(7.1)<br>IG2: 14(7.9)<br>CG: 13(7.2)		10
**Vermeire 2025 - DIVERSITY B**	IG1:Oral filgotinib 200 mg<br><br>IG2: Oral filgotinib 100 mg<br><br>All once daily for 11 weeks.	CG: Placebo once daily for 11 weeks	11 weeks	11 weeks	Randomised maintenance phase to week 58	CDAI<150	≥100-point reduction from baseline in CDAI or CDAI <150	SES-CD score of ≤2	CDAI<br><br>IG1: 306(54.0)<br>IG2: 321(55.7)<br>CG: 322(57.5)	SES-CD<br><br>IG1:15(7.9)<br>IG2: 15(8.2)<br>CG: 15(7.8)		10
**Watanabe 2012 - Induction**	IG1: Adalimumab 80/40mg at baseline and week 2 (n=34)	Placebo at baseline and week 2 (n=23)	2 weeks	52 weeks	NA	CDAI < 150	Decrease in CDAI by ≥70 points (70-point response) or ≥100 points (100-point response)	NA	CDAI score:<br>IG1: 302.7 (66.6)<br>IG2: 300.5 (66.5)<br>CG: 308.1 (63.8)<br>mean (SD)<br><br>IBDQ score:<br>IG1: 148.6 (27.9)<br>IG2: 145.9 (25.2)	NR		4

	IG2: Adalimumab 160/80mg at baseline and week 2 (n=33)								CG: 139.4 (26.8) mean (SD)		
**Watanabe 2020 - Induction**	Vedolizumab 300mg, intravenously at weeks 0, 2 and 6 (n=79)	Placebo, intravenously at weeks 0, 2 and 6 (n=78)	Infusions given at weeks 0, 2 and 6)	10 weeks	NA	CDAI < 150	Decrease in CDAI by ≥100 points	NA	CDAI score: IG: 303.9 (63.2) CG: 295 (64.8) mean (SD)	NR	10
**Winter 2004**	IG1: Certolizumab 1.25mg/kg*, intravenously, single infusion (n=2) IG2: Certolizumab 5mg/kg, intravenously, single infusion (n=25)	Placebo, intravenously, single infusion (n=25)	Single infusion	8 months	NA	CDAI < 150	Decrease in CDAI score by ≥ 100 points	NA	All participants: mean CDAI ranging from 220-450; separate scores per group reported visually only	NR	4

	IG3: Certolizumab 10mg/kg, intravenously, single infusion (n=17) IG4: Certolizumab 20mg/kg, intravenously, single infusion (n=23)<br><br>*replaced with a dose of 10mg/kg due to low efficacy										
**Ye 2019**	Infliximab 5 mg/kg, intravenously + Azathioprine (dosage NR) (n=109)	CTP13 5 mg/kg, intravenously + Azathioprine (dosage NR) (n=111)	6 weeks	54 weeks	NA	CDAI < 150	Decrease in CDAI by ≥70 points (70-point response) or ≥100 points (100-point response)	NA	CDAI score:<br>IG: 295.7 (55.46)<br>CG: 296.3 (54.3)<br>mean SD<br><br>SIBDQ score:<br>IG: 33.9 (9.27)<br>CG: 34.3 (10.92)<br>mean (SD)	SES-CD<br>IG: 9.6 (7.92)<br>CG: 9.8 (7.83)	6

eTable 3. Outcomes reported in the included studies.

Study ID (author, Year)	Clinical remission	Clinical response	Endoscopic remission	Withdrawals due to adverse events	Serious adverse events	Total adverse events
**Allez 2023 (TRIDENT Part 1)**	Pl: 7/72<br>Tes: 15/73	Pl: 11/72<br>Tes: 21/73	Pl: 5/72<br>Tes: 10/73	Pl: 14/72<br>Tes: 25/73	Pl: 1/72<br>Tes: 8/73	Pl: 24/72<br>Tes: 45/73
**Allez 2023 (TRIDENT Part 2)**	Pl: 7/48<br>Tes: 35/148<br>Ust: 25/47	Pl: 11/48<br>Tes: 56/148<br>Ust: 34/47	Pl: 6/48<br>Tes: 6/148<br>Ust: not reported.	Pl: 8/48<br>Tes: 31/148<br>Ust: 5/47	Pl: 3/48<br>Tes: 9/148<br>Ust: 2/47	Pl: 19/48<br>Tes: 88/148<br>Ust: 22/47
**Ardizzone 2003**	Aza:17/27<br>Meth:15/27	NR	NR	Aza: 3/27<br>Meth: 3/27	NR	Aza:7/27<br>Meth:17/27
**Arora 1999**	Pl:16/18<br>Meth: 10/15	NR	NR	Pl: 0/18<br>Meth: 3/15	NR	NR
**Candy 1995**	Aza: 24/33<br>Pl: 19/30	NR	NR	Aza: 0/33<br>Pl: 2/30	NR	NR
**Chen 2020**	AdaAza: 38/102<br>Aza:7/103	AdaAza: 69/102<br>Aza: 28/103	NR	AdaAza: 3/102<br>Aza: 5/103	AdaAza: 1/102<br>Aza: 2/103	AdaAza: 38/102<br>Aza: 38/103
**Chen 2025 (NCT03234907)**	Ved: 13/144<br>Pl: 8/71	Ved: 28/144<br>Pl: 17/71	NR	Ved: 5/144<br>Pl: 7/71	Ved: 12/144<br>Pl: 5/71	Ved: 72/144<br>Pl: 26/71
**Colombel 2007 (charm)-induction (non-responders)**	NR	NR	NR	NR	NR	NR

**Colombel 2010 (SONIC)**	Inf: 81/169<br><br>InfAza: 102/169<br>Aza: 54/170	Inf<br>95/169 (CDAI70)<br>92/169 (CDAI100)<br><br>InfAza: 113/169 (CDAI70)<br>105/169 (CDAI100)<br><br>Aza<br>71/170(CDAI70)<br>64/170(CDAI100)	NR	Inf: 37/169<br><br>InfAza: 29/169<br>Aza: 43/170	Inf: 39/169<br><br>Inflaza: 27/169<br><br>Aza: 43/170	Inf: 53/169<br><br>Inflaza: 53/169<br><br>Aza: 69/170
**D'Haens 1999**	NR	NR	NR	NR	NR	NR
**D'Haens 2008**	InfAza: 43/67<br>Str: 21/66	NR	NR	InfAza: 14/67<br>Str: 14/66	Infaza: 20/67<br>Str: 19/66	InfAza: 43/67<br>Str: 21/66
**D'Haens 2022 (ADVANCE)**	Ris: 293/675<br><br>Pl: 44/186	Ris: 420/675<br><br>Pl: 64/186	Ris: 162/675<br><br>Pl: 16/186	Ris: 23/675<br><br>Pl: 19/186	Ris: 40/675<br><br>Pl: 18/186	Ris: 401/675<br><br>Pl: 105/186
**D'Haens 2022 (MOTIVATE)**	Ris: 158/382<br><br>Pl: 37/187	Ris: 229/382<br><br>Pl: 56/187	Ris: 76/382<br><br>Pl: 8/187	Ris: 9/382<br><br>Pl: 19/187	Ris: 19/382<br><br>Pl: 25/187	Ris: 219/382<br><br>Pl: 137/187
**Duan 2013**	Inf: 4/8<br><br>InfAza: 5/8<br><br>Aza: 3/8	Inf: 2/8<br><br>InfAza: 2/8<br><br>Aza: 2/8	NR	Inf: 1/8<br><br>InfAza: 0/8<br><br>Aza: 1/8	NR	NR
**Ewe 1993**	Aza: 16/21<br><br>Pl: 8/21	NR	NR	Aza: 1/21<br><br>Pl: 0/21	NR	NR
**Feagan 1995**	Meth: 37/94<br><br>Pl: 9/47	NR	NR	Meth: 16/94<br><br>Pl: 1/47	NR	NR
**Feagan 2008c**	Pl: 12/58<br><br>Ved: 43/125	PL<br>24/58 (CDAI70)<br>18/58 (CDAI100)	NR	Pl: 0/58<br><br>Ved: 2/127	Ved: 54/127<br><br>Pl: 29/58	Ved: 117/127<br><br>Pl: 50/58

		Ved<br>64/127 (CDAI70) 58/127 (CDAI100)				
**Feagan 2014**	InfMeth:<br>48/63<br>Inf: 49/63	NOT REPORTED	NR	InfMeth: 2/63<br>Inf: 1/63		NR
**Feagan 2015a (UNITI-1)**	Ust: 91/494<br><br>Pl: 18/247	Pl<br>75/247(CDAI70)<br>53/247(CDAI100)<br><br>UST<br>222/494(CDAI70)<br>168/494(CDAI100)	NR	Ust: 23/494<br><br>Pl: 10/247	Ust: 30/494<br><br>Pl: 15/247	Ust: 323/494<br><br>Pl: 159/247
**Feagan 2015b (UNITI-2)**	Ust: 148/418<br><br>Pl: 41/210	Pl<br>81/210(CDAI70)<br>60/210(CDAI100)<br><br>Ust<br>258/418(CDAI70)<br>224/418(CDAI100)	NR	Ust: 11/418<br><br>Pl: 12/210	Ust: 16/418<br><br>Pl: 12/210	Ust: 221/418<br><br>Pl: 113/210
**Feagan 2017**	Ris: 25/82<br><br>Pl: 6/39	Ris: 64/82<br><br>Pl: 8/39	Ris: 14/82<br><br>Pl: 1/39	Ris: 7/82<br><br>Pl: 6/39	Ris: 12/82<br><br>Pl: 12/39	Ris: 63/82<br><br>Pl: 32/39
**Ferrante 2024 (VIVID-1)**	Miri: 218/631<br><br>Uste: NR/309<br><br>Placebo: 50/212	Miri: 409/631<br><br>Uste: NR/309<br><br>Placebo: 103/212	Miri: 63/631<br><br>Uste: NR/309<br><br>Placebo: 8/212	Miri: 5/579<br><br>Uste: 7/287<br><br>Placebo: 3/199	Miri: 65/630<br><br>Uste: 33/309<br><br>Placebo: 36/211	Miri: NR<br><br>Uste: NR<br><br>Placebo: NR
**Ghosh 2003**	Nat: 65/185<br><br>Pl:17/63	Nat:116/185<br><br>Pl: 24/63	NR	Nat: 10/185<br><br>Pl: 7/63	Nat: 10/185<br><br>Pl: 7/63	Nat: 147/185<br><br>Pl: 51/63

**Gordon 2001**	Nat: 7/18<br><br>Pl: 1/12	NR	NR	Nat: 1/18<br><br>Pl: 2/12	NR	NR
**Hanauer 2002-ACCENT I (non-responders)**	NR	NR	NR	NR	NR	NR
**Hanauer 2006 (CLASSIC I)**	Ada: 78/224<br><br>Pl: 12/74	Ada<br>172/224(CDAI70)<br>124/224(CDAI100)<br><br>Pl<br>37/74(CDAI70)<br>25/74(CDAI100)	NR	Ada: 5/224<br><br>Pl: 5/74	Ada: 4/224<br><br>Pl: 3/74	Ada: 158/224<br><br>Pl: 55/74
**Hanauer 2021 (VOLTAIRE-CD)**	Ada: 54/75<br><br>Bi: 46/72	Ada: 55/75<br><br>Bi: 59/72	NR	Ada:16/75<br><br>Bi: 15/72	Ada:8/75<br><br>Bi: 6/72	Ada: 42/75<br><br>Bi: 45/72
**Hart 2025 (GRAVITI)**	Combined gus: 129/232<br>Placebo: 25/118	Combined gus: 169/232<br>Placebo: 39/118	Combined gus: 66/232<br>Placebo: 17/118	Combined gus: 3/232<br>Placebo: 12/118	Combined gus: 24/230<br>Placebo: 16/117	Combined gus: 187/230<br>Placebo: 77/117
**Jairath 2025 (RELIEVE UCCD)**	NR	NR	NR	IG1: 31/46<br>IG2: 20/46<br>CG: 22/46	NR	IG1: 4/46<br>IG2: 1/46<br>CG: 1/46
**Klein 1974**	NR	Aza: 6/13<br>Pl: 6/13	NR	Aza: 2/13<br>Pl: 0/13	NR	NR
**Lemann 2006**	Aza: 17/58<br><br>InfAza: 32/57	NR	NR	Aza: 5/58<br><br>InfAza:2/57	Aza: 3/58<br><br>InfAza:3/57	Aza: 28/58<br><br>InfAza:29/57
**Loftus 2023 U-EXCEL**	Upa: 173/350<br><br>Pl: 51/176	Upa: 198/350<br><br>Pl: 65/176	Upa: 101/350<br><br>Pl: 13/176	Upa: 18/350<br><br>Pl: 12/176	Upa: 24/350<br><br>Pl: 12/176	Upa: 219/350<br><br>Pl: 103/176

**Loftus 2023 U-EXCEED**	Upa: 126/324<br><br>Pl: 36/171	Upa: 163/324<br><br>Pl: 47/171	Upa: 62/324<br><br>Pl: 4/171	Upa: 28/324<br>Pl: 14/171	Upa: 30/324<br>Pl: 17/171	Upa: 221/324<br>Pl: 112/171
**Mantzaris 2004**	InfAza: 19/27<br><br>Inf:14/23	NR	NR	InfAza: 3/27<br><br>Inf: 1/23	InfAza: 3/27<br>Inf:1/23	InfAza: 15/27<br>Inf: 6/23
**Mate-jimenez 2000**	Aza:15/16<br><br>Meth: 12/15<br><br>5asa: 1/7	Not reported	NR	Aza: 1/16<br><br>Meth: 2/15<br><br>5asa: 0/7	NR	Aza: 1/16<br><br>Meth: 2/15<br><br>5asa: 0/7
**Matsumoto 2016 - DIAMOND**	AdaAza: 62/92<br><br>Ada: 61/85	AdaAza: 55/92<br><br>Ada: 64/85	AdaAza: 57/92<br><br>Ada: 58/85	Adaaza: 22/92<br><br>Ada: 19/85	NR	AdaAza: 3/92<br><br>Ada: 21/85
**Oren 1997**	Aza: 9/32<br><br>Meth: 7/26<br><br>Pl: 6/26	Not reported	NR	Aza: 1/32<br><br>Meth: 1/26<br><br>Pl: 0/26	NR	NR
**Panaccione 2024 (GALAXI-2)**	Combined guselkumab: 136/297<br>Placebo: 17/77<br>Ustekinumab NR	NR	NR	Pooled GALAXI 2/3<br><br>Gus group 1: 31/296<br>Gus group 2: 38/286<br>Ustekinumab: 47/291<br>Placebo: 27/148	Gus group 1: 6/148<br>Gus group 2: 19/148<br>Ustekinumab: 18/150<br>Placebo: 6/77	Gus group 1: 86/148<br>Gus group 2: 85/148<br>Ustekinumab: 96/150<br>Placebo: 27/77
**Panaccione 2024 (GALAXI-3)**	Combined guselkumab: 138/299	NR	NR	Pooled GALAXI 2/3<br><br>Gus group 1: 31/296	Gus group 1: 15/151<br>Gus group 2: 13/148<br>Ustekinumab: 17/150	Gus group 1: 95/151<br>Gus group 2: 82/148<br>Ustekinumab: 90/150

	Placebo: 11/76<br>Ustekinumab: NR			Gus group 2: 38/286<br>Ustekinumab: 47/291<br>Placebo: 27/148	Placebo: 10/76	Placebo: 40/76
**Panes 2017 Induction**	Tof: 81/188)<br><br>Pl: 31/33	Tof:<br>142/188 (CDAI70)<br>131/188 (CDAI100)<br><br>Pl:<br>56/92 (CDAI70)<br>49/92 (CDAI100)	NR	Tof: 21/188<br><br>Pl: 15/92	Tof: 26/188<br><br>Pl: 11/92	Tof: 111/188<br><br>Pi: 55/92
**Present 1980**	NR	Aza: 26/36<br><br>Pl: 5/36<br>N	NR	Aza: 10/56<br><br>Pl: 3/36	NR	NR
**Present 1999**	Inf: 30/63<br><br>Placebo: 4/31	Inf: 39/63<br><br>Placebo: 8/31	NR	Inf: 2/63<br>Placebo: 0/31	Inf: 4/63<br>Placebo: 1/31	Inf: 36/63<br>Placebo: 12/31
**Reinisch 2008**	Aza:25/52<br><br>Pl:15/29<br><br>Ever:31/63	NR	NR	Aza: 8/52<br><br>Pl: 4/29<br><br>Ever: 29/63	Aza: 11/52<br><br>Pl: 5/29<br><br>Ever: 12/63	Aza: 36/52<br><br>Pl: 24/29<br><br>Ever: 49/63
**Rhodes 1971**	NR	Aza: 0/9<br><br>Pl: 0/7	NR	Aza: 1/9<br><br>Pl: 0/7	NR	NR
**Rutgeerts 2006**	Oner: 40/169<br><br>Pl: 9/38	Oner: 67/169<br><br>Pl: 14/38	NR	Oner: 7/169<br><br>Pl: 3/38	Oner: 5/169<br><br>Pl: 1/38	Oner: 121/169<br><br>Pl: 26/38
**Rutgeerts 2012 (EXTEND)**	NR	NR	Ada: 33/64<br><br>Pl: 18/65	NR	NR	NR
**Sandborn 2001a**	Hum: 12/111<br><br>Placebo 2/58	Hum: 33/111<br><br>Pl: 8/58	NR	Hum: 62/111<br>Pl: 40/58	NR	NR

**Sandborn 2001b**	Eta: 2/23<br>Pl: 4/20	Eta: 9/23<br>Pl: 9/20	NR	Eta: 14/23<br>Pl: 9/20	Eta: 2/23<br>Pl: 5/20	Eta: 17/23<br>Pl: 10/20
**Sandborn 2005a (ENACT 1)**	Nat: 267/724<br>Pla: 55/181	Nat: 408/724<br>Pl: 88/181	NR	Nat: 59/724<br>Pl: 13/181	Nat: 52/724<br>Pl: 12/181	Nat: 626/724<br>Pl: 153/181
**Sandborn 2007a**	AdaAza: 34/159<br>Aza: 12/166	AdaAza: 61/159<br>Aza:41/166	NR	AdaAza: 3/159<br>Aza: 5/166	AdaAza: 2/159<br>Aza: 8/166	AdaAza: 91/159<br>Aza: 121/166
**Sandborn 2007c (PRECISE 1)**	Czp: 47/333<br>Pl: 32/329	Czp: 75/333<br>Pl: 52/329	NR	Czp:126/333<br>Pl: 151/329	Czp: 34/333<br>Pl: 23/329	Czp: 269/333<br>Pl: 260/329
**Sandborn 2008 (Population 1)**	Ust: 13/51<br>Pl: 9/53	Ust: 25/51<br>Pl: 15/53	NR	Ust: 5/51<br>Pl: 2/53	Ust: 2/51<br>Pl: 3/53	Ust: 37/51<br>Pl: 41/53
**Sandborn 2011**	Czp:<br>63/223<br>Pl: 53/216	Czp:<br>87/223<br>Pl: 71/216	NR	Czp:<br>16/223<br>Pl: 24/216	Czp:<br>12/223<br>Pl: 8/216	Czp:<br>114/223<br>Pl: 100/216
**Sandborn 2012 (CERTIFI)-induction**	Ust: 58/394<br>Pl: 14/132	Czp:<br>76/394<br>Pl: 38/132	NR	Ust: 30/394<br>Pl:19/132	Ust: 23/394<br>Pl:11/132	Ust: 257/394<br>Pl: 94/132
**Sandborn 2012 (CERTIFI) non-responders**	Ust: 12/109<br>Pl: 7/110	Ust: 22/109<br>Pl: 20/110	NR	Ust: 17/109<br>Pl: 22/110	Ust: 22/109<br>Pl: 21/110	Ust: 86/109<br>Pl: 89/110
**Sandborn 2013 (GEMNI II)-induction**	Ved: 33/ 220<br>Pl: 10/148	Ved: 69/ 220<br>Pl: 38/148	NR	Ved: 21/220<br>PL: 11/148	Ved: 20/220<br>PL: 9/148	Ved: 124/220<br>PL: 88/148
**Sandborn 2014**	Tof: 24/105	Tof: 39/ 105	NR	Tof: 9/ 105	Tof: 9/ 105	Tof: 61/ 105

	Pl: 7/34	Pl: 10/34		Pl: 8/34	Pl: 5/34	Pl: 22/34
**Sandborn 2020d (CELEST)-induction**	Upa: 32/183<br><br>Pl: 4/37	Upa: 51/183<br><br>Pl: 2/37	Upa: 23/183<br><br>Pl: 0/37	Upa: 28/183<br><br>Pl: 9/37	Upa: 27/183<br><br>Pl: 2/37	Upa: 151/183<br><br>Pl: 27/37
**Sandborn 2022 (GALAXI 1)**	Gus: 98/217<br><br>Ust: 29/71<br><br>Pl: 10/70	Gus: 122/217<br><br>Ust: 42/71<br><br>Pl: 15/70	Gus: 21/217<br><br>Ust: 7/71<br><br>Pl: 6/70	Gus: 80/217<br><br>Ust: 8/71<br><br>PL: 11/70	Gus: 8/217<br><br>Ust: 4/71<br><br>PL: 4/70	Gus: 100/217<br><br>Ust: 36/71<br><br>PL: 42/70
**Sandborn 2023-BERGAMONT (cohort 1)**	Etr: 57/241<br><br>Pl: 6/59	NR	NR	NR	NR	NR
**Sandborn 2023-BERGAMONT (cohort 3)**	Etr: 91/288<br><br>Pla: 28/97	NR	Etr: 37/288<br><br>Pla: 8/97	Etr: 22/288<br><br>Pla: 12/97	Etr:20/ 288<br><br>Pl: 8/97	Etr: 180/288<br><br>Pl: 51/97
**Sands 2004 (accent ii) induction (non-responders)**	NR	Aza :7/44<br><br>Infaza: 9/43	NR	NR	NR	NR
**Sands 2007**	InfAza: 8/27<br><br>NatinfAza: 19/52	NR	NR	InfAza: 4/27<br><br>NatinfAza: 7/52	InfAza: 1/27<br><br>NatinfAza: 1/52	InfAza: 27/27<br><br>NatinfAza: 48/52
**Sands 2010**	Am: 19/147<br><br>Pl: 14/73	Am: 26/147<br><br>Pl: 22/73	NR	Am: 32/147<br><br>Pl: 19/73	Am: 10/147<br><br>Pl: 8/73	Am: 112/147<br><br>Pl: 59/73
**Sands 2014 (GEMINI III)**	Ved: 40/209<br><br>Pl: 25/207	Ved: 82/209<br><br>Pl: 47/207	NR	Ved: 13/209<br><br>Pl: 15/207	Ved: 13/209<br><br>Pl: 16/207	Ved: 117/209<br><br>Pl: 124/207
**Sands 2022 (SEAVUE)**	Ada: 117/195	Ada: 129/195	Ada: 55/195	Ada: 22/195	Ada: 32/195	Ada: 152/195

	Ust: 109/191	Ust: 138/191	Ust: 51/191	Ust: 12/191	Ust: 25/191	Ust: 153/191
**Sands 2022 (SERENITY)**	Miri: 35/127<br><br>Pl: 6/64	Miri: 60/127<br><br>Pl: 15/64	Miri: 20/127<br><br>Pl: 1/64	Miri: 4/127<br><br>Pl: 4/64	Miri: 5/127<br><br>Pl: 7/64	Miri: 81/127<br><br>Pl: 45/64
**Schreiber 2005**	Czp: 53/219<br><br>Pl: 17/73	Czp: 85/219<br><br>Pl: 26/73	NR	Czp: 100/219<br><br>Pl: 32/73	Czp: 23/219<br><br>Pl: 6/73	Czp: 160/219<br><br>Pl: 51/73
**Schreiber 2018a**	And: 26/159<br><br>Placebo: 6/28	And: 21/159<br><br>Placebo: 4/28	NR	And: 12/159<br><br>Placebo: 1/28	And: 15/159<br><br>Placebo: 3/28	And: 102/159<br><br>Placebo: 19/28
**Schroder 2006**	InfMeth 6/11<br><br>Inf: 3/8	NR	NR	InfMeth 0/11<br><br>Inf: 0/8	NR	NR
**Sequence**	Ust: 54/137<br><br>Ris: 75/128	NR	Ust: 43/265<br><br>Ris: 81/255	Ust: 37/137<br><br>Ris: 20/128	Ust: 37/137<br><br>Ris: 20/128	Ust: 51/137<br><br>Ris: 42/128
**Summers 1979**	Aza: 21/59<br><br>Pl: 20/77<br><br>5-asa: 28/74<br><br>Pred: 40/85	NR	NR	Aza: 6/59<br><br>Pl: 4/77<br><br>5-asa: 0/74<br><br>Pred: NR	Aza: 4/59<br><br>Pl: 1/77<br><br>5-asa: 0/74<br><br>Pred: NR	Aza: 6/59<br><br>Pl: 4/77<br><br>5-asa: 0/74<br><br>Pred: NR
**Targan 1997**	Inf: 41/83<br><br>Pl: 2/25	Inf: 54/83<br><br>Pl: 4/25	NR	NR	NR	NR
**Targan 2007 (ENCORE)**	Nat: 98/259<br><br>Pl: 62/250	Nat: 127/259<br><br>Pl: 77/250	NR	Nat: 36/259<br><br>Pl: 42/250	Nat: 13/259<br><br>Pl: 24/250	Nat: 222/259<br><br>Pl: 206/250
**Vermeire 2017 FITZROY induction**	Fil: 60/130<br><br>Pl: 10/44	Fil: 76/130<br><br>Pl: 18/44	Fil: 18/130<br><br>Pl: 3/44	Fil: 31/152<br><br>Pl: 7/44	Fil:33/152<br>PL: 8/44	Fil:128/152<br>PL: 31/44

**Vermeire 2017 FITZROY non-responders**	NR	NR	NR	NR	NR	NR
**Vermeire 2025 – DIVERSITY A**	Filgo200: 73/223<br>Filgo100: 63/245<br>Placebo: 47/239	Filgo200: 116/223<br>Filgo100: 114/245<br>Placebo: 94/239	Filgo200: 24/223<br>Filgo100: NR<br>Placebo: 19/239	Filgo200: 13/222<br>Filgo100: 19/245<br>Placebo: 18/237	Filgo200: 18/222<br>Filgo100: 16/245<br>Placebo: 15/237	Filgo200: 114/222<br>Filgo100: 136/245<br>Placebo: 137/237
**Vermeire 2025 – DIVERSITY B**	Filgo200: 54/204<br>Filgo100: 38/230<br>Placebo: 34/231	Filgo200: 78/204<br>Filgo100: 81/230<br>Placebo: 63/231	Filgo200: 4/204<br>Filgo100: NR<br>Placebo: 11/231	Filgo200: 22/202<br>Filgo100: 35/228<br>Placebo: 28/229	Filgo200: 19/202<br>Filgo100: 36/228<br>Placebo: 26/229	Filgo200: 141/202<br>Filgo100: 254/228<br>Placebo: 156/229
**Watanabe 2012 - Induction**	Ada:17/67<br><br>Pl: 3/23	Ada: 32/67<br><br>Pl: 4/23	NR	Ada: 3/67<br><br>Pl: 1/23	Ada: 3/67<br><br>Pl: 1/23	Ada: 37/67<br><br>Pl: 12/23
**Watanabe 2020 - Induction**	Ved: 14/79<br><br>Pl: 8/78	Ved: 21/79<br><br>Pl: 13/78	NR	Ved: 3/79<br><br>Pl: 12/78	Ved: 8/79<br><br>Pl: 10/78	Ved: 49/79<br><br>Pl: 42/78
**Willoughby 1971**	NR	Aza:6/6<br><br>Pl: 1/6	NR	Aza:0/6<br><br>Pl: 0/6	NR	NR
**Winter 2004**	Czp: 18/67<br><br>Pl: 10/25	Czp: 36/67<br><br>Pl: 14/25	NR	Czp: 31/67<br><br>Pl: 5/25	Czp: 7/67<br><br>Pl: 2/25	Czp: 43/67<br><br>Pl: 15/25
**Ye 2019**	InfAza: 49/109<br><br>Ctp13Aza: 47/111	InfAza<br>77/109 (CDAI70)<br>67/109 (CDAI100)<br><br>Ctp13Aza<br>81/111 (CDAI70)<br>70/111 (CDAI100)	NR	InfAza: 21/109<br><br>Ctp13Aza: 17/111	InfAza: 9/109<br><br>Ctp13Aza: 6/111	InfAza: 70/109<br><br>Ctp13Aza: 63/111

eTable 4. Excluded studies and reasons for exclusion.

**D'Haens 2022 -SERENE**	No control group (compared two dosages of adalimumab)
**JPRN-UMIN000023735**	Wrong intervention (assessing treatment strategy)
**JPRN-UMIN000024566**	Wrong intervention (dose escalation trial)
**Klein 1974**	Not properly randomised
**Moroi 2024**	Phase 2a Ustekinumab with Budesonide vs Ustekinumab
**NCT03599622 (LATTICE-CD)**	Phase 2a (terminated due to lack of efficacy)
**NCT04245215 (REScUE)**	No control group (compared two dosages of ustekinumab)
**NCT05688852 (Harmony-CD)**	Phase 2a (terminated – sponsor decision)
**NCT04835506**	Wrong intervention (trough levels)
**NCT02646683 (LOVE-CD)**	Not RCT
**NCT03559517 (CARMEN CD 305)**	Early termination (sponsor decision)
**NCT03566823 (CARMEN CD 306)**	Early termination (sponsor decision)
**NCT06227910 (VICTRIVA)**	Ongoing
**Rhodes 1971**	Not properly randomised
**Present 1980**	Not properly randomised
**Reinisch 2009 (CARE)**	Wrong study type
**Reinisch 2024 (DIVERGENCE 2 - NCT03077412)**	Phase 2a for fistula treatment
**Sandborn 2008 (Population 2)**	No control group (compared two dosages of adalimumab)
**Sandborn 2010 (WELCOME)**	No control group (compared two dosages of certolizumab)

**Sandborn 2022 - BERGAMOT Cohort 2**	No control group (compared two dosages of etrolizumab)
**Schreiber 2023 -POWER**	No control group (compared two dosages of ustekinumab+placebo)
**Schulberg 2022 (STRIDENT)**	Wrong population (treatment of strictures)
**Schwartz 2021 - ENTERPRISE**	No control group (compared two dosages of vedolizumab)
**Willoughby 1971**	Not properly randomised
**Wu 2016**	No control group (compared two routes of ustekinumab)

eTable 5. Predefined Magnitude Effect Thresholds

	Trivial to Small	Small to Moderate	Moderate to Large
**Clinical Remission**	10%	20%	31%
**Clinical Response**	13%	23%	35%
**Endoscopic relapse**	9%	17%	28%
**Withdrawals due to adverse events**	7%	14%	23%
**Serious Adverse Events**	6%	11%	17%
**Total Adverse Events**	9%	16%	24%

eTable 6. SUMMARY OF FINDINGS TABLES AND GRADE DECISIONS (red colouring denotes treatment crosses the line of no effect)

Clinical remission							
Patient or population: people with active Crohn's disease							
Settings: hospital setting							
Intervention: biologics/purine analogues/methotrexate							
Comparison: placebo							
**Treatment**	**Network evidence**		**Anticipated absolute effects for network estimate**			**NNT (95% CI)**	**Notes**
	**RR (95% CI)**	**Certainty**	**Risk with Placebo**[a]	**Risk with Agent**[b]	**% Risk Difference with Agent**[c]		
Adalimumab and purine analogues	2.87 (1.99 to 4.14)	Moderate ⊕⊕⊕⊖	189 per 1,000	542 per 1,000 (376 to 782)	35.3% more (18.7% more to 59.3% more)	3 (2 to 5)	It is probably better than placebo by a large effect size (ranging from small to large)
Natalizumab with Infliximab and purine analogues	2.99 (1.32 to 6.81)	Very low ⊕⊖⊖⊖	189 per 1,000	565 per 1,000 (249 to 1,000)	37.6% more (6% more to 100% more)	NA	The evidence is very uncertain
Guselkumab	2.5 (1.95 to 3.21)	Moderate ⊕⊕⊕⊖	189 per 1,000	473 per 1,000 (369 to 607)	28.4% more (18% more to 41.8% more)	4 (2 to 6)	It's probably better than placebo by a moderate effect size (ranging from small to large)
Adalimumab	2.46 (1.84 to 3.29)	Moderate	189 per 1,000			4 (2 to 6)	

		⊕⊕⊕⊖		465 per 1,000 (348 to 622)	27.6% more (15.9% more to 43.3% more)		It's probably better than placebo by a moderate effect size (ranging from small to large)
Humicade	3.14 (0.7 to 13.94)	Very low<br>⊕⊖⊖⊖	189 per 1,000	593 per 1,000 (132 to 1000)	40.4% more (5.7% less to 100% more)	NA	The evidence is very uncertain
Infliximab with purine analogues	2.43 (1.71 to 3.44)	Moderate<br>⊕⊕⊕⊖	189 per 1,000	459 per 1,000 (323 to 650)	27% more (13.4% more to 46.1% more)	4 (2 to 7)	It's probably better than placebo by a moderate effect size (ranging from small to large)
Risankizumab	2.26 (1.79 to 2.86)	Very low<br>⊕⊖⊖⊖	189 per 1,000	427 per 1,000 (338 to 541)	23.8% more (14.9% more to 35.2% more)	NA	The evidence is very uncertain
CTP13 with purine analogues	2.29 (1.32 to 3.95)	Low<br>⊕⊕⊖⊖	189 per 1,000	433 per 1,000 (249 to 747)	24.4% more (6% to 55.8% more)	NA	It may be better than placebo by a moderate effect size (ranging from trivial to large)
BI695501	2.18 (1.36 to 3.49)	Low<br>⊕⊕⊖⊖	189 per 1,000	412 (257 to 660)	22.3% more (6.8% more 47.1% more)	NA	It may be better than placebo by a moderate effect size (ranging from trivial to large)
MethotrexateInfliximab	2.13 (1.3 to 3.48)	Very low	189 per 1,000	403 per 1,000 (246 to 658)		NA	

		⊕⊖⊖⊖			21.4% more (5.7% more to 46.9% more)		The evidence is very uncertain
Infliximab	2.09 (1.46 to 3.00)	Very low<br>⊕⊖⊖⊖	189 per 1,000	395 per 1,000 (276 to 567)	20.6% more (8.7% more to 37.8% more)	NA	The evidence is very uncertain
Ustekinumab	2.04 (1.69 to 2.46)	Moderate<br>⊕⊕⊕⊖	189 per 1,000	386 per 1,000 (319 to 465)	19.7% more (13% more to 27.6% more)	5 (4 to 8)	It's probably better than placebo by a small effect size (ranging from small to moderate)
Upadacitinib	1.76 (1.33 to 2.32)	Low<br>⊕⊕⊖⊖	189 per 1,000	333 per 1,000 (251 to 438)	14.4% more (6.2% more to 24.9% more)	NA	It may be better than placebo by a small effect size (ranging from trivial to moderate)
Mirikizumab	1.65 (1.15 to 2.37)	Low<br>⊕⊕⊖⊖	189 per 1,000	312 per 1,000 (217 to 448)	12.3% more (2.8% more to 25.9% more)	NA	It may be better than placebo by a small effect size (ranging from trivial to moderate)
Vedolizumab	1.57 (1.15 to 2.14)	Low<br>⊕⊕⊖⊖	189 per 1,000	297 per 1,000 (217 to 404)	10.8% more (2.8% more to 21.5% more)	NA	It may be better than placebo by a small effect size (ranging from trivial to moderate)
Filgotinib	1.55 (1.17 to 2.06)	Low	189 per 1,000			NA	

		⊕⊕⊖⊖		293 per 1,000 (221 to 389)	10.4% more (3.2% to 20% more)		It may be better than placebo by a small effect size (ranging from trivial to small)
Natalizumab	1.37 (1.07 to 1.75)	Low<br>⊕⊕⊖⊖	189 per 1,000	259 per 1,000 (202 to 331)	7% more (1.3% more to 14.2% more)	NA	It may be better than placebo by a trivial effect size (ranging from trivial to small)
Etrolizumab	1.3 (0.87 to 1.95)	Very Low<br>⊕⊖⊖⊖	189 per 1,000	246 per 1,000 (164 to 369)	5.7% more (2.5% less to 18% more)	NA	The evidence is very uncertain
Purine analogues	1.21 (0.97 to 1.5)	Very low<br>⊕⊖⊖⊖	189 per 1,000	229 per 1,000 (183 to 284)	4% more (0.6% less to 9.5% more)	NA	The evidence is very uncertain
Tesnatilimab	1.19 (0.77 to 1.83)	Very low<br>⊕⊖⊖⊖	189 per 1,000	225 pe 1,000 (146 to 346)	3.6% more (4.3% less to 15.7% more)	NA	The evidence is very uncertain
Certolizumab	1.17 (0.87 to 1.57)	Low<br>⊕⊕⊖⊖	189 per 1,000	221 per 1,000 (164 to 297)	3.2% more (2.5% less to 10.8% more)	NA	It may be the same as placebo with an effect that can range from trivially less than placebo to small effect

							more than placebo
Tofacitinib	1.18 (0.8 to 1.73)	Low<br>⊕⊕⊖⊖	189 per 1,000	223 per 1,000 (151 to 327)	3.4% more (3.8% less to 13.8% more)	NA	It may be the same as placebo with an effect that can range from trivially less than placebo to small effect more than placebo
Onercept	1 (0.5 to 2.01)	Very low<br>⊕⊖⊖⊖	189 per 1,000	189 per 1,000 (95 to 380)	0% (9.5% less to 19.1% more)	NA	The evidence is very uncertain
Methotrexate	1.06 (0.81 to 1.4)	Very low<br>⊕⊖⊖⊖	189 per 1,000	200 per 1,000 (153 to 265)	1.1% more (3.6% less to 7.6% more)	NA	The evidence is very uncertain
Andecaliximab	0.76 (0.33 to 1.78)	Very low<br>⊕⊖⊖⊖	189 per 1,000	144 per 1,000 (62 to 336)	4.5% less (12.7% less to 14.7% more)	NA	The evidence is very uncertain
Etanercept	0.43 (0.09 to 2.19)	Very low<br>⊕⊖⊖⊖	189 per 1,000	81 per 1,000 (17 to 414)	10.8% less (17.2% less to 22.5% more)	NA	The evidence is very uncertain

Apilimod mesylate	0.67 (0.34 to 1.35)	Low<br>⨁⨁⊖⊖	189 per 1,000	127 per 1,000 (64 to 255)	6.2% less (12.5% less to 6.6% more)	NA	It may be the same as placebo with an effect that can range from a small effect less than placebo to a trivial effect more than placebo

GRADE Working Group grades of evidence

High certainty: we are very confident that the true effect lies close to that of the estimate of the effect.

Moderate certainty: we are moderately confident in the effect estimate; the true effect is likely to be close to the estimate of the effect, but there is a possibility that it is substantially different.

Low certainty: our confidence in the effect estimate is limited; the true effect may be substantially different from the estimate of the effect.

Very low certainty: we have very little confidence in the effect estimate; the true effect is likely to be substantially different from the estimate of effect.

CI: confidence interval; RR: risk ratio

[a] The risk with placebo has been calculated based on the cumulative placebo rates of all studies with a placebo arm.
[b]The risk with treatment has been calculated by multiplying the risk with control with the RR(95% CI). If the calculation results in more than 1000 per 1000 people the number has been capped to 1000. Numbers have been rounded up to the closest whole number.
[c]The % risk difference has been calculated by subtracting the risk with control from the risk with treatment(95% CI) and dividing by 10. If the calculation results in more than 100% the number has been capped to 100%. Numbers have been rounded up to the closest whole number.
*red colouring indicates the treatment crosses the line of no effect

Sucra	Intervention (n=32)	network estimate RR	lower 95%CI	higher 95% CI	Number of direct studies	Direct GRADE	Reasons for direct downgrade	Indirect GRADE	Reasons for indirect downgrade	Network GRADE	Reasons for network downgrade
1	AdalimumabAzathioprine	2.87	1.99	4.14	0	x	x	moderate	rob in the strongest loop	moderate	none
2	NatalizumabInfliximabAzathioprine	2.99	1.32	6.81	0	x	x	low	rob in the strongest loop	very low	once imprecision

3	Guselkumab	2.5	1.95	3.21	4	moderate	once RoB	modera te	once rob in loop	moderate	none
4	Adalimumab	2.46	1.84	3.29	2	moderate	once rob	modera te	rob in the strongest loop	moderate	none
5	Humicade	3.14	0.7	13.94	1	moderate	once rob	x	x	very low	twice imprecision
6	InfliximabAzathioprine	2.43	1.71	3.44	0	x	x	modera te	rob in the strongest loop	moderate	none
7	Risankizumab	2.26	1.79	2.86	3	moderate	once inconsistency	modera te	once rob in loop	very low	once imprecision, twice incoherence
8	CTP13Azathioprine	2.29	1.32	3.95	0	x	x	modera te	rob in the strongest loop	low	once imprecision
9	BI695501	2.18	1.36	3.49	0	x	x	modera te	rob in the strongest loop	low	once imprecision
10	MethotrexateInfliximab	2.13	1.3	3.48	0	x	x	low	rob in the strongest loop	very low	once imprecision
11	Infliximab	2.09	1.46	3	2	moderate	once due to rob	modera te	rob in the strongest loop	very low	once imprecision; once incoherence
12	Ustekinumab	2.04	1.69	2.46	7	high	none	high	none	moderate	once incoherence
13	Upadacitinib	1.76	1.33	2.32	3	high	none	x	x	low	twice imprecision
14	Mirikizumab	1.65	1.15	2.37	2	high	none	x	x	low	twice imprecision
15	Vedolizumab	1.57	1.15	2.14	5	high	none	x	x	low	twice imprecision
16	Prednisone	n/a	n/a	n/a	n/a	n/a	n/a	n/a	n/a	n/a	n/a
17	Filgotinib	1.55	1.17	2.06	3	high	none	x	x	low	twice imprecision
18	Natalizumab	1.37	1.07	1.75	4	high	none	x	x	low	twice imprecision
19	Etrolizumab	1.3	0.87	1.95	2	moderate	once inconsistency	x	x	very low	twice imprecision
20	5-ASA	n/a	n/a	n/a	n/a	n/a	n/a	n/a	n/a	n/a	n/a
21	Corticosteroids	n/a	n/a	n/a	n/a	n/a	n/a	n/a	n/a	n/a	n/a
22	Azathioprine	1.21	0.97	1.5	5	moderate	from Chande	low	rob and inconsistency in the loop	very low	twice imprecision
23	Tesnatilimab	1.19	0.77	1.83	2	high	none	high	none	very low	twice imprecision, twice incoherence
24	Certolizumab	1.17	0.87	1.57	3	moderate	once due to rob	x	x	low	once imprecision
25	Tofacitinib	1.18	0.8	1.73	2	high	none	x	x	low	twice imprecision
26	Onercept	1.00	0.5	2.01	1	moderate	once rob	low	rob in the strongest loop	very low	twice imprecision
27	Everolimus	n/a	n/a	n/a	n/a	n/a	n/a	n/a	n/a	n/a	n/a

28	Methotrexate	1.06	0.81	1.4	3	low	from McDonald	low	rob and inconsistency in the loop	very low	once imprecision
29	Placebo	1	n/a	n/a	n/a	n/a	n/a	n/a	n/a	n/a	n/a
30	Andecaliximab	0.76	0.33	1.78	1	moderate	once due to rob	x	x	very low	twice imprecision
31	Etanercept	0.43	0.09	2.19	1	moderate	once due to rob	x	x	very low	twice imprecision
32	Apilimod Mesylate	0.67	0.34	1.35	1	high	none	x	x	low	twice imprecision

Clinical response							
Patient or population: people with active Crohn's disease							
Settings: hospital setting							
Intervention: biologics/purine analogues							
Comparison: placebo							
**Treatment**	**Network evidence**		**Anticipated absolute effects for network estimate**			**NNT (95% CI)**	**Notes**
	**RR (95% CI)**	**Certainty**	**Risk with Placebo[a]**	**Risk with Agent[b]**	**% Risk Difference with Agent[c]**		
CTP13 with purine analogues	2.76 (1.44 to 5.29)	Low ⊕⊕⊖⊖	296 per 1,000	817 per 1,000 (426 to 1000)	52.1% more (13% more to 100% more)	NA	It may be better than placebo by a large effect size (ranging from trivial to large)
Adalimumab and purine analogues	2.68 (1.75 to 4.09)	Moderate ⊕⊕⊕⊖	296 per 1,000	793 per 1,000 (518 to 1000)	49.7% more (22.2% more to 91.5% more)	2 (1 to 5)	It's probably better than placebo by a large effect size (ranging from small to large)
Infliximab with purine analogues	2.69 (1.65 to 4.4)	Moderate ⊕⊕⊕⊖	296 per 1,000	796 per 1,000 (518 to 1000)	50% more (22.2% more to 91.5% more)	2 (1 to 5)	It's probably better than placebo by a large effect size (ranging from small to large)
BI695501	2.81 (1.6 to 4.94)	Low ⊕⊕⊖⊖	296 per 1,000	832 per 1,000 (474 to 1000)	53.6% more (17.8% more to 100% more)	NA	It may be better than placebo by a large effect size (ranging from small to large)
Adalimumab	2.52 (1.72 to 3.69)	Low	296 per 1,000	746 per 1,000 (509 to 1000)	45% more (21.3% to 79.6% more)	3 (2 to 6)	It may be better than

		⊕⊕⊖⊖					placebo by a large effect size (ranging from small to large)
Infliximab	2.5 (1.63 to 3.83)	Moderate<br>⊕⊕⊕⊖	296 per 1,000	740 per 1,000 (482 to 1000)	44.4% more (18.6% more to 83.8% more)	3 (2 to 6)	It's probably better than placebo by a large effect size (ranging from small to large)
Risankizumab	2.11 (1.59 to 2.8)	Moderate<br>⊕⊕⊕⊖	296 per 1,000	625 per 1,000 (471 to 829)	32.9% more (17.5% more to 53.3% more)	NA	It's probably better than placebo by a moderate effect size (ranging from moderate to large)
Guselkumab	2.08 (1.52 to 2.84)	Low<br>⊕⊕⊖⊖	296 per 1,000	616 per 1,000 (450 to 841)	32% more (15.4% more to 54.5% more)	NA	It's maybe better than placebo by a moderate effect size (ranging from small to large)
Humicade	2.16 (0.97 to 4.78)	Very low<br>⊕⊖⊖⊖	296 per 1,000	639 per 1,000 (287 to 1000)	34.3% more (0.9% less to 100% more)	NA	the evidence is very uncertain
Ustekinumab	1.87 (1.54 to 2.27)	Moderate<br>⊕⊕⊕⊖	296 per 1,000	554 per 1,000 (456 to 672)	25.8% more (16% more to 37.6% more)	4 (3 to 6)	It's probably better than placebo by a moderate effect size (ranging from small to large)
Upadacitinib	1.76 (1.29 to 2.39)	Low	296 per 1,000			NA	

		⊕⊕⊖⊖		521 per 1,000 (382 to 707)	22.5% more (8.6% more to 41.1% more)		It may be better than placebo by a small effect size (ranging from trivial to large)
Purine analogues	1.67 (1.14 to 2.46)	Very low<br>⊕⊖⊖⊖	296 per 1,000	494 per 1,000 (337 to 728)	19.8% more (4.1% more to 43.2% more)	NA	the evidence is very uncertain
Mirikizumab	1.51 (1.08 to 2.12)	Very low<br>⊕⊖⊖⊖	296 per 1,000	447 per 1,000 (320 to 628)	15.1% more (2.4% more to 33.2% more)	NA	the evidence is very uncertain
Natalizumab	1.42 (1.1 to 1.83)	Very low<br>⊕⊖⊖⊖	296 per 1,000	420 per 1,000 (326 to 542)	12.4% more (3% more to 24.6% more)	NA	the evidence is very uncertain
Vedolizumab	1.35 (1.05 to 1.74)	Low<br>⊕⊕⊖⊖	296 per 1,000	400 per 1,000 (311 to 515)	10.4% more (1.5% more to 21.9% more)	NA	It may be better than placebo by a trivial effect size (ranging from trivial to small)
Filgotinib	1.32 (1.02 to 1.73)	Low<br>⊕⊕⊖⊖	296 per 1,000	391 per 1,000 (302 to 512)	9.5% more (0.6% more to 21.6% more)	NA	It may be better than placebo by a trivial effect size (ranging from trivial to small)
Tofacitinib	1.3 (0.9 to 1.87)	Low	296 per 1,000	385 per 1,000 (266 to 554)	8.9% more (3% less 25.8% more)	NA	It may be the same as

		⊕⊕⊖⊖					placebo with an effect that can range from trivially less than placebo to moderate effect more than placebo
Tesnatilimab	1.26 (0.85 to 1.87)	very low<br>⊕⊖⊖⊖	296 per 1,000	373 per 1,000 (252 to 554)	7.7% more (4.4% less to 25.8% more)	NA	the evidence is very uncertain
Certolizumab	1.17 (0.91 to 1.5)	Low<br>⊕⊕⊖⊖	296 per 1,000	346 per 1,000 (269 to 444)	5% more (2.7% less to 14.8% more)	NA	It may be the same as placebo with an effect that can range from trivially less than placebo to small effect more than placebo
Andecaliximab	0.92 (0.32 to 1.09)	Very low<br>⊕⊖⊖⊖	296 per 1,000	272 per 1,000 (95 to 323)	2.4% less (20.1% less to 2.7% more)	NA	the evidence is very uncertain
Onercept	1.08 (0.6 to 1.94)	Very low<br>⊕⊖⊖⊖	296 per 1,000	320 per 1,000 (178 to 574)	2.4% more (11.8% less to 27.8% more)	NA	the evidence is very uncertain
Etanercept	0.87 (0.39 to 1.93)	Very low<br>⊕⊖⊖⊖	296 per 1,000	258 per 1,000 (115 to 571)	3.8% less (18.1% less to 27.5% more)	NA	the evidence is very uncertain

Apilimod mesylate	0.59 (0.32 to 1.09)	Low<br>⊕⊕⊖⊖	296 per 1,000	175 per 1,000 (95 to 323)	12.1% less (20.1% less to 2.7 more)	NA	It may be the same as placebo with an effect that can range from small less than placebo to trivial effect more than placebo

GRADE Working Group grades of evidence

High certainty: we are very confident that the true effect lies close to that of the estimate of the effect.

Moderate certainty: we are moderately confident in the effect estimate; the true effect is likely to be close to the estimate of the effect, but there is a possibility that it is substantially different.

Low certainty: our confidence in the effect estimate is limited; the true effect may be substantially different from the estimate of the effect.

Very low certainty: we have very little confidence in the effect estimate; the true effect is likely to be substantially different from the estimate of effect.

CI: confidence interval; RR: risk ratio

[a] The risk with placebo has been calculated based on the cumulative placebo rates of all studies with a placebo arm.
[b]The risk with treatment has been calculated by multiplying the risk with control with the RR(95% CI). If the calculation results in more than 1000 per 1000 people the number has been capped to 1000. Numbers have been rounded up to the closest whole number.
[c]The % risk difference has been calculated by subtracting the risk with control from the risk with treatment(95% CI) and dividing by 10. If the calculation results in more than 100% the number has been capped to 100%. Numbers have been rounded up to the closest whole number.
*red colouring indicates the treatment crosses the line of no effect

Sucra	Intervention (n=24)	network estimate RR	lower 95%CI	higher 95% CI	Number of direct studies	Direct GRADE	Reasons for direct downgrade	Indirect GRADE	Reasons for indirect downgrade	Network GRADE	Reasons for network downgrade
1	CTP13Azathioprine	2.76	1.44	5.29	0	x	x	moderate	RoB in the loop	low	once imprecision
2	AdalimumabAzathioprine	2.68	1.75	4.09	0	x	x	moderate	RoB in the loop	moderate	none

3	InfliximabAzathioprine	2.69	1.75	4.09	0	x	x	moderate	RoB in the loop	moderate	none
4	BI695501	2.81	1.6	4.94	0	x	x	moderate	RoB in the loop	low	once imprecision
5	Adalimumab	2.52	1.72	3.69	2	moderate	once RoB	moderate	RoB in the loop	low	once incoherence
6	Infliximab	2.5	1.63	3.83	2	moderate	once RoB	moderate	RoB in the loop	moderate	none
7	Risankizumab	2.11	1.59	2.80	3	moderate	once inconsistency	x	x	moderate	none
8	Guselkumab	2.08	1.52	2.84	2	moderate	once RoB	moderate	once RoB	low	once incoherence
9	Humicade	2.16	0.97	4.78	1	moderate	once RoB	x	x	very low	twice due to imprecision
10	Ustekinumab	1.87	1.54	2.27	7	high	none	x	x	moderate	once incoherence
11	Upadacitinib	1.76	1.29	2.39	3	moderate	once RoB	x	x	low	once imprecision
12	Azathioprine	1.67	1.14	2.46	3	low	once RoB, once inconsistency	moderate	rob in the loop	very low	once imprecision; once incoherence
13	Mirikizumab	1.51	1.08	2.12	2	moderate	once inconsistency	x	x	very low	twice imprecision
14	Natalizumab	1.42	1.1	1.83	3	low	once inconsistency, once RoB	x	x	very low	twice imprecision
15	Vedolizumab	1.35	1.05	1.74	5	high	none	x	x	low	twice imprecision
16	Filgotinib	1.32	1.02	1.73	3	high	none	x	x	low	twice imprecision
17	Tofacitinib	1.3	0.9	1.87	2	high	none	x	x	low	twice imprecision
18	Tesnatilimab	1.26	0.85	1.87	2	high	none	x	x	very low	twice imprecision, once incoherence
19	Certolizumab	1.17	0.91	1.50	4	moderate	once RoB	x	x	low	once imprecision
20	Andecaliximab	0.92	0.32	1.09	1	moderate	once RoB	x	x	very low	twice imprecision
21	Onercept	1.08	0.6	1.94	1	moderate	once RoB	x	x	very low	twice imprecision
22	Etanercept	0.87	0.39	1.93	1	moderate	once RoB	x	x	very low	twice imprecision
23	Placebo	1									
24	Apilimod Mesylate	0.59	0.32	1.09	1	high	none	x	x	low	twice imprecision

Endoscopic remission							
Patient or population: people with active Crohn's disease							
Settings: hospital setting							
Intervention: biologics/purine analogues							
Comparison: placebo							
**Treatment**	**Network evidence**		**Anticipated absolute effects for network estimate**			**NNT (95% CI)**	**Notes**
	**RR (95% CI)**	**Certainty**	**Risk with Placebo**[a]	**Risk with Agent**[b]	**% Risk Difference with Agent**[c]		
Upadacitinib	5.1 (2.74 to 9.49)	Moderate ⊕⊕⊕⊖	70 per 1,000	358 per 1,000 (192 to 664)	28.8% more (12.2% more to 59.4% more)	3 (2 to 8)	It's probably better than placebo by a moderate effect size (ranging from small to large)
Risankizumab	3.48 (2.18 to 5.58)	Moderate ⊕⊕⊕⊖	70 per 1,000	244 per 1,000 (153 to 391)	17.4% more (8.3% more to 32.1% more)	6 (3 to 12)	It's probably better than placebo by a moderate effect size (ranging from trivial to large)
Mirikizumab	3.28 (1.44 to 7.48)	Low ⊕⊕⊖⊖	70 per 1,000	230 per 1,000 (101 to 524)	16% more (3.1% more to 45.4% more)	NA	It may be better than placebo by a small effect size (ranging from trivial to large)
Adalimumab	1.83 (1.05 to 3.2)	Very low ⊕⊖⊖⊖	70 per 1,000	128 per 1,000 (74 to 224)	5.8% more (0.4% more to 15.4% more)	NA	The evidence is very uncertain

Ustekinumab	1.71 (1 to 2.92)	Very low<br>⊕⊖⊖⊖	70 per 1,000	120 per 1,000 (70 to 204)	5% more (0% to 13.4% more)	NA	The evidence is very uncertain
Adalimumab with purine analogues	1.66 (0.74 to 3.72)	Very low<br>⊕⊖⊖⊖	70 per 1,000	116 per 1,000 (52 to 260)	4.6% more (1.8% less to 19% more)	NA	The evidence is very uncertain
Guselkumab	1.69 (0.97 to 2.95)	Very low<br>⊕⊖⊖⊖	70 per 1,000	118 per 1,000 (68 to 207)	4.8% more (0.2% less to 13.7% more)	NA	The evidence is very uncertain
Etrolizumab	1.56 (0.63 to 3.86)	Low<br>⊕⊕⊖⊖	70 per 1,000	109 per 1,000 (44 to 270)	3.9% more (2.6% less to 20% more)	NA	It may be the same as placebo (trivial effect less to moderate effect more)
Filgotinib	1.13 (0.62 to 2.04)	Very low<br>⊕⊖⊖⊖	70 per 1,000	79 per 1,000 (43 to 143)	0.9% more (2.7% less to 7.3% more)	NA	The evidence is very uncertain
Tesnatilimab	0.83 (0.36 to 1.92)	Low<br>⊕⊕⊖⊖	70 per 1,000	58 per 1,000 (25 to 134)	1.2% less (4.5% less to 6.4% more)	NA	It may be the same as placebo (trivial effect less to trivial

							effect more)

GRADE Working Group grades of evidence

High certainty: we are very confident that the true effect lies close to that of the estimate of the effect.

Moderate certainty: we are moderately confident in the effect estimate; the true effect is likely to be close to the estimate of the effect, but there is a possibility that it is substantially different.

Low certainty: our confidence in the effect estimate is limited; the true effect may be substantially different from the estimate of the effect.

Very low certainty: we have very little confidence in the effect estimate; the true effect is likely to be substantially different from the estimate of effect.

CI: confidence interval; RR: risk ratio

[a] The risk with placebo has been calculated based on the cumulative placebo rates of all studies with a placebo arm.
[b]The risk with treatment has been calculated by multiplying the risk with control with the RR(95% CI). If the calculation results in more than 1000 per 1000 people the number has been capped to 1000. Numbers have been rounded up to the closest whole number.
[c]The % risk difference has been calculated by subtracting the risk with control from the risk with treatment(95% CI) and dividing by 10. If the calculation results in more than 100% the number has been capped to 100%. Numbers have been rounded up to the closest whole number.
*red colouring indicates the treatment crosses the line of no effect

Sucra	Intervention (n=11)	network estimate RR	lower 95%CI	higher 95% CI	Number of direct studies	Direct GRADE	Reasons for direct downgrade	Indirect GRADE	Reasons for indirect downgrade	Network GRADE	Reasons for network downgrade
1	Upadacitinib	5.1	2.74	9.49	3	Moderate	once Rob	x	x	**Moderate**	none
2	Risankizumab	3.48	2.18	5.58	3	High	none	moderate	once rob	**Moderate**	once impрecision
3	Mirikizumab	3.28	1.44	7.48	2	High	none	x	x	**Low**	twice imprecision
4	Adalimumab	1.83	1.05	3.2	1	Moderate	once Rob	moderate	once rob	**very low**	twice imprecision
5	Ustekinumab	1.71	1	2.92	1	High	none	high	none	**very low**	twice imprecision, once incoherence
6	AdalimumabAza	1.66	0.74	3.72	0	x	x	moderate	once rob	**very low**	twice imprecision
7	Guselkumab	1.69	0.97	2.95	2	Moderate	once Rob	moderate	once rob	**very low**	twice imprecision

8	Etrolizumab	1.56	0.63	3.86	1	High	none	x	x	**low**	twice imprecision
9	Filgotinib	1.13	0.62	2.04	3	Moderate	once inconsistency	x	x	**very low**	twice imprecision
10	Placebo	1									
11	Tesnatilimab	0.83	0.36	1.92	2	Low	twice inconsistency	x	x	**low**	none

Withdrawals due to adverse events								
Patient or population: people with active Crohn's disease								
Settings: hospital setting								
Intervention: biologics/purine analogues/methotrexate								
Comparison: placebo								
**Treatment**	**Network evidence**		**Anticipated absolute effects for network estimate**			**NNT (95% CI)**	**Notes**
	**RR (95% CI)**	**Certainty**	**Risk with Placebo[a]**	**Risk with Agent[b]**	**% Risk Difference with Agent[c]**		
Risankizumab	0.36 (0.22 to 0.57)	Low ⊕⊕⊖⊖	137 per 1,000	49 per 1,000 (30 to 78)	8.8% less (10.7% less to 5.9% less)	NA	It maybe leads to less withdrawals due to adverse events than placebo by a small effect size (ranging from small to trivial)
Mirikizumab	0.39 (0.15 to 1.01)	Low ⊕⊕⊖⊖	137 per 1,000	53 per 1,000 (21 to 138)	8.4% less (11.6% less to 0.1% more)	NA	It maybe leads to similar withdrawals due to adverse events as placebo. Ranging from small effect less withdrawals to trivially more.
Tofacitinib	0.53 (0.27 to 1.04)	Low ⊕⊕⊖⊖	137 per 1,000	73 per 1,000 (37 to 142)	6.4% less (10% less to 0.5% more)	NA	It maybe leads to similar withdrawals due to adverse events as placebo. Ranging from small effect less withdrawals to trivially more.
Onercept	0.52 (0.12 to 2.22)	very low ⊕⊖⊖⊖	137 per 1,000	71 per 1,000 (16 to 304)	6.6% less (12.1% less to 16.7% more)	NA	The evidence is very uncertain
Ustekinumab	0.72 (0.53 to 0.97)	Low ⊕⊕⊖⊖	137 per 1,000	99 per 1,000 (73 to 133)	3.8% less (6.4% less to 0.4% less)	NA	It maybe leads to trivially less withdrawals due to adverse events than

							placebo (ranging from trivial to trivial effect sizes)
Vedolizumab	0.71 (0.42 to 1.22)	Low<br>⊕⊕⊖⊖	137 per 1,000	97 per 1,000 (58 to 167)	4% less (7.9% less to 3% more)	NA	It maybe leads to similar withdrawals due to adverse events as placebo. Ranging from trivial effect less withdrawals to trivially more.
Upadacitinib	0.8 (0.48 to 1.35)	Low<br>⊕⊕⊖⊖	137 per 1,000	110 per 1,000 (66 to 185)	2.7% less (7.1% less to 4.8% more)	NA	It maybe leads to similar withdrawals due to adverse events as placebo. Ranging from small effect less withdrawals to trivially more.
Natalizumab	0.8 (0.49 to 1.3)	Low<br>⊕⊕⊖⊖	137 per 1,000	110 per 1,000 (67 to 178)	2.7% less (7% less to 4.1% more)	NA	It maybe leads to similar withdrawals due to adverse events as placebo. Ranging from small effect less withdrawals to trivially more.
Humicade	0.81 (0.42 to 1.57)	Very low<br>⊕⊖⊖⊖	137 per 1,000	111 per 1,000 (58 to 215)	2.6% less (7.9% less to 7.8% more)	NA	The evidence is very uncertain
Apilimod Mesylate	0.84 (0.38 to 1.84)	Moderate<br>⊕⊕⊕⊖	137 per 1,000	123 per 1,000 (41 to 370)	1.4% less (9.6% less to 23.3% more)	NA	It maybe leads to similar withdrawals due to adverse events as placebo. Ranging from small effect less withdrawals to small more.
BI695501	0.9 (0.3 to 2.7)	very low<br>⊕⊖⊖⊖	137 per 1,000	126 per 1,000 (54 to 300)	1.6% less (8.8% less to 15.8% more)	NA	The evidence is very uncertain

Guselkumab	0.86 (0.55 to 1.37)	Low<br>⊕⊕⊖⊖	137 per 1,000	118 per 1,000 (75 to 188)	1.9% less (6.2% less to 5.1% more)	NA	It maybe leads to similar withdrawals due to adverse events as placebo. Ranging from trivial effect less withdrawals to trivially more.
Adalimumab	0.92 (0.47 to 1.79)	Very low<br>⊕⊖⊖⊖	137 per 1,000	126 per 1,000 (64 to 245)	1.1% less (7.3% less to 10.8% more)	NA	The evidence is very uncertain
Etrolizumab	0.86 (0.42 to 1.77)	Very low<br>⊕⊖⊖⊖	137 per 1,000	118 per 1,000 (58 to 242)	1.9% less (7.9% less to 10.5% more)	NA	The evidence is very uncertain
Certolizumab	0.97 (0.66 to 1.43)	Low<br>⊕⊕⊖⊖	137 per 1,000	133 per 1,000 (90 to 196)	0.4% less (4.7% less to 5.9% more)	NA	It maybe leads to similar withdrawals due to adverse events as placebo. Ranging from trivial effect less withdrawals to trivially more.
AdalimumabAzathioprine	1.06 (0.46 to 2.44)	very low<br>⊕⊖⊖⊖	137 per 1,000	145 per 1,000 (63 to 334)	0.8% more (7.4% less to 19.7% more)	NA	The evidence is very uncertain
Filgotinib	1.06 (0.66 to 1.72)	Low<br>⊕⊕⊖⊖	137 per 1,000	145 per 1,000 (90 to 236)	0.8% more (4.7% less to 9.9% more)	NA	It maybe leads to similar withdrawals due to adverse events as placebo. Ranging from trivial effect less

							withdrawals to small effect size more.
CTP13Azathioprine	1.25 (0.38 to 4.17)	very low<br>⊕⊖⊖⊖	137 per 1,000	171 per 1,000 (52 to 571)	3.4% more (8.5% less to 43.4% more)	NA	The evidence is very uncertain
Duvakitug	1.16 (0.57 to 2.36)	very low<br>⊕⊖⊖⊖	137 per 1,000	159 per 1,000 (78 to 323)	2.2% more (5.9% less to 18.6% more)	NA	The evidence is very uncertain
NatalizumabInfliximabAzathioprine	1.43 (0.3 to 6.74)	Very Low<br>⊕⊖⊖⊖	137 per 1,000	196 per 1,000 (41 to 923)	5.9% more (9.6% less to 78.6% more)	NA	The evidence is very uncertain
Infliximab	1.33 (0.56 to 3.16)	very low<br>⊕⊖⊖⊖	137 per 1,000	182 per 1,000 (77 to 433)	4.5% more (6% less to 29.6% more)	NA	The evidence is very uncertain
Etanercept	1.35 (0.58 to 3.16)	very low<br>⊕⊖⊖⊖	137 per 1,000	185 per 1,000 (79 to 433)	4.8% more (5.8% less to 29.6% more)	NA	The evidence is very uncertain
Tesnatilimab	1.51 (0.85 to 2.71)	Low<br>⊕⊕⊖⊖	137 per 1,000	207 per 1,000 (116 to 371)	7% more (2.1% less to 23.4% more)	NA	It maybe leads to similar withdrawals due to adverse events as placebo. Ranging from trivial effect less withdrawals to large effect size more.
Andecaliximab	2.11 (0.26 to 17.13)	Very low<br>⊕⊖⊖⊖	137 per 1,000	289 per 1,000 (36 to 1000)	15.2% more (10.1% less to 100% more)	NA	The evidence is very uncertain

InfliximabAzathioprine	1.57 (0.67 to 3.7)	Very low<br>⊕⊖⊖⊖	137 per 1,000	215 per 1,000 (92 to 507)	7.8% more (4.5% less to 37% more)	NA	The evidence is very uncertain
MethotrexateInfliximab	2.66 (0.2 to 35.87)	Very low<br>⊕⊖⊖⊖	137 per 1,000	364 per 1,000 (27 to 1000)	22.7% more (11% less to 100% more)	NA	The evidence is very uncertain
Purine analogues	1.99 (1.12 to 3.54)	Very low<br>⊕⊖⊖⊖	137 per 1,000	273 per 1,000 (153 to 485)	13.6% more (1.6% more to 34.8% more)	NA	The evidence is very uncertain
Methotrexate	3.27 (1.22 to 8.74)	Very low<br>⊕⊖⊖⊖	137 per 1,000	448 per 1,000 (167 to 1000)	31.1% more (3% more to 100% more)	NA	The evidence is very uncertain

GRADE Working Group grades of evidence

High certainty: we are very confident that the true effect lies close to that of the estimate of the effect.

Moderate certainty: we are moderately confident in the effect estimate; the true effect is likely to be close to the estimate of the effect, but there is a possibility that it is substantially different.

Low certainty: our confidence in the effect estimate is limited; the true effect may be substantially different from the estimate of the effect.

Very low certainty: we have very little confidence in the effect estimate; the true effect is likely to be substantially different from the estimate of effect.

CI: confidence interval; RR: risk ratio

[a] The risk with placebo has been calculated based on the cumulative placebo rates of all studies with a placebo arm.
[b]The risk with treatment has been calculated by multiplying the risk with control with the RR(95% CI). If the calculation results in more than 1000 per 1000 people the number has been capped to 1000. Numbers have been rounded up to the closest whole number.

[c]The % risk difference has been calculated by subtracting the risk with control from the risk with treatment(95% CI) and dividing by 10. If the calculation results in more than 100% the number has been capped to 100%. Numbers have been rounded up to the closest whole number.
*red colouring indicates the treatment crosses the line of no effect

Sucra	Intervention (n=32)	network estimate RR	lower 95%CI	higher 95% CI	Direct studies	Direct GRADE	Reasons for direct downgrade	Indirect GRADE	Reasons for indirect downgrade	Network GRADE	Reasons for network downgrade
1	Risankizumab	0.36	0.22	0.57	3	moderate	once RoB	x	x	**low**	once imprecision
2	Mirikizumab	0.39	0.15	1.01	2	high	none	high	none	**low**	twice imprecision
3	Tofacitinib	0.53	0.27	1.04	2	high	none	x	x	**low**	twice imprecision
4	5-ASA	n/a	n/a	n/a	n/a	n/a	n/a	n/a	n/a	n/a	n/a
5	Onercept	0.52	0.12	2.22	1	moderate	once rob	x	x	**very low**	twice imprecision
6	Ustekinumab	0.72	0.53	0.97	7	moderate	once RoB	moderate	once inconsistency in loop	**low**	once imprecision
7	Vedolizumab	0.71	0.42	1.22	5	moderate	once inconsistency	x	x	**low**	once imprecision
8	Upadacitinib	0.8	0.48	1.35	3	moderate	once RoB	x	x	**low**	once imprecision
9	Natalizumab	0.8	0.49	1.3	4	moderate	once rob	x	x	**low**	once imprecision
10	Humicade	0.81	0.42	1.57	1	moderate	once rob	x	x	**very low**	twice imprecision
11	Apilimod Mesylate	0.84	0.38	1.84	1	high	none	x	x	**low**	twice imprecision
12	BI695501	0.9	0.3	2.7	0	x	x	moderate	once RoB in loop	**very low**	twice imprecision
13	Guselkumab	0.86	0.55	1.37	3	low	twice inconsistency	low	twice inconsistency in loop	**low**	none
14	Adalimumab	0.92	0.47	1.79	2	moderate	once RoB	moderate	once RoB in loop	**very low**	twice incoherence and once imprecision

15	Etrolizumab	0.86	0.42	1.77	2	moderate	once inconsistency	x	x	**very low**	twice imprecision
16	Everolimus	n/a	n/a	n/a	n/a	n/a	n/a	n/a	n/a	n/a	n/a
17	Certolizumab	0.97	0.66	1.43	4	low	once rob, once inconsistency	x	x	**low**	none
18	Placebo	1	n/a	n/a	n/a	n/a	n/a	n/a	n/a	n/a	n/a
19	AdalimumabAzathioprine	1.06	0.46	2.44	0	x	x	low	twice RoB in loop	**very low**	twice imprecision
20	Filgotinib	1.06	0.66	1.72	3	high	none	x	x	**low**	twice imprecision
21	CTP13Azathioprine	1.25	0.38	4.17	0	x	x	moderate	once RoB in loop	**very low**	twice imprecision
22	Duvakitug	1.16	0.57	2.36	1	low	twice RoB	x	x	**very low**	twice imprecision
23	NatalizumabInfliximabAzathioprine	1.43	0.3	6.74	0	x	x	low	twice RoB in loop	**very low**	twice imprecision
24	Infliximab	1.33	0.56	3.16	1	low	twice rob	moderate	once RoB in loop	**very low**	twice imprecision once incoherence
25	Etanercept	1.35	0.58	3.16	1	moderate	once rob	x	x	**very low**	twice imprecision
26	Tesnatilimab	1.51	0.85	2.71	2	high	none	x	x	**low**	twice imprecision
27	Corticosteroids	n/a	n/a	n/a	n/a	n/a	n/a	n/a	n/a	n/a	n/a
28	Andecaliximab	2.11	0.26	17.13	1	moderate	once rob	x	x	**very low**	twice imprecision
29	InfliximabAzathioprine	1.57	0.67	3.7	0	x	x	moderate	once RoB in loop	**very low**	twice imprecision
30	MethotrexateInfliximab	2.66	0.2	35.87	0	x	x	low	twice RoB in loop	**very low**	twice imprecision
31	Purine analogues	1.99	1.12	3.54	8	moderate	once RoB	moderate	once RoB in loop	**very low**	twice imprecision, once incoherence
32	Methotrexate	3.27	1.22	8.74	3	moderate	once RoB	x	x	**very low**	twice incoherence and twice imprecision

Serious Adverse events							
Patient or population: people with active Crohn's disease							
Settings: hospital setting							
Intervention: biologics/purine analogues/methotrexate							
Comparison: placebo							
**Treatment**	**Network evidence**		**Anticipated absolute effects for network estimate**			**NNT (95% CI)**	**Notes**
	**RR (95% CI)**	**Certainty**	**Risk with Placebo[a]**	**Risk with Agent[b]**	**% Risk Difference with Agent[c]**		
Risankizumab	0.48 (0.34 to 0.68)	Low ⊕⊕⊖⊖	95 per 1,000	43 per 1,000 (31 to 61)	4.7% less (5.9% less to 2.9% less)	NA	It maybe leads to less serious adverse events than placebo by a trivial effect size (ranging from trivial to trivial)
Etanercept	0.35 (0.08 to 1.6)	Low ⊕⊕⊖⊖	90 per 1,000	32 per 1,000 (7 to 144)	5.9% less (8.3% less to 5.4% more)	NA	It maybe leads to similar serious adverse events as placebo. Ranging from small effect less serious events to trivially more.
Apilimod Mesylate	0.62 (0.26 to 1.51)	Moderate ⊕⊕⊕⊖	90 per 1,000	56 per 1,000 (23 to 136)	3.4% less (6.7% less to 4.6% more)	NA	It probably leads to similar serious adverse events as placebo. Ranging from small effect less serious events to trivially more.
Mirikizumab	0.65 (0.47 to 0.89)	Low ⊕⊕⊖⊖	90 per 1,000	59 per 1,000 (42 to 80)	3.2% less (4.8% less to 1% less)	NA	It maybe leads to similar serious adverse events as placebo. Ranging from trivial effect less serious events to trivially more.
CTP13Azathioprine	0.73 (0.19 to 2.75)	very low ⊕⊖⊖⊖	90 per 1,000	66 per 1,000 (17 to 248)	2.4% less (7.3% less to 15.8% more)	NA	The evidence is very uncertain

Ustekinumab	0.8 (0.65 to 1)	Moderate<br>⊕⊕⊕⊖	90 per 1,000	72 per 1,000 (59 to 90)	1.8% less (3.2% less to 0%)	NA	It probably leads to similar serious adverse events as placebo. Ranging from trivial effect less serious events to the same as placebo.
NatalizumabInfliximabAzathioprine	0.58 (0.03 to 10.17)	very low<br>⊕⊖⊖⊖	90 per 1,000	52 per 1,000 (3 to 915)	3.8% less (8.7% less to 82.5% more)	NA	The evidence is very uncertain
AdalimumabAzathioprine	0.78 (0.17 to 3.54)	very low<br>⊕⊖⊖⊖	90 per 1,000	70 per 1,000 (15 to 319)	2% less (7.5% less to 22.9% more)	NA	The evidence is very uncertain
BI695501	0.71 (0.23 to 2.17)	very low<br>⊕⊖⊖⊖	90 per 1,000	64 per 1,000 (21 to 195)	2.6% less (6.9% less to 10.5% more)	NA	The evidence is very uncertain
Guselkumab	0.81 (0.6 to 1.09)	Moderate<br>⊕⊕⊕⊖	90 per 1,000	73 per 1,000 (54 to 98)	1.7% less (3.6% less to 0.8% more)	NA	It probably leads to similar serious adverse events as placebo. Ranging from trivial effect less serious events to trivial more.
Natalizumab	0.81 (0.54 to 1.19)	Moderate<br>⊕⊕⊕⊖	90 per 1,000	73 per 1,000 (49 to 107)	1.7% less (4.1% less to 1.7% more)	NA	It probably leads to similar serious adverse events as placebo. Ranging from trivial effect less serious events to trivially more.
Etrolizumab	0.84 (0.38 to 1.85)	Moderate<br>⊕⊕⊕⊖	90 per 1,000	76 per 1,000 (34 to 167)	1.4% less (5.6% less to 7.7% more)	NA	It probably leads to similar serious adverse events as placebo. Ranging from trivial effect less

							serious events to small effect more.
Andecaliximab	0.88 (0.27 to 2.84)	very low<br>⊕⊖⊖⊖	90 per 1,000	79 per 1,000 (24 to 256)	1.1% less (6.6% lesss to 16.6% more)	NA	The evidence is very uncertain
Adalimumab	0.91 (0.56 to 1.47)	Moderate<br>⊕⊕⊕⊖	90 per 1,000	82 per 1,000 (50 to 132)	0.8% less (4% less to 4.2% more)	NA	It probably leads to similar serious adverse events as placebo. Ranging from trivial effect less serious events to trivial more.
Tofacitinib	0.95 (0.54 to 1.65)	Moderate<br>⊕⊕⊕⊖	90 per 1,000	86 per 1,000 (49 to 149)	0.5% less (4.1% less to 5.9% more)	NA	It probably leads to similar serious adverse events as placebo. Ranging from trivial effect less serious events to trivial effect more.
Vedolizumab	0.9 (0.71 to 1.18)	Moderate<br>⊕⊕⊕⊖	90 per 1,000	82 per 1,000 (64 to 106)	0.8% less (2.6% less to 1.6% more)	NA	It probably leads to similar serious adverse events as placebo. Ranging from trivial effect less serious events to trivial more.
Upadacitinib	1.05 (0.7 to 1.59)	Moderate<br>⊕⊕⊕⊖	90 per 1,000	95 per 1,000 (63 to 143)	0.5% more (2.7% less to 5.3% more)	NA	It probably leads to similar serious adverse events as placebo. Ranging from trivial effect less serious events to trivial more.
Filgotinib	1.13 (0.82 to 1.54)	Very Low<br>⊕⊖⊖⊖	90 per 1,000	102 per 1,000 (74 to 139)	1.2% more (1.6% less to 4.9% more)	NA	The evidence is very uncertain
Onercept	1.12 (0.14 to 9.35)	Very Low<br>⊕⊖⊖⊖	90 per 1,000	101 per 1,000 (13 to 842)	1.1% more (7.7% less to 75.2% more)	NA	The evidence is very uncertain

InfliximabAzathioprine	1.11 (0.46 to 2.67)	very low<br>⊕⊖⊖⊖	90 per 1,000	100 per 1,000 (41 to 240)	1% more (4.9% less to 15% more)	NA	The evidence is very uncertain
Infliximab	1.5 (0.64 to 3.51)	Very low<br>⊕⊖⊖⊖	90 per 1,000	135 per 1,000 (58 to 316)	4.5% more (3.2% less to 22.6% more)	NA	The evidence is very uncertain
Tesnatilimab	1.59 (0.62 to 4.1)	Very low<br>⊕⊖⊖⊖	90 per 1,000	143 per 1,000 (56 to 369)	5.3% more (3.4% less to 27.9% more)	NA	The evidence is very uncertain
Certolizumab	1.41 (0.97 to 2.06)	Low<br>⊕⊕⊖⊖	90 per 1,000	127 per 1,000 (87 to 185)	3.7% more (0.3% less to 9.5% more)	NA	It maybe leads to similar serious adverse events as placebo. Ranging from trivial effect less serious events to small effect more.
Purine analogues	1.64 (0.75 to 3.61)	Very low<br>⊕⊖⊖⊖	90 per 1,000	148 per 1,000 (68 to 325)	5.8% more (2.3% less to 23.5% more)	NA	The evidence is very uncertain

GRADE Working Group grades of evidence

High certainty: we are very confident that the true effect lies close to that of the estimate of the effect.

Moderate certainty: we are moderately confident in the effect estimate; the true effect is likely to be close to the estimate of the effect, but there is a possibility that it is substantially different.
Low certainty: our confidence in the effect estimate is limited; the true effect may be substantially different from the estimate of the effect.

Very low certainty: we have very little confidence in the effect estimate; the true effect is likely to be substantially different from the estimate of effect.

CI: confidence interval; RR: risk ratio

[a] The risk with placebo has been calculated based on the cumulative placebo rates of all studies with a placebo arm.
[b]The risk with treatment has been calculated by multiplying the risk with control with the RR(95% CI). If the calculation results in more than 1000 per 1000 people the number has been capped to 1000. Numbers have been rounded up to the closest whole number.
[c]The % risk difference has been calculated by subtracting the risk with control from the risk with treatment(95% CI) and dividing by 10. If the calculation results in more than 100% the number has been capped to 100%. Numbers have been rounded up to the closest whole number.
*red colouring indicates the treatment crosses the line of no effect

**Sucra**	**Intervention (n=28)**	**network estimate RR**	**lower 95%CI**	**higher 95% CI**	**Direct studies**	**Direct GRADE**	**Reasons for direct downgrade**	**Indirect GRADE**	**Reasons for indirect downgrade**	**Network GRADE**	**Reasons for network downgrade**
1	5-ASA	n/a	n/a	n/a	n/a	n/a	n/a	n/a	n/a	n/a	n/a
2	Risankizumab	0.48	0.34	0.68	3	moderate	once RoB	x	x	**low**	once imprecision
3	Etanercept	0.35	0.08	1.6	1	moderate	once rob	x	x	**low**	once imprecision
4	Apilimod Mesylate	0.62	0.26	1.51	1	high	none	x	x	**moderate**	once imprecision
5	Mirikizumab	0.65	0.47	0.89	2	high	none	high	none	**low**	once imprecision, once incoherence
6	CTP13Azathioprine	0.73	0.19	2.75	0	x	x	moderate	once rob in loop	**very low**	twice imprecision
7	Ustekinumab	0.8	0.65	1	7	moderate	once RoB	moderate	once rob in loop	**moderate**	none
8	NatalizumabInfliximabAzathioprine	0.58	0.03	10.17	0	x	x	low	twice rob in loop	**very low**	twice imprecision
9	AdalimumabAzathioprine	0.78	0.17	3.54	0	x	x	low	twice rob in loop	**very low**	twice imprecision
10	BI695501	0.71	0.23	2.17	0	x	x	moderate	once rob in loop	**very low**	twice imprecision
11	Guselkumab	0.81	0.6	1.09	3	moderate	once RoB	moderate	once rob in loop	**moderate**	none
12	Natalizumab	0.81	0.54	1.19	3	moderate	once rob	x	x	**moderate**	none
13	Etrolizumab	0.84	0.38	1.85	1	high	none	x	x	**moderate**	once imprecision
14	Andecaliximab	0.88	0.27	2.84	1	moderate	once rob	x	x	**very low**	twice imprecision

15	Adalimumab	0.91	0.56	1.47	2	moderate	once RoB	moderate	once rob in loop	**moderate**	none
16	Tofacitinib	0.95	0.54	1.65	2	moderate	once RoB	x	x	**low**	once imprecision
17	Vedolizumab	0.91	0.71	1.18	5	moderate	once RoB	x	x	**moderate**	none
18	Placebo	1									
19	Upadacitinib	1.05	0.7	1.59	3	moderate	once RoB	x	x	**moderate**	none
20	Filgotinib	1.13	0.82	1.54	3	moderate	once RoB	x	x	**very low**	twice imprecision
21	Onercept	1.12	0.14	9.35	1	moderate	once rob	x	x	**very low**	twice imprecision
22	InfliximabAzathioprine	1.11	0.46	2.67	0	x	x	moderate	once rob in loop	**very low**	twice imprecision
23	Corticosteroids	n/a	n/a	n/a	n/a	n/a	n/a	n/a	n/a	n/a	n/a
24	Everolimus	n/a	n/a	n/a	n/a	n/a	n/a	n/a	n/a	n/a	n/a
25	Infliximab	1.5	0.64	3.51	1	low	twice rob	moderate	once rob in loop	**very low**	twice imprecision
26	Tesnatilimab	1.59	0.62	4.1	2	moderate	once inconsistency	x	x	**very low**	twice imprecision
27	Certolizumab	1.41	0.97	2.06	4	moderate	once rob	x	x	**low**	once imprecision
28	Purine analogues	1.64	0.75	3.61	2	moderate	once rob	moderate	once rob in loop	**very low**	twice imprecision

Total Adverse events							
Patient or population: people with active Crohn's disease							
Settings: hospital setting							
Intervention: biologics/purine analogues/methotrexate							
Comparison: placebo							
**Treatment**	**Network evidence**		**Anticipated absolute effects for network estimate**			**NNT (95% CI)**	**Notes**
	**RR (95% CI)**	**Certainty**	**Risk with Placebo**[a]	**Risk with Agent**[b]	**% Risk Difference with Agent**[c]		
AdalimumabAzathioprine	0.72 (0.51 to 1.02)	very low ⊕⊖⊖⊖	642 per 1,000	462 per 1,000 (327 to 655)	18% less (31.5% less to 1.3% more)	NA	The evidence is very uncertain
Infliximab	0.85 (0.6 to 1.19)	very low ⊕⊖⊖⊖	642 per 1,000	546 per 1,000 (385 to 764)	9.6% less (25.7% less to 12.2% more)	NA	The evidence is very uncertain
CTP13Azathioprine	0.79 (0.49 to 1.25)	very low ⊕⊖⊖⊖	642 per 1,000	507 per 1,000 (315 to 803)	13.5% less (32.7% less to 16.1% more)	NA	The evidence is very uncertain
NatalizumabInfliximabAzathioprine	0.82 (0.54 to 1.25)	very low ⊕⊖⊖⊖	642 per 1,000	526 per 1,000 (347 to 803)	11.6% less (29.5% less to 16.1% more)	NA	The evidence is very uncertain
InfliximabAzathioprine	0.89 (0.62 to 1.28)	very low ⊕⊖⊖⊖	642 per 1,000	571 per 1,000 (398 to 822)	7.1% less (24.4% less to 18% more)	NA	The evidence is very uncertain
Risankizumab	0.91 (0.79 to 1.05)	Low	642 per 1,000	584 per 1,000 (507 to 674)	5.8% less (13.5% less to 3.2% more)	NA	It maybe leads to similar total adverse events as

		⊕⊕⊖⊖					placebo. Ranging from small effect size less total events to trivially more.
Mirikizumab	0.91 (0.68 to 1.21)	Low<br>⊕⊕⊖⊖	642 per 1,000	584 per 1,000 (437 to 777)	5.8% less (20.5% less to 13.5% more)	NA	It maybe leads to similar total adverse events as placebo. Ranging from moderate effect less total events to small effect size more.
Purine analogues	0.91 (0.69 to 1.2)	very low<br>⊕⊖⊖⊖	642 per 1,000	584 per 1,000 (443 to 770)	5.8% less (19.9% less to 12.8% more)	NA	The evidence is very uncertain
Tofacitinib	0.95 (0.76 to 1.19)	Low<br>⊕⊕⊖⊖	642 per 1,000	610 per 1,000 (488 to 764)	3.2% less (15.4% less to 12.2% more)	NA	It maybe leads to similar total adverse events as placebo. Ranging from small effect less total events to small effect size more.
Apilimod Mesylate	0.94 (0.73 to 1.21)	Low<br>⊕⊕⊖⊖	642 per 1,000	603 per 1,000 (469 to 777)	3.9% less (17.3% less to 13.5% more)	NA	It maybe leads to similar total adverse events as placebo. Ranging from moderate effect less total events to small effect size more.
Andecaliximab	0.95 (0.67 to 1.34)	very low<br>⊕⊖⊖⊖	642 per 1,000	610 per 1,000 (430 to 860)	3.2% less (21.2% less to 21.8% more)	NA	The evidence is very uncertain
Adalimumab	0.97 (0.91 to 1.04)	Low<br>⊕⊕⊖⊖	642 per 1,000	655 per 1,000 (552 to 770)	1.3% less (9% less to 12.8% more)	NA	It maybe leads to similar total adverse events as placebo. Ranging from small effect less total events to small effect more.

Ustekinumab	1.03 (0.94 to 1.12)	High<br>⊕⊕⊕⊕	642 per 1,000	661 per 1,000 (603 to 719)	1.9% less (3.9% less to 7.7% more)	NA	It leads to similar total adverse events as placebo. Ranging from trivially less total events to trivially more.
Natalizumab	1.02 (0.89 to 1.16)	Moderate<br>⊕⊕⊕⊖	642 per 1,000	655 per 1,000 (571 to 745)	1.3% more 7.1% less to 10.3% more)	NA	It probably leads to similar total adverse events as placebo. Ranging from trivially less total events to small effect size more.
Vedolizumab	1.04 (0.92 to 1.19)	Low<br>⊕⊕⊖⊖	642 per 1,000	668 per 1,000 (591 to 764)	2.6% more (5.1% less to 12.2% more)	NA	It maybe leads to similar total adverse events as placebo. Ranging from trivially less total events to small effect size more.
Onercept	1.05 (0.76 to 1.43)	Very low<br>⊕⊖⊖⊖	642 per 1,000	674 per 1,000 (488 to 918)	3.2% more (15.4% less to 27.6% more)	NA	The evidence is very uncertain
Certolizumab	1.05 (0.92 to 1.21)	Low<br>⊕⊕⊖⊖	642 per 1,000	674 per 1,000 (591 to 777)	3.2% more (5.1% less to 13.5% more)	NA	It maybe leads to similar total adverse events as placebo. Ranging from trivially less total events to small effect more.
Guselkumab	1.05 (0.91 to 1.22)	very low<br>⊕⊖⊖⊖	642 per 1,000	674 per 1,000 (584 to 783)	3.2% less (5.8% less to 14.1% more)	NA	The evidence is very uncertain
Upadacitinib	1.07 (0.92 to 1.25)	Very low<br>⊕⊖⊖⊖	642 per 1,000	687 per 1,000 (591 to 803)	4.5% more (5.1% less to 16.1% more)	NA	The evidence is very uncertain
BI695501	1.13 (0.78 to 1.66)	Very low	642 per 1,000			NA	

		⊕⊖⊖⊖		725 per 1,000 (501 to 1000)	8.3% more (14.1% less to 42.4% more)		The evidence is very uncertain
Filgotinib	1.15 (1 to 1.33)	Low<br>⊕⊕⊖⊖	642 per 1,000	738 per 1,000 (642 to 854)	9.6% less (0% to 21.2% more)	NA	It maybe leads to similar total adverse events as placebo. Ranging from the same total events as placebo to moderate effect size more.
Etrolizumab	1.22 (0.95 to 1.57)	Low<br>⊕⊕⊖⊖	642 per 1,000	783 per 1,000 (610 to 1000)	14.1% more (3.2% less to 36.6% more)	NA	It maybe leads to similar total adverse events as placebo. Ranging from trivially less total events to large effect size more.
Duvakitug	2.5 (0.3 to 20.99)	Very low<br>⊕⊖⊖⊖	642 per 1,000	1,000 per 1,000 (193 to 1,000)	96.3% more (44.9% less to 100% more)	NA	The evidence is very uncertain
Etanercept	1.48 (0.86 to 2.54)	Very low<br>⊕⊖⊖⊖	642 per 1,000	950 per 1,000 (552 to 1000)	30.8% more (9% less to 98.9% more)	NA	The evidence is very uncertain
Tesnatilimab	1.54 (1.19 to 1.99)	Low<br>⊕⊕⊖⊖	642 per 1,000	989 per 1,000 (764 to 1000)	34.7% more (12.2% more to 63.6% more)	NA	It maybe leads to a large effect size more total adverse events as placebo. Ranging from small effect size less total events to large effect size more.
Methotrexate	2.17 (1.03 to 4.6)	Very low<br>⊕⊖⊖⊖	642 per 1,000	1000 per 1,000 (661 to 1000)	75.1% more (1.9% more to 100% more)	NA	The evidence is very uncertain

GRADE Working Group grades of evidence

High certainty: we are very confident that the true effect lies close to that of the estimate of the effect.

Moderate certainty: we are moderately confident in the effect estimate; the true effect is likely to be close to the estimate of the effect, but there is a possibility that it is substantially different.

Low certainty: our confidence in the effect estimate is limited; the true effect may be substantially different from the estimate of the effect.

Very low certainty: we have very little confidence in the effect estimate; the true effect is likely to be substantially different from the estimate of effect.

CI: confidence interval; RR: risk ratio

[a] The risk with placebo has been calculated based on the cumulative placebo rates of all studies with a placebo arm.
[b]The risk with treatment has been calculated by multiplying the risk with control with the RR(95% CI). If the calculation results in more than 1000 per 1000 people the number has been capped to 1000. Numbers have been rounded up to the closest whole number.
[c]The % risk difference has been calculated by subtracting the risk with control from the risk with treatment(95% CI) and dividing by 10. If the calculation results in more than 100% the number has been capped to 100%. Numbers have been rounded up to the closest whole number.
*red colouring indicates the treatment crosses the line of no effect

**Sucra**	**Intervention (n=29)**	**network estimate RR**	**lower 95%CI**	**higher 95% CI**	**Direct studies**	**Direct GRADE**	**Reasons for direct downgrade**	**Indirect GRADE**	**Reasons for indirect downgrade**	**Network GRADE**	**Reasons for network downgrade**
1	5-ASA	n/a	n/a	n/a	n/a	n/a	n/a	n/a	n/a	n/a	n/a
2	AdalimumabAzathioprine	1.02	0.86	1.2	0	x	x	Low	twice rob in the loop	**very low**	once imprecision
3	Infliximab	0.85	0.6	1.19	1	low	twice rob	moderate	once rob in loop	**very low**	twice imprecision, twice incoherence
4	CTP13Azathioprine	0.79	0.49	1.25	0	x	x	moderate	once rob in loop	**very low**	twice imprecision
5	NatalizumabInfliximabAzathioprine	0.82	0.54	1.25	0	x	x	Low	twice rob in the loop	**very low**	twice imprecision
6	InfliximabAzathioprine	0.89	0.62	1.28	0	x	x	moderate	once rob in the loop	**very low**	twice imprecision
7	Risankizumab	0.91	0.79	1.05	3	low	twice inconsistency	x	x	**low**	twice imprecision
8	Mirikizumab	0.91	0.68	1.21	1	high	none	x	x	**low**	twice imprecision
9	Purine analogues	0.91	0.69	1.2	1	moderate	once RoB	x	x	**very low**	twice imprecision, once incoherence
10	Tofacitinib	0.95	0.76	1.19	2	high	none	x	x	**low**	twice imprecision

11	Apilimod Mesylate	0.94	0.73	1.21	1	high	none	x	x	**low**	twice imprecision
12	Andecaliximab	0.95	0.67	1.34	1	moderate	once rob	x	x	**very low**	twice imprecision
13	Placebo	1	n/a	n/a	n/a	n/a	n/a	n/a	n/a	n/a	n/a
14	Adalimumab	1.02	0.86	1.2	2	moderate	once RoB	moderate	once rob in the loop	**low**	twice imprecicion
15	Ustekinumab	1.03	0.94	1.12	7	moderate	once inconsistency	moderate	once inconsistency in the loop	**moderate**	none
16	Natalizumab	1.02	0.89	1.16	3	moderate	once rob	x	x	**moderate**	none
17	Vedolizumab	1.04	0.92	1.19	5	moderate	once RoB	x	x	**low**	once imprecision
18	Onercept	1.05	0.76	1.43	1	moderate	once rob	x	x	**very low**	twice imprecision
19	Certolizumab	1.05	0.92	1.21	4	moderate	once rob	x	x	**low**	once imprecicion
20	Guselkumab	1.05	0.91	1.22	3	very low	twice inconsistency, once RoB	very low	twice inconsistency in loop, once RoB	**very low**	once imprecision, once incoherence
21	Upadacitinib	1.07	0.92	1.25	3	moderate	once RoB	x	x	**very low**	twice imprecision
22	BI695501	1.13	0.78	1.66	0	x	x	moderate	once rob in loop	**very low**	twice imprecision
23	Filgotinib	1.15	1	1.33	3	low	twice inconsistency	x	x	**very low**	twice imprecision
24	Etrolizumab	1.22	0.95	1.57	1	high	none	x	x	**low**	twice imprecision
25	Duvakitug	2.5	0.3	20.99	1	low	twice RoB	x	x	**very low**	twice imprecision
26	Etanercept	1.48	0.86	2.54	1	moderate	once rob	x	x	**very low**	twice imprecision
27	Tesnatilimab	1.54	1.19	1.99	2	high	none	x	x	**low**	twice incoherence
28	Methotrexate	2.17	1.03	4.6	0	x	x	low	twice rob in the loop	**very low**	once imprecicion
29	Everolimus	n/a	n/a	n/a	n/a	n/a	n/a	n/a	n/a	n/a	n/a

eFigures1. Network plots

Network plot for induction of clinical remission

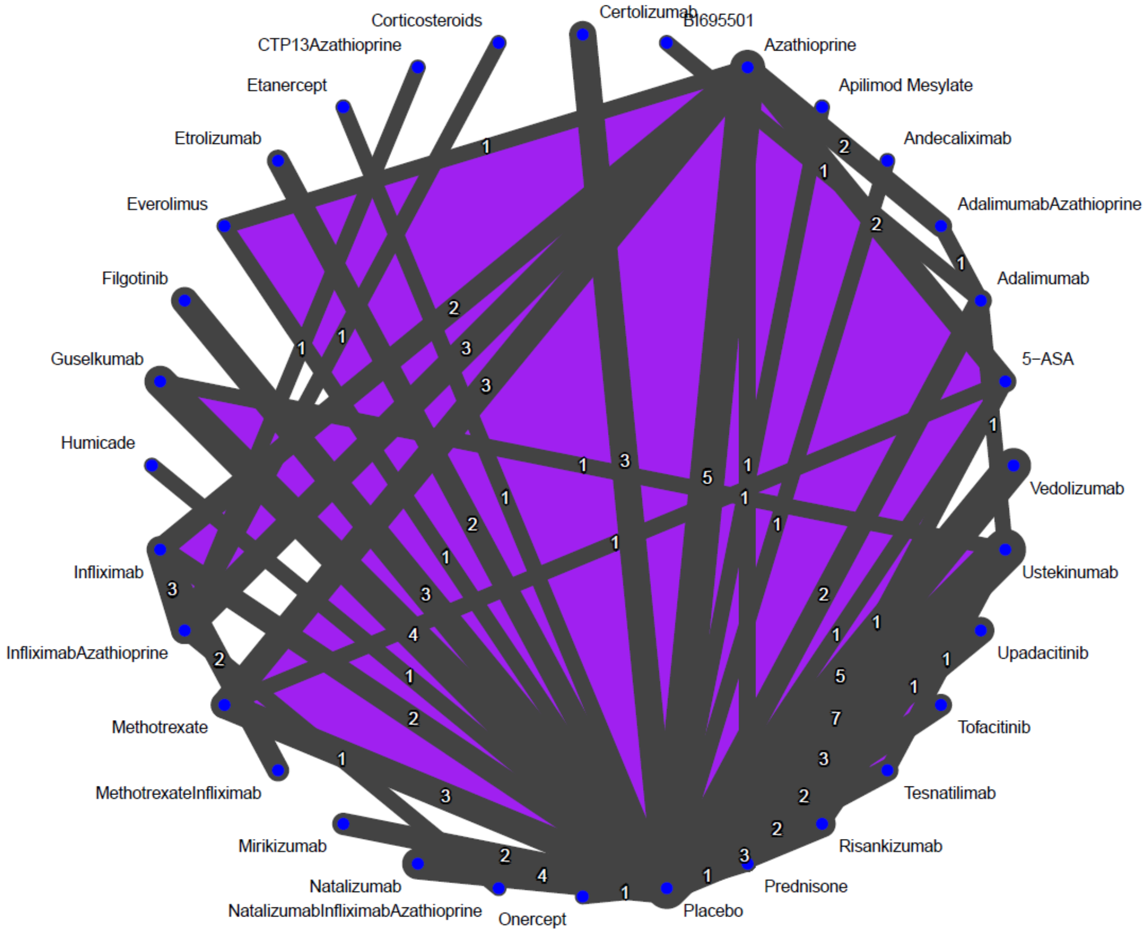

Network plot for induction of clinical response

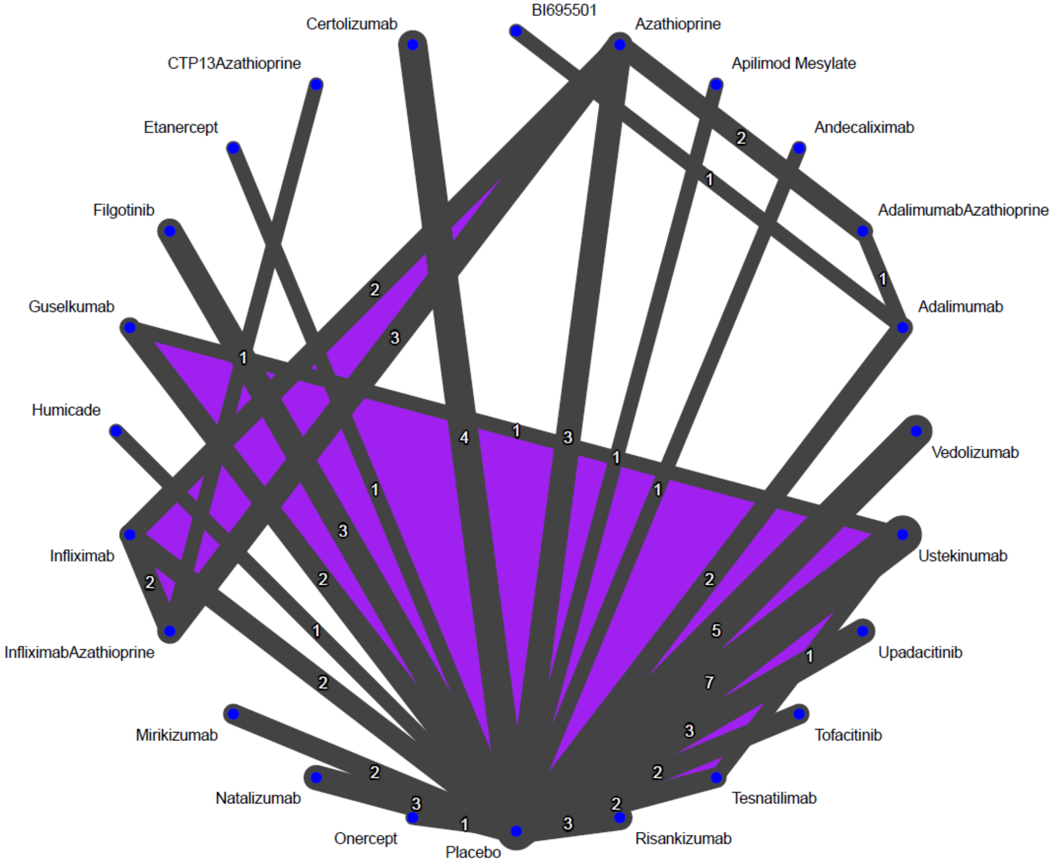

Network plot for induction of Endoscopic remission.

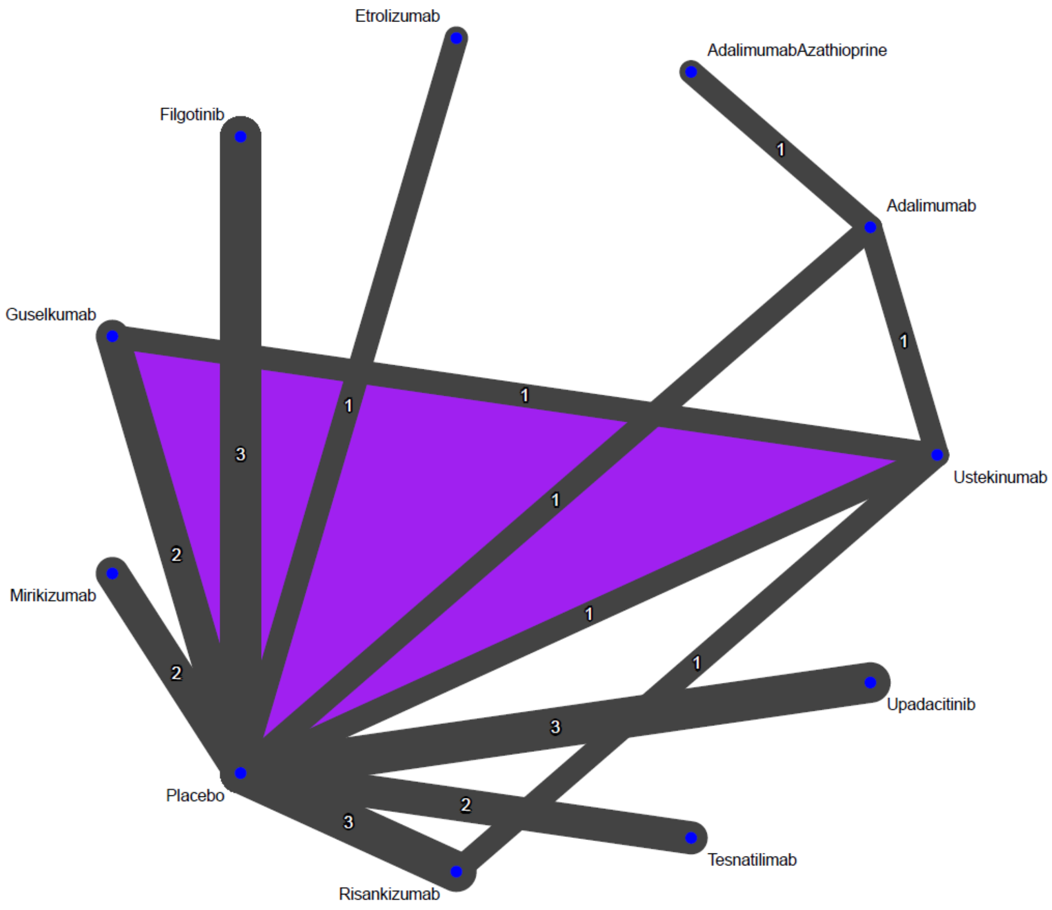

Network plot for withdrawals due to adverse events

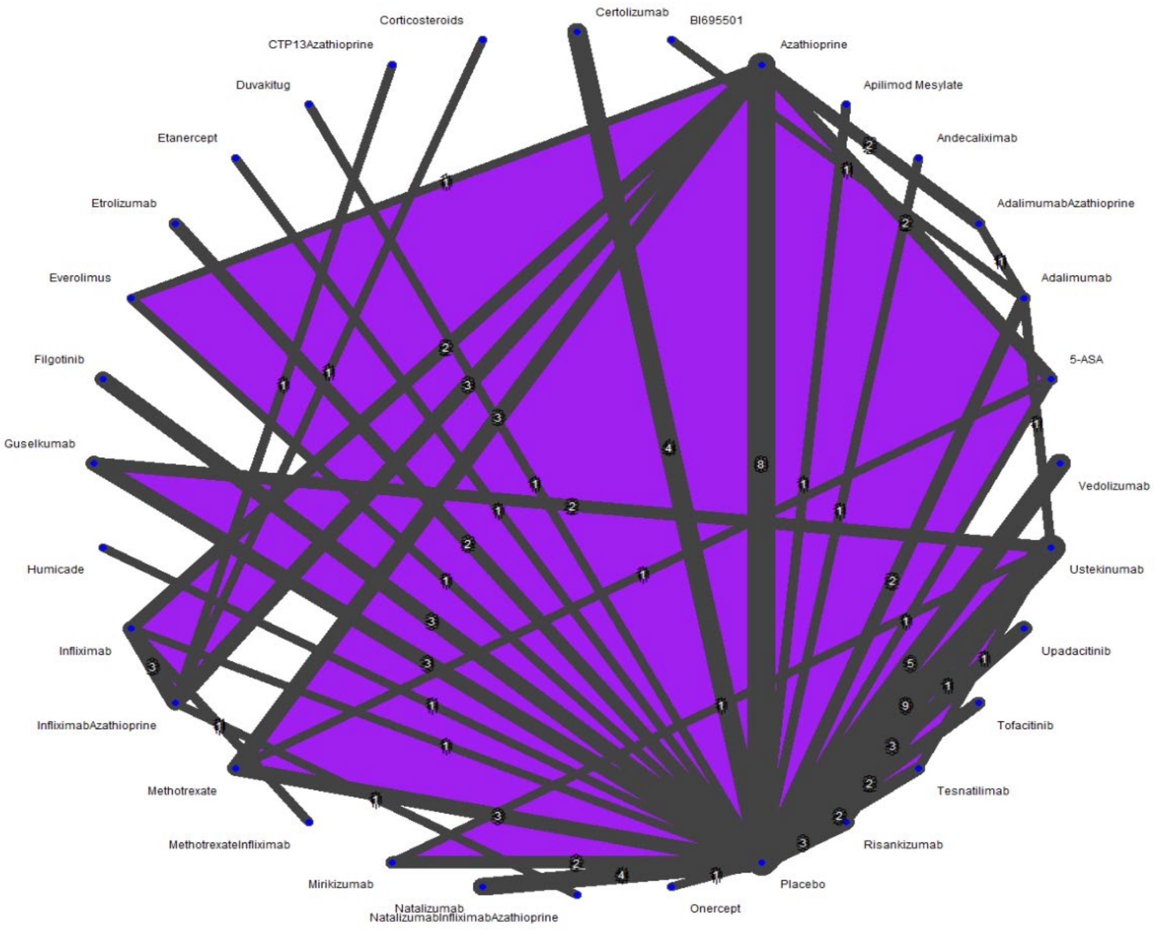

Network plot for serious adverse events

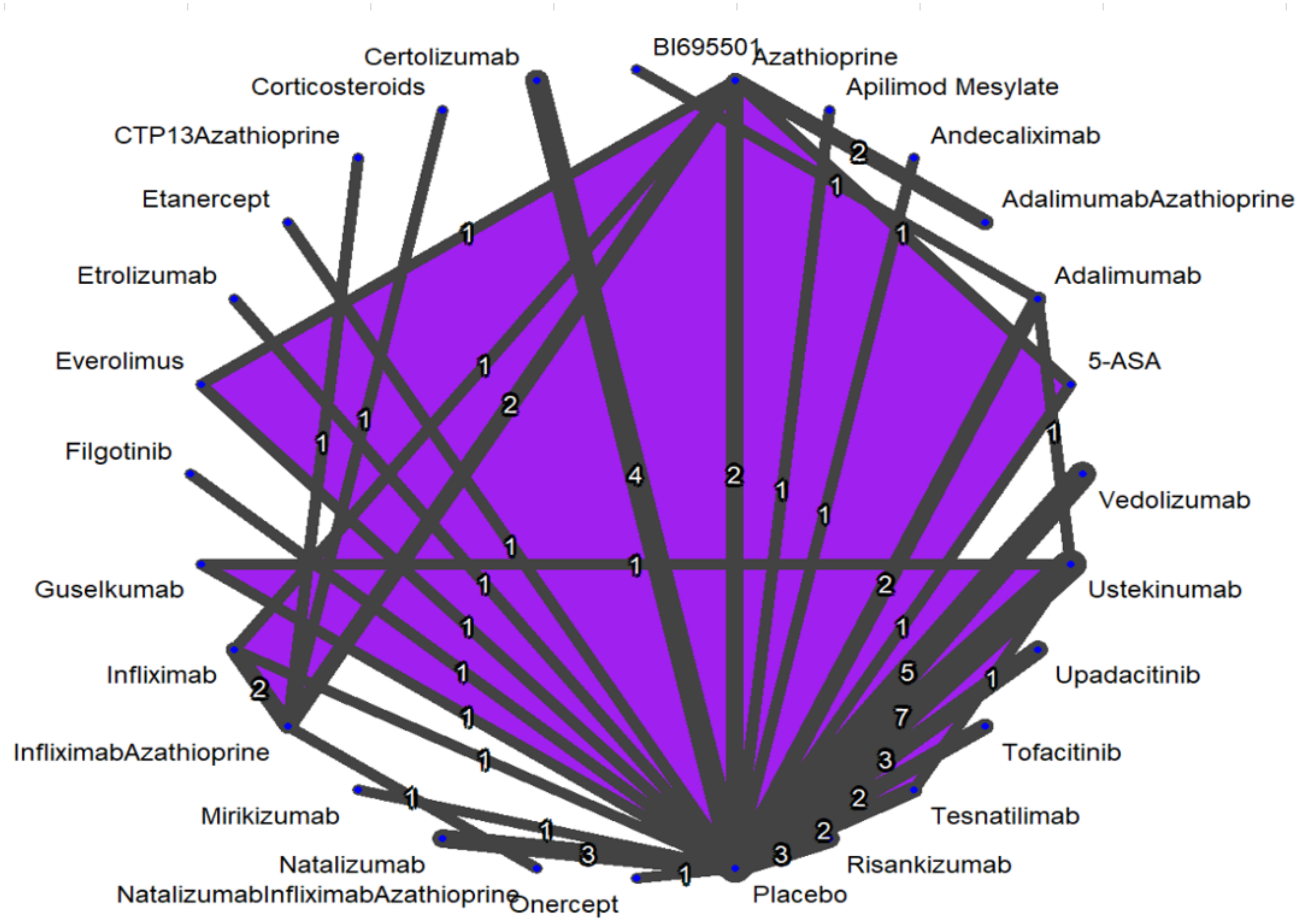

Network plot for total adverse events

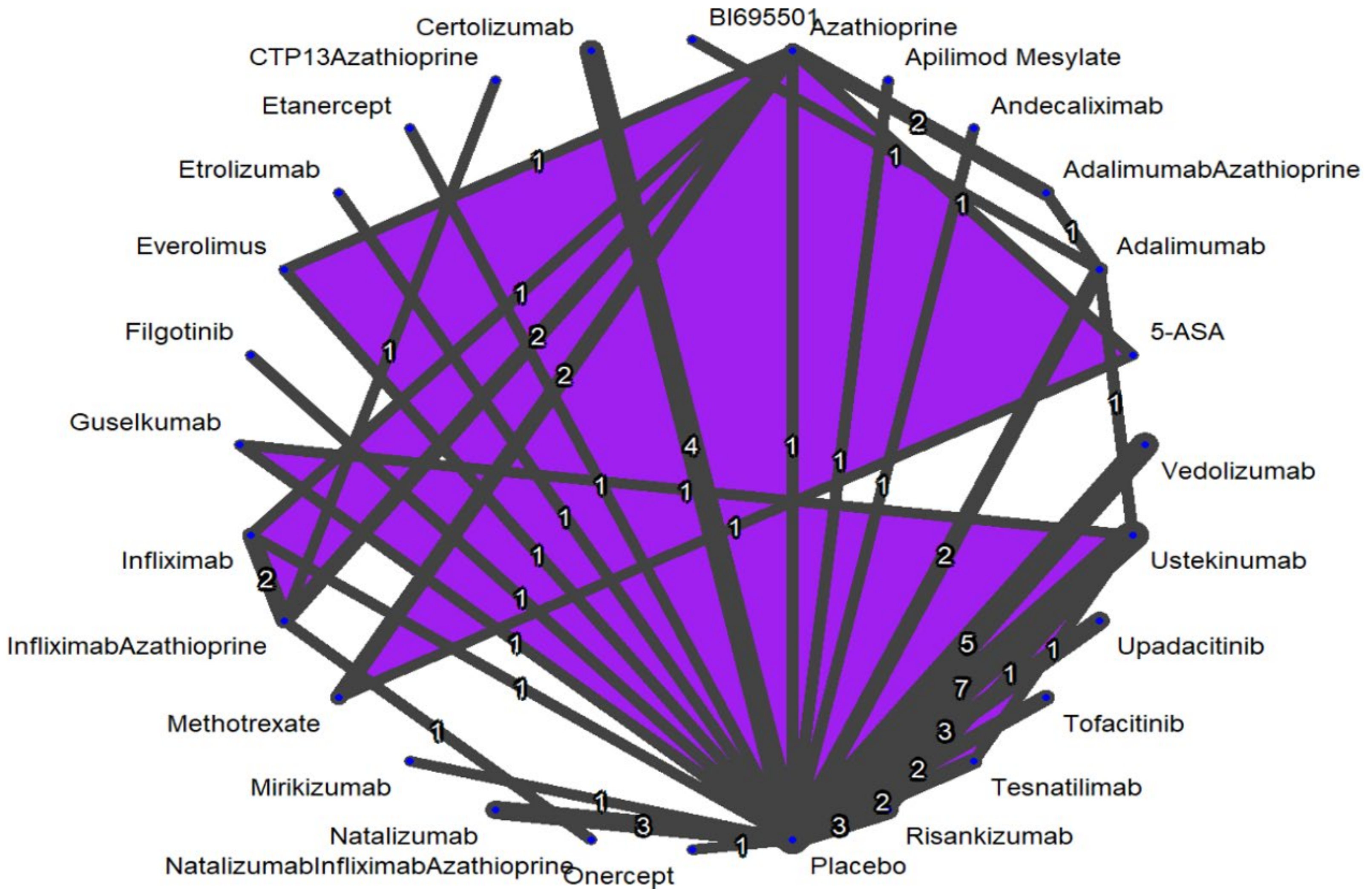

eFigures 2. NETWORK FOREST PLOTS, SUCRA PROBABILITIES, AND DIRECT/INDIRECT/NETWORK ESTIMATES FOREST PLOTS

Network forest plot Clinical Remission

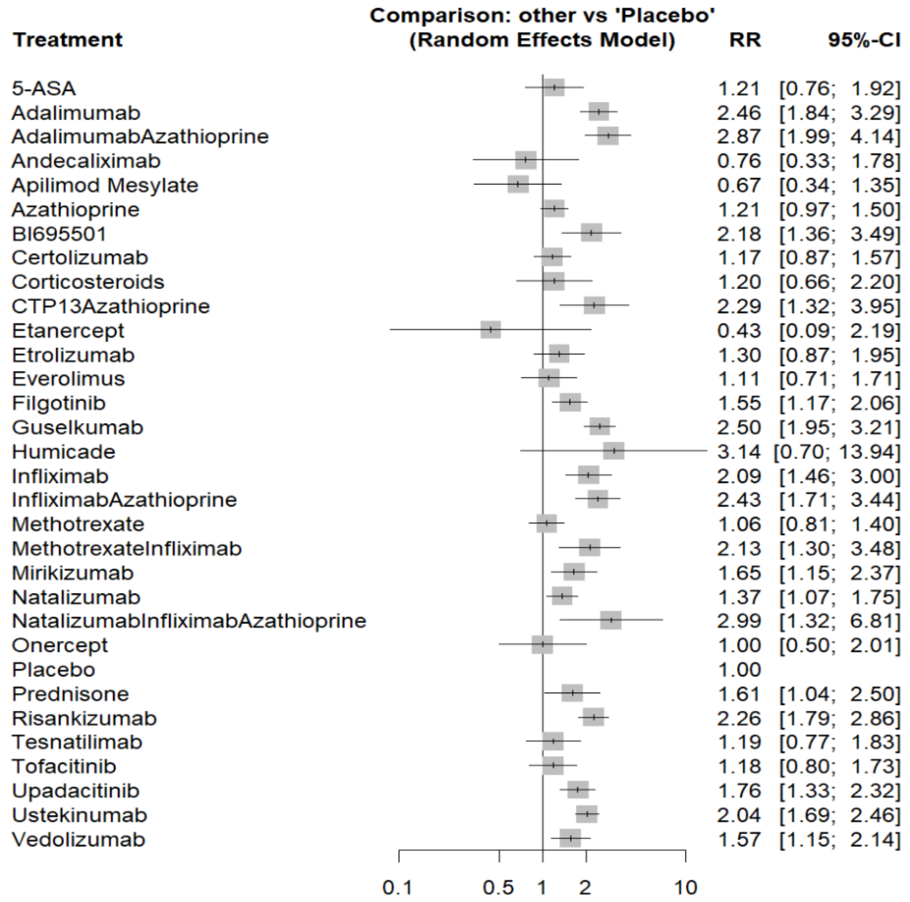

SUCRA probabilities Clinical Remission

	SUCRA (common)	SUCRA (random)
AdalimumabAzathioprine	0.8584	0.9132
NatalizumabInfliximabAzathioprine	0.8816	0.8868
Guselkumab	0.9039	0.8465
Adalimumab	0.8394	0.8442
Humicade	0.8110	0.8355
InfliximabAzathioprine	0.8513	0.8032
Risankizumab	0.8290	0.7926
CTP13Azathioprine	0.7919	0.7735
BI695501	0.7490	0.7516
MethotrexateInfliximab	0.7232	0.7461
Infliximab	0.6813	0.7194
Ustekinumab	0.7200	0.7132
Upadacitinib	0.6303	0.6052
Mirikizumab	0.5390	0.5658
Vedolizumab	0.5119	0.5258
Prednisone	0.5587	0.5194
Filgotinib	0.5006	0.4819
Natalizumab	0.4171	0.4077
Etrolizumab	0.3565	0.3290
5-ASA	0.3116	0.3123
Corticosteroids	0.2300	0.3103
Azathioprine	0.2810	0.3039
Tesnatilimab	0.2523	0.3003
Certolizumab	0.3077	0.2826
Tofacitinib	0.3155	0.2713
Onercept	0.2284	0.2497
Everolimus	0.2410	0.2348
Methotrexate	0.1610	0.2035
Placebo	0.1671	0.1629
Andecaliximab	0.1468	0.1465
Etanercept	0.1252	0.0868
Apilimod Mesylate	0.0784	0.0745

- based on 100 simulations

Direct/indirect/network estimates forest plot Clinical Remission

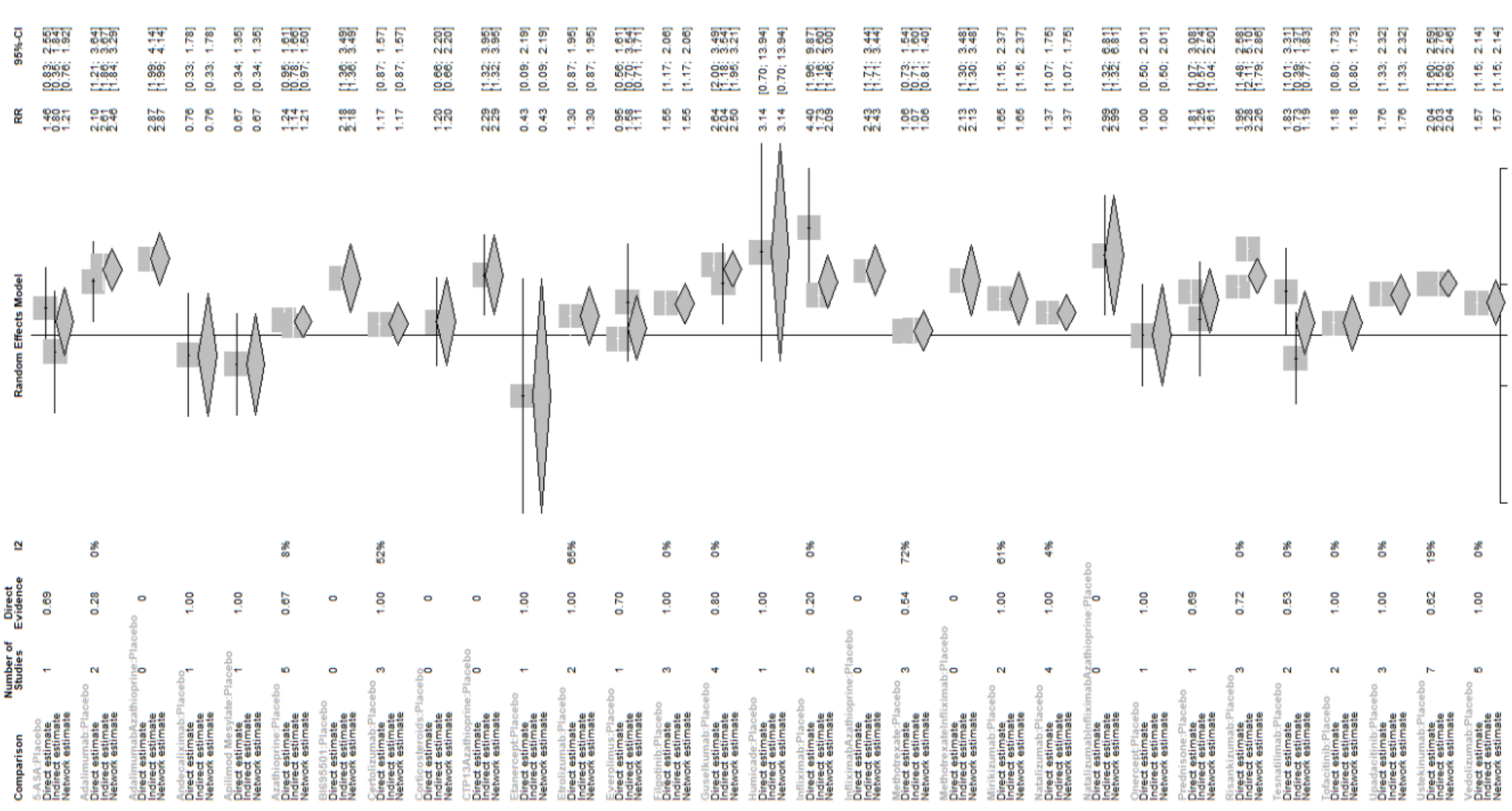

Network forest plot Clinical Response

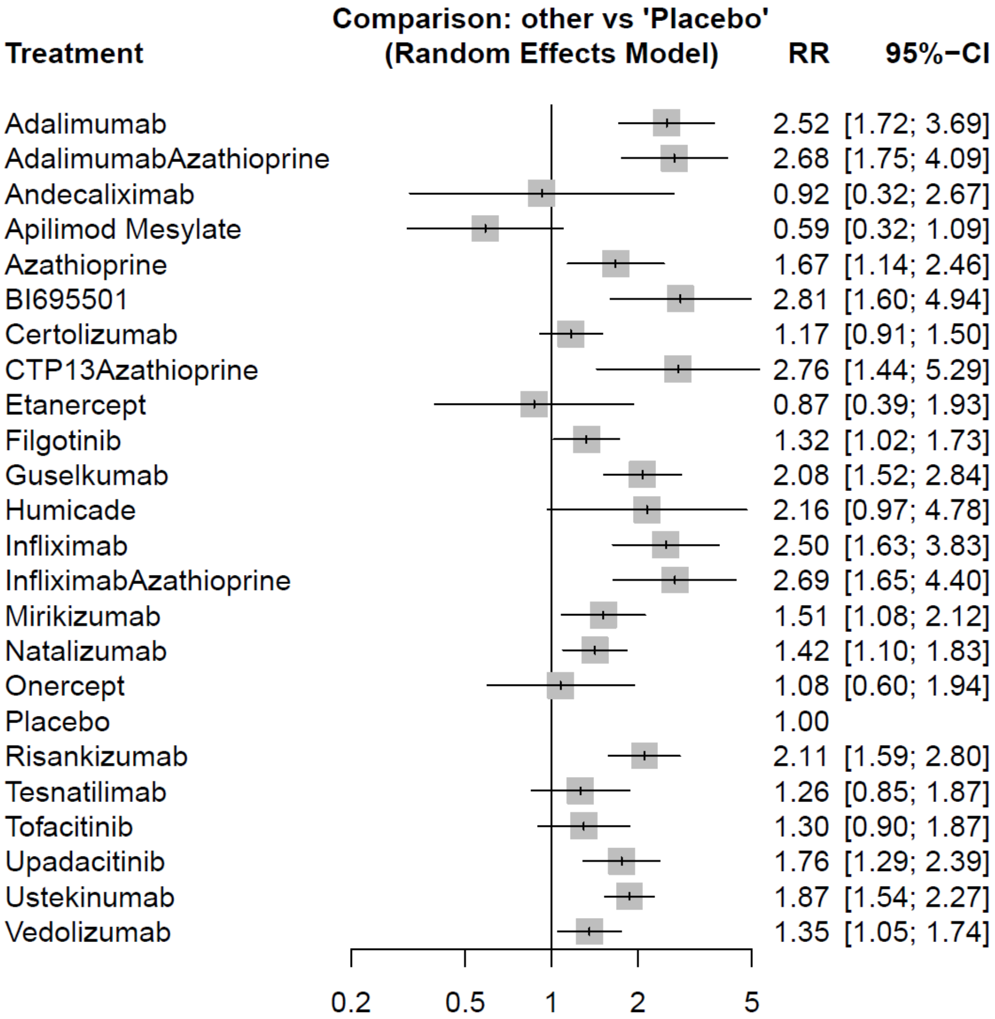

SUCRA probabilities Clinical Response

	SUCRA (common)	SUCRA (random)
CTP13Azathioprine	0.8457	0.8770
AdalimumabAzathioprine	0.8048	0.8678
InfliximabAzathioprine	0.8265	0.8591
BI695501	0.9470	0.8470
Adalimumab	0.8878	0.8426
Infliximab	0.7678	0.8143
Risankizumab	0.7204	0.7235
Guselkumab	0.7261	0.6717
Humicade	0.7513	0.6491
Ustekinumab	0.6643	0.6122
Upadacitinib	0.5783	0.5800
Azathioprine	0.3983	0.5170
Mirikizumab	0.4187	0.4309
Natalizumab	0.3761	0.4109
Vedolizumab	0.4291	0.3670
Filgotinib	0.3404	0.3300
Tofacitinib	0.3448	0.3148
Tesnatilimab	0.2300	0.3087
Certolizumab	0.2443	0.2426
Andecaliximab	0.2235	0.2230
Onercept	0.1917	0.2074
Etanercept	0.1322	0.1413
Placebo	0.1261	0.1339
Apilimod Mesylate	0.0248	0.0283

- based on 100 simulations

Direct/indirect/network estimates forest plot Clinical Response

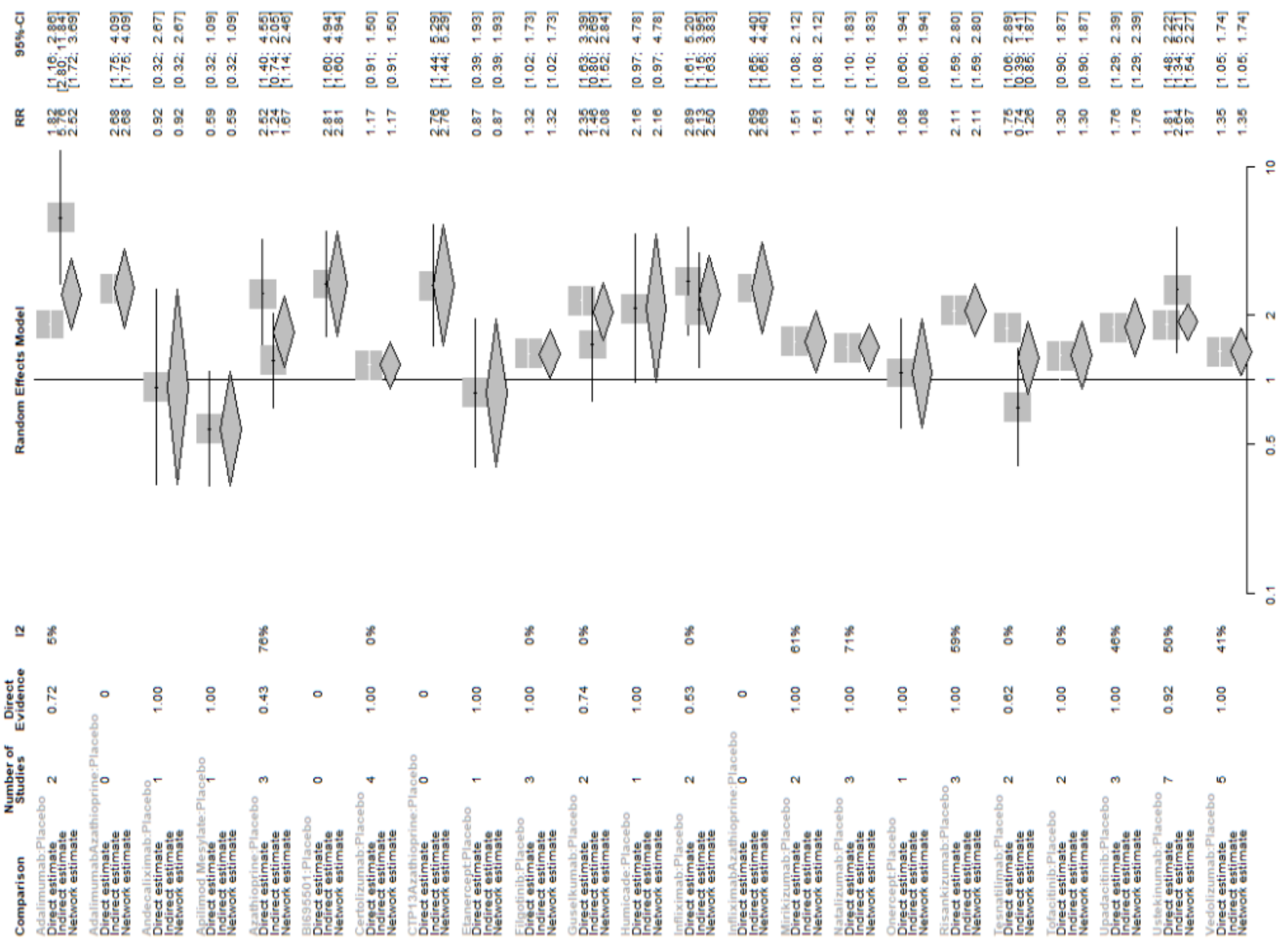

Network forest plot Endoscopic Remission

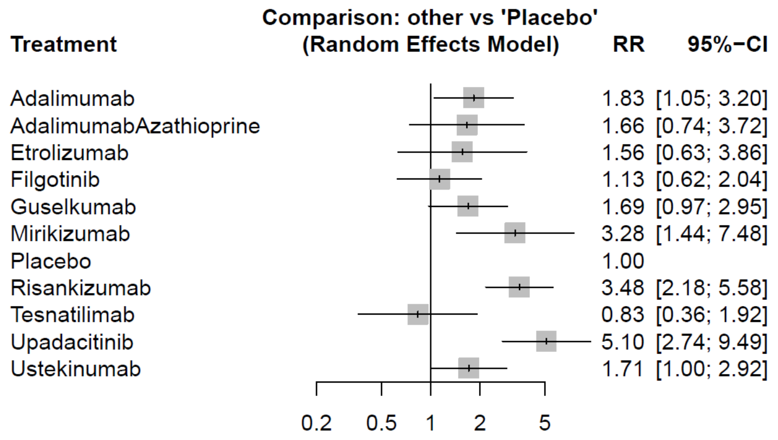

SUCRA probabilities Endoscopic Remission

	SUCRA (common)	SUCRA (random)
Upadacitinib	0.967	0.948
Risankizumab	0.864	0.854
Mirikizumab	0.820	0.808
Adalimumab	0.567	0.551
Ustekinumab	0.475	0.498
AdalimumabAzathioprine	0.438	0.480
Guselkumab	0.510	0.478
Etrolizumab	0.455	0.396
Filgotinib	0.222	0.208
Placebo	0.100	0.145
Tesnatilimab	0.082	0.134

- based on 100 simulations

Direct/indirect/network estimates forest plot Endoscopic Remission

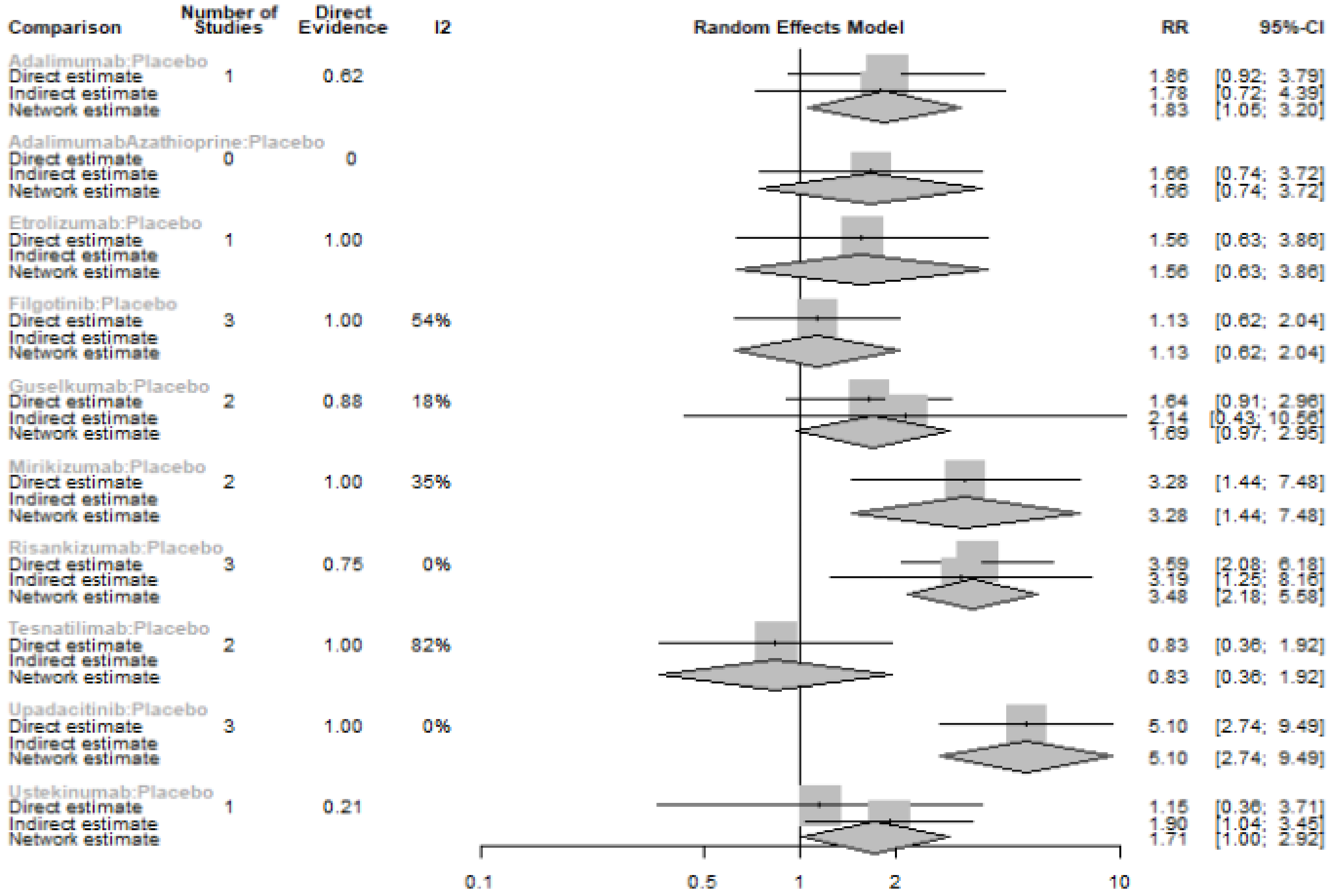

Network forest plot Withdrawals due to adverse events

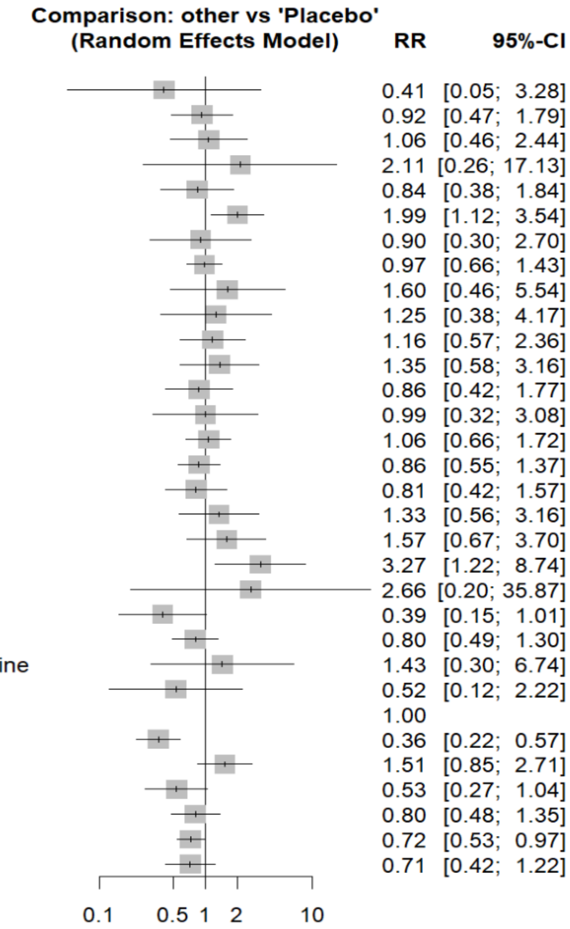

Treatment	RR	95%-CI
5-ASA	0.41	[0.05; 3.28]
Adalimumab	0.92	[0.47; 1.79]
AdalimumabAzathioprine	1.06	[0.46; 2.44]
Andecaliximab	2.11	[0.26; 17.13]
Apilimod Mesylate	0.84	[0.38; 1.84]
Azathioprine	1.99	[1.12; 3.54]
BI695501	0.90	[0.30; 2.70]
Certolizumab	0.97	[0.66; 1.43]
Corticosteroids	1.60	[0.46; 5.54]
CTP13Azathioprine	1.25	[0.38; 4.17]
Duvakitug	1.16	[0.57; 2.36]
Etanercept	1.35	[0.58; 3.16]
Etrolizumab	0.86	[0.42; 1.77]
Everolimus	0.99	[0.32; 3.08]
Filgotinib	1.06	[0.66; 1.72]
Guselkumab	0.86	[0.55; 1.37]
Humicade	0.81	[0.42; 1.57]
Infliximab	1.33	[0.56; 3.16]
InfliximabAzathioprine	1.57	[0.67; 3.70]
Methotrexate	3.27	[1.22; 8.74]
MethotrexateInfliximab	2.66	[0.20; 35.87]
Mirikizumab	0.39	[0.15; 1.01]
Natalizumab	0.80	[0.49; 1.30]
NatalizumabInfliximabAzathioprine	1.43	[0.30; 6.74]
Onercept	0.52	[0.12; 2.22]
Placebo	1.00	
Risankizumab	0.36	[0.22; 0.57]
Tesnatilimab	1.51	[0.85; 2.71]
Tofacitinib	0.53	[0.27; 1.04]
Upadacitinib	0.80	[0.48; 1.35]
Ustekinumab	0.72	[0.53; 0.97]
Vedolizumab	0.71	[0.42; 1.22]

SUCRA probabilities Withdrawals due to adverse events

	SUCRA (common)	SUCRA (random)
Risankizumab	0.9468	0.9397
Mirikizumab	0.9142	0.8684
Tofacitinib	0.8410	0.8287
5-ASA	0.8048	0.7935
Onercept	0.7948	0.7223
Ustekinumab	0.7174	0.6984
Vedolizumab	0.6642	0.6577
Upadacitinib	0.6490	0.6526
Natalizumab	0.6310	0.6361
Humicade	0.6613	0.6155
Apilimod Mesylate	0.6126	0.6129
BI695501	0.5329	0.5926
Guselkumab	0.6390	0.5874
Adalimumab	0.5300	0.5816
Etrolizumab	0.6326	0.5777
Everolimus	0.5455	0.5032
Certolizumab	0.5894	0.4987
Placebo	0.4810	0.4681
AdalimumabAzathioprine	0.3906	0.4603
Filgotinib	0.4132	0.4484
CTP13Azathioprine	0.3390	0.4045
Duvakitug	0.3871	0.3890
NatalizumabInfliximabAzathioprine	0.3232	0.3671
Infliximab	0.3029	0.3416
Etanercept	0.3458	0.3206
Tesnatilimab	0.2306	0.2603
Corticosteroids	0.2094	0.2590
Andecaliximab	0.2790	0.2584
InfliximabAzathioprine	0.1990	0.2287
MethotrexateInfliximab	0.2084	0.2268
Azathioprine	0.1255	0.1406
Methotrexate	0.0587	0.0594

- based on 100 simulations

Direct/indirect/network estimates forest plot Withdrawals due to Adverse Events

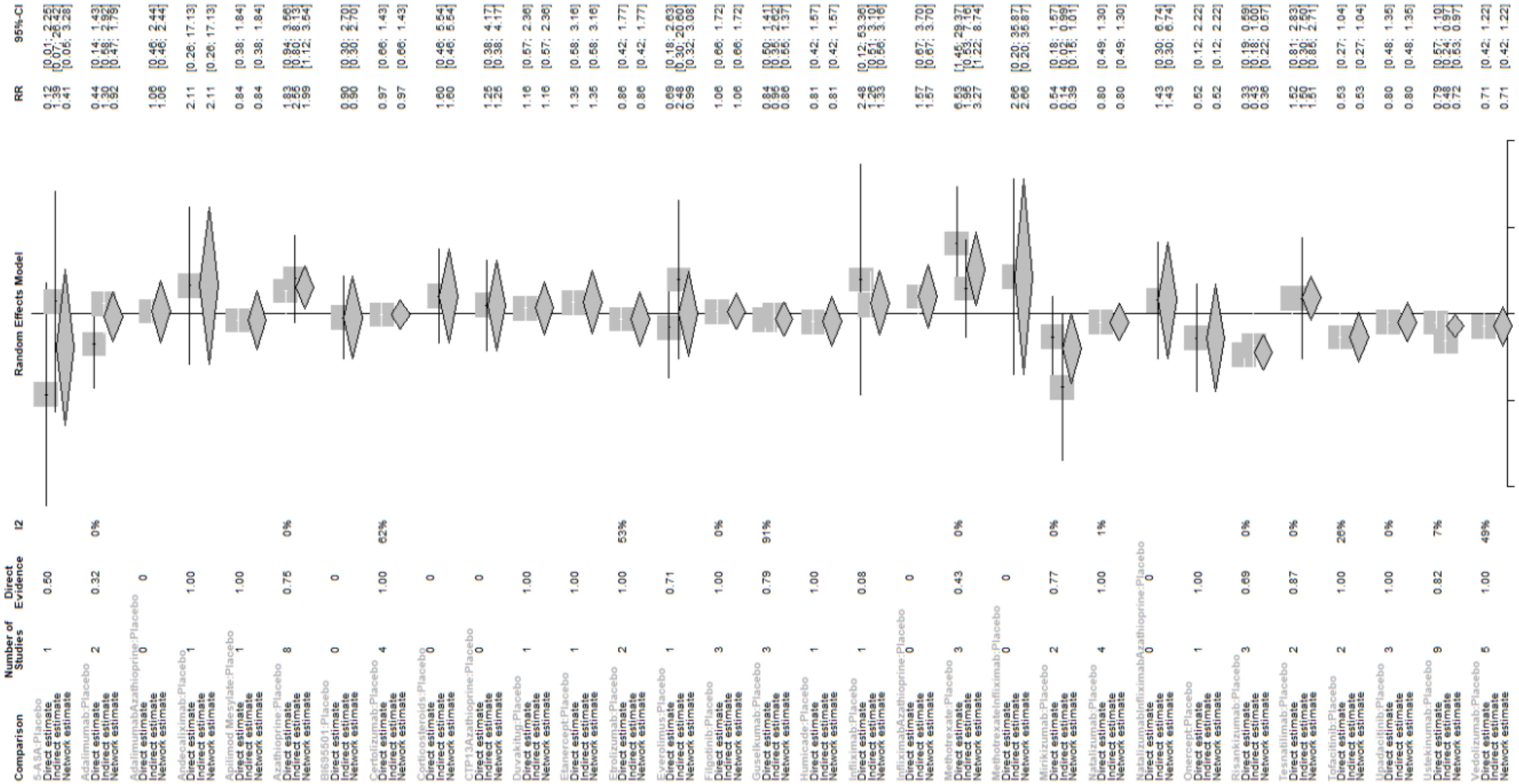

Network forest plot Serious Adverse Events

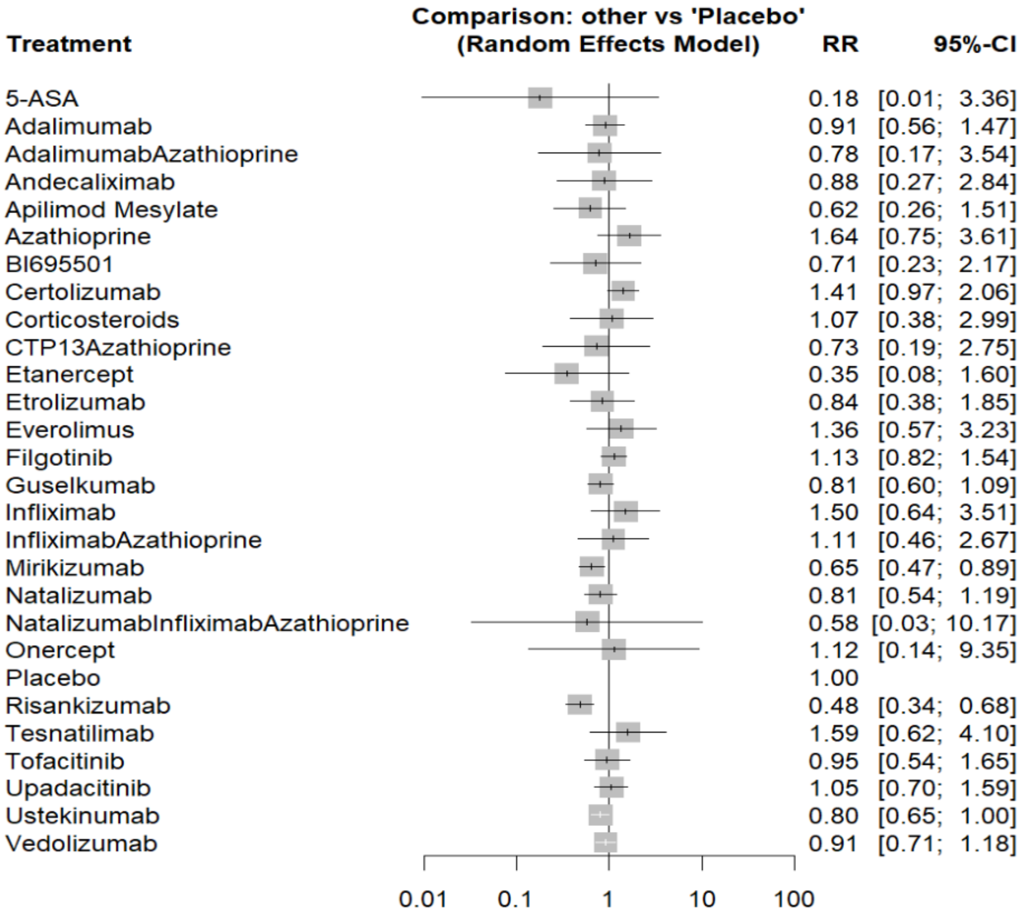

SUCRA probabilities Serious Adverse Events

	SUCRA (common)	SUCRA (random)
5-ASA	0.8441	0.8674
Risankizumab	0.8559	0.8548
Etanercept	0.8048	0.8352
Apilimod Mesylate	0.6781	0.7393
Mirikizumab	0.7607	0.7137
CTP13Azathioprine	0.5941	0.6648
Ustekinumab	0.5981	0.6241
NatalizumabInfliximabAzathioprine	0.6481	0.6096
AdalimumabAzathioprine	0.5100	0.6052
BI695501	0.6093	0.5948
Guselkumab	0.5981	0.5881
Natalizumab	0.5719	0.5759
Etrolizumab	0.5589	0.5522
Andecaliximab	0.4907	0.5281
Adalimumab	0.5081	0.5007
Tofacitinib	0.4385	0.4889
Vedolizumab	0.4752	0.4756
Placebo	0.4233	0.4215
Upadacitinib	0.3841	0.3867
Filgotinib	0.3496	0.3637
Onercept	0.3989	0.3596
InfliximabAzathioprine	0.4119	0.3515
Corticosteroids	0.4333	0.3493
Everolimus	0.2600	0.2496
Infliximab	0.2107	0.1904
Tesnatilimab	0.2144	0.1856
Certolizumab	0.2237	0.1848
Azathioprine	0.1452	0.1389

- based on 100 simulations

Direct/indirect/network estimates forest plot Serious Adverse Events

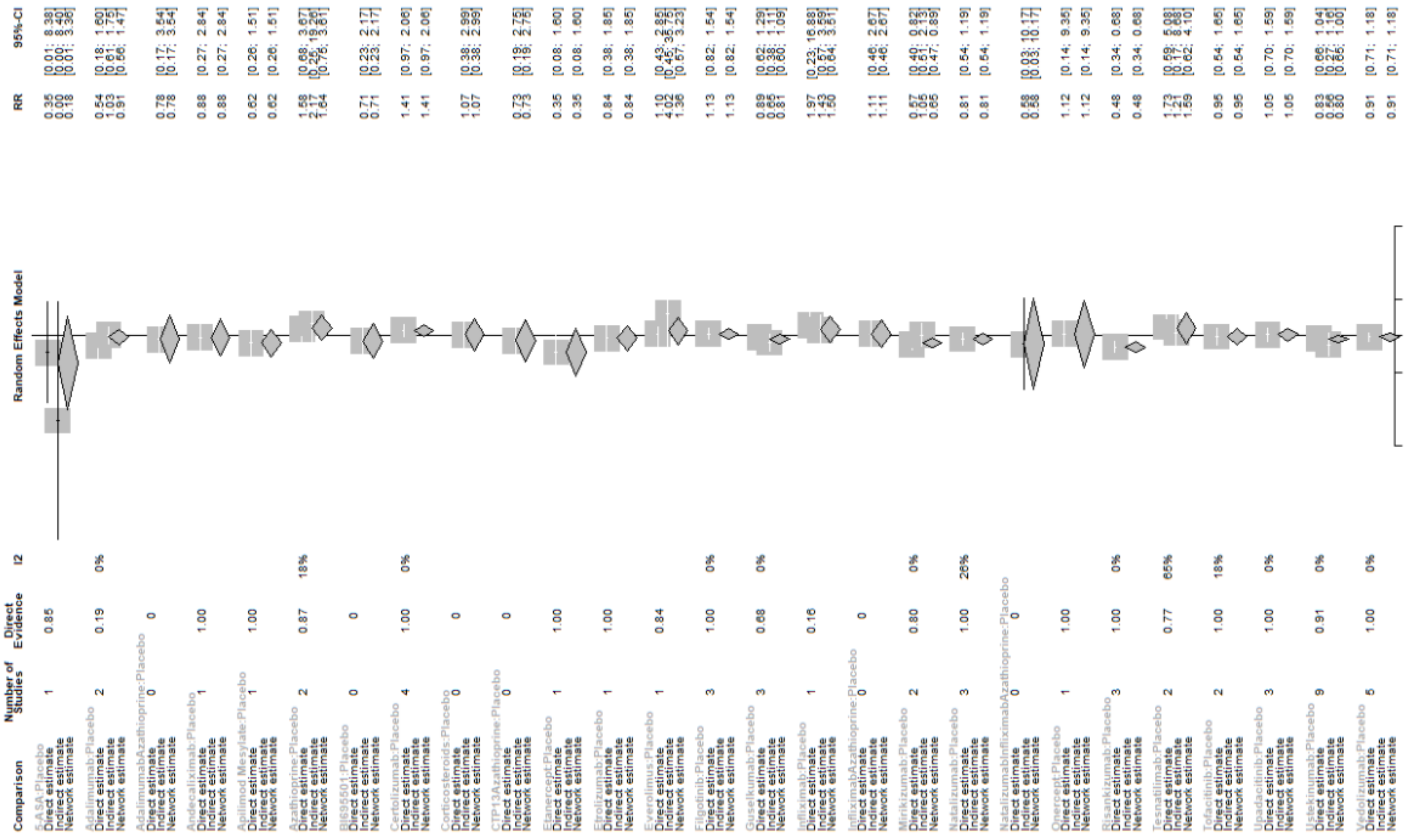

Network forest plot Total Adverse Events

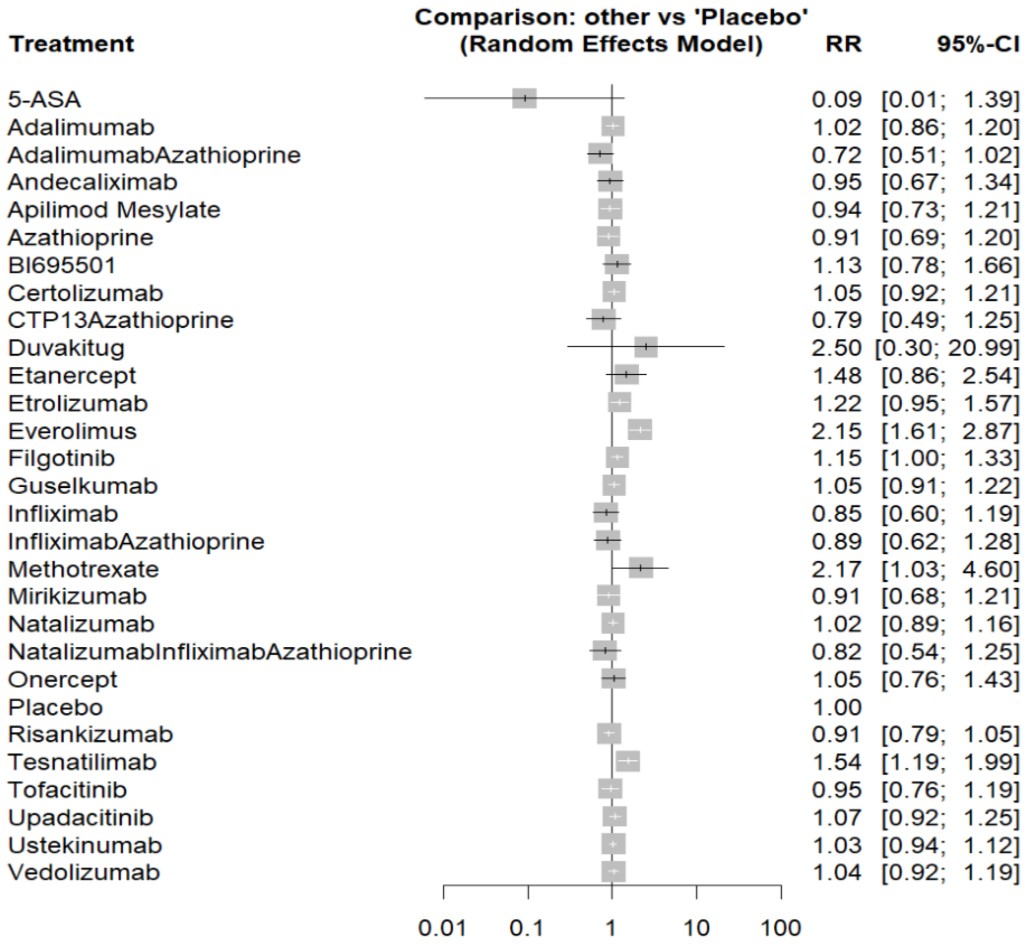

SUCRA probabilities Total Adverse Events

	SUCRA (common)	SUCRA (random)
5-ASA	0.9818	0.9496
AdalimumabAzathioprine	0.9018	0.8696
Infliximab	0.8164	0.7807
CTP13Azathioprine	0.8743	0.7775
NatalizumabInfliximabAzathioprine	0.8354	0.7650
InfliximabAzathioprine	0.7332	0.7007
Risankizumab	0.7464	0.6875
Mirikizumab	0.6714	0.6793
Azathioprine	0.6943	0.6721
Tofacitinib	0.6250	0.6379
Apilimod Mesylate	0.6243	0.6257
Andecaliximab	0.5486	0.5700
Placebo	0.5293	0.5400
Adalimumab	0.5493	0.4975
Ustekinumab	0.4696	0.4879
Natalizumab	0.4393	0.4836
Vedolizumab	0.4571	0.4639
Onercept	0.4154	0.4539
Certolizumab	0.4268	0.4439
Guselkumab	0.3979	0.4368
Upadacitinib	0.3664	0.3986
BI695501	0.3532	0.3975
Filgotinib	0.2075	0.2996
Etrolizumab	0.2121	0.2368
Duvakitug	0.2432	0.2214
Etanercept	0.1536	0.1979
Tesnatilimab	0.1136	0.1232
Methotrexate	0.0689	0.0575
Everolimus	0.0439	0.0443

- based on 100 simulations

Direct/indirect/network estimates forest plots Total Adverse Events

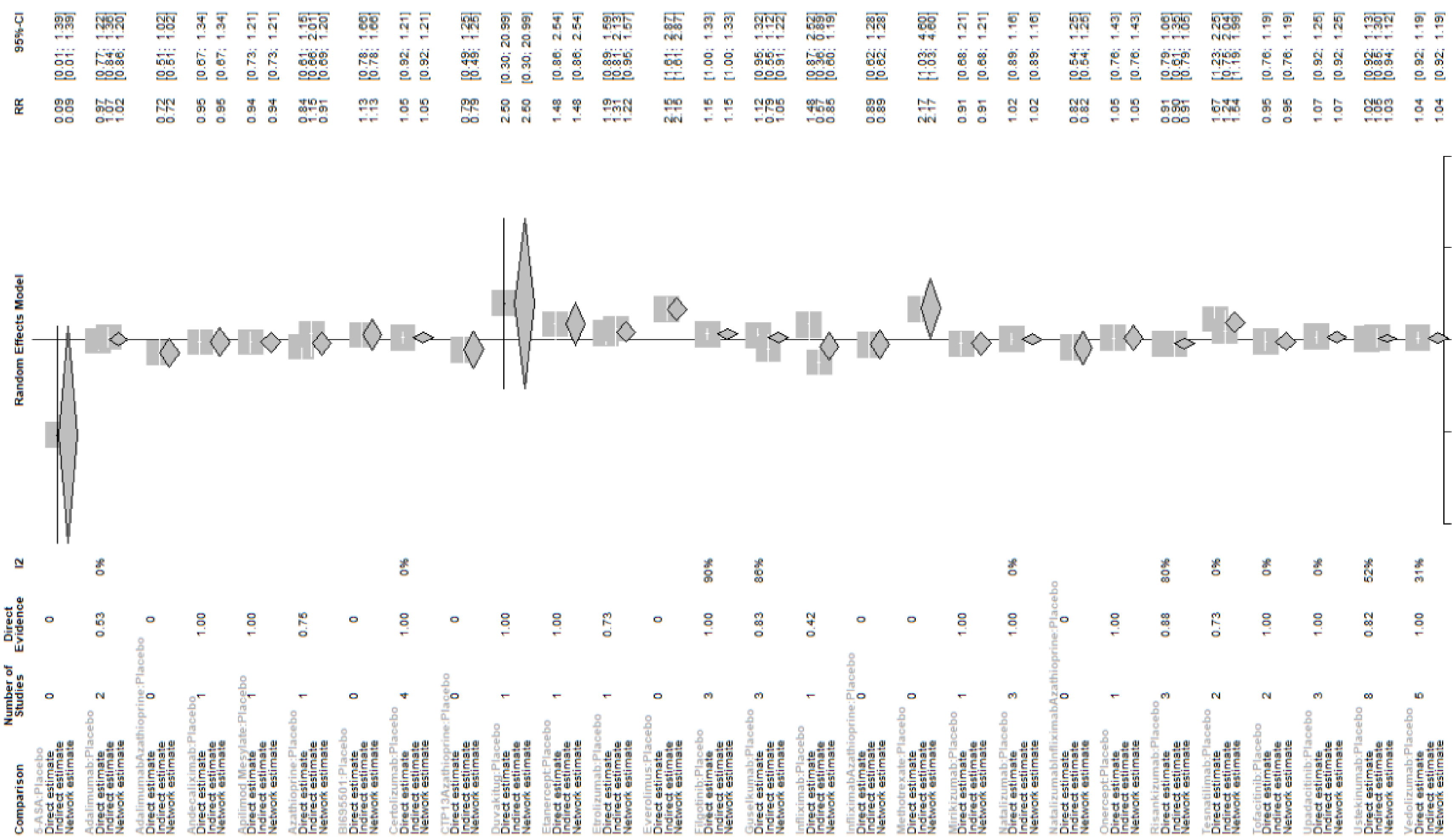

eFigures 3. SUBGROUP AND SENSITIVITY ANALYSES

CLINICAL REMISSION

Subgroup analysis for studies with >=50% advanced therapy-naïve patients

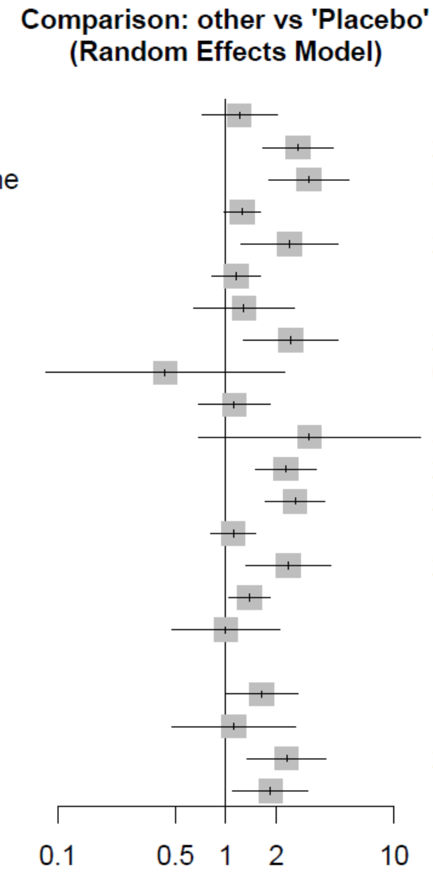

Treatment	RR	95%−CI
5−ASA	1.20	[0.72; 2.01]
Adalimumab	2.69	[1.66; 4.35]
AdalimumabAzathioprine	3.11	[1.79; 5.40]
Azathioprine	1.25	[0.98; 1.59]
BI695501	2.39	[1.23; 4.62]
Certolizumab	1.15	[0.82; 1.61]
Corticosteroids	1.28	[0.64; 2.55]
CTP13Azathioprine	2.43	[1.28; 4.61]
Etanercept	0.43	[0.08; 2.23]
Everolimus	1.12	[0.68; 1.85]
Humicade	3.14	[0.69; 14.26]
Infliximab	2.27	[1.50; 3.45]
InfliximabAzathioprine	2.58	[1.71; 3.88]
Methotrexate	1.11	[0.81; 1.50]
MethotrexateInfliximab	2.35	[1.31; 4.20]
Natalizumab	1.38	[1.04; 1.83]
Onercept	1.00	[0.48; 2.10]
Placebo	1.00	
Prednisone	1.63	[1.00; 2.68]
Tofacitinib	1.11	[0.48; 2.58]
Ustekinumab	2.30	[1.34; 3.96]
Vedolizumab	1.84	[1.10; 3.08]

Subgroup analysis for studies with <50% advanced therapy-naïve patients

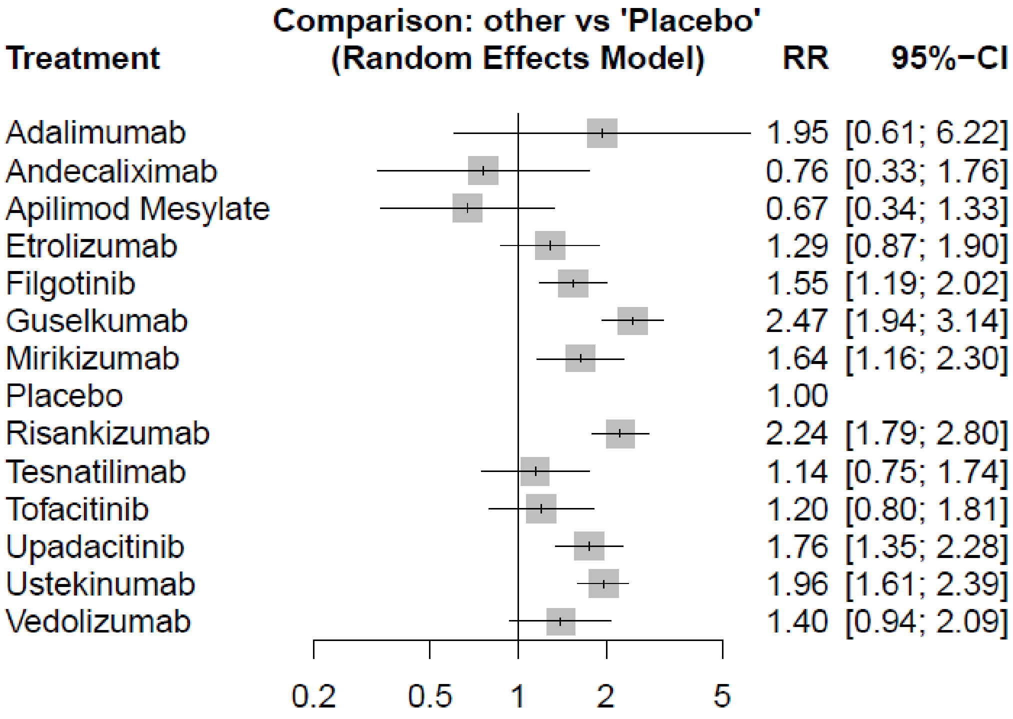

Sensitivity analysis for studies with less than 20% of patients on concomitant thiopurines

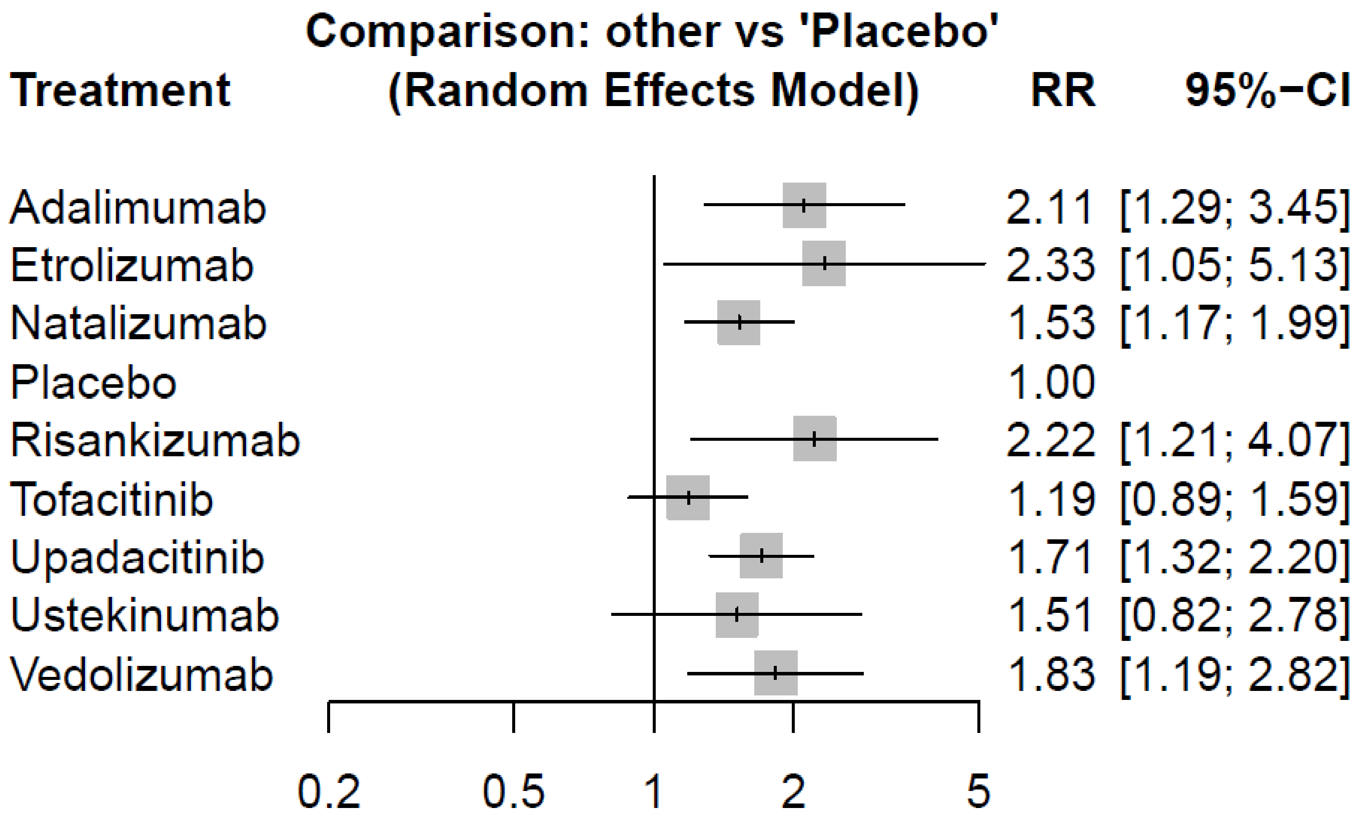

Sensitivity analysis for studies with more than 20% of patients on concomitant thiopurines

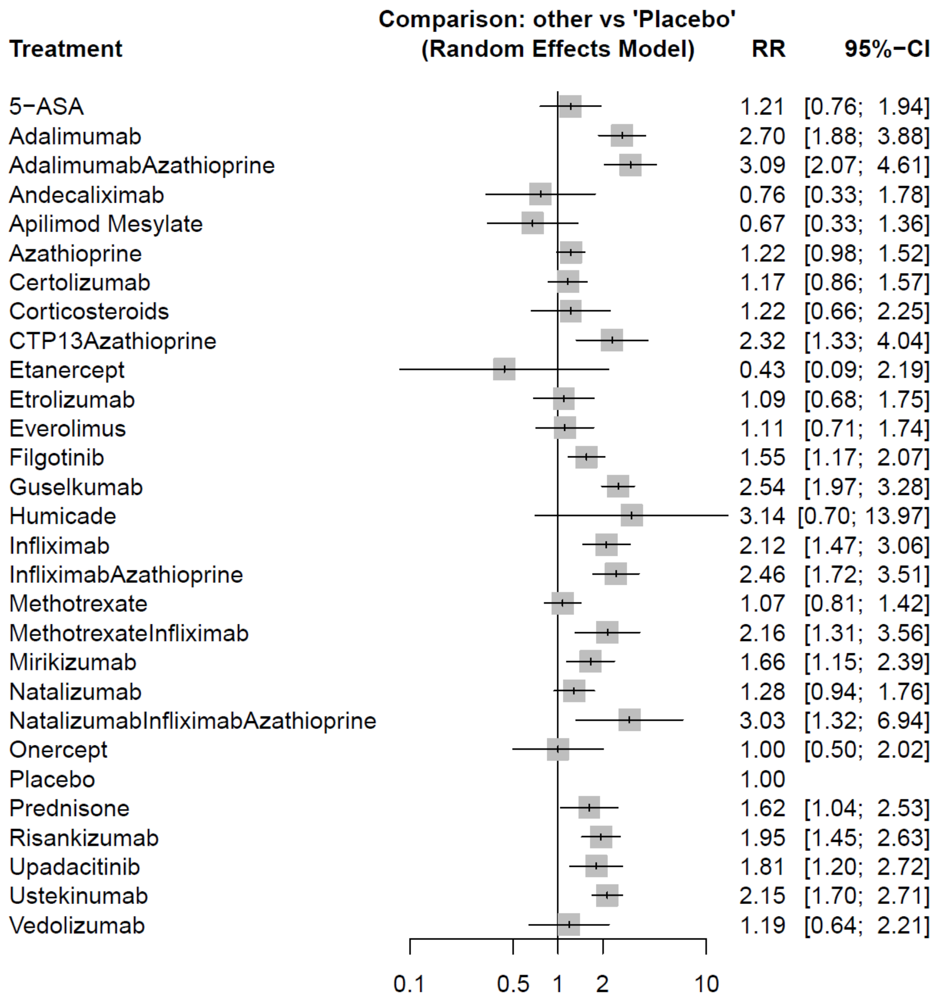

Sensitivity analysis for studies investigating advanced treatments (excluding studies on thiopurines/methotrexate if not compared to an advanced treatment)

Comparison: other vs 'Placebo' (Random Effects Model)

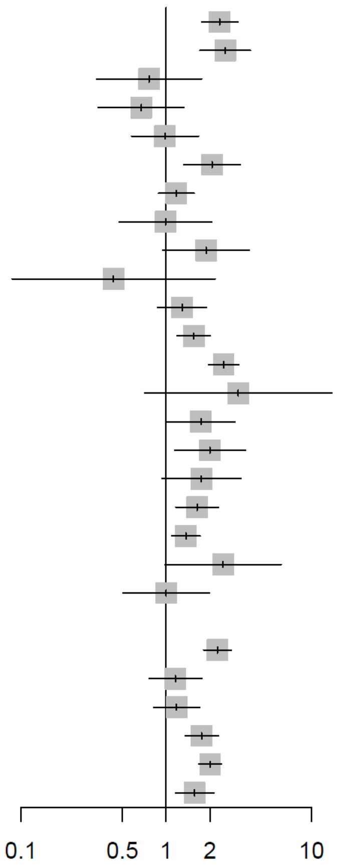

Treatment	RR	95%−CI
Adalimumab	2.34	[1.75; 3.11]
AdalimumabAzathioprine	2.55	[1.71; 3.81]
Andecaliximab	0.76	[0.33; 1.75]
Apilimod Mesylate	0.67	[0.34; 1.33]
Azathioprine	0.98	[0.58; 1.67]
BI695501	2.07	[1.33; 3.23]
Certolizumab	1.17	[0.89; 1.55]
Corticosteroids	0.99	[0.48; 2.06]
CTP13Azathioprine	1.88	[0.95; 3.74]
Etanercept	0.43	[0.09; 2.17]
Etrolizumab	1.29	[0.88; 1.90]
Filgotinib	1.55	[1.19; 2.02]
Guselkumab	2.49	[1.96; 3.15]
Humicade	3.14	[0.71; 13.84]
Infliximab	1.73	[1.00; 2.98]
InfliximabAzathioprine	2.00	[1.14; 3.50]
MethotrexateInfliximab	1.75	[0.94; 3.27]
Mirikizumab	1.63	[1.17; 2.29]
Natalizumab	1.36	[1.09; 1.71]
NatalizumabInfliximabAzathioprine	2.47	[0.98; 6.18]
Onercept	1.00	[0.51; 1.97]
Placebo	1.00	
Risankizumab	2.26	[1.81; 2.81]
Tesnatilimab	1.16	[0.76; 1.76]
Tofacitinib	1.18	[0.82; 1.70]
Upadacitinib	1.76	[1.35; 2.28]
Ustekinumab	2.01	[1.67; 2.40]
Vedolizumab	1.57	[1.16; 2.12]

Sensitivity analysis for studies published on or after 2003 (start of the "biologic era")

Comparison: other vs 'Placebo'
(Random Effects Model)

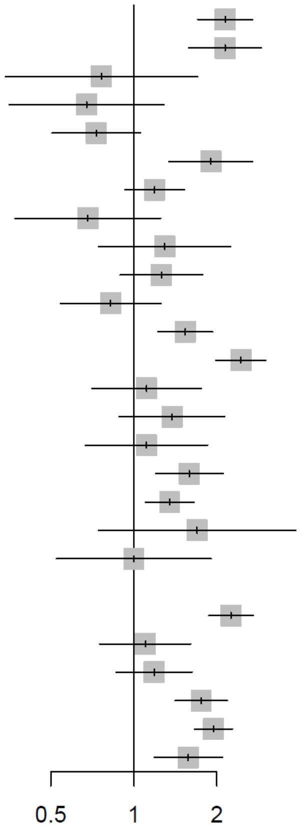

Treatment	RR	95%−CI
Adalimumab	2.14	[1.70; 2.70]
AdalimumabAzathioprine	2.14	[1.58; 2.90]
Andecaliximab	0.76	[0.34; 1.70]
Apilimod Mesylate	0.67	[0.35; 1.29]
Azathioprine	0.73	[0.50; 1.06]
BI695501	1.90	[1.34; 2.70]
Certolizumab	1.19	[0.93; 1.52]
Corticosteroids	0.68	[0.37; 1.25]
CTP13Azathioprine	1.29	[0.75; 2.24]
Etrolizumab	1.26	[0.89; 1.78]
Everolimus	0.82	[0.54; 1.25]
Filgotinib	1.54	[1.22; 1.93]
Guselkumab	2.44	[1.98; 3.01]
Infliximab	1.11	[0.70; 1.75]
InfliximabAzathioprine	1.37	[0.88; 2.13]
MethotrexateInfliximab	1.11	[0.67; 1.85]
Mirikizumab	1.59	[1.20; 2.10]
Natalizumab	1.35	[1.10; 1.65]
NatalizumabInfliximabAzathioprine	1.69	[0.74; 3.86]
Onercept	1.00	[0.52; 1.91]
Placebo	1.00	
Risankizumab	2.25	[1.87; 2.70]
Tesnatilimab	1.10	[0.75; 1.61]
Tofacitinib	1.18	[0.86; 1.63]
Upadacitinib	1.75	[1.41; 2.18]
Ustekinumab	1.94	[1.66; 2.28]
Vedolizumab	1.57	[1.18; 2.09]

ENDOSCOPIC REMISSION

Sensitivity analysis for studies with follow-up up to week 26

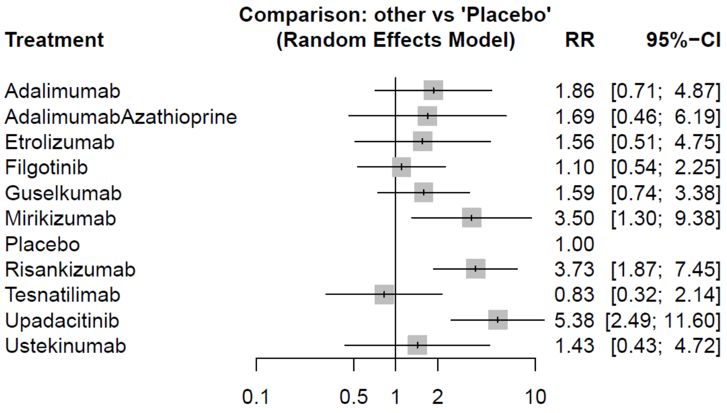

Sensitivity analysis for studies with endoscopic remission definition as SES-CD score ≤4

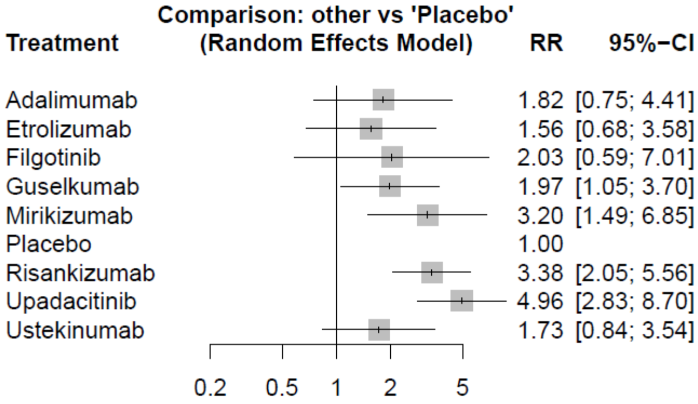

Sensitivity analysis for studies with only advanced therapy-exposed populations

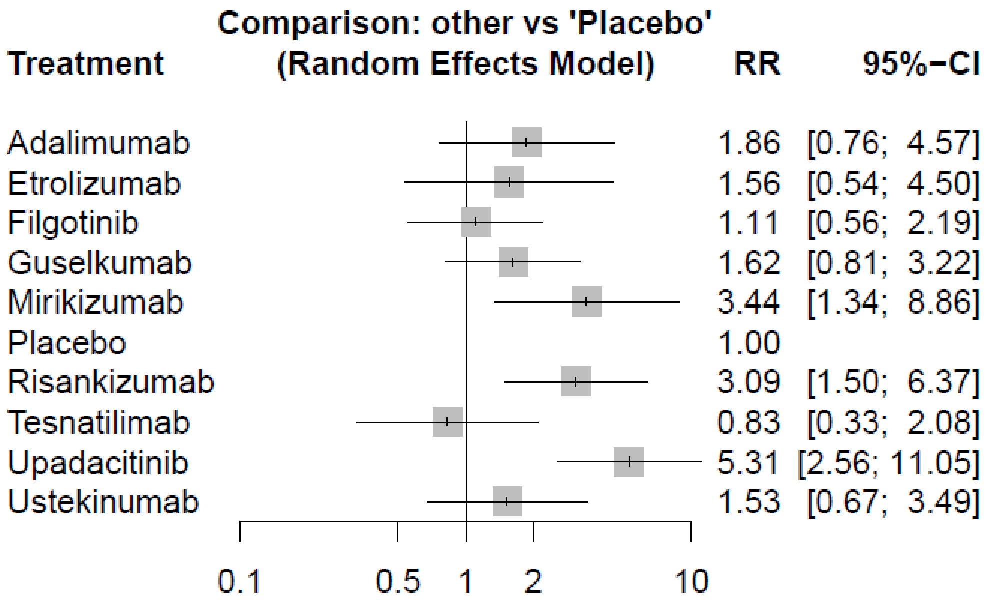

eFigures 4. Comparison adjusted funnel plots for the assessment of small study effects

Clinical remission

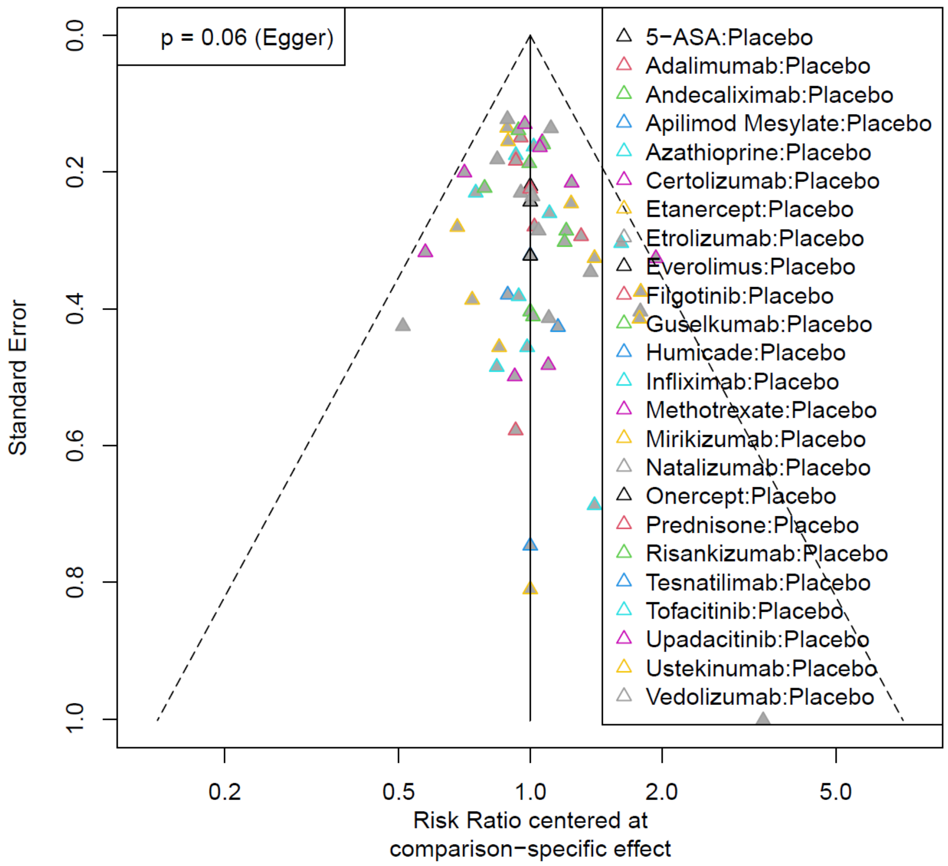

Clinical response

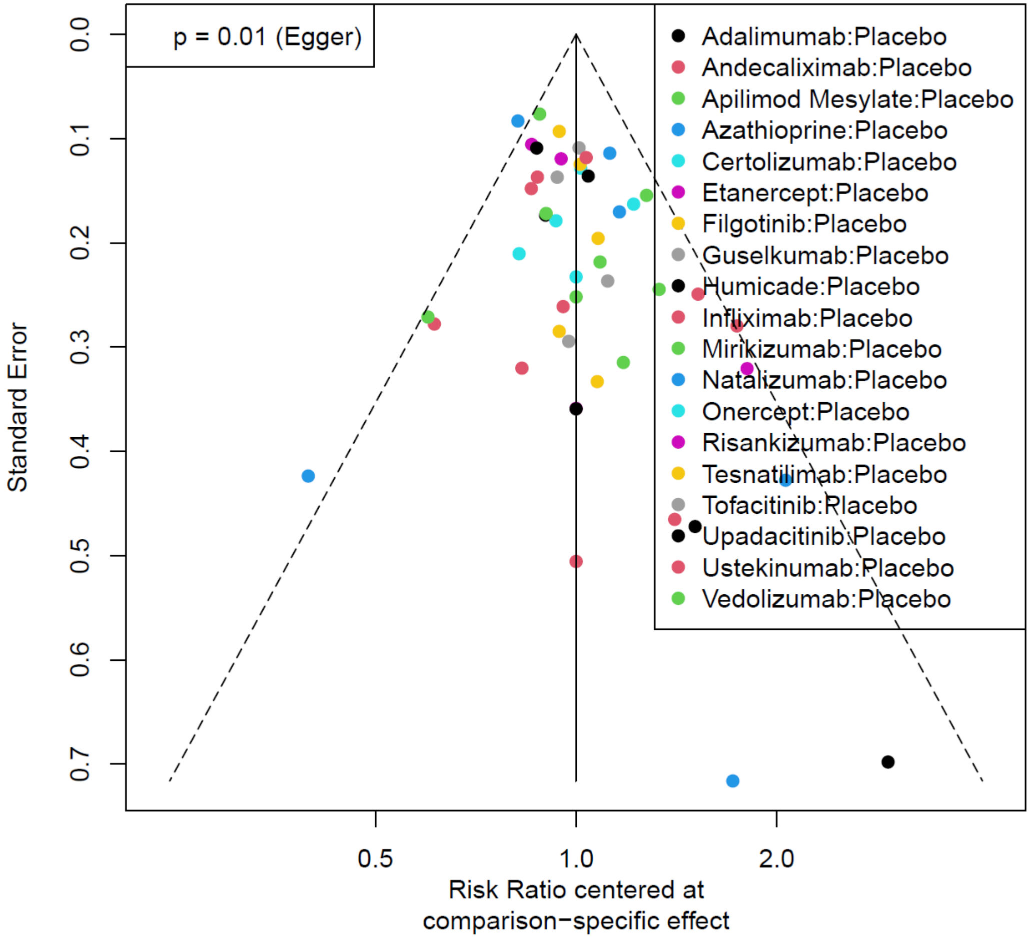

Endoscopic remission

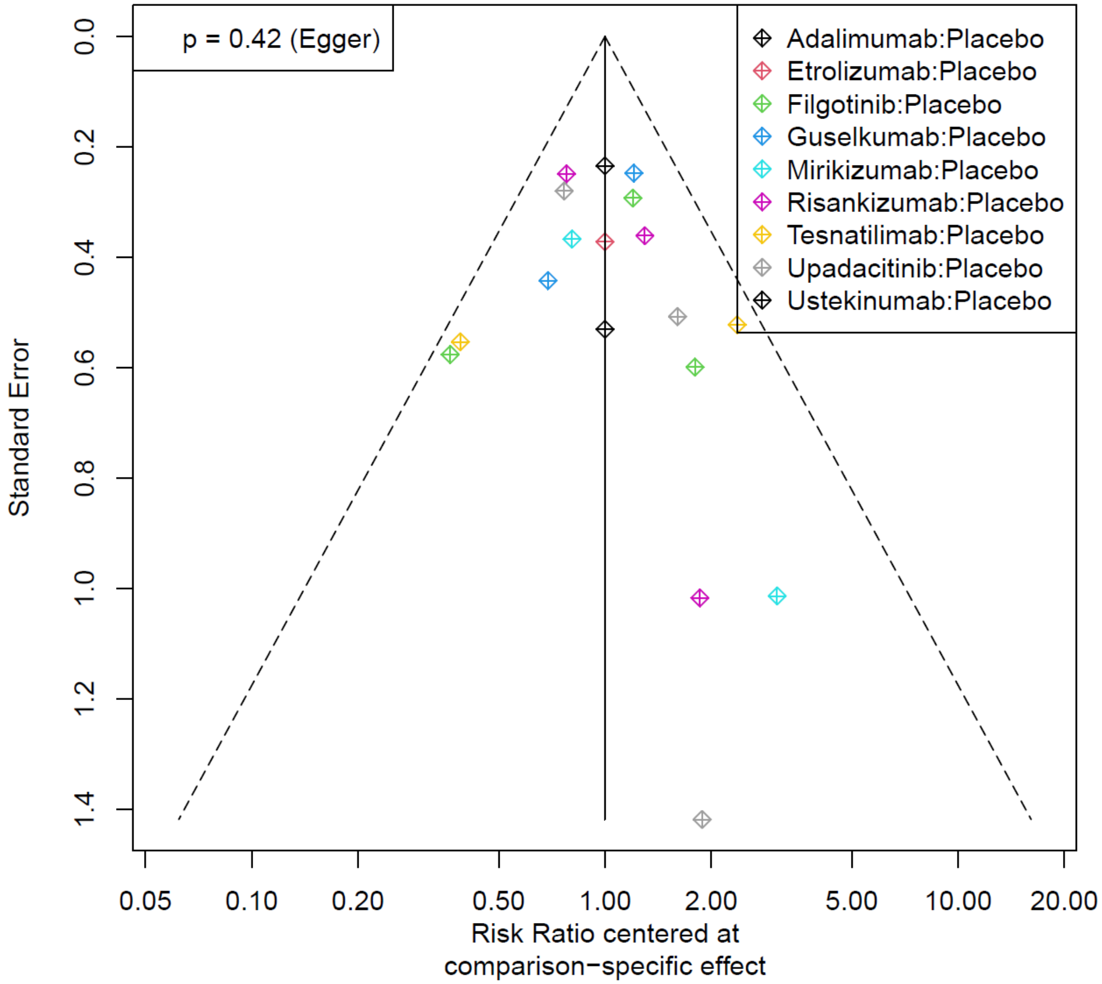

eAppendix 1. Search strategies

CENTRAL via Cochrane Library

#1 [mh "Inflammatory Bowel Diseases"] or [mh "Colitis, Ulcerative"] or [mh "Crohn Disease"] or ("Inflammatory Bowel Disease" or "Inflammatory Bowel Diseases" or Crohn* or Colitis or Enteritis or Proctocolitis" or Ileocolitis or Enterocolitis or Ileitides or Ileitis or Colorectitis"):ti,ab with Cochrane Library publication date from Jan 2018 to present, in Trials

#2 (clinicaltrials* or trialsearch*):so

#3 #1 NOT #2

MEDLINE via Ovid SP

1 exp Inflammatory Bowel Diseases/ or Colitis, Ulcerative/ or Crohn Disease/ or ("Inflammatory Bowel Disease" or "Inflammatory Bowel Diseases" or Crohn* or Colitis or Enteritis or Proctocolitis or Ileocolitis or Enterocolitis or Ileitides or Ileitis or Colorectitis).ti,ab.

2 ((Randomized Controlled Trial or Controlled Clinical Trial).pt. or (Randomi?ed or Placebo or Randomly or Trial or Groups).ab. or Drug Therapy.fs.) not (exp Animals/ not Humans.sh.)

3 1 and 2

4 limit 3 to ed=20180101-20221231

5 limit 3 to dt=20180101-20221231

6 4 or 5

Embase via Ovid SP

1 Randomized controlled trial/ or Controlled clinical study/ or randomization/ or intermethod comparison/ or double blind procedure/ or human experiment/ or (random$ or placebo or (open adj label) or ((double or single or doubly or singly) adj (blind or blinded or blindly)) or parallel group$1 or crossover or cross over or ((assign$ or match or matched or allocation) adj5 (alternate or group$1 or intervention$1 or patient$1 or subject$1 or participant$1)) or assigned or allocated or (controlled adj7 (study or design or trial)) or volunteer or volunteers).ti,ab. or (compare or compared or comparison or trial).ti. or ((evaluated or evaluate or evaluating or assessed or assess) and (compare or compared or comparing or comparison)).ab.

2 (random$ adj sampl$ adj7 ("cross section$" or questionnaire$1 or survey$ or database$1)).ti,ab. not (comparative study/ or controlled study/ or randomi?ed controlled.ti,ab. or randomly assigned.ti,ab.)

3 Cross-sectional study/ not (randomized controlled trial/ or controlled clinical study/ or controlled study/ or (randomi?ed controlled or control group$1).ti,ab.) (305385)

4 (((case adj control$) and random$) not randomi?ed controlled).ti,ab.

5 (Systematic review not (trial or study)).ti.

6 (nonrandom$ not random$).ti,ab.

7 ("Random field$" or (random cluster adj3 sampl$)).ti,ab.

8 (review.ab. and review.pt.) not trial.ti.

9 "we searched".ab. and (review.ti. or review.pt.)

10 ("update review" or (databases adj4 searched)).ab.

11 (rat or rats or mouse or mice or swine or porcine or murine or sheep or lambs or pigs or piglets or rabbit or rabbits or cat or cats or dog or dogs or cattle or bovine or monkey or monkeys or trout or marmoset$1).ti. and animal experiment/

12 Animal experiment/ not (human experiment/ or human/)

13 or/2-12

14 1 not 13

15 Inflammatory Bowel Disease/ or exp Crohn Disease/ or Ulcerative Colitis/ or Acute Severe Ulcerative Colitis/ or ("Inflammatory Bowel Disease" or "Inflammatory Bowel Diseases" or Crohn* or Colitis or Enteritis or Proctocolitis or Ileocolitis or Enterocolitis or Ileitides or Ileitis or Colorectitis).ti,ab.

16 14 and 15

17 limit 16 to dc=20180101-20221231

18 limit 16 to dd=20180101-20221231

19 17 or 18

20 limit 19 to embase

Science Citation Index-Expanded via Web of Science Core Collection

#3 #1 AND #2 Editions: Science Citation Index Expanded (SCI-EXPANDED)

#2 "Inflammatory Bowel Disease" or "Inflammatory Bowel Diseases" or Crohn* or Colitis or Enteritis or Proctocolitis or Ileocolitis or Enterocolitis or Ileitides or Ileitis or Colorectitis (Title) or "Inflammatory Bowel Disease" or "Inflammatory Bowel Diseases" or Crohn* or Colitis or Enteritis or Proctocolitis or Ileocolitis or Enterocolitis or Ileitides or Ileitis or Colorectitis (Abstract)

#1 Random* OR Blind* OR Allocat* OR Assign* OR Trial* OR Placebo* OR Crossover* OR Cross-Over* (Title) or Random* OR Blind* OR Allocat* OR Assign* OR Trial* OR Placebo* OR Crossover* OR Cross-Over* (Abstract)

eAppendix 2. References of included studies

1. Allez M, Sands BE, Feagan BG, et al. A Phase 2b, Randomised, Double-blind, Placebo-controlled, Parallel-arm, Multicenter Study Evaluating the Safety and Efficacy of Tesnatilimab in Patients with Moderately to Severely Active Crohn's Disease. J Crohns Colitis. 2023;17(8):1235-51. Epub 2023/03/21.

2. Ardizzone S, Bollani S, Manzionna G, Imbesi V, Colombo E, Porro GB. Comparison between methotrexate and azathioprine in the treatment of chronic active Crohn’s disease: a randomised, investigator-blind study. Digestive and liver disease. 2003 Sep 1;35(9):619-27.

3. Arora S, Katkov W, Cooley J, et al. Methotrexate in Crohn's disease: results of a randomized, double-blind, placebo-controlled trial. Hepatogastroenterology. 1999;46(27):1724-9. Epub 1999/08/03.

4. Candy S, Wright J, Gerber M, et al. A controlled double blind study of azathioprine in the management of Crohn's disease. Gut. 1995;37(5):674-8. Epub 1995/11/01.

5. Chen B, Gao X, Zhong J, et al. Efficacy and safety of adalimumab in Chinese patients with moderately to severely active Crohn's disease: results from a randomized trial. Therap Adv Gastroenterol. 2020;13:1756284820938960. Epub 2020/08/01.

6. Chen M, Gao X, Cao Q, Rossiter G, Kitagawa T, Sun Y, Yang L. Efficacy and safety of intravenous vedolizumab treatment in Chinese patients with moderate-to-severe Crohn's disease. Clinics and Research in Hepatology and Gastroenterology. 2025;49(6):102591.

7. Colombel JF, Sandborn WJ, Rutgeerts P, et al. Adalimumab for maintenance of clinical response and remission in patients with Crohn's disease: the CHARM trial. Gastroenterology. 2007;132(1):52-65. Epub 2007/01/24.

8. Colombel JF, Sandborn WJ, Reinisch W, Mantzaris GJ, Kornbluth A, Rachmilewitz D, Lichtiger S, d'Haens G, Diamond RH, Broussard DL, Tang KL. Infliximab, azathioprine, or combination therapy for Crohn's disease. New England journal of medicine. 2010 Apr 15;362(15):1383-95.

9. D'Haens G, Baert F, van Assche G, et al. Early combined immunosuppression or conventional management in patients with newly diagnosed Crohn's disease: an open randomised trial. Lancet. 2008;371(9613):660-7. Epub 2008/02/26.

10. D'Haens G, Panaccione R, Baert F, et al. Risankizumab as induction therapy for Crohn's disease: results from the phase 3 ADVANCE and MOTIVATE induction trials. Lancet. 2022;399(10340):2015-30. Epub 2022/06/02.

11. D’Haens G, van Deventer S, VAN HOGEZAND RA, et al. Endoscopic and Histological Healing With Infliximab Anti–Tumor Necrosis Factor Antibodies in Crohn’s Disease: A European Multicenter Trial. GASTROENTEROLOGY. 1999;116(5):1029–34.

12. Duan Z, Luo J, Li W. Efficacy of Infliximab Combined with Azathioprine for Moderate to Severe Crohn's Disease. Chinese journal of gastroenterology. 2013;18(4):229-32.

13. Ewe K, Press AG, Singe CC, et al. Azathioprine combined with prednisolone or monotherapy with prednisolone in active Crohn's disease. Gastroenterology. 1993;105(2):367-72.

14. Feagan BG, Greenberg GR, Wild G, et al. Treatment of active Crohn's disease with MLN0002, a humanized antibody to the alpha4beta7 integrin. Clin Gastroenterol Hepatol. 2008;6(12):1370-7. Epub 2008/10/03.

15. Feagan BG, McDonald JW, Panaccione R, et al. Methotrexate in combination with infliximab is no more effective than infliximab alone in patients with Crohn's disease. Gastroenterology. 2014;146(3):681-8.e1. Epub 2013/11/26.

16. Feagan BG, Rochon J, Fedorak RN, et al. Methotrexate for the treatment of Crohn's disease. New England Journal of Medicine. 1995;332(5):292-7.

17. Feagan BG, Sandbom WJ, D'Haens G, et al. INDUCTION THERAPY WITH THE SELECTIVE INTERLEUKIN-23 INHIBITOR RISANKIZUMAB IN PATIENTS WITH MODERATE-TO SEVERE CROHN'S DISEASE: A RANDOMISED, DOUBLE-BLIND, PLACEBO-CONTROLLED PHASE 2 STUDY. Lancet. 2017;389:1699-709.

18. Feagan BG, Sandborn WJ, Gasink C, et al. Ustekinumab as Induction and Maintenance Therapy for Crohn's Disease. N Engl J Med. 2016;375(20):1946-60. Epub 2016/12/14.

19. Ferrante M, D'Haens G, Jairath V, et al. Efficacy and safety of mirikizumab in patients with moderately-to-severely active Crohn's disease: a phase 3, multicentre, randomised, double-blind, placebo-controlled and active-controlled, treat-through study. The Lancet. 2024;404(10470):2423-2436.

20. Ghosh S, Goldin E, Gordon FH, et al. Natalizumab for Active Crohn's Disease. n engl j med 2003;348(1):24-32.

21. Gordon FH, Lai CW, Hamilton MI, et al. A randomized placebo-controlled trial of a humanized monoclonal antibody to alpha4 integrin in active Crohn's disease. Gastroenterology. 2001;121(2):268-74. Epub 2001/08/07.

22. Hanauer S, Feagan BG, Lichtenstein GR, et al. Maintenance infliximab for Crohn's disease: the ACCENT I randomised trial. The Lancet. 2002;359.

23. Hanauer S, Liedert B, Balser S, et al. Safety and efficacy of BI 695501 versus adalimumab reference product in patients with advanced Crohn's disease (VOLTAIRE-CD): a multicentre, randomised, double-blind, phase 3 trial. Lancet Gastroenterol Hepatol. 2021;6(10):816-25. Epub 2021/08/14.

24. Hanauer SB, Sandborn WJ, Rutgeerts P, et al. Human anti-tumor necrosis factor monoclonal antibody (adalimumab) in Crohn's disease: the CLASSIC-I trial. Gastroenterology. 2006;130(2):323-33; quiz 591. Epub 2006/02/14.

25. Hart A, Panaccione R, Steinwurz F, et al. Efficacy and safety of guselkumab subcutaneous induction and maintenance in participants with moderately to severely active Crohn's disease: results from the phase 3 GRAVITI study. Gastroenterology. 2025.

26. Jairath V, Kierkus J, Duvall GA, et al. OP40 Duvakitug (TEV-48574), an anti-TL1a monoclonal antibody, demonstrates efficacy and favourable safety as an induction treatment in adults with moderately to severely active Crohn's disease: results from a phase 2b, randomised, double-blind, placebo-controlled dose-ranging, basket trial (RELIEVE UCCD). Journal of Crohn's and Colitis. 2025;19(Supplement_1):i77-i78.

27. Lemann M, Mary JY, Duclos B, et al. Infliximab plus azathioprine for steroid-dependent Crohn's disease patients: a randomized placebo-controlled trial. Gastroenterology. 2006;130(4):1054-61. Epub 2006/04/19.

28. Loftus EV, Jr., Panes J, Lacerda AP, et al. Upadacitinib Induction and Maintenance Therapy for Crohn's Disease. N Engl J Med. 2023;388(21):1966-80. Epub 2023/05/24.

29. Mantzaris G, Ployzou P, Karagiannidis A, et al. A prospective, randomized trial of infliximab (IFX) and azathioprine (AZA) for the induction and maintenance of remission of steroid-dependent Crohn's disease. Gastroenterology. 2004;126:A437.

30. Maté-Jiménez J, Hermida C, Cantero-Perona J, Moreno-Otero R. 6-mercaptopurine or methotrexate added to prednisone induces and maintains remission in steroid-dependent inflammatory bowel disease. Eur J Gastroenterol Hepatol. 2000;12(11):1227-33. Epub 2000/12/09.

31. Matsumoto T, Motoya S, Watanabe K, et al. Adalimumab Monotherapy and a Combination with Azathioprine for Crohn's Disease: A Prospective, Randomized Trial. J Crohns Colitis. 2016;10(11):1259-66. Epub 2016/10/30.

32. Panaccione R, Danese S, Feagan BG, et al. Efficacy and safety of guselkumab therapy in patients with moderately to severely active Crohn’s disease: results of the GALAXI 2 & 3 phase 3 studies. Gastroenterology. 2024;166(5):1057b-1057b1052.

33. Oren R, Moshkowitz M, Odes S, et al. Methotrexate in chronic active Crohn's disease: a double-blind, randomized, Israeli multicenter trial. Am J Gastroenterol. 1997;92(12):2203-9. Epub 1997/12/17.

34. Panes J, Sandborn WJ, Schreiber S, et al. Tofacitinib for induction and maintenance therapy of Crohn's disease: results of two phase IIb randomised placebo-controlled trials. Gut. 2017;66(6):1049-59. Epub 2017/02/18.

35. Peyrin-Biroulet L, Chapman JC, Colombel JF, Caprioli F, D’Haens G, Ferrante M, Schreiber S, Atreya R, Danese S, Lindsay JO, Bossuyt P. Risankizumab versus ustekinumab for moderate-to-severe Crohn’s disease. New England Journal of Medicine. 2024 Jul 18;391(3):213-23.

36. Present DH, Rutgeerts P, Targan S, et al. INFLIXIMAB FOR THE TREATMENT OF FISTULAS IN PATIENTS WITH CROHN’S DISEASE. The New England Journal of Medicine. 1999;340(18):1398-404.

37. Reinisch W, Panés J, Lémann M, et al. A multicenter, randomized, double-blind trial of everolimus versus azathioprine and placebo to maintain steroid-induced remission in patients with moderate-to-severe active Crohn's disease. Am J Gastroenterol. 2008;103(9):2284-92. Epub 2008/08/02.

38. Rutgeerts P, Sandborn WJ, Fedorak RN, et al. Onercept for moderate-to-severe Crohn's disease: a randomized, double-blind, placebo-controlled trial. Clin Gastroenterol Hepatol. 2006;4(7):888-93. Epub 2006/06/27.

39. Rutgeerts P, Van Assche G, Sandborn WJ, Wolf DC, Geboes K, Colombel JF, Reinisch W, Kumar A, Lazar A, Camez A, Lomax KG. Adalimumab induces and maintains mucosal healing in patients with Crohn's disease: data from the EXTEND trial. Gastroenterology. 2012 May 1;142(5):1102-11.

40. Sandborn WJ, Colombel JF, Enns R, et al. Natalizumab Induction and Maintenance Therapy for Crohn’s Disease. N engl j med. 2005;353(18):1913-25.

41. Sandborn WJ, D'Haens GR, Reinisch W, et al. Guselkumab for the Treatment of Crohn's Disease: Induction Results From the Phase 2 GALAXI-1 Study. Gastroenterology. 2022;162(6):1650-64 e8. Epub 2022/02/09.

42. Sandborn WJ, Feagan BG, Fedorak RN, et al. A randomized trial of Ustekinumab, a human interleukin-12/23 monoclonal antibody, in patients with moderate-to-severe Crohn's disease. Gastroenterology. 2008;135(4):1130-41. Epub 2008/08/19.

43. Sandborn WJ, Feagan BG, Hanauer SB, et al. An engineered human antibody to TNF (CDP571) for active Crohn's disease: a randomized double-blind placebo-controlled trial. Gastroenterology. 2001;120(6):1330-8. Epub 2001/04/21.

44. Sandborn WJ, Feagan BG, Loftus EV, Jr., et al. Efficacy and Safety of Upadacitinib in a Randomized Trial of Patients With Crohn's Disease. Gastroenterology. 2020;158(8):2123-38 e8. Epub 2020/02/12.

45. Sandborn WJ, Feagan BG, Rutgeerts P, et al. Vedolizumab as induction and maintenance therapy for Crohn's disease. N Engl J Med. 2013;369(8):711-21. Epub 2013/08/24.

46. Sandborn WJ, Feagan BG, Stoinov S, et al. Certolizumab pegol for the treatment of Crohn's disease. New England journal of medicine. 2007;357(3):228-38.

47. Sandborn WJ, Gasink C, Gao LL, et al. Ustekinumab induction and maintenance therapy in refractory Crohn's disease. N Engl J Med. 2012;367(16):1519-28. Epub 2012/10/19.

48. Sandborn WJ, Ghosh S, Panes J, et al. A phase 2 study of tofacitinib, an oral Janus kinase inhibitor, in patients with Crohn's disease. Clin Gastroenterol Hepatol. 2014;12(9):1485-93 e2. Epub 2014/02/01.

49. Sandborn WJ, Hanauer SB, Katz S, et al. Etanercept for active Crohn's disease: a randomized, double-blind, placebo-controlled trial. Gastroenterology. 2001;121(5):1088-94. Epub 2001/10/26.

50. Sandborn WJ, Hanauer SB, Rutgeerts P, et al. Adalimumab for maintenance treatment of Crohn's disease: results of the CLASSIC II trial. Gut. 2007;56(9):1232-9. Epub 2007/02/15.

51. Sandborn WJ, Panes J, Danese S, et al. Etrolizumab as induction and maintenance therapy in patients with moderately to severely active Crohn's disease (BERGAMOT): a randomised, placebo-controlled, double-blind, phase 3 trial. Lancet Gastroenterol Hepatol. 2023;8(1):43-55. Epub 2022/10/15.

52. Sandborn WJ, Schreiber S, Feagan BG, et al. Certolizumab pegol for active Crohn's disease: a placebo-controlled, randomized trial. Clin Gastroenterol Hepatol. 2011;9(8):670-8 e3. Epub 2011/06/07.

53. Sands BE, Anderson FH, Bernstein CN, et al. Infliximab Maintenance Therapy for Fistulizing Crohn's Disease. N Engl J Med. 2004;350(9):876-85.

54. Sands BE, Peyrin-Biroulet L, Kierkus J, et al. Efficacy and Safety of Mirikizumab in a Randomized Phase 2 Study of Patients With Crohn's Disease. Gastroenterology. 2022;162(2):495-508. Epub 2021/11/09.

55. Sands BE, Feagan BG, Rutgeerts P, et al. Effects of vedolizumab induction therapy for patients with Crohn's disease in whom tumor necrosis factor antagonist treatment failed. Gastroenterology. 2014;147(3):618-27 e3. Epub 2014/05/27.

56. Sands BE, Jacobson EW, Sylwestrowicz T, et al. Randomized, double-blind, placebo-controlled trial of the oral interleukin-12/23 inhibitor apilimod mesylate for treatment of active Crohn's disease. Inflammatory bowel diseases. 2010;16(7):1209-18.

57. Sands BE, Irving PM, Hoops T, et al. Ustekinumab versus adalimumab for induction and maintenance therapy in biologic-naive patients with moderately to severely active Crohn's disease: a multicentre, randomised, double-blind, parallel-group, phase 3b trial. Lancet. 2022;399(10342):2200-11. Epub 2022/06/13.

58. Sands BE, Kozarek R, Spainhour J, et al. Safety and tolerability of concurrent natalizumab treatment for patients with Crohn's disease not in remission while receiving infliximab. Inflamm Bowel Dis. 2007;13(1):2-11. Epub 2007/01/09.

59. Schreiber S, Rutgeerts P, Fedorak RN, et al. A randomized, placebo-controlled trial of certolizumab pegol (CDP870) for treatment of Crohn's disease. Gastroenterology. 2005;129(3):807-18. Epub 2005/09/07.

60. Schreiber S, Siegel CA, Friedenberg KA, et al. A Phase 2, Randomized, Placebo-Controlled Study Evaluating Matrix Metalloproteinase-9 Inhibitor, Andecaliximab, in Patients With Moderately to Severely Active Crohn's Disease. J Crohns Colitis. 2018;12(9):1014-20. Epub 2018/05/31.

61. Schröder O, Blumenstein I, Stein J. Combining infliximab with methotrexate for the induction and maintenance of remission in refractory Crohn's disease: a controlled pilot study. European journal of gastroenterology & hepatology. 2006;18(1):11-6.

62. Summers RW, Switz DM, Sessions JT, Jr., et al. National Cooperative Crohn's Disease Study: results of drug treatment. Gastroenterology. 1979;77(4 Pt 2):847-69. Epub 1979/10/01.

63. Targan S, Hanauer S, SANDER JH, et al. A SHORT-TERM STUDY OF CHIMERIC MONOCLONAL ANTIBODY cA2 TO TUMOR NECROSIS FACTOR a FOR CROHN’S DISEASE. The New England Journal of Medicine 1997;337(15):1029-35.

64. Targan SR, Feagan BG, Fedorak RN, et al. Natalizumab for the treatment of active Crohn's disease: results of the ENCORE Trial. Gastroenterology. 2007;132(5):1672-83. Epub 2007/05/09.

65. Vermeire S, Schreiber S, Petryka R, et al. Clinical remission in patients with moderate-to-severe Crohn's disease treated with filgotinib (the FITZROY study): results from a phase 2, double-blind, randomised, placebo-controlled trial. Lancet. 2017;389(10066):266-75. Epub 2016/12/19.

66. Vermeire S, Schreiber S, Rubin DT, et al. Efficacy and safety of filgotinib as induction and maintenance therapy for Crohn's disease (DIVERSITY): a phase 3, double-blind, randomised, placebo-controlled trial. The Lancet Gastroenterology & Hepatology. 2025;10(2):138-153.

67. Watanabe K, Motoya S, Ogata H, et al. Effects of vedolizumab in Japanese patients with Crohn's disease: a prospective, multicenter, randomized, placebo-controlled Phase 3 trial with exploratory analyses. J Gastroenterol. 2020;55(3):291-306. Epub 2019/12/15.

68. Watanabe M, Hibi T, Lomax KG, et al. Adalimumab for the induction and maintenance of clinical remission in Japanese patients with Crohn's disease. J Crohns Colitis. 2012;6(2):160-73. Epub 2012/02/14.

69. Winter TA, Wright J, Ghosh S, et al. Intravenous CDP870, a PEGylated Fab' fragment of a humanized antitumour necrosis factor antibody, in patients with moderate-to-severe Crohn's disease: an exploratory study. Aliment Pharmacol Ther. 2004;20(11-12):1337-46. Epub 2004/12/21.

70. Ye BD, Pesegova M, Alexeeva O, et al. Efficacy and safety of biosimilar CT-P13 compared with originator infliximab in patients with active Crohn's disease: an international, randomised, double-blind, phase 3 non-inferiority study. Lancet. 2019;393(10182):1699-707. Epub 2019/04/02.
