# Supplementary material for: Immunomodulators and Advanced Therapies for Induction of Remission in Crohn’s Disease: A Systematic Review and Network Meta-Analysis
Source: Inflamm Bowel Dis. 2025 Sep 19;32(1):53–66. doi: 10.1093/ibd/izaf191 (PMC12759050; doi:10.1093/ibd/izaf191)
Supplement: izaf191_Supplementary_Data [file izaf191_supplementary_data.zip › Supplement 2 RoB details.pdf]

Figure 1. Risk of bias summary for the included studies in the advanced treatments for the induction of remission in Crohn's Disease NMA (n=79).

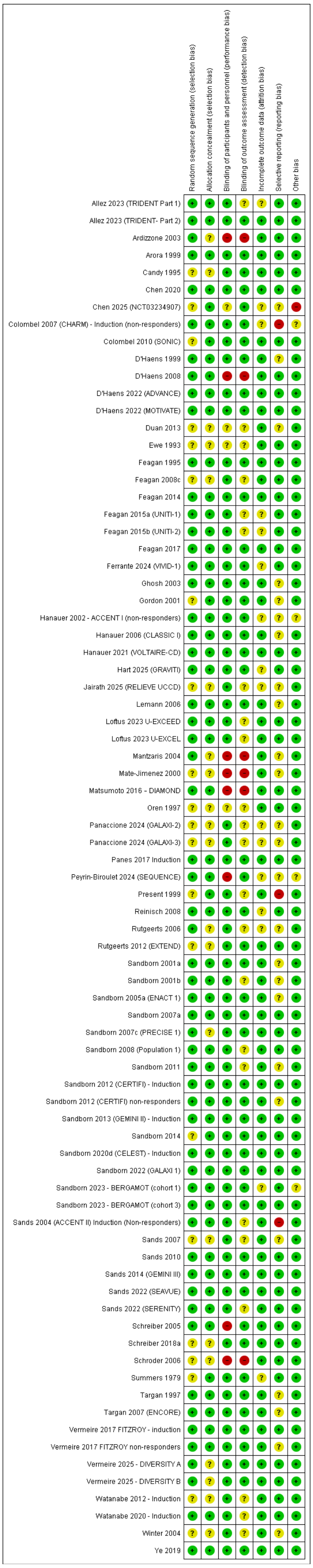

Table 1. Risk of bias judgements and justifications for the included studies in the advanced treatments for the induction of remission in Crohn's Disease NMA. Judgements for Reinisch 2008; Candy 1995; Summers 1979; Ewe 1993; Oren 1997; Mantzaris 2004; Ardizzone 2003; Mate-Jimenez 2000; Rhodes 1971; Klein 2974; Willoughby 1971; Present 1980; are taken from the Chande 2016 Cochrane systematic review (DOI: 10.1002/14651858.CD000545.pub5). Judgements for Arora 1999; Feagan 1995; Schroder 2006; Feagan 2014; are taken from the McDonald 2014 Cochrane systematic review (DOI: 10.1002/14651858.CD003459.pub4).

Allez 2023 (TRIDENT- Part 1)

**Bias**	**Authors' judgement**	**Support for judgement**
Random sequence generation (selection bias)	Low risk	Computer-generated randomization
Allocation concealment (selection bias)	Low risk	The interactive web response system (IWRS) assigned a unique treatment code, which dictated the treatment assignment and matching study drug kit for the subject. The requestor must use his or her own user identification and personal identification number when contacting the IWRS, and then give the relevant subject details to uniquely identify the subject.
Blinding of participants and personnel (performance bias)	Low risk	To maintain the study blind, the study agent container had a label containing the study name and medication number or syringe number. The label did not identify the study agent in the container. The medication number or syringe number was entered in the case report form (CRF) when the drug was dispensed. The study agents were identical in appearance and packaging.
Blinding of outcome assessment (detection bias)	Unclear risk	Planned efficacy and safety evaluations were performed under blinded conditions
Incomplete outcome data (attrition bias)	Unclear risk	Low, balanced and explained attrition (from NCT website)
Selective reporting (reporting bias)	Low risk	Main outcomes reported as per the trial registration
Other bias	Low risk	Balanced baseline characteristics. No other concerns

Allez 2023 (TRIDENT- Part 2)

Bias	Authors' judgement	Support for judgement
Random sequence generation (selection bias)	Low risk	Computer-generated randomization
Allocation concealment (selection bias)	Low risk	The interactive web response system (IWRS) assigned a unique treatment code, which dictated the treatment assignment and matching study drug kit for the subject. The requestor must use his or her own user identification and personal identification number when contacting the IWRS, and then give the relevant subject details to uniquely identify the subject.
Blinding of participants and personnel (performance bias)	Low risk	To maintain the study blind, the study agent container had a label containing the study name and medication number or syringe number. The label did not identify the study agent in the container. The medication number or syringe number was entered in the case report form (CRF) when the drug was dispensed. The study agents were identical in appearance and packaging.
Blinding of outcome assessment (detection bias)	Low risk	Planned efficacy and safety evaluations were performed under blinded conditions
Incomplete outcome data (attrition bias)	Low risk	Slightly more patients left the tesnatilimab groups, however not enough to have likely influenced the outcomes (from NCT website)
Selective reporting (reporting bias)	Low risk	Outcomes reported appropriately and per the trial registration
Other bias	Low risk	Balanced baseline characteristics. No other concerns

Ardizzone 2003

Bias	Authors' judgement	Support for judgement
Random sequence generation (selection bias)	Low risk	Quote: "randomized by a computer-generated list"
Allocation concealment (selection bias)	Unclear risk	Not described

Blinding of participants and personnel (performance bias)	High risk	Quote: "investigator-blind"<br>Quote: "patients and Chief of the Institute (GBP), who supervised the randomization, were aware of the treatment"<br>Quote: "The principal investigator (SA), who was blinded to treatment assignment, evaluated the efficacy of treatment at each scheduled visit and at the end of the study, according to the information provided by other physicians<br>in the investigation team (SB, GM, VB, EC), all of whom were also blinded and evaluated each patient's clinical condition, computed the CDAI on the basis of patient diaries, recorded all the biochemical parameters considered in the<br>study, and monitored compliance and toxicity"
Blinding of outcome assessment (detection bias)	High risk	Quote: "investigator-blind"<br>Quote: "patients and Chief of the Institute (GBP), who supervised the randomization, were aware of the treatment"<br>Quote: "The principal investigator (SA), who was blinded to treatment assignment, evaluated the efficacy of treatment at each scheduled visit and at the end of the study, according to the information provided by other physicians<br>in the investigation team (SB, GM, VB, EC), all of whom were also blinded and evaluated each patient's clinical condition, computed the CDAI on the basis of patient diaries, recorded all the biochemical parameters considered in the<br>study, and monitored compliance and toxicity"
Incomplete outcome data (attrition bias)	Low risk	Quote: "No patients were lost to follow-up. Six patients (three in azathioprine and three in methotrexate group) discontinued treatment due to adverse events. Withdrawal from the trial medication was considered as a treatment failure"
Selective reporting (reporting bias)	Low risk	All expected outcomes were reported
Other bias	Low risk	The study appears to be free of other sources of bias

Arora 1999

Bias	Authors' judgement	Support for judgement
Random sequence generation (selection bias)	Low risk	Patients were randomized through a code directed through the hospital pharmacy
Allocation concealment (selection bias)	Low risk	Centralized pharmacy randomization
Blinding of participants and personnel (performance bias)	Low risk	Double-blind with identical placebo
Blinding of outcome assessment (detection bias)	Low risk	Double-blind with identical placebo
Incomplete outcome data (attrition bias)	Low risk	Twenty-six of 33 patients withdrew from the study (11 from methotrexate, 15<br>from placebo)<br>However, there were predetermined withdrawal criteria: including treatment<br>failure with a flare of Crohn's disease, significant side effects, non-compliance,<br>serious concomitant disease, pregnancy, or presence of Clostridium difficile<br>toxin in the stool Eighteen of 26 patients withdrew due to disease flare<br>The remainder withdrew for other reasons, as described in the published study
Selective reporting (reporting bias)	Low risk	All outcomes were reported
Other bias	Low risk	No other issues

Candy 1995

Bias	Authors' judgement	Support for judgement
Random sequence generation (selection bias)	Unclear risk	Method of randomization not described.Contains sub-stratification for disease site; method not described, but matched in control and intervention groups
Allocation concealment (selection bias)	Unclear risk	Not described

Blinding of participants and personnel (performance bias)	Low risk	Double blinded, control and intervention groups matched for treatment regime (taken once daily), including azathioprine and matching placebo<br>Physician who monitored hematological and biochemical data was independent of the trial<br>Nurse practitioner relayed dose adjustment information to patient, sampled blood, monitored compliance via pill count and assessed symptoms<br>Physical exam physician monitored for adverse events and concomitant therapies taken
Blinding of outcome assessment (detection bias)	Low risk	Double blinded, control and intervention groups matched for treatment regime (taken once daily), including azathioprine and matching placebo<br>Physician who monitored hematological and biochemical data was independent of the trial<br>Nurse practitioner relayed dose adjustment information to patient, sampled blood, monitored compliance via pill count and assessed symptoms<br>Physical exam physician monitored for adverse events and concomitant therapies taken
Incomplete outcome data (attrition bias)	Low risk	Missing outcome data were approximately balanced between intervention and placebo groups across the 2 phases of the trial, with similar reasons, other than phase failure, provided, including side effects, loss to follow-up or requested withdrawal
Selective reporting (reporting bias)	Low risk	All primary and secondary outcomes described in the methods section were reported
Other bias	Low risk	The study appears to be free of other sources of bias

Chen 2020

**Bias**	**Authors' judgement**	**Support for judgement**
Random sequence generation (selection bias)	Low risk	The paper states that "patients were randomised by IRT to a treatment group". Authors were asked for clarification: "At the screening visit, patients were assigned a unique identification number by the interactive response technology (IRT) system. Once enrolled, patients were randomized by the IRT to a treatment group. During the double-blind period, all study investigators, study site personnel, and<br>patients were blinded to each patient’s treatment. To maintain blinding, pre-packaged study drug kits were assigned by the IRT according to the patient’s randomized treatment schedule."

Allocation concealment (selection bias)	Low risk	The study centrally allocates, statiing: "At the screening visit, patients were assigned a unique identification number by the interactive response technology (IRT) system. Once enrolled, patients were randomized by the IRT to a treatment group. During the DB period, all study investigators, study site personnel, and patients were blinded to each patient's treatment."
Blinding of participants and personnel (performance bias)	Low risk	Measures were taken to ensure blinding. The study states that" During the double blinded period, all study investigators, study site personnel, and patients were blinded to each patient's treatment...... To maintain blinding, prepackaged study drug kits were assigned by the IRT according to the patient's randomized treatment schedule. The injections matched."
Blinding of outcome assessment (detection bias)	Low risk	The study took the measures above to ensure blinding of outcome assessment.
Incomplete outcome data (attrition bias)	Low risk	Patient attrition was balanced between the two groups. Reasons are given for all except 1 in each group defined as 'other'. We asked the authors for clarification on what 'other' reasons were: "If a reason for premature discontinuation is not safety related nor due to lack of efficacy, these are reported as free text under the "other" category. Examples include subjects moving to another city or the loss of their insurance."
Selective reporting (reporting bias)	Low risk	Trial registration published and main outcomes reviewed and match the final published study. All expected appropriate outcomes are included.
Other bias	Low risk	No other sources apparent. Baseline characteristics match between groups.

Chen 2025 (NCT03234907)

Bias	Authors' judgement	Support for judgement
Random sequence generation (selection bias)	Unclear risk	"Study treatment blinding was established using an interactive web response system to assign patients to treatment groups according to the randomization schedule": Unclear how the randomisation schedule was procuded.
Allocation concealment (selection bias)	Low risk	"Study treatment blinding was established using an interactive web response system to assign patients to treatment groups according to the randomization schedule"
Blinding of participants and personnel (performance bias)	Unclear risk	"All patients and study personnel, except those involved with drug preparation, were blinded to treatment assignment during the study." Unclear who prepared the drugs and if they had any other role in the study.
Blinding of outcome assessment (detection bias)	Low risk	Clinical outcomes were patient self-reported and endoscopists must have been blinded according to the statement authors have made on blinding.
Incomplete outcome data (attrition bias)	Unclear risk	20% of the placebo group discontinued vs 7% from the vedolizumab group. Withdrawal by patient and due to lack of efficacy elevated in the placebo group.
Selective reporting (reporting bias)	Unclear risk	Clinical pre-planned outcomes appropriate and reported but endoscopic not mentioned in the plan. NCT03234907.
Other bias	High risk	The placebo group has higher prior biologic treatment rates, inadequate response and intolerance to them. It also has higher history of prior CD surgery.

Colombel 2007 (CHARM) - Induction (non-responders)

Bias	Authors' judgement	Support for judgement
Random sequence generation (selection bias)	Low risk	Author states that patients were "*randomised centrally using an interactive voice response system*".
Allocation concealment (selection bias)	Low risk	Author states "Patients, study coordinators, and study investigators were blinded to treatment assignment throughout the blinded portion of the study".

Blinding of participants and personnel (performance bias)	Low risk	Author states that "Patients, study coordinators, and study investigators were blinded to treatment assignment throughout the blinded portion of the study".
Blinding of outcome assessment (detection bias)	Low risk	Author states that "Patients, study coordinators, and study investigators were blinded to treatment assignment throughout the blinded portion of the study".
Incomplete outcome data (attrition bias)	Unclear risk	Attrition is balanced in all three groups but no reason provided for loss in numbers. Authors were contacted for clarification.
Selective reporting (reporting bias)	High risk	The authors have not reported the one primary outcome (clinical remission) as reported in the trial registration for all groups of non-responders
Other bias	Unclear risk	Baseline characteristics not reported for the three groups - placebo, ADA 40mg eow and ADA 40mg weekly. Authors were contacted for clarification.

Colombel 2010 (SONIC)

Bias	Authors' judgement	Support for judgement
Random sequence generation (selection bias)	Unclear risk	Author states that "Randomisation was performed centrally with the use of an adaptive randomisation procedure stratified according to centre, the duration of Crohn's disease (<3 years or ≥ 3 years), and status with respect to the systemic corticosteroid dose (the equivalent of <20 mg or ≥ 20 mg of prednisone daily)."
Allocation concealment (selection bias)	Low risk	Central allocation concealment.
Blinding of participants and personnel (performance bias)	Low risk	Patients were "followed through week 30...with blinding maintained" and each group received infusions (placebo or infliximab) and tablets (placebo or azathioprine).
Blinding of outcome assessment (detection bias)	Low risk	Authors state that "All colonoscopies were videotaped with the use of a standard protocol and interpreted by a single reviewer, who was unaware of study-group assignments and the timing of the procedure (i.e., at baseline or week 26)."
Incomplete outcome data (attrition bias)	Low risk	There are relatively higher rates of attrition with AZA only having 86 out of 162 patients (53.1%), IFX only having 111 out of 166 patients (66.9%), and combination therapy group having 121 out of 180 patients (67.2%). We did not think it was enough to bias outcomes.
Selective reporting (reporting bias)	Low risk	Outcomes reported match the trial registration, and appropriate for the review.
Other bias	Low risk	Baseline characteristics are reported and balanced. No other sources of bias apparent.

D'Haens 1999

Bias	Authors' judgement	Support for judgement
Random sequence generation (selection bias)	Low risk	Randomization was performed centrally by an independent organization (PPD Pharmaco,Austin, TX).
Allocation concealment (selection bias)	Low risk	The infliximab and placebo solutions were prepared by a pharmacist at each site. The investigators, all study personnel, and the patients were blinded to the treatment assignments.
Blinding of participants and personnel (performance bias)	Low risk	The placebo preparation contained 0.1% human serum albumin instead of infliximab and was identical in appearance to the infliximab solution.

Blinding of outcome assessment (detection bias)	Low risk	All biopsy specimens were routinely processed by H&E staining and interpreted by a single, blinded gastrointestinal pathologist in random order. It's not mentioned how the CDAI was assessed.
Incomplete outcome data (attrition bias)	Low risk	Low and balanced attrition with reasons
Selective reporting (reporting bias)	Unclear risk	It's not entirely clear from the methods section but the intended outcomes seem to have been mucosal healing and histological changes and they have been reported. However, there is no trial registration for this study.
Other bias	Low risk	No major imabalances. No other sources of bias.

D'Haens 2008

**Bias**	**Authors' judgement**	**Support for judgement**
Random sequence generation (selection bias)	Low risk	Patients were randomised according to a "computer-generated schedule".
Allocation concealment (selection bias)	Low risk	Person independent of the trial performed the allocation
Blinding of participants and personnel (performance bias)	High risk	Open-label trial
Blinding of outcome assessment (detection bias)	High risk	Open-label trial
Incomplete outcome data (attrition bias)	Low risk	Attrition was accounted for and balanced between both groups.
Selective reporting (reporting bias)	Low risk	Trial registration was NCT00554710 and the study reported according to the primary outcomes stated - proprotion of patients with corticosteroid-free remission and remission without surgical resection.
Other bias	Low risk	Baseline characteristics reported and balanced in both groups. No other apparent sources of bias.

D'Haens 2022 (ADVANCE)

**Bias**	**Authors' judgement**	**Support for judgement**

Random sequence generation (selection bias)	Low risk	Randomly assigned with interactive response technology.
Allocation concealment (selection bias)	Low risk	Randomly assigned with interactive response technology. An unmasked pharmacist (or qualified designee) prepared the intravenous solutions.
Blinding of participants and personnel (performance bias)	Low risk	All patients and study personnel (excluding pharmacists who prepared intravenous solutions) were masked to treatment allocation throughout the study. Saline of equal volume to study drug was administered as the placebo. Placebo and study drug were administered via covered syringes.
Blinding of outcome assessment (detection bias)	Low risk	All patients and study personnel (excluding pharmacists who prepared intravenous solutions) were masked to treatment allocation throughout the study. Saline of equal volume to study drug was administered as the placebo. Placebo and study drug were administered via covered syringes.
Incomplete outcome data (attrition bias)	Low risk	10% attrition in the placebo group, compared to 3% and 5% in the other two. However, we don't think this difference has influenced outcome results to a great extent.
Selective reporting (reporting bias)	Low risk	Outcomes stated and reported per trial registration.
Other bias	Low risk	No differences at baseline. No other concerns.

D'Haens 2022 (MOTIVATE)

Bias	Authors' judgement	Support for judgement
Random sequence generation (selection bias)	Low risk	Randomly assigned with interactive response technology
Allocation concealment (selection bias)	Low risk	Randomly assigned with interactive response technology. An unmasked pharmacist (or qualified designee) prepared the intravenous solutions
Blinding of participants and personnel (performance bias)	Low risk	All patients and study personnel (excluding pharmacists who prepared intravenous solutions) were masked to treatment allocation throughout the

		study. Saline of equal volume to study drug was administered as the placebo. Placebo and study drug were administered via covered syringes.
Blinding of outcome assessment (detection bias)	Low risk	All patients and study personnel (excluding pharmacists who prepared intravenous solutions) were masked to treatment allocation throughout the study. Saline of equal volume to study drug was administered as the placebo. Placebo and study drug were administered via covered syringes.
Incomplete outcome data (attrition bias)	Low risk	14% attrition in the placebo group, compared to 3% and 4% in the other two. However, we don't think this difference has influenced outcome results to a great extent.
Selective reporting (reporting bias)	Low risk	All appropriate outcomes reported as per the trial registration
Other bias	Low risk	No differences at baseline. No other concerns.

Duan 2013

Bias	Authors' judgement	Support for judgement
Random sequence generation (selection bias)	Unclear risk	Authors report that "patients were randomly divided into infliximab group, azathioprine group, and infliximab combined with azathioprine group" but do not clarify how this was performed.
Allocation concealment (selection bias)	Unclear risk	Authors have not described any method of concealment during assignment to treatment groups.
Blinding of participants and personnel (performance bias)	Unclear risk	Authors have not described blinding of participants or personnel.
Blinding of outcome assessment (detection bias)	Unclear risk	No evidence and/or method of blinding the endoscopist or clinician collecting CDAI score or interpreter has been described.
Incomplete outcome data (attrition bias)	Low risk	No evidence of attrition from the original 24 enroled and randomised to each group.
Selective reporting (reporting bias)	Unclear risk	No protocol or trial registration found but study reports on CDAI and endoscopic mucosal healing
Other bias	Low risk	Reported baseline characteristics of no significant difference - age, gender. No other sources of bias are apparent.

Ewe 1993

Bias	Authors' judgement	Support for judgement
Random sequence generation (selection bias)	Unclear risk	Not described
Allocation concealment (selection bias)	Unclear risk	Not described (Quote: "allocation to each group was double blinded")
Blinding of participants and personnel (performance bias)	Unclear risk	Quote: "double blinded"
Blinding of outcome assessment (detection bias)	Unclear risk	Quote: "double blinded"
Incomplete outcome data (attrition bias)	Low risk	Quote: "None of the patients dropped out during the course of the trial"
Selective reporting (reporting bias)	Low risk	The primary outcome (remission) was reported, along with all lab values

Other bias	Low risk	The study appears to be free of other sources of bias

Feagan 1995

Bias	Authors' judgement	Support for judgement
Random sequence generation (selection bias)	Low risk	Computer generated randomization
Allocation concealment (selection bias)	Low risk	Centralized randomization
Blinding of participants and personnel (performance bias)	Low risk	Double-blind<br>The placebo was identical in appearance to the active drug and the investigators<br>were unaware of the treatment assignments
Blinding of outcome assessment (detection bias)	Low risk	Double-blind<br>The placebo was identical in appearance to the active drug and the investigators<br>were unaware of the treatment assignments
Incomplete outcome data (attrition bias)	Low risk	No patients were lost to follow-up<br>The same proportion of patients were withdrawn from treatment prematurely<br>in the two groups (28%)
Selective reporting (reporting bias)	Low risk	All outcomes were reported
Other bias	Low risk	No other issues

Feagan 2008c

Bias	Authors' judgement	Support for judgement
Random sequence generation (selection bias)	Unclear risk	Not described. Authors were contacted for clarification.
Allocation concealment (selection bias)	Unclear risk	Not described. Authors were contacted for clarification.
Blinding of participants and personnel (performance bias)	Low risk	Placebo-controlled trial.
Blinding of outcome assessment (detection bias)	Unclear risk	Not described. Authors were contacted for clarification.
Incomplete outcome data (attrition bias)	Low risk	Low, balanced and explained attrition
Selective reporting (reporting bias)	Low risk	Reported as per the trial registration.
Other bias	Low risk	Balanced baseline characteristics. No other concerns.

Feagan 2014

Bias	Authors' judgement	Support for judgement
Random sequence generation (selection bias)	Low risk	Randomly assigned by computer
Allocation concealment (selection bias)	Low risk	Centralized randomization
Blinding of participants and personnel (performance bias)	Low risk	Double-blind with identically appearing placebo
Blinding of outcome assessment (detection bias)	Low risk	Double-blind with identically appearing placebo
Incomplete outcome data (attrition bias)	Low risk	Six patients withdrew from the study for reasons not related to treatment failure; two patients were assigned to methotrexate (both due to adverse events) and four patients were assigned to placebo (two withdrew consent, one due to adverse event, one lost to follow-up)
Selective reporting (reporting bias)	Low risk	All outcomes were reported
Other bias	Low risk	No other issues

Feagan 2015a (UNITI-1)

Bias	Authors' judgement	Support for judgement
Random sequence generation (selection bias)	Low risk	Randomisation was described as 1:1:1 and performed "centrally with the use of permuted blocks".
Allocation concealment (selection bias)	Low risk	Randomisation was described as 1:1:1 and performed "centrally with the use of permuted blocks".

Blinding of participants and personnel (performance bias)	Low risk	Double-blind placebo controlled trial<br>From the protocol: "To maintain the study blind, the study agent container will have a multilingual label containing the study name, medication number, and reference number, but the label will not identify the study agent in the container. A tear-off label is designed to be separated from the study agent container and attached to the subject's source documents. The medication number will be entered in the CRF when the drug is dispensed. The study agents will be identical in appearance and packaging"
Blinding of outcome assessment (detection bias)	Unclear risk	Not mentioned. Authors were contacted for clarification.
Incomplete outcome data (attrition bias)	Unclear risk	Authors mention attrition was low but the study flow is unclear and not presented in the supplementary figures mentioned in the text
Selective reporting (reporting bias)	Low risk	Reported outcomes match those described in the trial registration.
Other bias	Low risk	Authors reported balanced baseline characteristics in each group. No other sources of bias.

Feagan 2015b (UNITI-2)

**Bias**	**Authors' judgement**	**Support for judgement**
Random sequence generation (selection bias)	Low risk	Randomisation was described as 1:1:1 and performed "centrally with the use of permuted blocks".
Allocation concealment (selection bias)	Low risk	Randomisation was described as 1:1:1 and performed "centrally with the use of permuted blocks".
Blinding of participants and personnel (performance bias)	Low risk	Double-blind placebo controlled trial<br>From the protocol: "To maintain the study blind, the study agent container will have a multilingual<br>label containing the study name, medication number, and reference number, but the label will not identify the study agent in the container. A tear-off label is designed to be separated from the study agent container and attached to the subject's source documents. The medication number will be entered in the CRF when the drug is dispensed. The study agents will be identical in appearance and packaging"
Blinding of outcome assessment (detection bias)	Unclear risk	Not mentioned. Authors were contacted for clarification.
Incomplete outcome data (attrition bias)	Unclear risk	Authors mention attrition was low but the study flow is unclear and not presented in the supplementary figures mentioned in the text
Selective reporting (reporting bias)	Low risk	Reported outcomes match those described in the trial registration.

Other bias	Low risk	Authors reported balanced baseline characteristics in each group. No other sources of bias.

Feagan 2017

Bias	Authors' judgement	Support for judgement
Random sequence generation (selection bias)	Low risk	An interactive response system was used to assign a double-blind investigational product to every patient. Randomisation was stratified by previous exposure to TNF antagonists (yes vs no). A randomisation list was generated using a validated system, which involved a pseudorandom number generator to guarantee the reproducibility of the assignments. This randomisation list was checked by an independent statistician and used to assign randomisation numbers to eligible patients.
Allocation concealment (selection bias)	Low risk	A randomisation list was generated using a validated system, which involved a pseudorandom number generator to guarantee the reproducibility of the assignments. This randomisation list was checked by an independent statistician and used to assign randomisation numbers to eligible patients. Access to the randomisation code was controlled and documented.
Blinding of participants and personnel (performance bias)	Low risk	All people directly involved in the conduct and analysis of the trial (including patients, investigators, and study personnel) were fully masked to the treatment allocation before the week 12 database lock. To maintain masking, study drug packaging was identical irrespective of treatment and was only distinguishable by medication number, which was managed by the central randomisation centre. Both risankizumab and placebo appeared as clear solutions.
Blinding of outcome assessment (detection bias)	Low risk	All people directly involved in the conduct and analysis of the trial (including patients, investigators, and study personnel) were fully masked to the treatment allocation before the week 12 database lock. To maintain masking, study drug packaging was identical irrespective of treatment and was only distinguishable by medication number, which was managed by the central randomisation centre. Both risankizumab and placebo appeared as clear solutions.
Incomplete outcome data (attrition bias)	Low risk	Attrition was low and balanced
Selective reporting (reporting bias)	Low risk	All relevant efficacy and safety outcomes reported as per the trial registration.

Other bias	Low risk	Baseline characteristics are balanced accross the study arms.<br><br>No other sources of bias apparent

Ferrante 2024 (VIVID-1)

Bias	Authors' judgement	Support for judgement
Random sequence generation (selection bias)	Low risk	"randomly assigned with an interactive web response system"
Allocation concealment (selection bias)	Low risk	"An unmasked pharmacist (or qualified designee) prepared the intravenous solutions. Study investigators, study site personnel, and participants were masked to treatment allocation."
Blinding of participants and personnel (performance bias)	Low risk	"All treatments were masked. To maintain masking, placebo was administered as appropriate, either intravenously, subcutaneously, or both, using a double-dummy design"
Blinding of outcome assessment (detection bias)	Low risk	Outcome assessment for patient reported clinical outcomes and endoscopies was blinded (central reading).
Incomplete outcome data (attrition bias)	Unclear risk	1152 patients were randomised but 87 were excluded before receiving medication and it's not mentioned how many were excluded per group. 7% of those who received treatment left the placebo group, 2.7% the mirikizumab group and 3.4% the ustekinumab group. The rates of participants discontinuing due to adverse events vary.
Selective reporting (reporting bias)	Low risk	No major concerns based on the trial registration and supplementary material amendments to the protocol section. NCT03926130.
Other bias	Low risk	No concerns

Ghosh 2003

Bias	Authors' judgement	Support for judgement
Random sequence generation (selection bias)	Low risk	"Randomly assigned to one of four treatment regimens according to a computer-generated, sitestratified,block randomization schedule".
Allocation concealment (selection bias)	Low risk	"Randomly assigned to one of four treatment regimens according to a computer-generated, sitestratified,block randomization schedule".

Blinding of participants and personnel (performance bias)	Low risk	The authors state that neither the study personnel nor the patients were aware of the interventions.
Blinding of outcome assessment (detection bias)	Low risk	The authors state that neither the study personnel nor the patients were aware of the interventions.
Incomplete outcome data (attrition bias)	Low risk	Attrition in each group was the same with reasons being provided. Of the 244 patients who received at least one dose of the assignments 27 withdrew from the study before completing 12 weeks: 10 in the placebo group, 6 in the single natalizumab group, 5 in the group given two 3mg natalizumab infusions, and 6 in the two 6mg natalizumab infusion.
Selective reporting (reporting bias)	Unclear risk	Based on the method section, the study reported CDAI results. However, there is no trial registration or protocol we could find.
Other bias	Low risk	Authors reported balanced baseline characteristics in each group. No other sources of bias.

Gordon 2001

Bias	Authors' judgement	Support for judgement
Random sequence generation (selection bias)	Unclear risk	It is unclear how the patients were randomised. Authors were contacted for clarification.
Allocation concealment (selection bias)	Low risk	"Individual randomization concealment codes were held by the trial's sponsor and each hospital's pharmacy for emergency use, and none were opened during the study".
Blinding of participants and personnel (performance bias)	Low risk	Author states "Investigators and patients remained blinded to the randomization codes until data analysis was complete".
Blinding of outcome assessment (detection bias)	Low risk	Author states "Investigators and patients remained blinded to the randomization codes until data analysis was complete".
Incomplete outcome data (attrition bias)	Low risk	All patients are accounted for and attrition rate is balanced between both groups. Reasons for withdrawal were clear.
Selective reporting (reporting bias)	Unclear risk	According to method section, study reported the outcomes in the results - CDAI score for both groups. However, we could not find a trial registration or protocol for this trial.
Other bias	Low risk	Baseline characteristics are reported and balanced. No other source of bias apparent

Hanauer 2002 - ACCENT I (non-responders)

Bias	Authors' judgement	Support for judgement
Random sequence generation (selection bias)	Low risk	Authors mention random assignment via adaptive randomisation voice response system
Allocation concealment (selection bias)	Low risk	Authors mention "allocation of patients to a treatment group was done with an adaptive stratified design" and "allocate patients centrally to treatment based on the current balance of treatment groups within each stratum".
Blinding of participants and personnel (performance bias)	Low risk	Authors state "Neither the patients nor study investigators were aware of the treatment assignment" and "pharmacist prepared the infusion (infliximab [Remicade] or an identically appearing placebo".
Blinding of outcome assessment (detection bias)	Low risk	"Neither the patients nor study investigators were aware of the treatment assignment" and "pharmacist prepared the infusion (infliximab [Remicade] or an identically appearing placebo".
Incomplete outcome data (attrition bias)	Unclear risk	This group was only followed-up for safety. Safety data is presented for the entire cohort and not separately throughout the end of the study.
Selective reporting (reporting bias)	Unclear risk	Safety data and primary efficacy data is given for the entire cohort and not separately. The trial registration information is very unclear.
Other bias	Unclear risk	Baseline characteristics reported were summarised for all patients, responders and non-responders.

Hanauer 2006 (CLASSIC I)

Bias	Authors' judgement	Support for judgement
Random sequence generation (selection bias)	Low risk	Patients were randomised using an "intercative voice response system".
Allocation concealment (selection bias)	Low risk	Authors states "pharmacist blinded to the identity of the study drug prepared each injection of adalimumab or an identical-appearing placebo"
Blinding of participants and personnel (performance bias)	Low risk	"patients, study coordinators, and study investigators were all blinded to treatment assignment"
Blinding of outcome assessment (detection bias)	Low risk	"patients, study coordinators, and study investigators were all blinded to treatment assignment"
Incomplete outcome data (attrition bias)	Low risk	Attrition is balanced between all 4 groups and reasons provided for each group accounts for the loss in numbers.
Selective reporting (reporting bias)	Unclear risk	Author reported according to method section on proportion of patients achieving clinical response and remission. No trial registration or protocol found.
Other bias	Low risk	All baseline characteristics were balanced. No other concerns.

Hanauer 2021 (VOLTAIRE-CD)

Bias	Authors' judgement	Support for judgement
Random sequence generation (selection bias)	Low risk	Author states "randomisation number-generating system".
Allocation concealment (selection bias)	Low risk	Author states "interactive response technology system assigned each patient a unique medication number for each drug administration"
Blinding of participants and personnel (performance bias)	Low risk	Authors state "All personnel directly involved in the conduct of the trial and patients remained masked to treatment allocation until after database lock at week 24. Unmasked trial personnel receiving, handling,and administering the trial medication were not involved in any other trial assessments or procedures."
Blinding of outcome assessment (detection bias)	Low risk	Authors state "All personnel directly involved in the conduct of the trial and patients remained masked to treatment allocation until after database lock at week 24" so authors contacted to confirm outcome assessors were unaware of treatment assignments.

Incomplete outcome data (attrition bias)	Low risk	Attrition in both groups accounted for and balanced, with adequate reasons provided respectively.
Selective reporting (reporting bias)	Low risk	According to trial registration and method section, authors reported relevant endpoint outcomes -clinical response and remission (CDAI scores).
Other bias	Low risk	Baseline characteristics reported for and balanced in both groups. No other apparent sources of bias.

Hart 2025 (GRAVITI)

Bias	Authors' judgement	Support for judgement
Random sequence generation (selection bias)	Low risk	"Computer-generated randomization schedule"
Allocation concealment (selection bias)	Low risk	Interactive web response system (IWRS) which assigns a unique intervention code, which dictates the intervention assignment andmatching study intervention kit for the participant. The requestor uses their own user identification and personal identification number when contacting the IWRS, and is then given the relevant participant details to uniquely identify the participant
Blinding of participants and personnel (performance bias)	Low risk	Treatment assignment remained blinded to the study sites and participants until the last participant completed the Week 48 evaluations. To maintain the study blind, the study intervention container had a label containing the study name, study intervention number, and reference number. The study intervention number was entered in the eCRF when the study intervention was dispensed. Each active study intervention and its matching placebo were identical in appearance and were packaged in identical containers. All participants received the same device(s), which were either active or matching placebo at 4-week intervals in order to maintain treatment blinding.
Blinding of outcome assessment (detection bias)	Low risk	Clinicians and patients were blinded for the assessment and self-assessment of clinical outcomes. Video endoscopies were assessed by a central facility that was blinded to treatment group and visit.
Incomplete outcome data (attrition bias)	Unclear risk	12/17 had discontinued placebo treatment by week 12 compared to 2/115 and 1/115 in the two guselkumab groups. Reasons per group are unclear but most common reason was withdrawal by participant (2.0% total).
Selective reporting (reporting bias)	Low risk	Pre-planned outcomes reported appropriately per trial registration. NCT05197049. Clinical response and endocopic remission do not appear in the NCT study plan but reported in results.

Other bias	Low risk	No concerns

Jairath 2025 (RELIEVE UCCD)

**Bias**	**Authors' judgement**	**Support for judgement**
Random sequence generation (selection bias)	Unclear risk	No details (abstract only publication)
Allocation concealment (selection bias)	Unclear risk	No details (abstract only publication)
Blinding of participants and personnel (performance bias)	Low risk	Double-blind study, no more details (abstract only publication)
Blinding of outcome assessment (detection bias)	Unclear risk	No details (abstract only publication)
Incomplete outcome data (attrition bias)	Unclear risk	No complete data on attrition per group.
Selective reporting (reporting bias)	Unclear risk	As this is an abstract only publication many of the outcomes have not been reported.
Other bias	Low risk	No concerns but males overrespresented.

Lemann 2006

Bias	Authors' judgement	Support for judgement
Random sequence generation (selection bias)	Low risk	Author stated "randomization was performed centrally, using permutation tables". Author contacted and confirmed that the permutation tables used were provided in J Lellouch and P Lazar, Méthodes statistiques en expérimentation biologique. Flammarion Médecine Sciences 1974, pp 259 and 258.

Allocation concealment (selection bias)	Low risk	Authors stated that randomisation was performed centrally suggesting concealment.<br>Author clarification: "When an investigator wanted to include a new patient and to obtain the treatment to be given to this patient, the investigator sent me by fax in my lab a randomization request sheet including patient's identification (3 first letters last name, 2 first letters first name), center, stratum, and the major inclusion criteria. If all inclusion criteria were satisfied, I updated the first free line of the list of randomization of the stratum within the center with patient's identification, investigator's name, date of randomization, the first free treatment number in the list of treatment numbers corresponding to assigned treatment through randomization. The list of treament numbers per treatment was common to my center and to Shering delivery service.<br>I completed the randomization request sheet with patient number within the stratum and center,<br>treatment number and sent it to investigator's center. I sent in parallel to Shering delivery service the same data plus treatment assigned, placebo or Remicade, allowing this service to check coherence with treatment number and name.<br><br>Shering delivery service sent to the pharmacy of the investigator's hospital the blind treatment with<br>patients' identification and treatment number.<br>If at least one inclusion criteria was missing or not satisfied, I sent back to the investigator the<br>randomization request sheet explaining why treatment allocation to his/her patient could not be performed. Thus, I had no contact with patients, I had no contact with investigators except through the randomization request sheet, I had knowledge of the treatment of a new patient when looking at the randomization list of the stratum and center after reception of the randomization request sheet."
Blinding of participants and personnel (performance bias)	Low risk	Author stated that "Neither the patients nor the study investigators were aware of the treat-ment<br>assigned" and that "identical placebo" to infliximab (Remicade) was administered.
Blinding of outcome assessment (detection bias)	Low risk	Author contacted and confirmed that the outcome assessors were not aware of patients's<br>treatment
Incomplete outcome data (attrition bias)	Low risk	Attrition was accounted for and balanced in both groups. Reasons for attrition were provided adequately and did not affect outcomes
Selective reporting (reporting bias)	Unclear risk	No trial registration or published protocol found. Authors report primary outcome as stated in their method section - clinical remission with CDAI score < 150 off steroids at week

		24.
Other bias	Low risk	Baseline characteristics have been reported and are overall balanced in both treatment groups. No other sources of bias apparent.

Loftus 2023 U-EXCEED

Bias	Authors' judgement	Support for judgement
Random sequence generation (selection bias)	Low risk	"Patients were randomly assigned through Web-based interactive response technology"
Allocation concealment (selection bias)	Low risk	"Patients were randomly assigned through Web-based interactive response technology"
Blinding of participants and personnel (performance bias)	Low risk	Described as double-blind placebo controlled.
Blinding of outcome assessment (detection bias)	Unclear risk	No details on outcome assessment blinding, apart from endoscopy. "Endoscopic scores were centrally read by qualified gastroenterologists that were blinded to the site, subject number, timepoint, date of the endoscopy, and study treatment."
Incomplete outcome data (attrition bias)	Low risk	Equal attrition and reasons reported and equal between groups.
Selective reporting (reporting bias)	Low risk	Outcomes reported appropriately and as per the trial registration.
Other bias	Low risk	Baseline characteristics similar between groups.

Loftus 2023 U-EXCEL

Bias	Authors' judgement	Support for judgement
Random sequence generation (selection bias)	Low risk	"Patients were randomly assigned through Web-based interactive response technology"
Allocation concealment (selection bias)	Low risk	"Patients were randomly assigned through Web-based interactive response technology"
Blinding of participants and personnel (performance bias)	Low risk	Described as double-blind placebo controlled.
Blinding of outcome assessment (detection bias)	Unclear risk	No details on outcome assessment blinding, apart from endoscopy. "Endoscopic scores were centrally read by qualified gastroenterologists that were blinded to the site, subject number, timepoint, date of the endoscopy, and study treatment."
Incomplete outcome data (attrition bias)	Low risk	Equal attrition and reasons reported and equal between groups.
Selective reporting (reporting bias)	Low risk	Outcomes reported appropriately and as per the trial registration.
Other bias	Low risk	Baseline characteristics similar between groups.

Mantzaris 2004

Bias	Authors' judgement	Support for judgement
Random sequence generation (selection bias)	Low risk	Computer-generated
Allocation concealment (selection bias)	Unclear risk	Sealed opaque envelopes
Blinding of participants and personnel (performance bias)	High risk	Single-blind<br>All assessors were blinded
Blinding of outcome assessment (detection bias)	High risk	Single-blind<br>All assessors were blinded
Incomplete outcome data (attrition bias)	Low risk	Seven patients were withdrawn from the study: 3 in each group because of relapse of CD and 1 on AZA because of severe leucopenia<br>Intention to treat analyses were performed
Selective reporting (reporting bias)	Unclear risk	All outcomes described were reported<br>No access to protocol or methods section
Other bias	Low risk	The study appears to be free of other sources of bias

Mate-Jimenez 2000

Bias	Authors' judgement	Support for judgement
Random sequence generation (selection bias)	Unclear risk	Not adequately described
Allocation concealment (selection bias)	Unclear risk	Not adequately described
Blinding of participants and personnel (performance bias)	High risk	The use of blinding was not described in the manuscript<br>We assume that blinding was not used
Blinding of outcome assessment (detection bias)	High risk	The use of blinding was not described in the manuscript<br>We assume that blinding was not used
Incomplete outcome data (attrition bias)	Low risk	Missing data was accounted for; 24/72 patients withdrew in the first 30 weeks, reasons described (side effects and treatment failure)<br>Intention to treat analysis used
Selective reporting (reporting bias)	Unclear risk	Primary outcome described in the methods section was reported, as well as a number of other post hoc outcomes
Other bias	Low risk	The study appears to be free of other sources of bias

Matsumoto 2016 - DIAMOND

Bias	Authors' judgement	Support for judgement
Random sequence generation (selection bias)	Low risk	"Randomization was done centrally at the Clinical Research Centre in Keio University with the use of an adaptive randomization procedure."
Allocation concealment (selection bias)	Low risk	"Randomization was done centrally at the Clinical Research Centre in Keio University with the use of an adaptive randomization procedure."
Blinding of participants and personnel (performance bias)	High risk	Open-label study
Blinding of outcome assessment (detection bias)	High risk	Open-label study
Incomplete outcome data (attrition bias)	Low risk	Numbers and reasons for attrition given per group and are balanced.
Selective reporting (reporting bias)	Low risk	Outcomes reported appropriately and per trial registration (UMIN registration No. 000005146)
Other bias	Low risk	No baseline characteristic imbalances between groups.

Oren 1997

Bias	Authors' judgement	Support for judgement
Random sequence generation (selection bias)	Unclear risk	Not described
Allocation concealment (selection bias)	Unclear risk	Not described
Blinding of participants and personnel (performance bias)	Unclear risk	Quote: "double blind"<br>Quote: "The investigators were blinded to treatment assignment"
Blinding of outcome assessment (detection bias)	Unclear risk	Quote: "double blind"<br>Quote: "The investigators were blinded to treatment assignment"
Incomplete outcome data (attrition bias)	Low risk	Attrition over the 9 month study were 13/26 for the methotrexate group, 9/32 for the 6-mercaptopurine group and 5/26 for the placebo. Data were analyzed by intention to treat and the method for dealing with missing data was outlined
Selective reporting (reporting bias)	Low risk	The primary outcome (remission) was reported, along with all secondary outcomes
Other bias	Low risk	The study appears to be free of other sources of bias

Panaccione 2024 (GALAXI-2)

Bias	Authors' judgement	Support for judgement
Random sequence generation (selection bias)	Unclear risk	No details (poster/abstract only)
Allocation concealment (selection bias)	Unclear risk	No details (poster/abstract only)
Blinding of participants and personnel (performance bias)	Low risk	Double-blind. No further details (poster/abstract only)
Blinding of outcome assessment (detection bias)	Unclear risk	No details (poster/abstract only)
Incomplete outcome data (attrition bias)	Unclear risk	No details (poster/abstract only)
Selective reporting (reporting bias)	Unclear risk	Outcomes reporting incomplete (poster/abstract only). NCT03466411.

Other bias	Low risk	No concerns but there is sex imbalance.

Panaccione 2024 (GALAXI-3)

Bias	Authors' judgement	Support for judgement
Random sequence generation (selection bias)	Unclear risk	No details (poster/abstract only)
Allocation concealment (selection bias)	Unclear risk	No details (poster/abstract only)
Blinding of participants and personnel (performance bias)	Low risk	Double-blind. No further details (poster/abstract only)
Blinding of outcome assessment (detection bias)	Unclear risk	No details (poster/abstract only)
Incomplete outcome data (attrition bias)	Unclear risk	No details (poster/abstract only)
Selective reporting (reporting bias)	Unclear risk	Outcomes reporting incomplete (poster/abstract only). NCT03466411.
Other bias	Low risk	No concerns but there is sex imbalance.

Panes 2017 Induction

Bias	Authors' judgement	Support for judgement
Random sequence generation (selection bias)	Low risk	Assignment of subject identification number and study drug were managed by a tele-randomisation system, by which the subject was enrolled online or via a telephone call.<br>In the induction study, patients were stratified by whether or not they had previous exposure to TNFi.
Allocation concealment (selection bias)	Low risk	Assignment of subject identification number and study drug were managed by a tele-randomisation system, by which the subject was enrolled online or via a telephone call.<br>Treatment randomisation information remained confidential and was not released to the investigator or study staff until the conclusion of the studies.

Blinding of participants and personnel (performance bias)	Low risk	Study treatment was blinded to patients, investigators and the sponsor.
Blinding of outcome assessment (detection bias)	Low risk	Study treatment was blinded to patients, investigators and the sponsor.
Incomplete outcome data (attrition bias)	Low risk	Balanced and explained attrition between groups.
Selective reporting (reporting bias)	Low risk	All outcomes reported per the trial registration (NCT01393626).<br>The data for the 16 patients randomised to the 15mg group are not reported for efficacy but only for safety after the authors explained that "due to the small number of patients treated with tofacitinib 15 mg twice daily, the data for this group were not included for comparison with placebo in the efficacy analyses but were included in the safety analyses"
Other bias	Low risk	Slight gender imbalance but all other characteristics balanced.<br>No other concerns.

Present 1999

Bias	Authors' judgement	Support for judgement
Random sequence generation (selection bias)	Unclear risk	Randomization was performed by an independent organization (PPD Pharmaco, Austin, Tex.) using a stratified treatment assignment, methods not described
Allocation concealment (selection bias)	Low risk	Central allocation by PPD
Blinding of participants and personnel (performance bias)	Low risk	Double blind study, the placebo was identical in appearance to the infliximab solution
Blinding of outcome assessment (detection bias)	Unclear risk	Not mentioned
Incomplete outcome data (attrition bias)	Low risk	Dropouts were reported and distributed evenly across treatment groups
Selective reporting (reporting bias)	High risk	Trial registration NCT00004941, however outcomes were not defined and primary outcome (30% draining of fistulas) used was uncommon
Other bias	Low risk	The study appears to be free of other sources of bias

Reinisch 2008

Bias	Authors' judgement	Support for judgement
Random sequence generation (selection bias)	Low risk	Quote: " randomization was performed using a validated interactive voice response randomization system that automated the random assignment of patient numbers to randomization numbers"
Allocation concealment (selection bias)	Low risk	Quote: " randomization was performed using a validated interactive voice response randomization system that automated the random assignment of patient numbers to randomization numbers"
Blinding of participants and personnel (performance bias)	Low risk	Quote: " A double-blind, double-dummy design was used to overcome the different forms of everolimus and azathioprine"
Blinding of outcome assessment (detection bias)	Low risk	Quote: " A double-blind, double-dummy design was used to overcome the different forms of everolimus and azathioprine"

Incomplete outcome data (attrition bias)	Unclear risk	A large proportion of the patients initially enrolled (n = 144) appear not to be in remission after 3 months (n = 48). Furthermore, 54 patients withdrew from the study for various reasons. However, intention to treat was followed for the remaining 96 patients
Selective reporting (reporting bias)	Low risk	All expected outcomes were reported
Other bias	Low risk	The study appears to be free of other sources of bias

Rutgeerts 2006

Bias	Authors' judgement	Support for judgement
Random sequence generation (selection bias)	Low risk	"Centralized randomization procedure using an interactive voice-response system"
Allocation concealment (selection bias)	Unclear risk	No specific details on allocation concealment.
Blinding of participants and personnel (performance bias)	Low risk	Study is described as 'double-blind'. "Solutions of onercept and placebo were identical in appearance, and labeling and packaging were prepared to preserve the blinded nature of the study."
Blinding of outcome assessment (detection bias)	Unclear risk	No details on outcome assessment blinding.
Incomplete outcome data (attrition bias)	Unclear risk	No details on attrition numbers and reasons per randomised group.
Selective reporting (reporting bias)	Unclear risk	No details on trial registration.
Other bias	Low risk	Baseline characteristics were well-balanced between groups.

Rutgeerts 2012 (EXTEND)

Bias	Authors' judgement	Support for judgement
Random sequence generation (selection bias)	Unclear risk	Not mentioned
Allocation concealment (selection bias)	Unclear risk	Not mentioned
Blinding of participants and personnel (performance bias)	Low risk	Described as double-blinded
Blinding of outcome assessment (detection bias)	Low risk	Author states "ileocolonoscopy procedures were recorded and subsequently evaluated by an independent, blinded central reviewer".
Incomplete outcome data (attrition bias)	Low risk	Attrition was accounted for and balanced in both groups, with adequate reasons provided.
Selective reporting (reporting bias)	Low risk	Authors reported relevant outcomes as planned per the trial registration
Other bias	Low risk	Baseline characteristics reported and balanced between both groups. No other apparent sources of bias.

Sandborn 2001a

Bias	Authors' judgement	Support for judgement
Random sequence generation (selection bias)	Low risk	Author stated "computer-generated randomization scheme performed the randomisation centrally".
Allocation concealment (selection bias)	Low risk	Central allocation by a statistician
Blinding of participants and personnel (performance bias)	Low risk	Described as double-blind and placebo-controlled
Blinding of outcome assessment (detection bias)	Low risk	Pharmacists prepared the IV infusions so the inference is outcomes assessment was blind
Incomplete outcome data (attrition bias)	Low risk	Attrition was accounted for, balanced in both groups and reasons provided adequately.
Selective reporting (reporting bias)	Unclear risk	All relevant outcomes reported, however we could not find a trial registration or protocol for this trial
Other bias	Low risk	Baseline characteristics reported and balanced in both groups. No other apparent sources of bias.

Sandborn 2001b

Bias	Authors' judgement	Support for judgement
Random sequence generation (selection bias)	Low risk	Author stated "randomization schedule was generated by a statistician at Immunex Corporation".
Allocation concealment (selection bias)	Low risk	Author stated "patients were assigned to treatment group according to the schedule maintained at Immunex Corporation".
Blinding of participants and personnel (performance bias)	Low risk	Double-blind, placebo-controlled
Blinding of outcome assessment (detection bias)	Unclear risk	Not mentioned
Incomplete outcome data (attrition bias)	Low risk	Attrition was accounted for with adequate reasons provided for each group. Balanced in both groups.
Selective reporting (reporting bias)	Unclear risk	No protocol or trial registration found. According to the method section author reported on stated primary outcome - proportion of patients with clinical response and remission.

Other bias	Low risk	Baseline characteristics reported and balanced in both groups. No other apparent sources of bias.

Sandborn 2005a (ENACT 1)

Bias	Authors' judgement	Support for judgement
Random sequence generation (selection bias)	Low risk	Stated as randomised centrally
Allocation concealment (selection bias)	Low risk	"Centrally allocated"
Blinding of participants and personnel (performance bias)	Low risk	Patients and investigators were unaware of treatment assignments, stated as double blinded.
Blinding of outcome assessment (detection bias)	Low risk	Patients and investigators were unaware of treatment assignments, stated as double blinded.
Incomplete outcome data (attrition bias)	Low risk	No attrition
Selective reporting (reporting bias)	Unclear risk	Trial registration has no outcomes. However, clincically relevant outcomes have been reported.
Other bias	Low risk	No other sources apparent. No baseline characteristic imbalance.

Sandborn 2007a

Bias	Authors' judgement	Support for judgement
Random sequence generation (selection bias)	Low risk	Author stated randomisation was achieved using a "central computer-generated scheme".
Allocation concealment (selection bias)	Low risk	Authors stated that "Patient numbers were centrally assigned by an interactive voice-response systemin consecutive order. The system provided access to blinded patient treatment information for medical emergencies only".
Blinding of participants and personnel (performance bias)	Low risk	Participants, personnel and outcome assessors were blinded
Blinding of outcome assessment (detection bias)	Low risk	Participants, personnel and outcome assessors were blinded
Incomplete outcome data (attrition bias)	Low risk	Attrition accounted for in both groups and balanced. Adequate explanations provided for both groups' attritions.

Selective reporting (reporting bias)	Low risk	No published protocol found. But according to trial registration and method section primary end-point was "proportion of patients with remission at week 4". This was reported accordingly.
Other bias	Low risk	Baseline characteristics reported and balanced in both groups. No other apparent sources of bias.

Sandborn 2007c (PRECISE 1)

Bias	Authors' judgement	Support for judgement
Random sequence generation (selection bias)	Low risk	Authors state randomisation was "perfomed centrally"
Allocation concealment (selection bias)	Unclear risk	Not mentioned.
Blinding of participants and personnel (performance bias)	Low risk	Described as double-blind
Blinding of outcome assessment (detection bias)	Low risk	Authors state "Data were collected and analyzed by ICON Clinical Research".
Incomplete outcome data (attrition bias)	Low risk	Attrition accounted for and balanced in both groups. Adequate reasons provided for withdrawals.
Selective reporting (reporting bias)	Low risk	Outcomes reported per the trial registration.
Other bias	Low risk	Baseline characteristics reported for and balanced in both groups. No other apparent sources of bias.

Sandborn 2008 (Population 1)

Bias	Authors' judgement	Support for judgement
Random sequence generation (selection bias)	Low risk	Computer-generated randomisation
Allocation concealment (selection bias)	Low risk	Centrally allocated
Blinding of participants and personnel (performance bias)	Low risk	Double-blind placebo controlled
Blinding of outcome assessment (detection bias)	Unclear risk	Not mentioned
Incomplete outcome data (attrition bias)	Low risk	Attrition numbers were accounted for and balanced in all four groups of poulation 1.
Selective reporting (reporting bias)	Low risk	Authors have reported relevant outcomes for population 1 according to the trial registration.

Other bias	Low risk	Baseline characteristics reported and balanced for patients in population 1. No other apparent sources of bias.

Sandborn 2011

Bias	Authors' judgement	Support for judgement
Random sequence generation (selection bias)	Low risk	Authors stated "Randomization was performed centrally according to a computer-generated scheme"
Allocation concealment (selection bias)	Low risk	Central allocation
Blinding of participants and personnel (performance bias)	Low risk	Double-blind study
Blinding of outcome assessment (detection bias)	Unclear risk	Not mentioned
Incomplete outcome data (attrition bias)	Low risk	Attrition was accounted and balanced in both groups, and adequate reasons provided for both.
Selective reporting (reporting bias)	Unclear risk	No published protocol found. Authors have reported relevant outcomes - clinical response (CDAI scores) at relevant intervals.
Other bias	Low risk	Baseline characteristics reported and balanced for patients in both groups. No other apparent sources of bias.

Sandborn 2012 (CERTIFI) - Induction

Bias	Authors' judgement	Support for judgement
Random sequence generation (selection bias)	Low risk	The sponsor confirmed that "Randomizations were performed through a central randomization centre using an interactive voice response system (IVRS)"
Allocation concealment (selection bias)	Low risk	The sponsor confirmed that "The pharmacist (or other appropriately licensed and authorized drugpreparation personnel) was unblinded to subject treatment at Week 0. If drug preparation at Week 0 was delegated to a non-pharmacist, such as a nurse, that individual could not be involved in any other aspects of the trial or in the care of trial subjects."

Blinding of participants and personnel (performanc bias)	Low risk	Study sponsor response after our enquiry: The pharmacist (or other appropriately licensed and authorized drug-preparation personnel) was unblinded to subject treatment at Week 0. The treatment at Week 8 and Week 16 was blinded to all study personnel, including the pharmacist. If drug preparation at Week 0 was delegated to a non-pharmacist, such as a nurse, that individual could not be involved in any other aspects of the trial or in the care of trial subjects. Placebo administrations had the same appearance as the respective ustekinumab administrations. Because 1 treatment arm was to receive ustekinumab 270 mg as three 90 mg SC injections at Week 8, all subjects were to receive 3 SC injections of study agent (ie, 3 ustekinumab 90 mg SC injections, 1 ustekinumab 90 mg SC plus 2 SC placebo injections, or 3 SC placebo injections) at Week 8 to protect the blind. Under no circumstances were the unblinded personnel to reveal the treatment assignment for a subject.
Blinding of outcome assessment (detection bias)	Low risk	Study sponsor response after our enquiry: The pharmacist (or other appropriately licensed and authorized drug-preparation personnel) was unblinded to subject treatment at Week 0. The treatment at Week 8 and Week 16 was blinded to all study personnel, including the pharmacist. If drug preparation at Week 0 was delegated to a non-pharmacist, such as a nurse, that individual could not be involved in any other aspects of the trial or in the care of trial subjects. Placebo administrations had the same appearance as the respective ustekinumab administrations. Because 1 treatment arm was to receive ustekinumab 270 mg as three 90 mg SC injections at Week 8, all subjects were to receive 3 SC injections of study agent (ie, 3 ustekinumab 90 mg SC injections, 1 ustekinumab 90 mg SC plus 2 SC placebo injections, or 3 SC placebo injections) at Week 8 to protect the blind. Under no circumstances were the unblinded personnel to reveal the treatment assignment for a subject.
Incomplete outcome data (attrition bias)	Low risk	According to the flowchart of Supplementary Figure 2 (S2), attrition was balanced in all groups with adequate reasons provided for loss in numbers shown in Tables S3A and S3B.
Selective reporting (reporting bias)	Low risk	Authors reported outcomes according to trial registration - clinical response and clinical remission (CDAI scores) at relevant intervals.
Other bias	Low risk	Baseline characteristics reported and balanced for patients in all groups. No other apparent sources of bias.

Sandborn 2012 (CERTIFI) non-responders

Bias	Authors' judgement	Support for judgement
Random sequence generation (selection bias)	Low risk	The sponsor confirmed that "Randomizations were performed through a central randomization centre using an interactive voice response system (IVRS)"
Allocation concealment (selection bias)	Low risk	The sponsor confirmed that "The pharmacist (or other appropriately licensed and authorized drugpreparation personnel) was unblinded to subject treatment at Week 0. If drug preparation at Week 0 was delegated to a non-pharmacist, such as a nurse, that individual could not be involved in any other aspects of the trial or in the care of trial subjects."
Blinding of participants and personnel (performance bias)	Low risk	The authors confirmed that "The pharmacist (or other appropriately licensed and authorized drugpreparation personnel) was unblinded to subject treatment at Week 0. The treatment at Week 8 and Week 16 was blinded to all study personnel, including the pharmacist. If drug preparation at Week 0 was delegated to a non-pharmacist, such as a nurse, that individual could not be involved in any other aspects<br>of the trial or in the care of trial subjects. Placebo administrations had the same appearance as the respective ustekinumab administrations. Because 1 treatment arm was to receive ustekinumab 270 mg as three 90 mg SC injections at Week 8, all subjects were to receive 3 SC injections of study agent (ie, 3 ustekinumab 90 mg SC injections, 1 ustekinumab 90 mg SC plus 2 SC placebo injections, or 3 SC placebo injections) at Week 8 to protect the blind. Under no circumstances were the unblinded personnel<br>to reveal the treatment assignment for a subject."
Blinding of outcome assessment (detection bias)	Low risk	The authors confirmed that "The pharmacist (or other appropriately licensed and authorized drugpreparation personnel) was unblinded to subject treatment at Week 0. The treatment at Week 8 and Week 16 was blinded to all study personnel, including the pharmacist. If drug preparation

		at Week 0 was delegated to a non-pharmacist, such as a nurse, that individual could not be involved in any other aspects of the trial or in the care of trial subjects. Placebo administrations had the same appearance as the respective ustekinumab administrations. Because 1 treatment arm was to receive ustekinumab 270 mg as three 90 mg SC injections at Week 8, all subjects were to receive 3 SC injections of study agent (ie, 3 ustekinumab 90 mg SC injections, 1 ustekinumab 90 mg SC plus 2 SC placebo injections, or 3 SC placebo injections) at Week 8 to protect the blind. Under no circumstances were the unblinded personnel<br>to reveal the treatment assignment for a subject."
Incomplete outcome data (attrition bias)	Low risk	Balanced and explained attrition between the two groups, per sponsor's response
Selective reporting (reporting bias)	Unclear risk	Outcome data for the non-responders are not clearly desrcribed but were provided to us by the study sponsor.
Other bias	Low risk	No baseline differences between groups per sponsor's response. No other concerns.

Sandborn 2013 (GEMINI II) - Induction

**Bias**	**Authors' judgement**	**Support for judgement**
Random sequence generation (selection bias)	Low risk	Author states "Randomization was computer-generated and was performed at a<br>central location."
Allocation concealment (selection bias)	Low risk	Authors state that randomisation "was performed at a central location".
Blinding of participants and personnel (performance bias)	Low risk	Double-blind placebo controlled
Blinding of outcome assessment (detection bias)	Low risk	Author states "sponsor [Millenium Pharmaceuticals] collected and analyzed the data".
Incomplete outcome data (attrition bias)	Low risk	According to the flowchart of Supplementary Figure 1 (S1), attrition was balanced in<br>all groups with adequate reasons provided for loss in numbers.
Selective reporting (reporting bias)	Low risk	Relevant outcomes reported per trial registration
Other bias	Low risk	Baseline characteristics reported and balanced for patients in all groups. No other apparent sources of bias.

Sandborn 2014

Bias	Authors' judgement	Support for judgement
Random sequence generation (selection bias)	Unclear risk	No details on randomisation
Allocation concealment (selection bias)	Low risk	The authors state allocation was concealed and electronically generated blinded codes were used which could be broken only in emergency situations for reasons of patient safety
Blinding of participants and personnel (performance bias)	Low risk	The study was placeco controlled, patient-, investigator-, and sponsor-blinded.
Blinding of outcome assessment (detection bias)	Low risk	The study was placeco controlled, patient-, investigator-, and sponsor-blinded.
Incomplete outcome data (attrition bias)	Low risk	Balanced and explained attrition
Selective reporting (reporting bias)	Low risk	Outcomes reported per the trial registration (NCT00615199)
Other bias	Low risk	No major baseline imbalances

Sandborn 2020d (CELEST) - Induction

Bias	Authors' judgement	Support for judgement
Random sequence generation (selection bias)	Low risk	"Centrally randomized using interactive response technology". We contacted the authors for clarification on the methods used: "at baseline, patients were randomized (1:1:1:1:1:1) to receive double-blind, 16-week induction treatment with placebo or the immediate-release formulation of upadacitinib 3-mg, 6-mg, 12-mg, or 24-mg twice daily or 24-mg once-daily oral doses. Patients were equally randomized (1:1) for the followup ileocolonoscopy at either week 12 or 16 for evaluation of the optimal timing of endoscopic assessment for future studies. Patients were centrally randomized using interactive response technology (IRT)."
Allocation concealment (selection bias)	Low risk	"Centrally randomized using interactive response technology".

Blinding of participants and personnel (performance bias)	Low risk	Authors state "patients, investigators, site coordinators, and sponsor were blinded to treatment assignment". We contacted the authors for more information: "Both the induction and maintenance treatments were double-blinded. The patients, investigators, site coordinators, and sponsor were blinded to treatment assignment. All study drug kits would have had the same appearance and would have been dispensed according to the IRT system. No study related personnel had unblinded access to the IRT and did not know what treatment was given to the patient."
Blinding of outcome assessment (detection bias)	Low risk	We contacted the authors for clarification: "Ileocolonoscopies performed during screening, week 12 or 16, and week 52 for the SES-CD were centrally read for eligibility and for the efficacy assessments by readers who were blinded to patient data and timepoints."
Incomplete outcome data (attrition bias)	Low risk	According to the flowchart of Supplementary Figure 2 (S2), attrition was balanced in all groups with adequate reasons provided for loss in numbers.
Selective reporting (reporting bias)	Low risk	Relevant outcomes reported per trial registration
Other bias	Low risk	Baseline characteristics reported and balanced for patients in all groups. No other apparent sources of bias.

Sandborn 2022 (GALAXI 1)

Bias	Authors' judgement	Support for judgement
Random sequence generation (selection bias)	Low risk	Author response after our enquiry:<br>"Central randomization will be implemented in GALAXI 1. Participants will be randomly assigned to 1 of 5 treatment groups (1:1:1:1:1 ratio), based on a computer-generated randomization schedule prepared before the studies by or under the supervision of the sponsor. The randomization will be balanced by using randomly permuted blocks and will be stratified by baseline CDAI score (≤ 300 or >300) and BIO-Failure status (Yes/No). The IWRS will assign a unique treatment code, which will dictate the treatment assignment and matching study intervention kit(s) for the participant. The requestor must use his or her own user identification and personal identification number when contacting the IWRS and will then be given the relevant participant details to uniquely identify the participant."

Allocation concealment (selection bias)	Low risk	Author response after our enquiry:<br>"Central randomization will be implemented in GALAXI 1. Participants will be randomly assigned to 1 of 5 treatment groups (1:1:1:1:1 ratio), based on a computer-generated randomization schedule prepared before the studies by or under the supervision of the sponsor. The randomization will be balanced by using randomly permuted blocks and will be stratified by baseline CDAI score (≤300 or >300) and BIO-Failure status (Yes/No). The IWRS will assign a unique treatment code, which will dictate the treatment assignment and matching study intervention kit(s) for the participant. The requestor must use his or her own user identification and personal identification number when contacting the IWRS and will then be given the relevant participant details to uniquely identify the participant."
Blinding of participants and personnel (performance bias)	Low risk	Author response after our enquiry:<br>All participants will continue to receive active or placebo study intervention administration in the LTE in a blinded fashion until study unblinding, which will occur after the Week 48 DBL and the Week 48 analyses have been completed for the Phase 2 study (for participants entering the LTE from GALAXI 1)<br>· Clinical assessors are blinded to treatment assignment and endoscopic assessors are blinded to treatment assignment, patient IDs and study visits.<br>· In GALAXI 1, the sponsor will remain blinded until the Week 48 DBL. However, a limited number of sponsor personnel will become unblinded at the Week 12 DBL for a dosing decision after the first 250 randomized participants have either completed the Week 12 visit or have terminated study participation<br>before Week 12. If a dosing decision cannot be made, a subsequent DBL will occur after the first 250 randomized participants have either completed the Week 24 visit or have terminated study participation before Week 24. At the time of each analysis, selected sponsor personnel will be unblinded for all randomized participants from the Initial Dose Decision Cohort and for any available participants from the Transition Cohort. After the Week 48 DBL when all randomized participants (including both the Initial Dose<br>Decision Cohort and the Transition Cohort) have either completed the Week 48 visit or have terminated study participation before Week 48, the treatment assignment information will be unblinded for all participants and released to the sponsor for analysis.

Blinding of outcome assessment (detection bias)	Low risk	Author response after our enquiry:<br>All participants will continue to receive active or placebo study intervention administration in the LTE in a blinded fashion until study unblinding, which will occur after the Week 48 DBL and the Week 48 analyses have been completed for the Phase 2 study (for participants entering the LTE from GALAXI 1)<br>· Clinical assessors are blinded to treatment assignment and endoscopic assessors are blinded to treatment assignment, patient IDs and study visits.<br>· In GALAXI 1, the sponsor will remain blinded until the Week 48 DBL. However, a limited number of sponsor personnel will become unblinded at the Week 12 DBL for a dosing decision after the first 250 randomized participants have either completed the Week 12 visit or have terminated study participation before Week 12. If a dosing decision cannot be made, a subsequent DBL will occur after the first 250 randomized participants have either completed the Week 24 visit or have terminated study participation before Week 24. At the time of

		each analysis, selected sponsor personnel will be unblinded for all randomized participants from the Initial Dose Decision Cohort and for any available participants from the Transition Cohort. After the Week 48 DBL when all randomized participants (including both the Initial Dose Decision Cohort and the Transition Cohort) have either completed the Week 48 visit or have terminated study participation before Week 48, the treatment assignment information will be unblinded for all participants and released to the sponsor for analysis.
Incomplete outcome data (attrition bias)	Low risk	Equal attrition and balanced reasons between groups
Selective reporting (reporting bias)	Low risk	All appropriate outcomes reported per trial registration and/or provided to us by the sponsor
Other bias	Low risk	Baseline characteristics reported and are balanced accross study arms. No other reasons for bias apparent

Sandborn 2023 - BERGAMOT (cohort 1)

Bias	Authors' judgement	Support for judgement
Random sequence generation (selection bias)	Low risk	An independent, interactive voice web-based response system provided by Parexel (Newton, MA) was used to generate the randomisation list and randomly assign patients to a treatment group
Allocation concealment (selection bias)	Low risk	An independent, interactive voice web-based response system provided by Parexel (Newton, MA) was used to generate the randomisation list and randomly assign patients to a treatment group
Blinding of participants and personnel (performance bias)	Low risk	Double-blinded placebo-controlled
Blinding of outcome assessment (detection bias)	Low risk	Central reading for endoscopic analysis
Incomplete outcome data (attrition bias)	Unclear risk	34% left the placebo group compared to 18% and 13% of the other two groups
Selective reporting (reporting bias)	Low risk	Appropriate outcomes reported per trial registration
Other bias	Unclear risk	The baseline characteristics for cohort 1 have not been presented.

Sandborn 2023 - BERGAMOT (cohort 3)

Bias	Authors' judgement	Support for judgement

Random sequence generation (selection bias)	Low risk	An independent, interactive voice web-based response system provided by Parexel (Newton, MA) was used to generate the randomisation list and randomly assign patients to a treatment group
Allocation concealment (selection bias)	Low risk	An independent, interactive voice web-based response system provided by Parexel (Newton, MA) was used to generate the randomisation list and randomly assign patients to a treatment group
Blinding of participants and personnel (performance bias)	Low risk	Double-blinded placebo-controlled
Blinding of outcome assessment (detection bias)	Low risk	Central reading for endoscopic analysis
Incomplete outcome data (attrition bias)	Low risk	No major attrition differences and reasons balanced.
Selective reporting (reporting bias)	Low risk	Appropriate outcomes reported per trial registration
Other bias	Low risk	No baseline differences. No other concerns

Sands 2004 (ACCENT II) Induction (Non-responders)

Bias	Authors' judgement	Support for judgement
Random sequence generation (selection bias)	Low risk	A computer-generated adaptive randomization scheme was used, which included the study site, the number of draining fistulas at baseline (one vs. more than one), and the presence or absence of active bowel disease at baseline (active bowel disease was considered to be present if the Crohn's Disease Activity Index was at least 150) as stratification factors.
Allocation concealment (selection bias)	Low risk	A pharmacist prepared each infusion of infliximab or an identical appearing placebo. Neither the patients nor the study investigators were aware of the treatment assignment. Crossovers were masked so that patients and physicians remained unaware of the treatment assignment.
Blinding of participants and personnel (performance bias)	Low risk	A pharmacist prepared each infusion of infliximab or an identical appearing placebo. Neither the patients nor the study investigators were aware of the treatment assignment. Crossovers were masked so that patients and physicians remained unaware of the treatment assignment.
Blinding of outcome assessment (detection bias)	Unclear risk	Not mentioned
Incomplete outcome data (attrition bias)	Low risk	Low and balanced attrition, that did not affect outcomes
Selective reporting (reporting bias)	High risk	The reported outcomes have shifted from those published in the trial registration (NCT00207766)
Other bias	Low risk	Baseline characteristics are balanced across the study arms. No other sources of bias are apparent

Sands 2007

Bias	Authors' judgement	Support for judgement
Random sequence generation (selection bias)	Unclear risk	Not mentioned
Allocation concealment (selection bias)	Unclear risk	Not mentioned
Blinding of participants and personnel (performance bias)	Low risk	Author states "placebo was identical in appearance to natalizumab".
Blinding of outcome assessment (detection bias)	Unclear risk	Not mentioned
Incomplete outcome data (attrition bias)	Low risk	Attrition was accounted for and balanced in both groups.
Selective reporting (reporting bias)	Unclear risk	Authors reported results according to the method section - CDAI score. The trial registration has no planned outcomes.
Other bias	Low risk	Baseline characteristics reported for both groups and balanced. No other apparent sources of bias.

Sands 2010

Bias	Authors' judgement	Support for judgement
Random sequence generation (selection bias)	Low risk	Randomization was performed centrally using a computer-generated randomization scheme and an integrated-voice-response system (IVRS) to assign patients to treatment arms or placebo. Patients were stratified according to C-reactive protein (CRP) levels (≥ 10 or <10 mg/L) and concurrent corticosteroid use (yes or no response). A permuted block design was employed using a 1:1:1 allocation to treatment arms and placebo. Enrollment of patients with CRP levels <10 mg/L was capped at 40% and patients were distributed evenly between groups.
Allocation concealment (selection bias)	Low risk	Central allocation
Blinding of participants and personnel (performance bias)	Low risk	Authors state 'Neither the patients nor the study investigators were aware of the group assignments'.
Blinding of outcome assessment (detection bias)	Low risk	They state 'Neither the patients nor the study investigators were aware of the group assignments'.
Incomplete outcome data (attrition bias)	Low risk	Balanced attrition

Selective reporting (reporting bias)	Low risk	All outcomes are reported
Other bias	Low risk	Baseline characteristics are balanced accross study arms. No other sources of bias apparent

Sands 2014 (GEMINI III)

Bias	Authors' judgement	Support for judgement
Random sequence generation (selection bias)	Low risk	Author state "randomization was computer-generated centrally".
Allocation concealment (selection bias)	Low risk	Author states as "patient enrollment, monitored by an interactive voice response system" and "treatment-qualified patient received a unique randomization number used to provide treatment assignments for dose preparation via the interactive voice response system. Salinebag covers and labels maintained blinding. Only the study site pharmacist was aware of treatment assignments".
Blinding of participants and personnel (performance bias)	Low risk	Author states "treatment-qualified patient received a unique randomization number used to provide treatment assignments for dose preparation via the interactive voice response system. Salinebag covers and labels maintained blinding. Only the study site pharmacist was aware of treatment assignments".
Blinding of outcome assessment (detection bias)	Low risk	Only the study site pharmacist was aware of treatment assignments.
Incomplete outcome data (attrition bias)	Low risk	Balanced attrition. Reasons are not explicitly stated but can be inferred from the text.
Selective reporting (reporting bias)	Low risk	According to trial registration and method section author reported relevant outcomes - proprotion of patients in clinical remission and response (CDAI scores).
Other bias	Low risk	Baseline characteristics reported for and balanced in all groups. No other apparent sources of bias.

Sands 2022 (SEAVUE)

Bias	Authors' judgement	Support for judgement
Random sequence generation (selection bias)	Low risk	Randomly assigned (1:1) via an interactive web response system to receive ustekinumab or adalimumab
Allocation concealment (selection bias)	Low risk	Concealed allocation was done via computer-generated randomisation schedule, managed by an independent vendor under supervision by the study funder
Blinding of participants and personnel (performance bias)	Low risk	Patients, investigators, and study site personnel were masked to treatment group assignment.<br>Placebo was administered as necessary so that all patients received the same number of<br>infusions and injections at the same timepoints to maintain blinding. Ustekinumab and<br>adalimumab syringes were not identical but were packaged identically, and study site personnel<br>did not see syringes out of containers. At day 0 and 2 weeks, a non-masked site<br>employee who was not part of the study team administered the study treatments and trained<br>patients for at-home administration after week 2. Efficacy and safety assessments were done by<br>masked study personnel
Blinding of outcome assessment (detection bias)	Low risk	Patients, investigators, and study site personnel were masked to treatment group assignment.<br>Placebo was administered as necessary so that all patients received the same number of<br>infusions and injections at the same timepoints to maintain blinding. Ustekinumab and<br>adalimumab syringes were not identical but were packaged identically, and study site personnel<br>did not see syringes out of containers. At day 0 and 2 weeks, a non-masked site<br>employee who was not part of the study team administered the study treatments and trained<br>patients for at-home administration after week 2. Efficacy and safety assessments were done by<br>masked study personnel.
Incomplete outcome data (attrition bias)	Low risk	Small difference in attrition rates and reasons, not likely to have influenced outcomes
Selective reporting (reporting bias)	Low risk	According to the trial registration the authors reported the relevant outcome measures

Other bias	Low risk	No baseline differences. No other concerns

Sands 2022 (SERENITY)

Bias	Authors' judgement	Support for judgement
Random sequence generation (selection bias)	Low risk	"Computer-generated random sequence using an interactive web-response system"
Allocation concealment (selection bias)	Low risk	"Computer-generated random sequence using an interactive web-response system."<br>"A study site pharmacist or other trained person was unblinded at the site for investigational product preparation."
Blinding of participants and personnel (performance bias)	Low risk	Desribed as double-blind.
Blinding of outcome assessment (detection bias)	Unclear risk	No details apart from endoscopy which was centrally read.
Incomplete outcome data (attrition bias)	Low risk	Equal attrition between groups and reasons provided and balanced.
Selective reporting (reporting bias)	Low risk	Outcomes appropriately reported per the trial registration (NCT02891226)
Other bias	Low risk	Baseline characteristics similar between groups.

Schreiber 2005

Bias	Authors' judgement	Support for judgement
Random sequence generation (selection bias)	Low risk	Author states "randomization code was prepared by an independent statistician"
Allocation concealment (selection bias)	Low risk	Authors state "patients were assigned to treatment by the use of a randomization allocation schedule managed via an interactive voice response system".
Blinding of participants and personnel (performance bias)	High risk	Author states "certolizumab and placebo did not have the same color or viscosity, full blinding<br>was not possible" but also mentioned "Consequently, patients received their treatment from a nurse or physician who was not involved in the study. All other staff involved in the study remained blind to treatment".

Blinding of outcome assessment (detection bias)	Low risk	Authors did state "All other staff involved in the study remained blind to treatment"
Incomplete outcome data (attrition bias)	Low risk	Attrition balanced between groups and reasons given
Selective reporting (reporting bias)	Low risk	Outcomes have been reported per trial registration
Other bias	Low risk	Baseline characteristics reported for and balanced for all groups. No other apparent sources of bias.

Schreiber 2018a

**Bias**	**Authors' judgement**	**Support for judgement**
Random sequence generation (selection bias)	Unclear risk	Not mentioned
Allocation concealment (selection bias)	Unclear risk	Not mentioned
Blinding of participants and personnel (performance bias)	Low risk	Authors state "Treatments were provided as single-use 150-mg/ml [1-ml] prefilled syringes with matching placebo"
Blinding of outcome assessment (detection bias)	Low risk	Centrally-read colonoscopy
Incomplete outcome data (attrition bias)	Low risk	Attrition accounted for and balanced in all groups, with adequate reasons provided respectively.
Selective reporting (reporting bias)	Low risk	According to trial registration and method section, authors reported relevant endpoint outcomes - proportion of patients with clinical remission (CDAI score) and endoscopic response (SES-CD score)
Other bias	Low risk	Baseline characteristics reported for and balanced in all groups. No other apparent source of bias.

Schroder 2006

Bias	Authors' judgement	Support for judgement
Random sequence generation (selection bias)	Unclear risk	"Not described in published study"
Allocation concealment (selection bias)	Unclear risk	"Not described in published study"
Blinding of participants and personnel (performance bias)	High risk	"Open-label"
Blinding of outcome assessment (detection bias)	High risk	"Open-label"
Incomplete outcome data (attrition bias)	Low risk	"2/8 receiving infliximab alone and 4/11 receiving infliximab plus methotrexate discontinued therapy by the end of the trial. The sole reason leading to the discontinuation of study treatment in both groups was lack of efficacy"
Selective reporting (reporting bias)	Low risk	"All outcomes were reported"
Other bias	Low risk	"No other issues"

SEQUENCE

Bias	Authors' judgement	Support for judgement
Random sequence generation (selection bias)	Unclear risk	No details (no full publication yet).
Allocation concealment (selection bias)	Unclear risk	No details (no full publication yet).
Blinding of participants and personnel (performance bias)	High risk	Single-blinded study (Outcomes Assessor)
Blinding of outcome assessment (detection bias)	Low risk	The site's investigator and personnel were blinded to CDAI and centrally read endoscopy scores during the study. Central reader for SES-CD was blinded to study treatment
Incomplete outcome data (attrition bias)	Unclear risk	90.2% completed the risenkizumab arm compared to 72.8% for ustekinumab. No other details yet.
Selective reporting (reporting bias)	Unclear risk	Clinical remission rates for week 24 have only been presented for 50% of randomised patients.
Other bias	Low risk	No baseline differences. No other concerns.

Summers 1979

Bias	Authors' judgement	Support for judgement
Random sequence generation (selection bias)	Unclear risk	Not described
Allocation concealment (selection bias)	Low risk	Centralized randomization
Blinding of participants and personnel (performance bias)	Low risk	Double-blinded, identically matched "all prepared in uncoated tablets of identical external and internal appearance"
Blinding of outcome assessment (detection bias)	Low risk	Double-blinded, identically matched "all prepared in uncoated tablets of identical external and internal appearance"
Incomplete outcome data (attrition bias)	Unclear risk	Attrition appears to be low (possible secondary to the fact that reasons for exiting the study were included in the "Outcome Ranking Scheme") A subset of patients were randomized and then excluded after study completion (justification was wrong diagnosis or inappropriate entry)
Selective reporting (reporting bias)	Low risk	All outcomes described for each part and phase were reported
Other bias	Low risk	The study appears to be free of other sources of bias

Targan 1997

Bias	Authors' judgement	Support for judgement
Random sequence generation (selection bias)	Low risk	Authors state "Randomization was performed centrally by an independent organization (PPDPharmaco, Austin, Tex.)"
Allocation concealment (selection bias)	Low risk	Authors state "Randomization was performed centrally by an independent organization (PPDPharmaco, Austin, Tex.)"
Blinding of participants and personnel (performance bias)	Low risk	Author states "The investigators, all other study personnel, and the patients were blinded to the treatment assignments"
Blinding of outcome assessment (detection bias)	Low risk	Author states "The investigators, all other study personnel, and the patients were blinded to the treatment assignments"

Incomplete outcome data (attrition bias)	Low risk	No attrition
Selective reporting (reporting bias)	Unclear risk	Outcomes reported per the trial registration. However, the safety data include nonrandomised patients.
Other bias	Low risk	Baseline characteristics were reported for and balanced in all groups. No other apparent sources of bias.

Targan 2007 (ENCORE)

Bias	Authors' judgement	Support for judgement
Random sequence generation (selection bias)	Low risk	Authors state "Randomization was stratified by site, and assignment to treatment was centrally performed"
Allocation concealment (selection bias)	Low risk	Authors state "Randomization was stratified by site, and assignment to treatment was centrally performed"
Blinding of participants and personnel (performance bias)	Low risk	Authors state "The patients, site staff, and study investigators were all blinded to treat-ment assignment."
Blinding of outcome assessment (detection bias)	Low risk	Authors state "The patients, site staff, and study investigators were all blinded to treat-ment assignment."
Incomplete outcome data (attrition bias)	Low risk	Attrition in both groups accounted for and balanced with adequate reasons provided.
Selective reporting (reporting bias)	Unclear risk	According to method section, authors reported relevant and planned outcome data - porportion of patients in clinical remission or response. However, the trial registration has no planned outcomes.
Other bias	Low risk	Baseline characteristics were reported for and balanced in all groups. No other apparent sources of bias.

Vermeire 2017 FITZROY - induction

Bias	Authors' judgement	Support for judgement

Random sequence generation (selection bias)	Low risk	A prespecified randomisation scheme prepared by an independent statistician was used to randomly allocate patients to treatment groups. Patients were stratified according to previous anti-TNF exposure, C-reactive protein concentration at screening (≤ 10 mg/L or >10 mg/L), and oral corticosteroid use at baseline, using an interactive web-based response system.
Allocation concealment (selection bias)	Low risk	For each patient at each visit, the clinic contacted the interactive web-based response system to obtain a treatment number corresponding to the appropriate study drug.
Blinding of participants and personnel (performance bias)	Low risk	Patients, investigators, study coordinators, the sponsor, and the entire study team were masked to treatment assignment. Filgotinib and placebo were presented as orally administered brown fi lmcoated tablets that were identical in appearance and contained the same excipients
Blinding of outcome assessment (detection bias)	Low risk	For the efficacy reads, assessment was done by two independent central readers in a masked fashion. In the case of discrepant results, a third, independent read by an adjudicator was done and was used as the final determination of efficacy read.
Incomplete outcome data (attrition bias)	Low risk	Balanced and mostly explained attrition for part 1 of this trial.
Selective reporting (reporting bias)	Low risk	All outcomes for part 1 of this trial reported per the trial registration (NCT02048618)
Other bias	Low risk	No baseline imbalances. No other concerns.

Vermeire 2017 FITZROY non-responders

Bias	Authors' judgement	Support for judgement
Random sequence generation (selection bias)	Low risk	A prespecified randomisation scheme prepared by an independent statistician was used to randomly allocate patients to treatment groups.
Allocation concealment (selection bias)	Low risk	For each patient at each visit, the clinic contacted the interactive web-based response system to obtain a treatment number corresponding to the appropriate study drug.
Blinding of participants and personnel (performance bias)	Low risk	Patients, investigators, study coordinators, the sponsor, and the entire study team were masked to treatment assignment. Filgotinib and placebo were presented as orally administered brown film-coated tablets that were identical in appearance and contained the same excipients

Blinding of outcome assessment (detection bias)	Low risk	For the efficacy reads, assessment was done by two independent central readers in a masked fashion. In the case of discrepant results, a third, independent read by an adjudicator was done and was used as the final determination of efficacy read.
Incomplete outcome data (attrition bias)	Low risk	Authors provided us with the data and it's balanced and the reasons explained
Selective reporting (reporting bias)	Unclear risk	The outcomes for non-responders are unclear
Other bias	Low risk	Authors provided us with the maintenance baseline data which are balanced and we have no other concerns

Vermeire 2025 (DIVERSITY A)

**Bias**	**Authors' judgement**	**Support for judgement**
Random sequence generation (selection bias)	Low risk	Randomisation was done by the investigator through an interactive web response system
Allocation concealment (selection bias)	Unclear risk	No mention.
Blinding of participants and personnel (performance bias)	Low risk	Everyone directly involved in the study conduct (including investigators, study personnel, and patients) was fully blinded to treatment allocation until the last patient completed the follow-up visit 30 days after completing 58 weeks of treatment. The appearance, packaging, and handling of active treatment (filgotinib 200 mg and filgotinib 100 mg) and placebo were identical to maintain blinding.
Blinding of outcome assessment (detection bias)	Low risk	All outcome assessors were blinded. Endoscopic central reading. Adverse events and clinical laboratory results were coded using the Medical Dictionary for Regulatory Activities version 25.0, and their severity was graded using the modified Common Terminology Criteria for Adverse Events version 4.03. An external, multidisciplinary data monitoring committee performed interim reviews of the safety data throughout the trial. All potential major adverse cardiovascular events (MACEs) and venous thromboembolic (VTE) events were reviewed and adjudicated periodically, and gastrointestinal perforation events were reviewed and adjudicated post hoc, in a blinded manner by an independent expert committee.
Incomplete outcome data (attrition bias)	Low risk	Balanced attrition and balanced reasons for withdrawals between groups.

Selective reporting (reporting bias)	Low risk	Outcomes appropriately reported per trial registration. NCT02914561
Other bias	Low risk	No concerns.

Vermeire 2025 (DIVERSITY B)

**Bias**	**Authors' judgement**	**Support for judgement**
Random sequence generation (selection bias)	Low risk	Randomisation was done by the investigator through an interactive web response system
Allocation concealment (selection bias)	Unclear risk	No mention.
Blinding of participants and personnel (performance bias)	Low risk	Everyone directly involved in the study conduct (including investigators, study personnel, and patients) was fully blinded to treatment allocation until the last patient completed the follow-up visit 30 days after completing 58 weeks of treatment. The appearance, packaging, and handling of active treatment (filgotinib 200 mg and filgotinib 100 mg) and placebo were identical to maintain blinding.
Blinding of outcome assessment (detection bias)	Low risk	All outcome assessors were blinded. Endoscopic central reading. Adverse events and clinical laboratory results were coded using the Medical Dictionary for Regulatory Activities version 25.0, and their severity was graded using the modified Common Terminology Criteria for Adverse Events version 4.03. An external, multidisciplinary data monitoring committee performed interim reviews of the safety data throughout the trial. All potential major adverse cardiovascular events (MACEs) and venous thromboembolic (VTE) events were reviewed and adjudicated periodically, and gastrointestinal perforation events were reviewed and adjudicated post hoc, in a blinded manner by an independent expert committee.
Incomplete outcome data (attrition bias)	Low risk	The filgotinib 200mg group had better retention but overall balanced attirition with balanced reasons between groups.
Selective reporting (reporting bias)	Low risk	Outcomes appropriately reported per trial registration. NCT02914561
Other bias	Low risk	No concerns.

Watanabe 2012 - Induction

Bias	Authors' judgement	Support for judgement
Random sequence generation (selection bias)	Unclear risk	Not described
Allocation concealment (selection bias)	Unclear risk	Not described
Blinding of participants and personnel (performance bias)	Low risk	Double-blinded study
Blinding of outcome assessment (detection bias)	Unclear risk	Not described
Incomplete outcome data (attrition bias)	Low risk	Attrition was accounted for and balanced in both groups with adequate reasons provided for loss in numbers.
Selective reporting (reporting bias)	Low risk	According to trial registration and method section authors reported the relevant endpoints - CDAI scores at specific timepoints.
Other bias	Low risk	Baseline characteristics were reported for and balanced in all groups. No other apparent sources of bias.

Watanabe 2020 - Induction

Bias	Authors' judgement	Support for judgement
Random sequence generation (selection bias)	Low risk	Author states "Randomization schedules were generated by personnel designated by the sponsor"
Allocation concealment (selection bias)	Low risk	Author states "allocations were not disclosed until opening of the study drug allocation table,<br>except to unblinded pharmacists at each site".
Blinding of participants and personnel (performance bias)	Low risk	Double-blinded study
Blinding of outcome assessment (detection bias)	Unclear risk	Not described
Incomplete outcome data (attrition bias)	Low risk	Attrition was accounted for and balanced in all groups with adequate reasons provided for loss in numbers.
Selective reporting (reporting bias)	Low risk	According to trial registration and method section authors reported the relevant endpoints -<br>proportion of patients with specific CDAI-100 response and clinical remission.
Other bias	Low risk	Baseline characteristics were reported for and balanced in all groups. No other apparent sources of bias.

Winter 2004

Bias	Authors' judgement	Support for judgement
Random sequence generation (selection bias)	Unclear risk	Not described
Allocation concealment (selection bias)	Unclear risk	Not described
Blinding of participants and personnel (performance bias)	Low risk	Double-blind placebo controlled
Blinding of outcome assessment (detection bias)	Unclear risk	Not described
Incomplete outcome data (attrition bias)	Low risk	Attrition accounted for in each group and adequate reasons provided per group.
Selective reporting (reporting bias)	Unclear risk	We could not find a published protocol or trial registration. According to method section, authors reported relevant endpoint outcomes - clinical response (CDAI scores).

Other bias	Low risk	Baseline characteristics reported for and balanced in all groups. No other apparent sources of bias.

Ye 2019

Bias	Authors' judgement	Support for judgement
Random sequence generation (selection bias)	Low risk	Author states "PPD Bioanalytical Laboratory Services (Bellshill, Scotland, UK) generated the randomisation schedule" so authors contacted and answered that they "generated randomization code by SAS, then linked to interactive voice response system for randomization schedule"
Allocation concealment (selection bias)	Low risk	Authors state "Randomisation codes were not revealed to patients, investigators, or centre personnel, except for predefined unblinded personnel from Celltrion and PPD, until all final clinical data were entered into the database and the database was locked and released for analysis". Authors were asked what the role of the unblinded personnel was and responded "Following our SOP, predefined members were unblinded for development of Week 6 CSR. It included person who was in charge of CSR developing, biostatistician, and who was in charge of regulatory affair (to submit final CSR to regulatory body).Minimum number of personnel was unblinded for reporting purpose. Other than those, people were blinded until study completion. When unblind was needed, all unblind process was approval and logged in writing form.
Blinding of participants and personnel (performance bias)	Low risk	Authors state "Randomisation codes were not revealed to patients, investigators, or centre personnel,except for predefined unblinded personnel from Celltrion and PPD, until all final clinical data were entered into the database and the database was locked and released for analysis". Authors also provided a clarification: "Site personnel and patient could not see their treatment arm fundamentally as they were physically separated from unblinded information."

Blinding of outcome assessment (detection bias)	Low risk	Authors state "Randomisation codes were not revealed to patients, investigators, or centre personnel,except for predefined unblinded personnel from Celltrion and PPD, until all final clinical data were entered into the database and the database was locked and released for analysis". Authors provided clarification: "Only pre-authorized person was unblinded for reporting purpose at the time of developing clinical study report. Only authorized person could access to unblinded folder. Blinded<br>person could not access to unblinded folder systemically. Other than authorized person could not access to unblinded data (positive regulation scheme. i.e., define unblinded person and prohibit anyone else). "
Incomplete outcome data (attrition bias)	Low risk	Attrition accounted for and balanced in all groups, with adequate reasons provided respectively
Selective reporting (reporting bias)	Low risk	According to trial registration and method section, authors reported relevant endpoint outcomes - clinical response and remission (CDAI scores).
Other bias	Low risk	Baseline characteristics were reported for and balanced in all groups. No other apparent sources of bias.
