# Supplementary material for: Immunomodulators and Advanced Therapies for Induction of Remission in Crohn’s Disease: A Systematic Review and Network Meta-Analysis
Source: Inflamm Bowel Dis. 2025 Sep 19;32(1):53–66. doi: 10.1093/ibd/izaf191 (PMC12759050; doi:10.1093/ibd/izaf191)

Clinical remission: Treatment vs. Placebo (Random Effects Model)

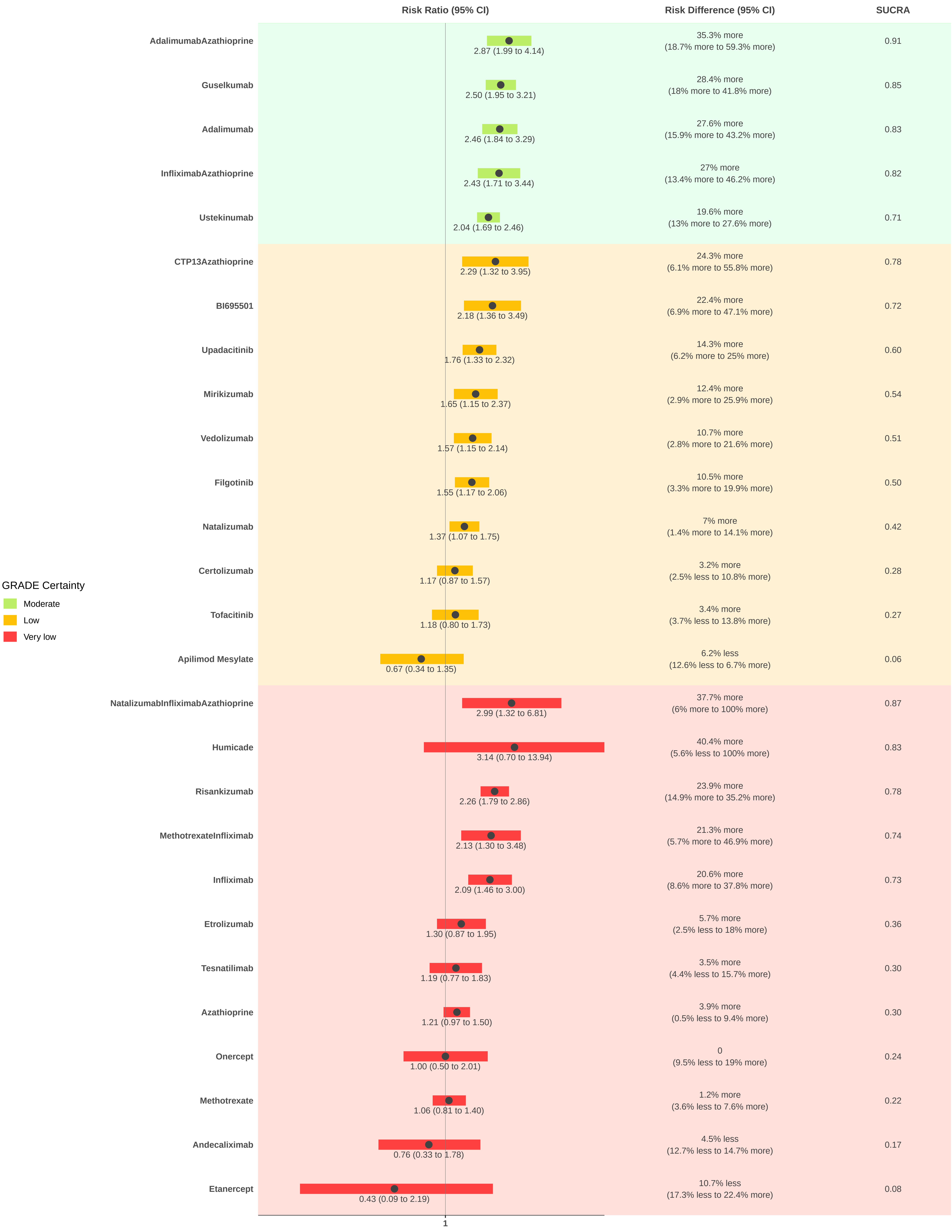

Clinical remission (50% or more naive): Treatment vs. Placebo (Random Effects Model)

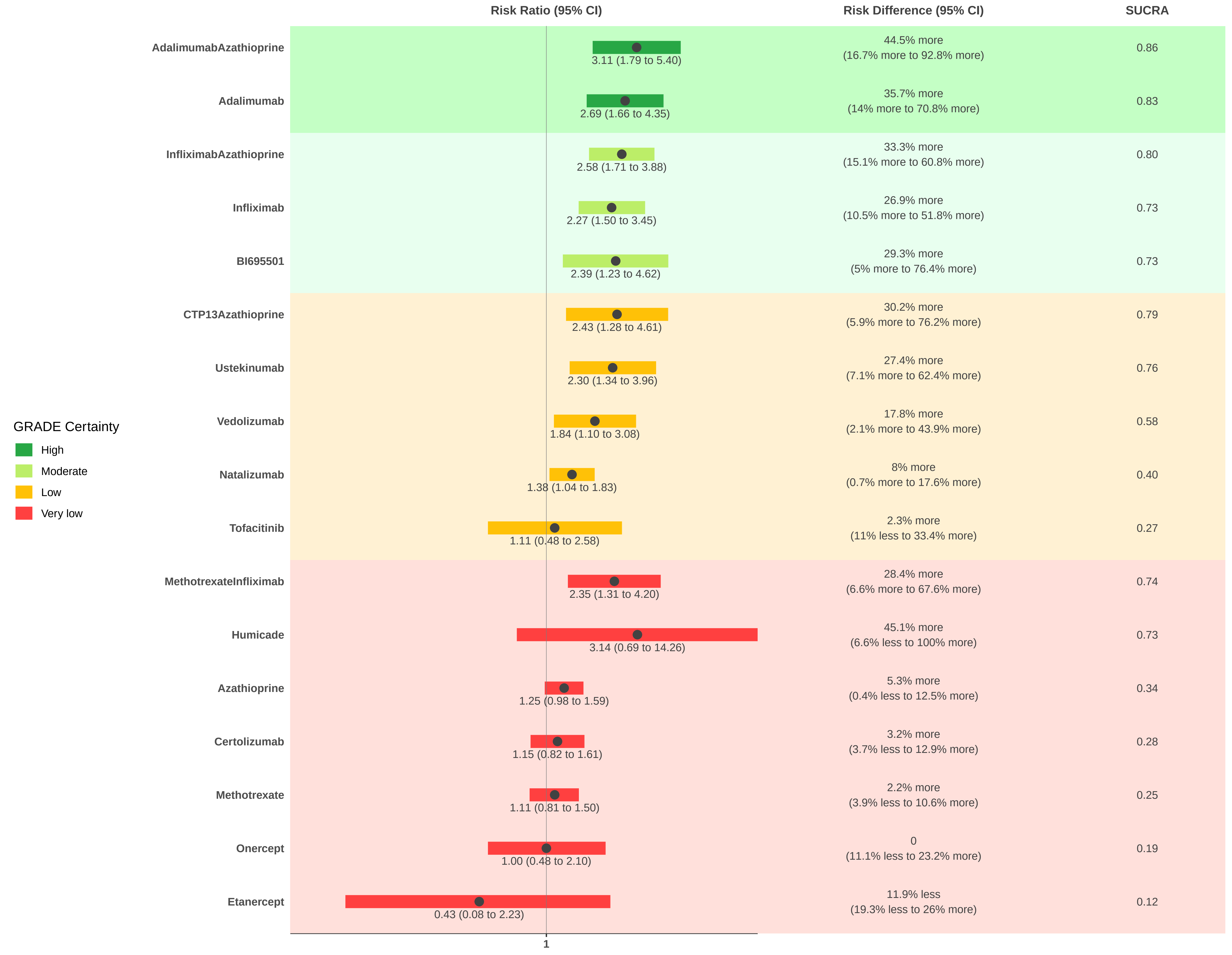

Clinical remission (50% or more exposed): Treatment vs. Placebo (Random Effects Model)

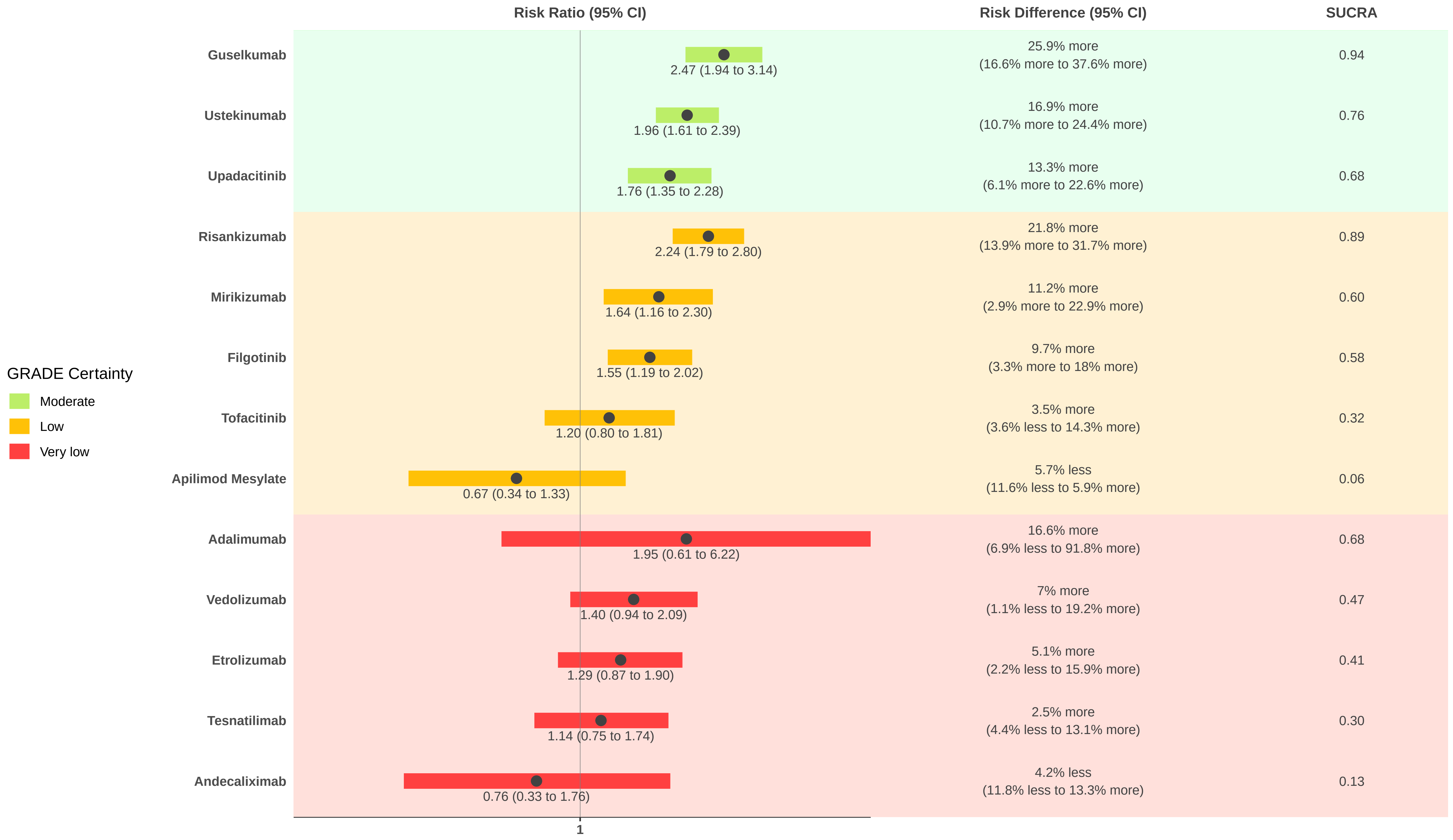

Clinical response: Treatment vs. Placebo (Random Effects Model)

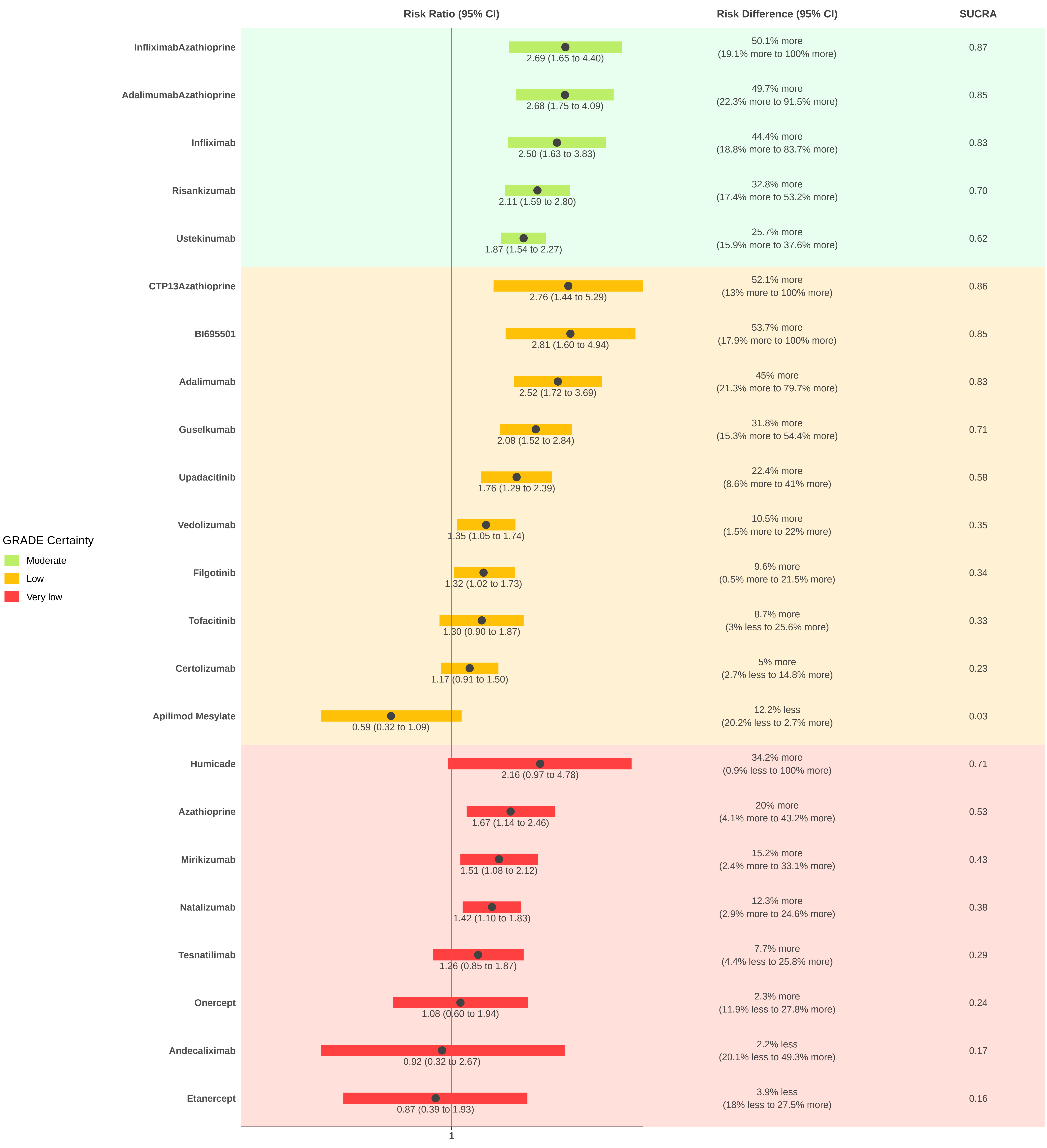

Endoscopic remission: Treatment vs. Placebo (Random Effects Model)

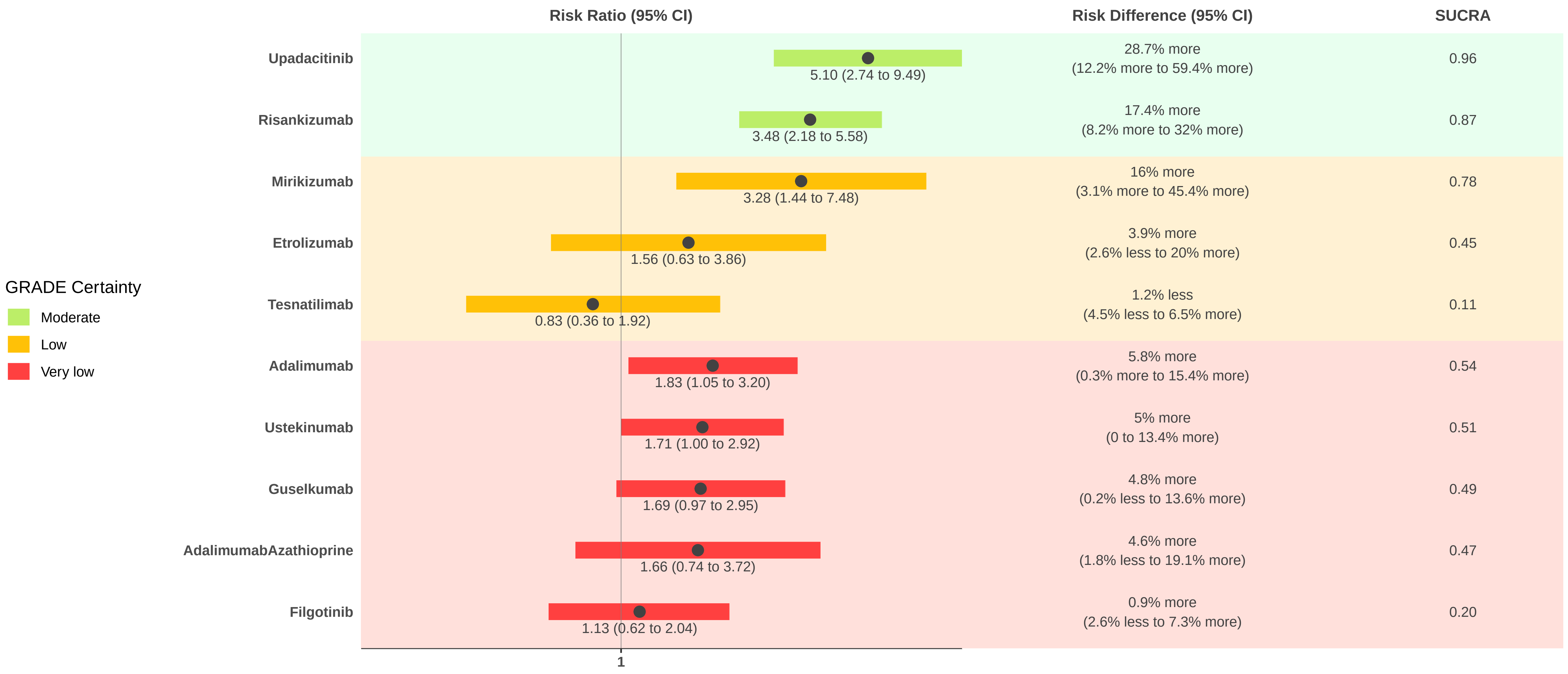

Withdrawals due to adverse events: Treatment vs. Placebo (Random Effects Model)

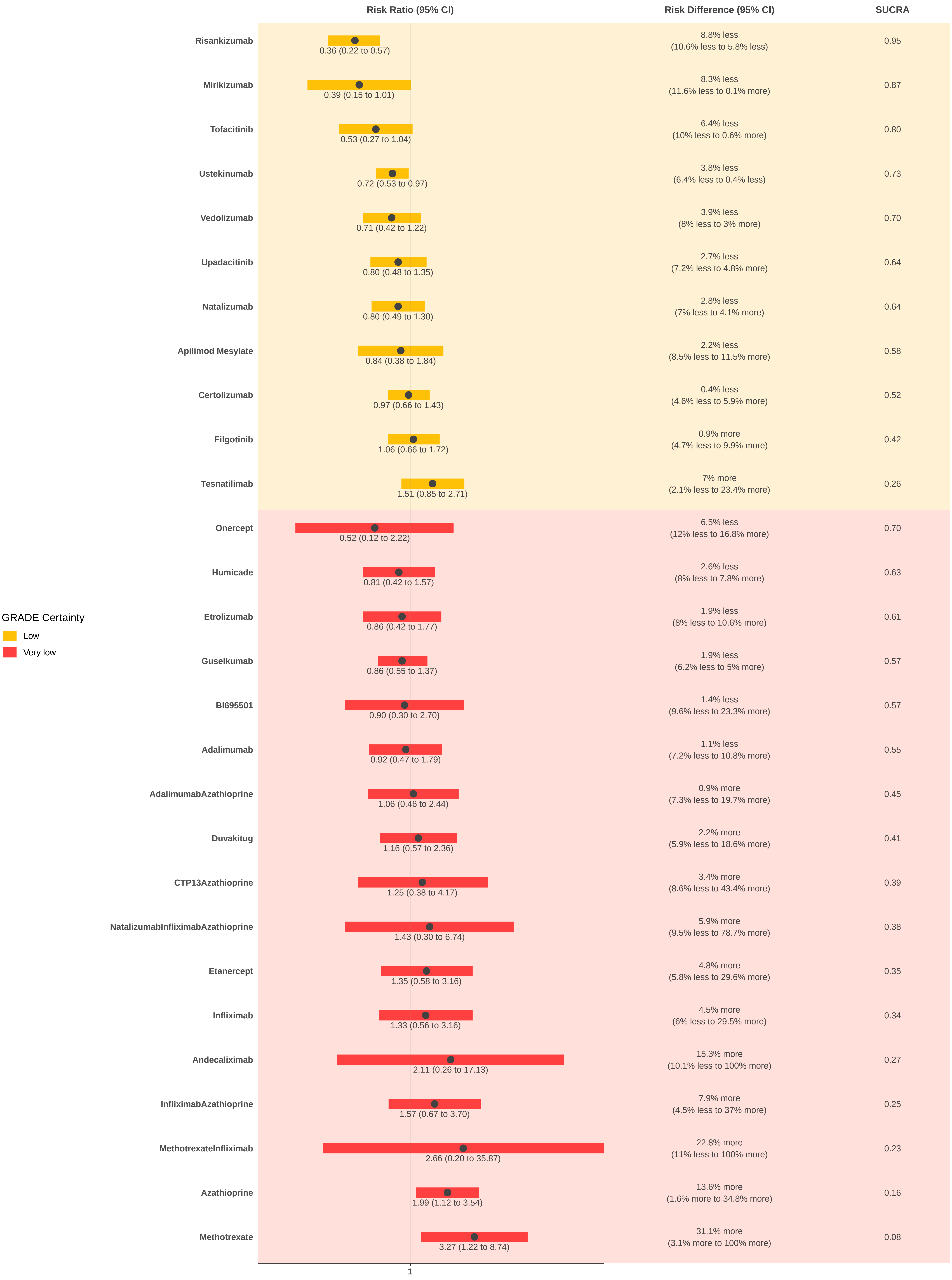

Serious adverse events: Treatment vs. Placebo (Random Effects Model)

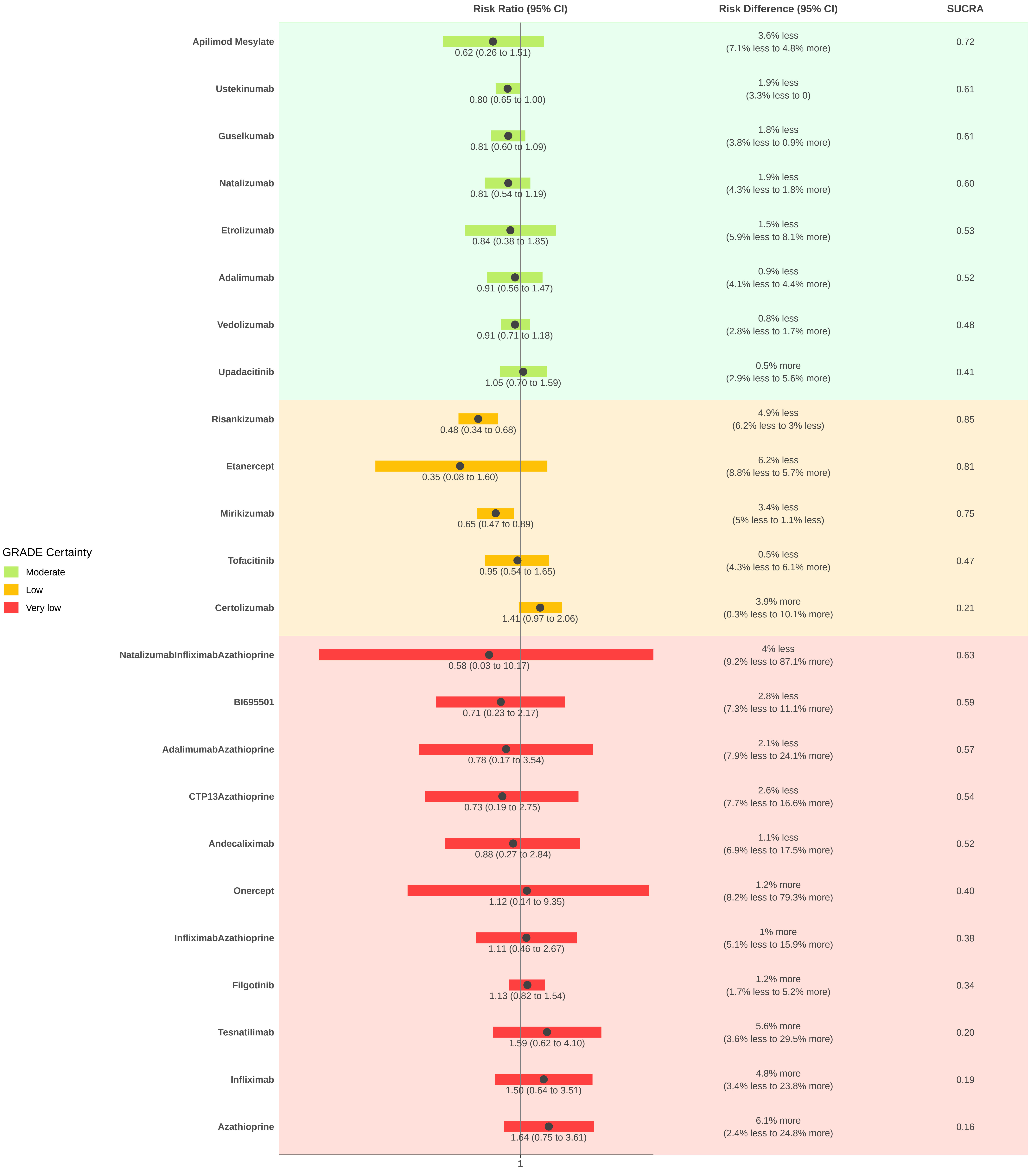

Total adverse events: Treatment vs. Placebo (Random Effects Model)

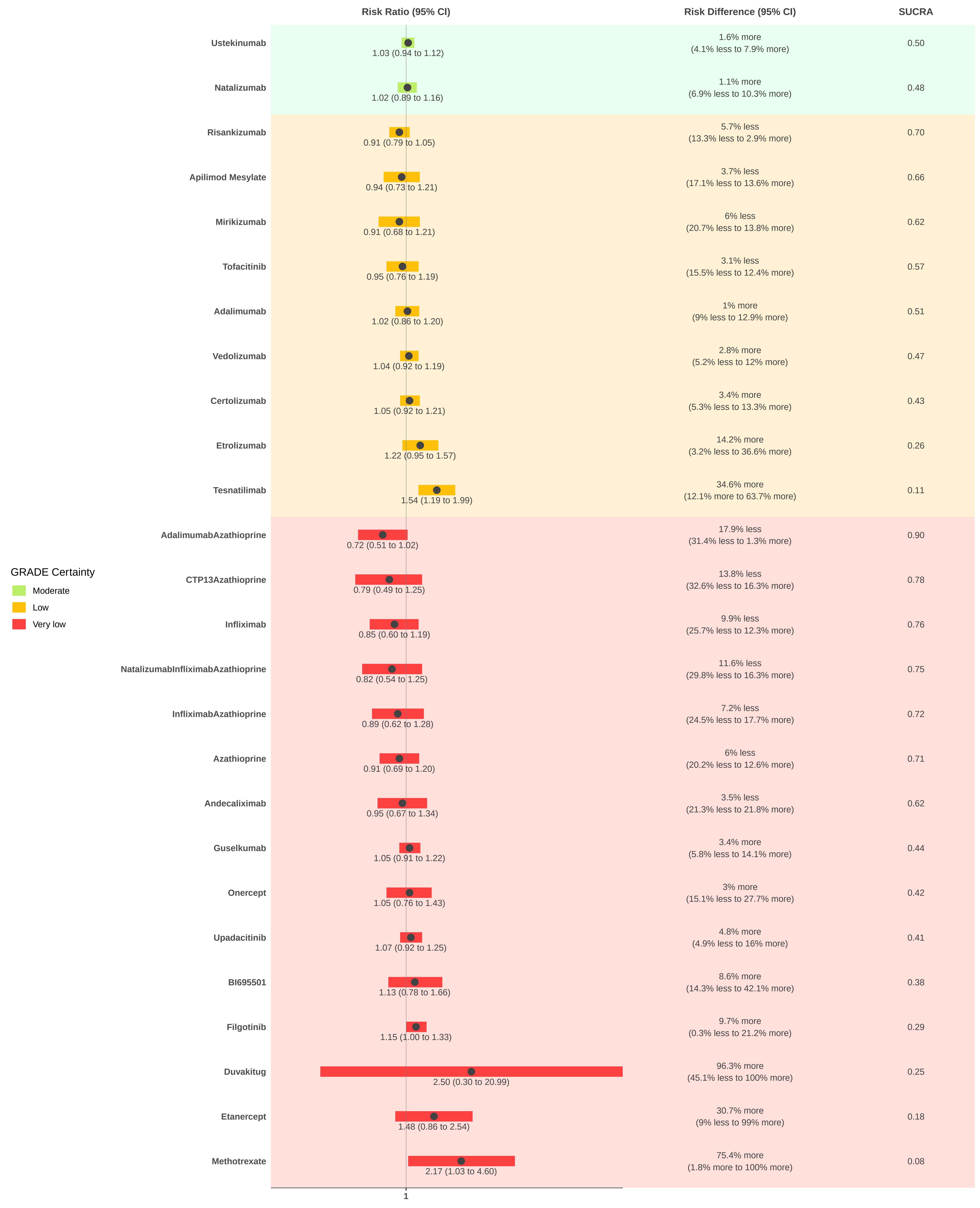

Supplement: izaf191_Supplementary_Data [file izaf191_supplementary_data.zip › Supplement 3 GORDON plots.pdf]
